# A Novel Coronavirus and a Broad Range of Viruses in Kenyan Cave Bats

**DOI:** 10.3390/v14122820

**Published:** 2022-12-17

**Authors:** Joseph Kamau, Koray Ergunay, Paul W. Webala, Silvia A. Justi, Brian P. Bourke, Maureen W. Kamau, James Hassell, Mary N. Chege, David K. Mwaura, Cynthia Simiyu, Sospeter Kibiwot, Samson Onyuok, Laura Caicedo-Quiroga, Tao Li, Dawn M. Zimmerman, Yvonne-Marie Linton

**Affiliations:** 1 One Health Centre, Institute of Primate Research (IPR), Nairobi 00502, Kenya; 2 Walter Reed Biosystematics Unit (WRBU), Smithsonian Institution Museum Support Center, Suitland, MD 20746, USA; 3 One Health Branch, Walter Reed Army Institute of Research (WRAIR), Silver Spring, MD 20910, USA; 4 Department of Medical Microbiology, Virology Unit, Faculty of Medicine, Hacettepe University, Ankara 06230, Turkey; 5 Department of Entomology, Smithsonian Institution–National Museum of Natural History (NMNH), Washington, DC 20560, USA; 6 Department of Forestry and Wildlife Management, Maasai Mara University, Narok 20500, Kenya; 7 Mpala Research Centre, Nanyuki 10400, Kenya; 8 Global Health Program, Smithsonian Conservation Biology Institute (SCBI), Front Royal, VA 22630, USA; 9 Department of Epidemiology of Microbial Disease, Yale School of Public Health, New Haven, CT 06520, USA; 10 International Livestock Research Institute (ILRI), Nairobi 00100, Kenya; 11 Zoology Department, National Museums of Kenya, Nairobi 00100, Kenya; 12 Viral Diseases Branch, Walter Reed Army Institute of Research, Silver Spring, MD 20910, USA

**Keywords:** coronavirus, astrovirus, retrovirus, bat, metagenome, Kenya, Africa

## Abstract

Background and Methods: To investigate virus diversity in hot zones of probable pathogen spillover, 54 oral-fecal swabs were processed from five bat species collected from three cave systems in Kenya, using metagenome sequencing. Results: Viruses belonging to the *Astroviridae*, *Circoviridae*, *Coronaviridae*, *Dicistroviridae*, *Herpesviridae* and *Retroviridae* were detected, with unclassified viruses. Retroviral sequences were prevalent; 74.1% of all samples were positive, with distinct correlations between virus, site and host bat species. Detected retroviruses comprised *Myotis myotis*, *Myotis ricketti*, *Myotis daubentonii* and Galidia endogenous retroviruses, murine leukemia virus-related virus and *Rhinolophus ferrumequinum* retrovirus (RFRV). A near-complete genome of a local RFRV strain with identical genome organization and 2.8% nucleotide divergence from the prototype isolate was characterized. Bat coronavirus sequences were detected with a prevalence of 24.1%, where analyses on the ORF1ab region revealed a novel alphacoronavirus lineage. Astrovirus sequences were detected in 25.9%of all samples, with considerable diversity. In 9.2% of the samples, other viruses including Actinidia yellowing virus 2, bat betaherpesvirus, Bole tick virus 4, Cyclovirus and Rhopalosiphum padi virus were identified. Conclusions: Further monitoring of bats across Kenya is essential to facilitate early recognition of possibly emergent zoonotic viruses.

## 1. Introduction

Spillover of a pathogen from the original source to intermediate or definitive recipient hosts often precede the onset of emerging infections [1]. For viruses potentially pathogenic for humans, the most probable source for such a spillover event is wildlife. Nevertheless, these events may involve various domesticated and non-domesticated animals before a zoonotic pathogen fully adapts to humans. Virus-host switching between non-human hosts is an early and crucial step on the path to disease emergence [2].

Bats are globally distributed mammals with high diversification [3,4]. They are documented to harbor several viruses including highly pathogenic agents, of which some are designated of pandemic concern by the World Health Organization [5]. Their distinctive ecological and biological properties including relatively long lifespans, capacity for sustained flight, hibernation and roosting behaviors, potential for persistent and asymptomatic shedding due to unique immune response potentially increase virus maintenance and transmission [6,7]. Bats are documented as hosts of viruses of significant human health threat including rabies, Hendra, Nipah and Ebola viruses [8]. They also harbor several coronaviruses including those responsible for severe acute respiratory syndrome (SARS) and Middle East respiratory syndrome (MERS) [9]. The causative agent of the COVID-19 pandemic is associated with a previously identified ancestral bat coronaviruses [10,11]. Hence, bats are optimal target species for screening virus diversity and potential agents with high spillover potential. Globally, anthropogenic environmental changes due to agriculture, industrial activities and urbanization have impacted bat populations, with increased probability of viral spillover into humans and animals [12,13]. 

Metagenome sequencing allows for an unbiased characterization of target nucleic acids in the sample and has become a powerful tool for identifying known and novel pathogens, especially viruses, in a wide range of samples [12,14]. It also enables detection of viruses that might be overlooked in amplification-based surveillance of established or probable pathogens. The increasing use of metagenome sequencing has facilitated characterization of many novel and highly diverse viruses in bats, considerably expanding the known virosphere and providing important information on the origin and distribution of particular viruses [12,14].

With highly diverse geography and climate, Kenya harbors a large diversity of wildlife species with potential as virus reservoirs, including several bat species [15,16]. Previous investigations in Kenyan bats have documented adenoviruses, astroviruses, caliciviruses, coronaviruses, rhabdoviruses, rotaviruses, paramyxoviruses and filoviruses [17,18,19,20,21,22]. Due to the targeted screening strategy employed in these studies, viral diversity could not be explored in full. Here, we aimed to carry out a metagenome-based screening for viruses in bats, to detect a broad range of viruses, better capturing virus diversity and probable co-infections. Moreover, our samplings targeted sites where bats and other human or non-human hosts are documented, representing locations where virus reservoirs and recipients overlap. 

## 2. Materials and Methods

### 2.1. Bat Capture, Handling and Sampling 

**Figure 1:**
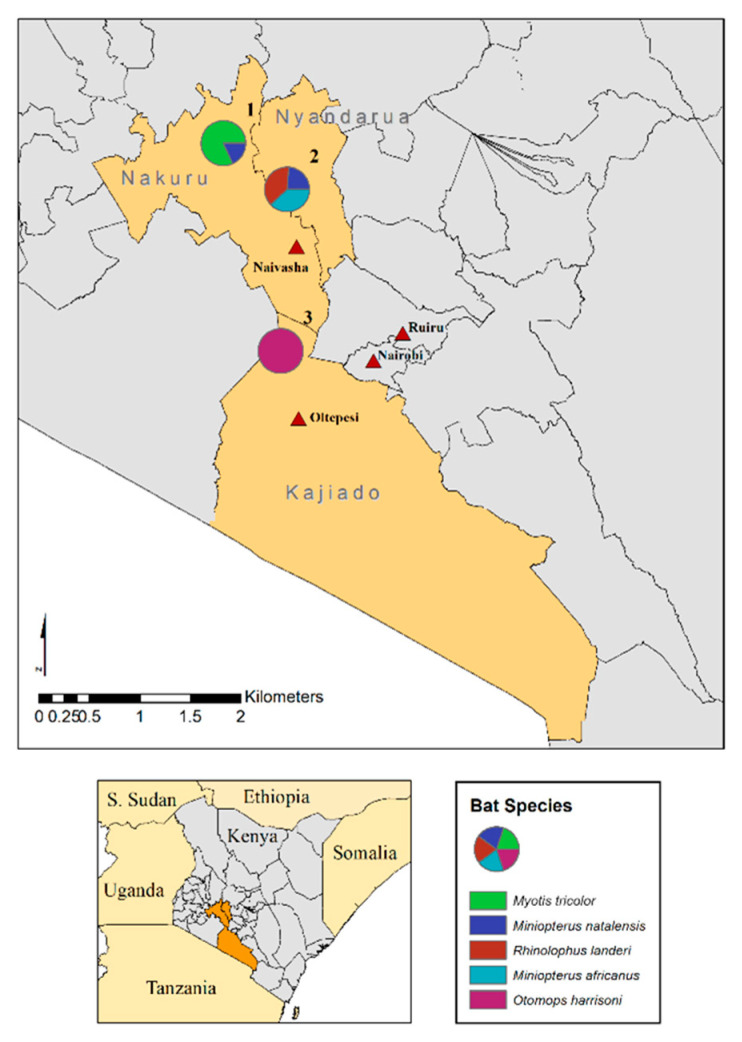
Map of the sites of the three bat caves and associated bat species sampled in this study: Menengai Crater (**1**) [*Miniopterus natalensis*, *Myotis tricolor*], Soysambu Conservancy (**2**) [*Miniopterus africanus, Miniopterus natalensis*, *Rhinolophus landeri*], Mount Suswa (**3**) [*Otomops harrisoni*].

As part of a collaboration between Maasai Mara University. Smithsonian Institution and the Institute of Primate Research of the National Museums of Kenya, bat samples were obtained from species captured in three cave systems in Kenya’s Rift Valley in 2020. The collection sites comprised the religious cave at Menengai Crater, an abandoned diatomite mine at Soysambu Conservancy and the volcanic tunnels and caves at Mount Suswa (Figure 1). 

The bats were captured using mist and hand nets. The 6- and 12-m nylon mist nets were deployed in probable flyways and monitored continuously, typically from dusk to about midnight. Mist nets are useful in identifying areas used by bats in foraging or commuting while hand nets are best employed to capture bats that roosts in caves, mines, and tunnels. The latter method is particularly informative as it sheds light on the social groupings as well as roosting preferences of bats. Captured bats were placed in individual cloth bags (to prevent cross-contamination) and transported to preparation areas, where they were examined, measured and identified to species by a trained field biologist. Subsequently, oral and rectal swabbing were performed in compliance with field protocol and the samples were transported in Trizol (ThermoFisher Scientific, MA, USA) on dry ice from the field to −80 °C storage before further processing. Oral and rectal swabs from individual bats were pooled prior to further processing. Sampled bats were released following sample collection. No bats were excluded from the study. The study protocol was approved by the National Zoological Park Institutional Animal Care and Use Committee (NZP-IACUC #20-02) and by Kenya Wildlife Service. Sample collection, experiments and reporting of the results follow the recommendations described in the ARRIVE guidelines. All collection, sampling and subsequent experiments were performed in accordance with these guidelines and approved protocols.

### 2.2. Sample Processing, Metagenome Sequencing and Data Analysis

Nucleic acid purification was conducted using the Direct-zol RNA MiniPrep Kits (Zymo Research USA) at IPR and purified nucleic acids were converted to complementary DNA (cDNA) using the RevertAid First Strand cDNA Synthesis Kit (Thermo Fisher Scientific), according to the manufacturers’ recommendations. The cDNA was quantified using a NanoDropTM 2000/2000c Spectrophotometer (Thermofisher Scientific) and samples were shipped to the Walter Reed Biosystematics Unit (WRBU, Suitland, MD, USA) for second strand cDNA synthesis, library preparation and further downstream processing. Double-stranded cDNA was prepared using NEBNext Non-Directional RNA Second Strand Synthesis module, utilizing random primer mix (New England Biolabs, Ipswich, MA, USA) according to manufacturer recommendations. Library preparations were carried out using KAPA HyperPlus Kits (Roche, CA, USA), with a fragmentation step of 20 mins at 35 °C, ligation step of 90 mins and 20 cycles of amplification, as recommended by the manufacturer. Library quantification and quality control were performed using the TapeStation 4200 Automated Electrophoresis instrument (Agilent Technologies, VA, USA). Excess adapters and fragments were removed using KAPA pure beads (Roche, CA, USA). Unbiased metagenomic sequencing was performed on the NovaSeq platform (Illumina, CA, USA) (PE 2 × 150) at the Walter Reed Army Institute of Research (WRAIR), Silver Spring, MD, USA. Samples were run on one lane of a S4 flow cell with XP workflow, designed to maximize the overall raw reads output. Initial read quality assessment was carried out using fastqc [23].

Raw data from the sequencing runs were uploaded to the publicly accessible cloud-based CZ-ID platform (formerly ID-Seq) for metagenomic pathogen detection [24] (https://www.czid.org, accessed on 17 November 2022). Initial data analysis was performed using the built-in opensource pipeline (version 6.8), incorporating steps for validation, host and adaptor removal, quality assessment, alignment, assembly and taxonomic identification using National Center for Biotechnology Information (NCBI) nucleotide and protein databases. Briefly, adapter trimming and host filtration in different steps were carried out using Trimmomatic, STAR, Bowtie2 and GSNAP [25,26,27,28]. Viruses were considered significant when contigs de novo assembled with SPADES, were verified in the final output, via BLAST alignment against the BLAST nucleotide and protein databases constructed from taxa identified from initial GSNAP and RASsearch2 read alignment against NCBI nucleotide (nt) and protein (nr) databases, respectively. Viruses were then manually reviewed, and taxon identity confirmed by BLASTing against the full NCBI nt and nr databases [29]. The virus reads were mapped to reference genomes in Geneious Prime version 2022.0.2 built-in mapper with default settings. Alignment and pairwise comparisons of the nucleotide and deduced amino acid sequences were generated using CLUSTAL W [30]. Bayesian phylogenetic analysis was performed on sequences using IQ-TREE 2 [31]. Optimal evolutionary models and partitioning schemes were determined for nucleotide and amino acid sequence alignment using the automatic model selection tools (-mMFP + MERGE). Amino acid models were restricted to those designed for viral sequences (-msub viral). A 70% majority-rule consensus tree was constructed by maximum likelihood using 1000 replicates from the ultrafast bootstrap approximation approach (UFBoot) [32]. The UFBoot support values are more unbiased than normal bootstrap support and significant clade support are considered at ≥95%. Screening for recombinations among virus genomes was performed through algorithms implemented in the RDP4 software [33], using default settings. 

## 3. Results

Oral-rectal swabs from 54 cave-dwelling bats originating from Menengai Crater (n = 11, 20.3%), Soysambu Conservancy (n = 21, 38.8%) and Mount Suswa (n = 22, 40.7%) were individually sequenced in the study. Five bat species were sampled: *Otomops harrisoni* (n = 22, 40.7%), *Myotis tricolor* (n = 9, 16.6%), *Rhinolophus landeri* (n = 8, 14.8%), *Miniopterus africanus* (n = 8, 14.8%) and *Miniopterus natalensis* (n = 7, 12.9%). Viruses from six families or those yet to be classified were detected by metagenome sequencing. Bat species distribution and virus detection data according to sampling sites are provided in Table 1. Total reads and virus family-specific reads within the range of 1.3 × 10^5^–7.8 × 10^5^ and 0.4 × 10–5.2 × 10^4^ respectively, were documented in the samples. Individual sequencing statistics, bat biological measurements and virus detection data are provided in Table S1.

### 3.1. Coronaviruses

Coronavirus sequences were detected in 13 samples (24.1%) with 12 positives originated from Mount Suswa and a single individual of R. landeri tested positive at Soysambu Conservancy (Table 1). Of note, the prevalence of CoV in O. harrisoni bats was 54.5% (12/22).

**Figure 2:**
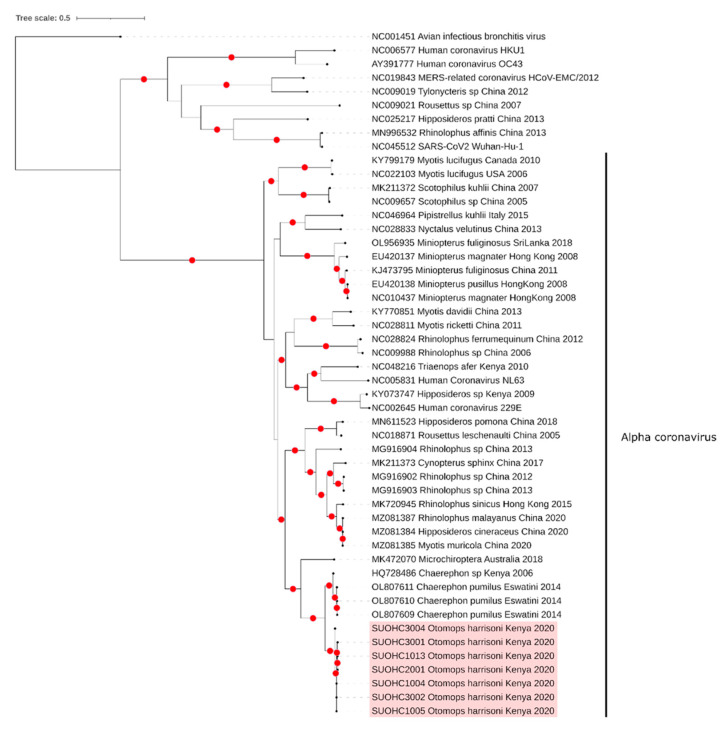
The Maximum Likelihood Consensus Tree Of The Coronavirus ORF1ab Sequences (8475 Nucleotides), Constructed Esing 1000 Replicates With Branches Achieving ≥95% Bootstrap Support Annotated By Red Dots.

We characterized ORF1ab sequences of 413–14,666 base pairs with 91.2–98.9% identities to bat alphacoronaviruses (Table S2). The deduced amino acid identities of the ORF1ab polyproteins revealed 85.8–98.9% pairwise identities to a recently reported bat alphacoronavirus described in little free-tailed bats (Chaerephon pumilus) from Eswatini [34]. The maximum likelihood analyses of nucleotide (Figure 2) and deduced amino acid sequence alignments (Figure S1) displayed a separate grouping of the sequences, forming a distinct lineage within alphacoronaviruses and sharing a common ancestor with viruses reported in bats from Eswatini and Kenya, with >97% amino acid identities [20,33]. Analysis of ORF1ab sequences (>8000 base pairs) failed to demonstrate any in silico evidence of recombination by all tools employed. Due to the lack of available biomaterial, we made no further attempt to complete the virus genome in any sample. All coronavirus consensus sequences are available as FASTA files (Data S1). Selected sequences were further deposited in GenBank with the accession numbers ON893136–ON893141. 

Further analysis of the coronavirus contigs in samples revealed segments of S, NS3b, E, M and N regions, encoding for the virus structural and other accessory proteins (Table S2). Sequences of the additional coding region—ORFx, involved in host immune modulation in bat coronaviruses [35], were present in six samples. The complete ORFx deduced amino acid sequences were identical in five samples, being 34.2% divergent from the closest sequence reported previously from Kenya (Figure S2). We could further characterize complete M regions in six samples, with limited intragroup diversity (Figure S2). Multiple segments covering virus spike S1′ and S2′ sections could be identified in a single sample, which revealed fusion peptide motifs and S2′ furin cleavage sites involved in processing (Figure S3). 

### 3.2. Retroviruses

Retroviral sequences were by far the most prevalent, being detected in 40 of the 54 bats (74.1%) sequenced. They were identified in varying prevalence in all sites (Menengai Crater: 9/11 or 81.8%; Soysambu Conservancy: 10/21 or 47.7%; Mount Suswa: 21/22 or 95.5%). Interestingly, host- and site-specificity was noted, with almost all members of distinct populations sharing the same retroviral signature (Table 2).

Retrovirus sequences with the highest identities to *Myotis myotis* endogenous retrovirus (MMER), *Myotis ricketti* endogenous retrovirus (MRER) and *Myotis daubentonii* endogenous retrovirus (MDER) were detected in bats at the Menengai Crater (Table 2). Here, all samples from *Myotis tricolor* bats were observed to harbor MMER sequences, with MRER and MDER co-infection in two samples, whereas all *M. natalensis* samples from the same location were negative for retroviruses. The sequences characterized in this study comprised 201–663 base pairs of the non-structural genes encompassing protease-reverse transcriptase coding regions (Table S3). The MMER sequences displayed a maximum intragroup nucleotide diversity of 1.8%. MMER, MRER and MDER sequence alignments are provided in Figure S4. 

In samples collected from Soysambu Conservancy, we identified sequences of another retrovirus with highest identities to *Rhinolophus ferrumequinum* retrovirus (RFRV) (Table 2). These were detected in all *Rhinolophus landeri* specimens (n = 8) as well as in *Miniopterus natalensis* (n = 1) and *Miniopterus africanus* (n = 1) bats. Segments of 350–2897 base pairs of retroviral pol and env regions were recovered, which revealed 89.8–98.8% identities within the group. Moreover, a near-complete viral genome comprising 8363 base pairs was characterized in the *Miniopterus natalensis* sample. This sequence (tentatively named as RFRV-Kenya, GenBank accession: ON893141) displayed 2.8% nucleotide divergence from the prototype RFRV (GenBank accession: JQ303125). Pairwise comparison of the deduced amino acid sequences of the gag, pol and env coding regions revealed 97%, 98% and 94.4% identities, respectively. Like the prototype virus, an additional ORF (located on nucleotides 5425–6069) encoding a partial integrase as well as a premature stop codon in pol gene were observed. However, one of the two env initiation codons identified in the prototype virus (5975–5977) was lacking. A different primer-binding site was also noted (Figure S5). 

Other distinct retroviruses were detected at Mount Suswa. In 19 of 22 bats (86.3%), virus sequences with highest identities to Galidia endogenous retrovirus (GERV) strain AMNH-110064 were observed (Table 2). The recovered segments, 151–460 bp in length, encompassed various regions of the viral genome (LTR, gag and pol) (Figure S6). Another endogenous retrovirus—detected in 17 of 22 samples (77.2%)—was highly similar to murine leukemia related virus (MLRV), initially identified in Mexican free-tailed bats (*Tadarida brasiliensis*) [36]. GERV and MLRV sequences were co-detected in 15 samples (68.1%). The MLRV sequences mostly encompassed viral protease-polymerase regions and demonstrated 86.6–92.8% nucleotide identities in comparison with the prototype strain (Figure S7). Finally, a third retrovirus pol sequence was identified in one *O. harrisoni* sample from Mount Suswa, with 76.8% identity to *Rhinolophus affinis* foamy virus 1 reported from China (Figure S8) [37]. Data on retrovirus contigs are provided in Table S3.

### 3.3. Astrovirus and Other Viruses

We detected astrovirus sequences in 14 bats (25.9%) collected at Menengai Crater and Soysambu Conservancy, but not at Mount Suswa (Table 1). Astroviruses were identified in all bat species sampled at these sites albeit with varying prevalence. The sequences comprised 235–2227 nucleotides, encompassing sections of the astrovirus ORF1a and ORF1b (Table S4, Figure S9). We observed identity ranges of 64.9–98% and 75.2–99% in the ORF1a–1b nucleotide and deduced amino acid sequences respectively, suggesting considerable genome diversity. 

We further detected virus sequences belonging to different families or unclassified viruses in five bat samples (9.2%) collected from the Menengai Crater and Soysambu Conservancy (Table 1). These sequences comprise Cyclovirus, *Rhopalosiphum padi* virus, bat betaherpesvirus B7D8, Bole tick virus 4 and Actinidia yellowing virus 2 (Table S4**)**. A partial sequence of the helicase/primase gene from bat betaherpesvirus was detected at Soysambu Conservancy, with 81.6% identity to B7D8 that was previously reported in *Miniopterus schreibersii* from Australia [38]. Virus sequences and alignments with closely related strains are provided in Figure S9. 

## 4. Discussion

We screened oral-rectal swabs obtained from individual bats from locations in Kenya’s Rift Valley, using metagenome sequencing. We targeted three bat dwelling sites with human and non-human primate hosts activity, illustrating a potential interface for pathogen spillover. The bat species documented in the study are widespread in Kenya and occur elsewhere outside the Rift Valley, in other roosting structures such as natural and coral caves, and in volcanic tubes and tunnels [15,16]. In the study, we employed a straightforward and robust cDNA-based metagenomic approach, capable of detecting a wide variety of microorganisms including viruses [14,39]. In metagenome investigations, host and environmental nucleic acids may be extremely abundant and overwhelm viral sequences. Therefore, various enrichment approaches including centrifugation/filtration, targeted virus sequence capture, random priming and nonspecific amplification have been developed, each having specific biases in the resultant datasets [40]. Nevertheless, the standard cDNA-based approach has been documented to provide sensitive and broad range detection of viruses, surpassing quantitative real-time PCR for particular targets [39]. 

We identified a variety of RNA and DNA viruses in the samples. Bat coronavirus sequences were detected with an overall prevalence of 24.1%, mainly in *O. harrisoni* bats at Mount Suswa. The sequences corresponded to the ORF1ab region of the coronavirus genome, encoding for the enzymes and accessory proteins required for virus genome replication. Phylogenetic analyses identified the sequences as a distinct alphacoronavirus lineage. High amino acid identities (>97%) further suggest these viruses to comprise a single alphacoronavirus species according to the current International Committee on Taxonomy of Viruses (ICTV) criteria, that set a demarcation threshold of 90% on conserved ORF1ab domains [41]. Previous findings from different sampling sites in Kenya have already demonstrated a considerable coronavirus diversity, with virus sequences preliminarily classified within *Alphacoronavirus* and *Betacoronavirus* genera [20,21]. Interestingly, genetically-related coronaviruses were detected in various bat species, while a given species in the same location was observed to harbor distinct viruses, as noted for *Chaerephon*, *Miniopterus* and *Rousettus* species [20,21]. We further obtained coronavirus S, NS3b, E, M, N and ORFx sequences in *O. harrisoni* samples, which indicated structural regions of the novel bat alphacoronavirus to be significantly conserved among viruses from different individuals. The presence of the ORFx further suggested tropism for bats. Our findings contribute and expand on the observations that the African continent harbors an extensive bat coronavirus diversity. Hence, potential spillover of this coronavirus from bats to other susceptible vertebrate species must be closely monitored. 

We observed retroviruses as the most frequently detected viruses at all sites. At Menengai Crater, sequences closely related to those previously reported from different *Myotis* spp. [14,42], were identified in all *M. tricolor* bats examined. At Soysambu Conservancy, sequences related to RFRV were recovered in all *R. landeri* specimens as well as in *M. natalensis* and *M. africanus* samples, albeit with a lower prevalence. RFRV was initially identified by transcriptome sequencing of the brain tissues from a greater horseshoe bat (*Rhinolophus ferrumequinum*) [42]. RFRV was observed to be closely related to non-bat endogenous retroviruses harbored by pangolin and ferret, which suggests cross-species transmission and further spread by the greater horseshoe bat [43]. The near-complete genome obtained in this study revealed the canonical retroviral genome organization and genomic markers described in the prototype strain [42]. As for the original RFRV, the RFRV-Kenya is also likely to be a defective virus. The lack of RFRV detection in all *R. ferrumequinum* samples as well as other bat species led to the assumption that RFRV may be present in a subset of the host gene pool and not yet fixed in the bat population [43]. Therefore, its replication-competent relatives may be circulating in nature [44]. Genome-wide investigations further suggested that RFRV and RFRV-like viruses likely originated from non-bat reservoirs and were probably received and dispersed by bats [43]. While detection in all *R. landeri* samples clearly suggest a vertical route in this study, the lower prevalence observed in non-*Rhinolophus* bat species may be due to ongoing or recent horizontal infections. It remains to be determined whether any other vertebrates in the region carry RFRV-Kenya. 

At Mount Suswa, we further recovered two retrovirus sequences (GERV and MLRV-like) in high prevalence and with frequent co-detection. Related to reticuloendotheliosis viruses, a group of amphotrophic bird retroviruses, GERV was described in tissues of ring-tailed mongooses (*Galidia elegans*)—native to Madagascar—with bats implicated in spread [45]. GERV detection in *O. harrisoni* samples herein is in line with pan-phylogenomic analyses indicating bats and rodents as major sources of origin and transmission of retroviruses to other mammals, significantly contributing to their spread and evolution [45]. We further identified a bat spumavirus-related sequence at this site, demonstrating sporadically reported spumaviruses to be present in this environment as well. 

Astroviruses were also prevalent in our metagenomic investigation. Viruses of the *Mamastrovirus* genus are widely distributed in many domestic animals and wildlife, as well as in humans [46]. Although data are lacking from large geographical areas, an increasing number of bat species have been discovered to host astroviruses globally, without any apparent symptoms [47]. Astroviruses in bats have also demonstrated a varying degree of host restriction and a broad sequence diversity, suggesting circulation of multiple strains [47]. We made similar observations where up to 24.8% amino acid divergence was observed. Moreover, virus sequences were present in all bat species sampled in two sites. This is the first report of astrovirus sequences in bats from Kenya, which were previously only documented as human and swine infections [48,49]. Currently, astrovirus shedding, persistence and pathogenicity in bats is not fully understood and information is insufficient to assess zoonotic potential. However, it can be speculated that emergence of a novel pathogenic strain is not unlikely, given the findings on prevalence and genetic diversity [46]. The impact of bats as astrovirus reservoirs requires further investigation.

Finally, we detected various DNA and RNA viruses in samples from Menengai Crater and Soysambu Conservancy, including a bat betaherpesvirus. Bats distributed on every continent host a variety of herpesvirus sequences [50], presumably without overt symptoms. Other viruses identified in the samples were documented in shrew intestinal contents (Cyclovirus) [51], aphids (*Rhopalosiphum padi* virus) [52], ticks (Bole tick virus 4) [53] and plants (Actinidia yellowing virus 2) [54], suggesting dietary or environmental origins. Interestingly, *Rhopalosiphum padi* virus has previously been detected in bat guano from Hungary along with bee viral pathogens [55]. Originally identified in China, Bole tick virus 4 is a flavi-like virus, having a similar genome organization but remains unassigned within *Flaviviridae* family [56]. Recent findings report new strains of the virus circulating in Kenya and infecting camels [53]. The sample with detectable Bole tick virus 4 originated from a bat with documented tick infestations, explaining the probable source of the infection. During their interaction with the environment, bats are exposed to a broad spectrum of viruses of arthropod and plant origin [57]. Similarly, viruses of bat ectoparasites can be detected in metagenomic datasets [58]. Therefore, the source of microbial sequences should be thoroughly questioned in metagenome investigations. 

## 5. Conclusions

We detected a broad spectrum of viruses and described a novel coronavirus lineage in bat samples collected from locations with spillover potential. A novel alphacoronavirus is reported in *O. harrisoni* bats. Astroviruses were initially documented in bats from Kenya. Each sampling site was characterized by a distinct retrovirus genome signature, associated with different bat species. Further monitoring of similar sites is recommended to facilitate early recognition of emerging or re-emerging viruses. 

## Figures and Tables

**Table 1:** Distribution of bat species and virus detection rates according to collection site.

		Viruses						
Site	Bat Species	*Astroviridae* (Bat Astrovirus)	*Circoviridae* (Cyclovirus)	*Coronaviridae* (Bat Alphacoronavirus)	*Dicistroviridae* (Rhopalosiphum Padi Virus)	*Herpesviridae* (Bat Herpesvirus)	*Retroviridae* ^1^	Unclassified ^2^
Menengai Crater	*Myotis tricolor* (n = 9)	1	-	-	-	-	9	-
	*Miniopterus* *natalensis* (n = 2)	1	-	-	1	-	-	1 (Actinidia yellowing virus 2)
Soysambu Conservancy	*Miniopterus* *natalensis* (n = 5)	4	1	-	-	-	1	-
	*Rhinolophus* *landeri* (n = 8)	2	-	1	-	-	8	-
	*Miniopterus africanus* (n = 8)	6	-	-	-	1	1	1 (Bole tick virus 4)
Mount Suswa	*Otomops* *harrisoni* (n = 22)	-	-	12	-	-	21	-
Total	54	14 (25.9%)	1 (1.8%)	13 (24.1%)	1 (1.8%)	1 (1.8%)	40 (74.1%)	2 (3.7%)

^1^ Distribution of Retroviruses are provided in Table 2; ^2^ Viruses; *Riboviria*; unclassified *Riboviria*; unclassified RNA.

**Table 2:** Distribution of retroviruses according to bat species and collection site.

		Retroviruses						
Site	Bat Species	Myotis Myotis Endogenous Retrovirus (MMER)	Myotis Myotis Endogenous Retrovirus (MDER)	Myotis Myotis Endogenous Retrovirus (MRER)	Rhinolophus Ferrumequinum Retrovirus (RFRV)	Galidia Endogenous Retrovirus (GERV)	Murine leukemia Related Virus (MLRV)	Rhinolophus Affinis Foamy Virus 1 (RAFV1)
Menengai Crater	*Myotis tricolor* (n = 9)	9	1	1	-	-	-	-
	*Miniopterus* *natalensis* (n = 2)	-	-	-	-	-	-	-
Soysambu Conservancy	*Miniopterus* *natalensis* (n = 5)	-	-	-	1	-	-	-
	*Rhinolophus landeri* (n = 8)	-	-	-	8	-	-	-
	*Miniopterus africanus* (n = 8)	-	-	-	1	-	-	-
Mount Suswa	*Otomops* *harrisoni* (n = 22)	-	-	-	-	19	16	1
Total	54	9 (16.6%)	1 (1.8%)	1 (1.8%)	10 (18.5%)	19 (35.1%)	16 (29.6%)	1 (1.8%)

## Data Availability

All data generated or analyzed in this study are available in the National Library of Medicine—National Center for Biotechnology Information (NCBI) Biosample and Sequence Read Archive under the BioProject PRJNA724685 (SRR14579806–SRR14579899), in GenBank with accession numbers ON28626882–ON28626931, and/or in the additional files.

## Supplementary Materials

The following supporting information can be downloaded at: https://www.mdpi.com/article/10.3390/v14122820/s1, Data S1: Coronavirus sequences obtained in the study. Table S1: Individual and sequencing Information on bat samples evaluated in the study, Table S2: Overview of the coronavirus contigs obtained in positive samples, Table S3: Overview of the retrovirus contigs obtained in positive samples, Table S4: Overview of the astrovirus and miscellaneous virus contigs obtained in positive samples, Figure S1: The Maximum Likelihood Consensus Tree Of The Coronavirus ORF1ab Sequences (2825 amino acids), Constructed Esing 1000 Replicates With Branches Achieving ≥95% Bootstrap Support Annotated By Red Dots. Figure S2: Deduced amino acid sequence alignments of the coronavirus complete ORFx and M region sequences in bats. Figure S3: Deduced amino acid sequence alignments of the partial spike protein with annotated fusion peptides and furin cleavage site in the sample SUOHC3004. Figure S4: Alignment of the *Myotis myotis* endogenous retrovirus (MMER) (A), *Myotis ricketti* endogenous retrovirus (MRER) (B) and *Myotis daubentonii* endogenous retrovirus (MDER) (C) sequences identified in bat samples. Figure S5: Alignment of the *Rhinolophus ferrumequinum* retrovirus isolate RfRV (JQ303225) with RFRV-Kenya-SYMN003 characterized in the study. Figure S6: Alignment of the Galidia endogenous retrovirus (GERV) sequences detected in bats. Figure S7: Alignment of the murine leukemia virus-related virus (MLRV) sequences detected in bats. Figure S8: Alignment of the Rhinolophus affinis foamy virus 1 sequence detected in a bat. Figure S9: Alignment of the astrovirus sequences detected in bats. Figure S10: Alignment of the Actinidia yellowing virus 2 (A), Rhopalosiphum padi virus (B), Bole tick virus 4 (C), Bat herpesvirus (D) and Cyclovirus (E) sequences identified in bat samples.
