# Supplementary material for: A Novel Coronavirus and a Broad Range of Viruses in Kenyan Cave Bats
Source: Viruses. 2022 Dec 17;14(12):2820. doi: 10.3390/v14122820 (PMC9785147; doi:10.3390/v14122820)
Supplement: Supplementary file 1 [file viruses-14-02820-s001.zip › FigureS10.pdf]

Supplementary Figure S10: Alignment of the Actinidia yellowing virus 2 (**A**), *Rhopalosiphum padi virus* (**B**), Bole tick virus 4 (**C**), Bat herpesvirus (**D**) and Cyclovirus (**E**) sequences identified in bat samples. Positions are numbered according to the reference sequence in each alignment

A: MN180071. Actinidia yellowing virus 2 isolate AYV2-Zhouzhi

```
                  2210      2220      2230      2240      2250      2260      2270      2280      2290      2300
          ....|....|....|....|....|....|....|....|....|....|....|....|....|....|....|....|....|....|....|....|
MN180071  GTCATTTTATGAACTGTACTCACGTAGTTCTGAAGGTTTCAAGATCAATAAGCTTCACAGAGTTGAGGGAGGACTCAAGCTTGCCTCGAAGGGCATGCAT
MEMN009   --------------------------------------------------------ACAGAGTTGAGGGAGGACTCAAGCTTGCCTCGAAAGGCATGCAC

                  2310      2320      2330      2340      2350      2360      2370      2380      2390      2400
          ....|....|....|....|....|....|....|....|....|....|....|....|....|....|....|....|....|....|....|....|
MN180071  CGCAAGTCTAGACCCATCGATGACCGTACTCGGTATTCGTTTTGGCTGGCATGGGGTGTTTTGCCTGACGCACAAGTCCACACGGAGGAGATGTTTTCAA
MEMN009   CGTAAGTTTCGACCAATAGATGATTACACTAGGTATTCATTTTGGTTAGCTTGGGGTGTATTACCCGACGCACAAGTCCACACAGAGGAGATGTTTTCAA

                  2410      2420      2430      2440      2450      2460      2470      2480      2490      2500
          ....|....|....|....|....|....|....|....|....|....|....|....|....|....|....|....|....|....|....|....|
MN180071  CCATGCATCTCCACTATCACGACCCTATTCTTTCTTTTGAATCGTTGTCGCTACCGTCTTATTTCTAAT-GGGTTGAATAGGGTAGGTTAACAAGGAGAG
MEMN009   CCATGCATCTCCACTATCACGACCCTGTTATTTCTCACGAGTCATTGGGATTGCCGATTTATTTCTAGTTGGGTTGAGT-GGGTAGGATCACAAGGAGAG

                  2510      2520      2530      2540      2550      2560      2570      2580      2590      2600
          ....|....|....|....|....|....|....|....|....|....|....|....|....|....|....|....|....|....|....|....|
MN180071  GTGAGCGTCTCCACCCTTTACCGAGTTTATTGGGTTAGTATGATTAATGGATGCAAAACGGTGCTGCTCAGGGTTTGTAACC-TGTGGCTTAATACTTCC
MEMN009   TTGAACGTCTCCACCCGTTACCGAGTTTATTGGGTTGGCTGAATTAATGGGAGCAAAACGGTGCTGCGCAGG-CTTGTAACC-TGTGGCTTAATACTTCC

                  2610      2620      2630      2640      2650      2660      2670      2680      2690      2700
          ....|....|....|....|....|....|....|....|....|....|....|....|....|....|....|....|....|....|....|....|
MN180071  GTACTAAGGGGTGACTTCTTAATGTCATGCATACCCGGAATGTCGAACGACTGCACGCATTCGCGCTAGAAGGTCTCGTCCCCTAGTGTACATACTGATG
MEMN009   GTACTAAGGGGTGGCTTGTTAATGCCACAT-TACCCGGAATGTCGAACGACTGCACGCTTTCGCGCCAGAAGGT-TAGTCCCCTGGTGTATCAGTCAATG

                  2710      2720      2730      2740      2750      2760      2770      2780      2790      2800
          ....|....|....|....|....|....|....|....|....|....|....|....|....|....|....|....|....|....|....|....|
MN180071  TACAGTCTCACTGCTGTTGGTGGGATCCAATACACAACAGACAACATGACTAAAACAACTCAACAGAAACGCACTAAGGTTGTCAACAACCACAGGTTCA
MEMN009   TACAGTCTCACTGCTGTTGGTGGGATCCAATACACAACAGAA----------------------------------------------------------
```

B: MN535735. Rhopalosiphum padi virus isolate RhPV/OV-157/M.dau/DK/2018

```
                  2610      2620      2630      2640      2650      2660      2670      2680      2690      2700
          ....|....|....|....|....|....|....|....|....|....|....|....|....|....|....|....|....|....|....|....|
MN535735  TTCTAATTTACAACGTTTACAGATTGAATCATTGAATTGTCCGGAAGCAGTGTCACGTCGTATAGATTTCGCTTTTAACGTTCGTATAGTACCAGAATAC
MEMN006   ----------CAACGTTTACAGATTGAATCACTGAATTGCCCCGAAGCAGTGTCGCGTCGTATAGATTTCGCTTTTAACGTTCGTATAGTACCAGAATAC

                  2710      2720      2730      2740      2750      2760      2770      2780      2790      2800
          ....|....|....|....|....|....|....|....|....|....|....|....|....|....|....|....|....|....|....|....|
MN535735  CAAATGGAATATATGAGCGCTAATGGTGATAAATTGTACAGACTAGACGCTGCCAAGGCAAGGCAAGATTTTGGAGACGTTTTATGTTTTGAAGTTTACA
MEMN006   CAAATGGAATATATGAGCGCTAATGGTGATAAATTGTACAGATTAGACGCTGCCAAGGCAAGACAAGATTTTGGAGACGTTTTATGTTTTGAAGTTTACA
```

```
                2810      2820      2830      2840      2850      2860      2870      2880      2890      2900
          ....|....|....|....|....|....|....|....|....|....|....|....|....|....|....|....|....|....|....|....|
MN535735  GATTTGATTTATTTGACGCTTCTAGCCGTCGCAACGTTTTGACTGATTTAACTTACACGGAAGTTGTGAAGTTGTGTCAGGATAAGATGCGCGATCGCGC
MEMN006   GGTTTGATTTATTTGATGCCTCTAGCCGTCGCAATATTTTGACTGATTTAACTTACACGGAAGTTGTGAAGTTGTGTCAG--------------------
```

C: MW561976. Bole tick virus 4 strain Iftin/H.dromedarii/2018

```
                5010      5020      5030      5040      5050      5060      5070      5080      5090      5100
          ....|....|....|....|....|....|....|....|....|....|....|....|....|....|....|....|....|....|....|....|
MW561976  CCGCCGCATCGCTATGGCTGGCATCTATGGGGAAGATGAACTTCAGCCTGGCGGAGATGAAGACACTCTCGCGGTCCGAGGGGCCTTGGCCGCCTTCACC
SYMA004   -----------------------------------------------CCGGCGGAGATGAAGACACTCTCGCGGTCCGAGGGGCCTTGGCCGCCTTCACC

                5110      5120      5130      5140      5150      5160      5170      5180      5190      5200
          ....|....|....|....|....|....|....|....|....|....|....|....|....|....|....|....|....|....|....|....|
MW561976  ACCAGGAAGAGCGCGCCTTACTTGGGGGCTAGTGTCGACATCGGATTCCTACGCTCCATCGCCGCAAAGATCGGCTTCGACGGTTTCTTTAAGGACGCCG
SYMA004   ACCAGGAAGAGTGCGCCTTACTTGGGGGCTAGTGTCGACATCGGATTCCTACGCTCCATCGCCGCAAAGATCGGCTTCGACGGTTTCTTTAAGGACGCCG

                5210      5220      5230      5240      5250      5260      5270      5280      5290      5300
          ....|....|....|....|....|....|....|....|....|....|....|....|....|....|....|....|....|....|....|....|
MW561976  ACATCAACTTTGCGGCCCCAGCTATCTTCCAGATGCAGACCAACACGGGTTGCACGAACACGGGTTTCTGCTTCCAACACCGCGGAGCCGTGTTCACAAA
SYMA004   ACATCAACTTTGCGGCCCCAGCTATCTTCCAGATGCAGACCAACACGGGTTGCACGAACACGGGTTTCTGCTTCCAACACCGCGGAGCCGTGTTCACAAA

                5310      5320      5330      5340      5350      5360      5370      5380      5390      5400
          ....|....|....|....|....|....|....|....|....|....|....|....|....|....|....|....|....|....|....|....|
MW561976  TTACCACGTCACAGAGGGCAGACCCCTCATCCTCCCGACCGTGTCCAACGAGGGCAAGGTGGATTACTCGCTGATCCGCTGCGTCTTCAAGAATGAGAAA
SYMA004   TTACCACGTCACAGAGGGCAGACCCCTCATCCTCCCGAC-------------------------------------------------------------
```

D: JQ805139. Bat betaherpesvirus B7D8

```
                  10        20        30        40        50        60        70        80        90       100
          ....|....|....|....|....|....|....|....|....|....|....|....|....|....|....|....|....|....|....|....|
JQ805139  GACACGATAGCGAGTTTTACATCACCGGCGGCGCGGCGAACGGTCATGACGCGTCGGCCGAAACGTGCATCAGAACCGTCCTGGGATGCTATTGGCAGAG
SYMA008   GACACGATAGCGAATTTTATATAACCGGCGGCGCCGCGAACGCTCACGACGCGTCGGCCGAAACGTGTGTCCGGACCGTCTTCGAGTGTTACTGGCGGAG

                 110       120       130       140       150       160       170       180       190       200
          ....|....|....|....|....|....|....|....|....|....|....|....|....|....|....|....|....|....|....|....|
JQ805139  GTTGTTCGGGGCGTCTGCGGGAAGCCCTGCGTGTTCTCAGTTGGACAGCGGTATGAGCGTGTTGCTGGTGTCCGATTGTCGGGTGATCAGTTCGTTCAGA
SYMA008   GTTGTTCGGGGGGCTGGACGGAAGCCCGACGTGCTCTCTGCTCGACAGCCACAGGAGCGTGTTGCTGGTGTCCGACTGTCGGACGATCAGCTCGTTCAGA

                 210       220       230       240       250       260       270       280       290       300
          ....|....|....|....|....|....|....|....|....|....|....|....|....|....|....|....|....|....|....|....|
JQ805139  TCCGTGTTTGACACGCACGGAGACGCTCACTGGACGTGCGTTATGGGCGATGCGATCTCGGACACGCTCTCTAGATGTCACGCCGACCCAGATTGGTTCG
SYMA008   TCGGCGTTTGACGCGCACAGGGAAACGCACTGGACGTGCGTCATGGGTGATGCGATGTCGGACATCCTTTCTAGATGTCACACCGACCCGGAATGGTTCG

                 310       320       330       340       350       360       370       380
          ....|....|....|....|....|....|....|....|....|....|....|....|....|....|....|....|.
JQ805139  GAGCGGAAAATATAAAAAGCGTGTTACGATCGTTGTTTTTTAAGTTTATAAGCATGAGACATAGACCGGAATTTTGGTTGG
SYMA008   GGTCCGAAAATATAAAAAACGTGCTACGATCCCTATTTTTTAAGTTTATAGCTATGAGACACCGGCCGGAGTTCTGGTTGG
```

E: AB937982. Cyclovirus ZM36a, AB937980. Cyclovirus ZM32

```
                  810       820       830       840       850       860       870       880       890       900
          ....|....|....|....|....|....|....|....|....|....|....|....|....|....|....|....|....|....|....|....|
AB937982  CCAGAAATAAAATAAATGT------TTCATTCAAAATGTATTTCTTTATTCAATCTCCTCCTTAAACTACTACAATCATACGCTAAAAATCTATACACCT
AB937980  TCGCAGGATAAATAAAAATTATATTTTTATTCAAAATGTATTTCTTTATTCAACCTTCTCCTCAAACTACTACAATCATAGGCTAAGAATCTATACACTT
SYMN011   ----------------------------------------TTTCTTTATTCAACCTTCTCCTCAAACTACTACAATCATAAGCTAAGAATCTGTACACTT

                  910       920       930       940       950       960       970       980       990      1000
          ....|....|....|....|....|....|....|....|....|....|....|....|....|....|....|....|....|....|....|....|
AB937982  CCTCTAGAGGTCTTTCGCTTGTGATCCATATATATTTAGATACGAAGTTTTCATATCCTCCTTTGACAGGGACTCTGTAAGGATATCTGTCGCAGATCTT
AB937980  CCTCTAGAGGTCTTTCGCTTGTGATCCATATATATTTAGATACGAAGTTTTCATATCCTCCTTTGACAGGGACTCGATAAGGATATCTGTCGCAGATCTT
SYMN011   CCTCTAGAGGTCGTTCACTTGTGATCCATATATATTTAGATACGAAGTTTTCATATCCTCCTTTGACAGGGACTCTGTAAGGATATCTGTCGCAGATCTT

                 1010      1020      1030      1040      1050      1060      1070      1080      1090      1100
          ....|....|....|....|....|....|....|....|....|....|....|....|....|....|....|....|....|....|....|....|
AB937982  GAGAAGCTCGTCGTATTTGATCCATCCGTAGAAGTCATCGATAACAACGCAGGGTTGTCCGGTGTATCCATCCCACCATTCTCCGCGCGGCTTGTAGTAG
AB937980  GAGAAGCTCATCGTATTTAATCCATCCGTAGAAGTCATCGATAACAACGCACGGTTGTCCGGTGTATCCATCCCACCATTCCCCGCGCGGCTTGTAGTAG
SYMN011   GAGAAGCTCATCGTACTTGATCCATCCGTAGAAGTCATCGATAACAACGCAGGGTTGTCCGGTGTATCCATCCCACCATTCCCCTCGTGGTTTGTAGTAG

                 1110      1120      1130      1140      1150      1160      1170      1180      1190      1200
          ....|....|....|....|....|....|....|....|....|....|....|....|....|....|....|....|....|....|....|....|
AB937982  GTTTCACCGAGAGCGCGGCTTTCCTCGCGCGCTTTACGGGACTTTCCAGTTCCAGTTGATCCCCAAAAGTAAAAGACTTCCGTTTTAAAGTCTCGTTCTT
AB937980  GTGTCACCAAGAGCGCGGCTTTCTTCGCGCGCTTTACGGGACTTTCCTGTTCCAGTCGCTCCCCAAAAGTAAAAGACTTCCGTTTTAAAGTCTCGTTCTG
SYMN011   GTTTCACCGAGAGCACGACTTTCTTCGCGCGCTTTACGGGACTTTCCAGTTCCAGTCGGTCCCCAAAAGTAAAAGACCTCCGTTTTAAAGTCTCGTTCTT

                 1210      1220      1230      1240      1250      1260      1270      1280      1290      1300
          ....|....|....|....|....|....|....|....|....|....|....|....|....|....|....|....|....|....|....|....|
AB937982  GATTTCCATGAGCGACTCTAATGTATTGTTCAATTCCTTTGAAATACTTGATGTACGCGACTGGGAACTGAATGGCCAAAGCTTTAATGTCTCTTTCTCC
AB937980  GATTTCCATGAGCGATTCTAATGTATTGTTCAATTCCCTTGAAATACTTGATATACGCTGTTGGGAACTGAAGGGCCAGGTTTTTAATGTTTCTCTCTCC
SYMN011   GATTTCCATGAGCGATTCTAATGTATTGTTCAATTCCTTTGTAATACTTGATATACGCGACTGGGAACTGAAGGGCCAAAGTTTTAAGGTCTCTCTCTCC

                 1310      1320      1330      1340      1350      1360      1370      1380      1390      1400
          ....|....|....|....|....|....|....|....|....|....|....|....|....|....|....|....|....|....|....|....|
AB937982  TCCTTCAATGACAGATACAACCTTTTGGAGGTCTGTTCGAGTTCCTTGAGTACTGGGTGATCCGCATTGCCAGACATCGCCACTCTTAGAGCAATATTCC
AB937980  TCCTTCAATGACAGAAACAACCTTTTGGAGGTCTGTTCTAGTTCCTTGAGTACTGGGTGATCCACATTGCCATACATCGCCACCTTTAGAGCAGTATTCC
SYMN011   TCCTTCAATGACAGAAACAACCTTTTGGAGGTCTGTTCTAGTTCCTTGAGCACTGGGCTCTCCGCATTCCCAGACATCGCCACTCTTAGAGCAATATTCC

                 1410      1420      1430      1440      1450      1460      1470      1480      1490      1500
          ....|....|....|....|....|....|....|....|....|....|....|....|....|....|....|....|....|....|....|....|
AB937982  TGATTTTCAATATCACTCCCTTTAGCTCGCTCAATGTGCGCACGGCCTCCAAATATTTGCCGTATGCTGTTGAATCGTTTGGGTCGCTGCAGATTGCTGA
AB937980  TGATTTTCGAGATCACTTCCTTTAGCTCTTTCAATGTGCGCTCGTCCGCCAAATATTTGTCGTATGCTGTTAAATCTTTTGGGTCGCTGCAAATTGCTGT
SYMN011   TTATTTTCGAGATCAGTTCCTTTAGCTCTTTCAATGTGCGCTCTACCTCCAAATATTTTCCGAATGCTGTTGAATCGTTGGGGTCGCTGCAGATTGCTGT

                 1510      1520      1530      1540      1550      1560      1570      1580      1590      1600
          ....|....|....|....|....|....|....|....|....|....|....|....|....|....|....|....|....|....|....|....|
AB937982  AAATAAAATCAATAAGTCGT-ACACTATGCACGGAGCCCGTCCCGCAGAGGGGTGGACGGGGCCCCGGCGCGGCGTAGCCGCGCCAGGGGGTGGTCCACC
AB937980  AATAGAACACCATATGTTACGACACTATGCTCGGAGCCCGTCCCGCAGAGAGGGGGGCGGGGCCCCCGCGCGCCGCAGGCGCGCGTAGGGGTGCCCC-CC
SYMN011   AATACAACACGATAAATTAATATCTAATTCTCGGAGCCCGTCCCGCAGAGAGGGGGGCGGGGCCCCCGCGCGGCGTAGCCGCGCGTAGGGGTGCCCC-CC
```

```
                1610      1620      1630      1640      1650      1660      1670      1680      1690      1700
        ....|....|....|....|....|....|....|....|....|....|....|....|....|....|....|....|....|....|....|....|
AB937982  CCGGAGCGGGATCGGGCGGCTCTATCCAACTGTGACTTTCCACTTGCA-CTAGGAGGTTATATAAAAAAA--ATTACCAAAATCCCTGGAGATGTTTTGT
AB937980  TCGGCGCGGGATCGGGCGGTTCTATCCAACTGAGACTATCCAACTGAAACAAGGAGGTTATATT----------TACCAAAATCCCTGGAGATGTTTTGT
SYMN011   TCGGCGCGGGATCGGGCGGTTCTATCCAACTGAGACTGTCCACATGCA-CTAGGAGGTTATATTAAAAAATAATTACCAAAATCCCTGGAGATGTTTTGT

                1710      1720      1730      1740      1750      1760      1770      1780      1790      1800
        ....|....|....|....|....|....|....|....|....|....|....|....|....|....|....|....|....|....|....|....|
AB937982  TCCAGTTGTAGGACAAATCTCTCGACCAAATATAGCAAATTTACATAGCTCAGCGAGGTCCTTCTGAAGCACATCGACATCGTCCTCCGTATAATTATTC
AB937980  TCCAGTACTAGGACATGTCTCTCGACCAAATATAGCAAATTTACAAAGCTCAGCGAGGTCCTTCTGAAGCACATCGACATCCTCGTCGGTATAGTTATTC
SYMN011   TCCAGTTGTAGGGCAAACCTCTCTACCAAATATAGCAAATTTACATAGCTCAGCGAGGTCCTTCTGAAGCACATCGACATCCTCGTCGGTATAATTATTC

                1810      1820      1830      1840      1850      1860      1870      1880      1890
        ....|....|....|....|....|....|....|....|....|....|....|....|....|....|....|....|....|....|....
AB937982  AGCGTCCAGCAGAATCTACGCACTGTGCTGTTGTTAGCCATAATGATGAGACCGAGCGTTACAGCGCCT-ATATATAACAACGAAGTGGCGGTA
AB937980  AGCGTCCAGCAGAATCTTCTCACGGTGCTGTTGTTAGCCATAATGATAGGTCCGAGCGTTACAACTCTTCATATATACTCACGAAGTGGCGGTA
SYMN011   AGCGTCCAGCAGAATCTTCGCAGCGTGCTGTTTTTAGCCATAATGGTGAGACCGAGCGTTACAACGTTGCTTATAAACTCCCGAAGTGGCGGTA
```
