# Supplementary material for: A Novel Coronavirus and a Broad Range of Viruses in Kenyan Cave Bats
Source: Viruses. 2022 Dec 17;14(12):2820. doi: 10.3390/v14122820 (PMC9785147; doi:10.3390/v14122820)

Supplementary Figure S2: Deduced amino acid sequence alignments of the complete ORFx and M region sequences in bats. Reference sequences (ADX59500 and ADX59498) represent Chaerephon bat coronavirusKenya/KY22/2006 strain BtKY22.

ORFx -

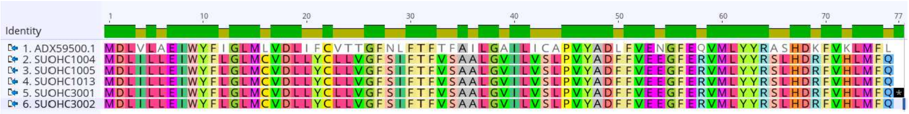

M protein -

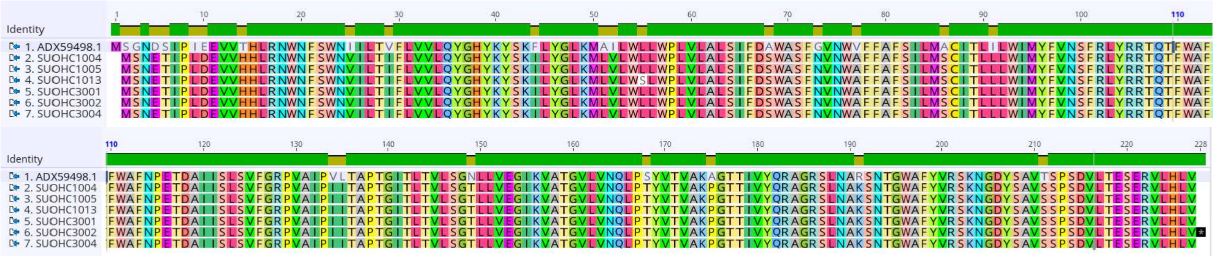

Supplementary Figure S3: Deduced amino acid sequence alignments of the partial spike protein with annotated fusion peptides and furin cleavage site in the sample SUOHC3004. The reference sequence (QGX41951) represent bat Alphacoronavirus isolate WA3607.

S protein -

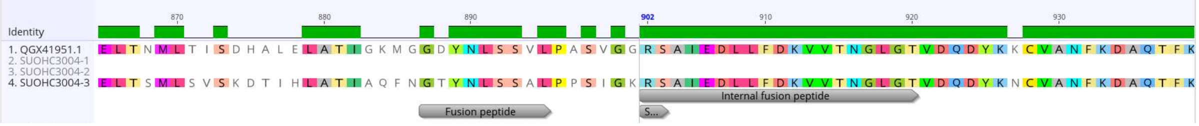

Supplement: Supplementary file 1 [file viruses-14-02820-s001.zip › FigureS2andS3.pdf]
