# Supplementary material for: A Novel Coronavirus and a Broad Range of Viruses in Kenyan Cave Bats
Source: Viruses. 2022 Dec 17;14(12):2820. doi: 10.3390/v14122820 (PMC9785147; doi:10.3390/v14122820)
Supplement: Supplementary file 1 [file viruses-14-02820-s001.zip › FigureS4.pdf]

Supplementary Figure S4: Alignment of th *Myotis myotis* endogenous retrovirus (MMER) (**A**), *Myotis ricketti* endogenous retrovirus (MRER) (**B**) and *Myotis daubentonii* endogenous retrovirus (MDER) (**C**) sequences identified in bat samples. Identical nucleotides with the reference sequence are shown as colored dots. Postions are numbered according to the reference sequence in each alignment

A: MN851302.Myotis myotis endogenous retrovirus isolate EuB-RV1

```
                  10        20        30        40        50        60        70        80        90       100
          ....|....|....|....|....|....|....|....|....|....|....|....|....|....|....|....|....|....|....|....|
MN851302  AGGGGCCGGAGGCTTTGGATCTAGTGATGCAGCATTTTGGATACAAGAAATCCACGCTACTAGACCTACAAAAATTTTACAAATTCAGGGAAAACCAATT
MEMT001   AGGGGCCGGAGGCTTTGGATCTAGTGATGCAGCATTTTGGATACAAGAAATCCATGCTACTAGACCTACAAAAATTTTACAAATTCAGGGAAAACCAATT
MEMT002   AGGGGCCGGAGGCTTTGGATCTAGTGATGCAGCATTTTGGATACAAGAAATCCATGCTACTAGACCTACAAAAATTTTACAAATTCAGGGAAAACCAATT
MEMT003   AGGGGCCGGAGGCTTTGGATCTAGTGATGCAGCATTTTGGATACAAGAAATCCATGCTACTAGACCTACAAAAATTTTACAAATTCAGGGAAAACCAATT
MEMT004   AGGGGCCGGAGGCTTTGGATCTAGTGATGCAGCATTTTGGATACAAGAAATCCATGCTACTAGACCTACAAAAATTTTACAAATTCAGGGAAAACCAATT
MEMT005   AGGGGCCGGAGGCTTTGGATCTAGTGATGCAGCATTTTGGATACAAGAAATCCATGCTACTAGACCTACAAAAATTTTACAAATTCAGGGAAAACCAATT
MEMT006   AGGGGCCGGAGGCTTTGGATCTAGTGATGCAGCATTTTGGATACAAGAAATCCATGCTACTAGACCTACAAAAATTTTACAAATTCAGGGAAAACCAATT
MEMT007   AGGGGCCGGAGGCTTTGGATCTAGTGATGCAGCATTTTGGATACAAGAAATCCATGCTACTAGACCTACAAAAATTTTACAAATTCAGGGAAAACCAATT
MEMT008   AGGGGCCGGAGGCTTTGGATCTAGTGATGCAGCATTTTGGATACAAGAAATCCATGCTACTAGACCTACAAAAATTTTACAAATTCAGGGAAAACCAATT
MEMT009   -----------------------------------------------------------------------------------------GAAAACCAATT

                 110       120       130       140       150       160       170       180       190       200
          ....|....|....|....|....|....|....|....|....|....|....|....|....|....|....|....|....|....|....|....|
MN851302  GAAGGTCTATTGGATACAGGAGCAGATGTTTCCTGCATAGCAGAGAAGGATTGGCCCTCTTCATGGCCCACGCACACCACCTCCACTTCCCTAGTAGGAC
MEMT001   GAAGGTCTATTGGATACAGGAGCAGATGTTTCCTGCATAGCAGGGAAGGATTGGCCCTCTTCATGGCCCACGCACACCACCTCCACTTCCCTAGTAGGAC
MEMT002   GAAGGTCTATTGGATACAGGAGCAGATGTTTCCTGCATAGCAGGGAAGGATTGGCCCTCTTCATGGCCCACGCACACCACCTCCACTTCCCTAGTAGGAC
MEMT003   GAAGGTCTATTGGATACAGGAGCAGATGTTTCCTGCATAGCAGGGAAGGATTGGCCCTCTTCATGGCCCACGCACACCACCTCCACTTCCCTAGTAGGAC
MEMT004   GAAGGTCTATTGGATACAGGAGCAGATGTTTCCTGCATAGCAGGGAAGGATTGGCCCTCTTCATGGCCCACGCACACCACCTCCACTTCCCTAGTAGGAC
MEMT005   GAAGGTCTATTGGATACAGGAGCAGATGTTTCCTGCATAGCAGGGAAGGATTGGCCCTCTTCATGGCCCACGCACACCACCTCCACTTCCCTAGTAGGAC
MEMT006   GAAGGTCTATTGGATACAGGAGCAGATGTTTCCTGCATAGCAGGGAAGGATTGGCCCTCTTCATGGCCCACGCACACCACCTCCACTTCCCTAGTAGGAC
MEMT007   GAAGGTCTATTGGATACAGGAGCAGATGTTTCCTGCATAGCAGGGAAGGATTGGCCCTCTTCATGGCCCACGCACACCACCTCCACTTCCCTAGTAGGAC
MEMT008   GAAGGTCTATTGGATACAGGAGCAGATGTTTCCTGCATAGCAGGGAAGGATTGGCCCTCTTCATGGCCCACGCACACCACCTCCACTTCCCTAGTAGGAC
MEMT009   GAAGGTCTATTGGATACAGGAGCAGATGTTTCCTGCATAGCAGGGAAGGATTGGCCCTCTTCATGGCCCACGCACACCACCTCCACTTCCCTAGTAGGAC

                 210       220       230       240       250       260       270       280       290       300
          ....|....|....|....|....|....|....|....|....|....|....|....|....|....|....|....|....|....|....|....|
MN851302  TAGGCAAGGCATCTAATGTAAAAAAGAGTTCTCAGATTCTAACTTGGACTGATGAAGATCAACATTCAGGAACATTCTGTCCCTATGTAATTACAACTAT
MEMT001   TAGGCAAGGCATCTAATGTAAAAAAGAGTTCTCAGATTCTAACTTGGGCTGATGAAGATCAACATTCAGGAACATTCTGTCCCTATGTAATTACGACTAT
MEMT002   TAGGCAAGGCATCTAATGTAAAAAAGAGTTCTCAGATTCTAACTTGGGCTGATGAAGATCAACATTCAGGAACATTCTGTCCCTATGTAATTACGACTAT
MEMT003   TAGGCAAGGCATCTAATGTAAAAAAGAGTTCTCAGATTCTAACTTGGGCTGATGAAGATCAACATTCAGGAACATTCTGTCCCTATGTAATTACGACTAT
MEMT004   TAGGCAAGGCATCTAATGTAAAAAAGAGTTCTCAGATTCTAACTTGGGCTGATGAAGATCAACATTCAGGAACATTCTGTCCCTATGTAATTACGACTAT
MEMT005   TAGGCAAGGCATCTAATGTAAAAAAGAGTTCTCAGATTCTAACTTGGGCTGATGAAGATCAACATTCAGGAACATTCTGTCCCTATGTAATTACGACTAT
MEMT006   TAGGCAAGGCATCTAATGTAAAAAAGAGTTCTCAGATTCTAACTTGGGCTGATGAAGATCAACATTCAGGAACATTCTGTCCCTATGTAATTACGACTAT
MEMT007   TAGGCAAGGCATCTAATGTAAAAAAGAGTTCTCAGATTCTAACTTGGGCTGATGAAGATCAACATTCAGGAACATTCTGTCCCTATGTAATTACGACTAT
MEMT008   TAGGCAAGGCATCTAATGTAAAAAAGAGTTCTCAGATTCTAACTTGGGCTGATGAAGATCAACATTCAGGAACATTCTGTCCCTATGTAATTACGACTAT
MEMT009   TAGGCAAGGCATCTAATGTAAAAAAGAGTTCTCAGATTCTAACTTGGGCTGATGAAGATCAACATTCAGGAACATTCTGTCCCTATGTAATTACCACTAC
```

```
                310       320       330       340       350       360       370       380       390       400
           ....|....|....|....|....|....|....|....|....|....|....|....|....|....|....|....|....|....|....|....|
MN851302   CCCCTTTACATTATGGGGAAGAGATATCTTATCTCAAATGGGAGTTCTATTATATAGCCCCAGTGATAGAGTAACATCACAGATGCTAGACACGAGATTT
MEMT001    CCCCTTTACATTATGGGGAAGAGATATCTTGTCTCAAATGGGAGTTGTATTATATAGCCCCAGTGATAGAGTAACATCACAGATGCTAGACACGAGATTT
MEMT002    CCCCTTTACATTATGGGGAAGAGATATCTTGTCTCAAATGGGAGTTCTATTATATAGCCCCAGTGATAGAGTAACATCACAGATGCTAGACACGAGATTT
MEMT003    CCCCTTTACATTATGGGGAAGAGATATCTTGTCTCAAATGGGAGTTGTATTATATAGCCCCAGTGATAGAGTAACATCACAGATGCTAGACACGAGATTT
MEMT004    CCCCTTTACATTATGGGGAAGAGATATCTTGTCTCAAATGGGAGTTCTATTATATAGCCCCAGTGATAGAGTAACATCACAGATGCTAGACACGAGATTT
MEMT005    CCCCTTTACATTATGGGGAAGAGATATCTTGTCTCAAATGGGAGTTGTATTATATAGCCCCAGTGATAGAGTAACATCACAGATGCTAGACACGAGATTT
MEMT006    CCCCTTTACATTATGGGGAAGAGATATCTTGTCTCAAATGGGAGTTGTATTATATAGCCCCAGTGATAGAGTAACATCACAGATGCTAGACACGAGATTT
MEMT007    CCCCTTTACATTATGGGGAAGAGATATCTTGTCTCAAATGGGAGTTGTATTATATAGCCCCAGTGATAGAGTAACATCACAGATGCTAGACACGAGATTT
MEMT008    CCCCTTTACATTATGGGGAAGAGATATCTTGTCTCAAATGGGAGTTGTATTATATAGCCCCAGTGATAGAGTAACATCACAGATGCTAGACACGAGATTT
MEMT009    CCCCTTTACATTATGGGGAAGAGATATCTTATCTCAAATGGGAGTTCTATTATATAGCCCCAGTGATAGAGTAACATCACAGATGCTAGACACGAGATTT

                410       420       430       440       450       460       470       480       490       500
           ....|....|....|....|....|....|....|....|....|....|....|....|....|....|....|....|....|....|....|....|
MN851302   AATCCAGGAAAGGGATTAGGAAAGGAAAGTAATACCTCATACTCCTCGACAGGATCTGTAATTGGGGCCATTGCCCTTAGGACTGCTGATCCCATAGTCT
MEMT001    AATCCAGGAAAGGGATTAGGAAAGGAAAGTAATACCTCACACTCCTCGACAGGATCTGTAATTGGGGCCATTGCCCTTAGGACTGCTGATCCCATAGTCT
MEMT002    AATCCAGGAAAGGGATTAGGAAAGGAAAGTAATACCTCACACTCCTCGACAGGATCTGTaattggggCCATtGCCCTTAGGACTGCTGATCCCATAGTCT
MEMT003    AATCCAGGAAAGGGATTAGGAAAGGAAAGTAATACCTCACACTCCTCGACAGGATCTGTAATTGGGGCCATTGCCCTTAGGACTGCTGATCCCATAGTCT
MEMT004    AATCCAGGAAAGGGATTAGGAAAGGAAAGTAATACCTCACACTCCTCGACAGGATCTGTAATTGGGGCCATTGCCCTTAGGACTGCTGATCCCATAGTCT
MEMT005    AATCCAGGAAAGGGATTAGGAAAGGAAAGTAATACCTCACACTCCTCGACAGGATCTGTAATTGGGGCCATTGCCCTTAGGACTGCTGATCCCATAGTCT
MEMT006    AATCCAGGAAAGGGATTAAGAAAGGAAAGTAATACCTCACACTCCTCGACAGGATCTGTAATTGGGGCCATTGCCCTTAGGACTGCTGATCCCATAGTCT
MEMT007    AATCCAGGAAAGGGATTAGGAAAGGAAAGTAATACCTCACACTCCTCGACAGGATCTGTAATTGGGGCCATTGCCCTTAGGACTGCTGATCCCATAGTCT
MEMT008    AATCCAGGAAAGGGATTAGGAAAGGAAAGTAATACCTCACACTCCTCGACAGGATCTGTAATTGGGGCCATTGCCCTTAGGACTGCTGATCCCATAGTCT
MEMT009    AATCCAGGAAAGGGATTAGGAAAGGAAAGTAATACCTCACACTCCTCGACAGAATCTGTAATTGGGGCCATTGCCCTTAGGACTGCTGATCCCATAGTCT

                510       520       530       540       550       560       570       580       590       600
           ....|....|....|....|....|....|....|....|....|....|....|....|....|....|....|....|....|....|....|....|
MN851302   GGAAATCGTCACAACCAGTATGGGTTGAGCAATGGCCCTTAACATCTGAGAAAATATCAGCAGCCCGACAATTAATTGATCAGCAATTAGCAGAGGGACA
MEMT001    GGAAATCGTCACAACCAGTATGGGTTGAGCAATGGCCCTTAACATCTGAGAAAATATCAGCAGCCCGACAATTAATTGATCAGCAATTAGCAGAGGGACA
MEMT002    GGAAATCGTCACAACCAGTATGGGTTGAGCAATGGCCCTTAACATCTGAGAAAATAGCAGCAGCCCGGCAATTAATTGATCAGCAATTAGCAGAGGGACA
MEMT003    GGAAATCGTCACAACCAGTATGGGTTGAGCAATGGCCCTTAACATCTGAGAAAATATCAGCAGCCCGACAATTAATTGATCAGCAATTAGCAGAGGGACA
MEMT004    GGAAATCGTCACAACCAGTATGGGTTGAGCAATGGCCCTTAACATCTGAGAAAATATCAGCAGCCCGACAATTAATTGATCAGCAATTAGCAGAGGGACA
MEMT005    GGAAATCGTCACAACCAGTATGGGTTGAGCAATGGCCCTTAACATCTGAGAAAATATCAGCAGCCCGACAATTAATTGATCAGCAATTAGCAGAGGGACA
MEMT006    GGAAATCGTCACAACCAGTATGGGTTGAGCAATGGCCCTTAAC---------------------------------------------------------
MEMT007    GGAAATCGTCACAACCAGTATGGGTTGAGCAATGGCCCTTAACATCTGAGAAAATATCAGCAGCCCGACAATTAATTGATCAGCAATTAGCAGAGGGACA
MEMT008    GGAAATCGTCACAACCAGTATGGGTTGAGCAATGGCCCTTAACATCTGAGAAAATATCAGCAGCCCGACAATTAATTGATCAGCAATTAGCAGAGGGACA
MEMT009    GAAAATCGTCACAACCAGTATGGGTTGAGCAATGTCCCTTAACATCTGAGAAAATATCAGCAGCCCGACAATTAATTGATCAGCAATTAGCAGAGGGACA
```

```
                 610       620       630       640       650       660
         ....|....|....|....|....|....|....|....|....|....|....|....|....
MN851302 TATAGAGCCATCAAATAGTCCCTGGAACACACCCATATTTGTAATAAGAAAGAAATCAGGCAAT
MEMT001  TATAGAGCCATCAAATAGTCCCTGGAACACACCCATATTTGTAATAAGAAAGAAATCAGGCAA
MEMT002  TATAGAGCCATCAAATAGTCCCTGGAACACACCCATATTTGTAATAAGAAAGAAATCAGGCAA-
MEMT003  TATAGAGCCATCAAATAGTCCCTGGAACACACCCATATTTGTAATAAGAAAGAAATCAGGCAA-
MEMT004  TATAGAGCCATCAAATAGTCCCTGGAACACACCCATATTTGTAATAAGAAAGAAATCAGGCAA-
MEMT005  TATAGAGCCATCAAATAGTCCCTGGAACACACCCATATTTGTAATAAGAAAGAAATCAGGCAA-
MEMT006  ----------------------------------------------------------------
MEMT007  TATAGAGCCATCAAATAGTCCCTGGAACACACCCATATTTGTAATAAGAAAGAAATCAGGCAA-
MEMT008  TATAGAGCCATCAAATAGTCCCTGGAACACACCCATATTTGTAATAAGAAAGAAATCAGGCAA-
MEMT009  TATAGAGCCATCAAATAGTCCCTGGAACACACCCATATTTGTAATAAGAAAG------------
```

B: MN851303.Myotis daubentonii endogenous retrovirus isolate EuB-RV3

```
                  10        20        30        40        50        60        70        80        90       100
         ....|....|....|....|....|....|....|....|....|....|....|....|....|....|....|....|....|....|....|....|
MN851303 GCCCTCAATTATTTTCATTTTGATGAGCATGGATTAACCCCTATGTATAAACATTGGGGAGGACTCCGGGACATAGCAACACCTCTTCCTTTGGTCCGGT
MEMT007  ------------------------------------------------AAACATTGGGGAGGTCTCAGGGACATACCAACACCTCTCCCCTTGGTCCGGT

                 110       120       130       140       150       160       170       180       190       200
         ....|....|....|....|....|....|....|....|....|....|....|....|....|....|....|....|....|....|....|....|
MN851303 GGAGAGACCCACTTACCGAAGCCTGGAAAGGCCCTGACCCACTCTTAACCCAGGGGAGAGGGTTTGCTTGTATTTTTCCAGAAGGCGACAAAAGTCCCGC
MEMT007  GGAGAGATCCACTAACCGGGGCTTGAAAGGGCCCCGACCCACTCTTAACCCAAGGGAGAGGGTTTGCTTGTGTTTTTCCAGAAGATGAGAAAAATCCCGC

                 210       220       230       240
         ....|....|....|....|....|....|....|....|....|....
MN851303 ATGGATCCCTGCTCGTGACATCAAACCCTACACCACCAGCGATGAGCCG
MEMT007  TTGGATTCCTACCCGTAACATCAAACCCTGCACCACCAGTGATGACCCG
```

C: JQ292912.Myotis ricketti retrovirus isolate MrRV

```
                 310       320       330       340       350       360       370       380       390       400
         ....|....|....|....|....|....|....|....|....|....|....|....|....|....|....|....|....|....|....|....|
JQ292912 GAGACAGAAGAAATTGCCTTTAATCAGATAAAGAAAGCTCTTTTGGCTGCTCCAGCCCTGGGCCTACCAGACATTACAAAACCCTTTCGCCTCTTTGTAG
MEMT001  -------------------------------------------------CTCCAGCCCTGGGCCTACCAGACATTACAAAACCCTTTCGCCTCTTTGTAG

                 410       420       430       440       450       460       470       480       490       500
         ....|....|....|....|....|....|....|....|....|....|....|....|....|....|....|....|....|....|....|....|
JQ292912 ACGAGCGCAAAGGAGTAGCAAAAGGAGTTTTAACCCAGGCTTTAGGCCCCTGGAGTCGCCCAGTAGCATATTTGTCTAAAAAGCTGGATCCCGTAGCTGC
MEMT001  ATGAGTGCAAATGAGTAGCAAGAGGGGTTTTAACCCAGGCTTTAGGCCTTTGCAGTCACCCAGTAGCATATTTGTCTAAAAAGCTGGATCCTGTAGCTGC
```

```
                         510       520       530       540       550       560       570       580       590       600
                ....|....|....|....|....|....|....|....|....|....|....|....|....|....|....|....|....|....|....|....|
JQ292912        CGGCTGGCCACCGTGCCTAAGAATCATTGCAGCAACTGCGCTCTTAGTCAAGGATGCCGACAAACTAACCCTGGGACAGGAGATCTGGATCACAACCCCG
MEMT001         CGGCTGGCCACCGTGCCTAAGAATCGTTGCAGCAACCGCGCTCTTAGTCAAGGATGCCAACAAACTGACCCTGGGACAGGAGATATGGATCACAACACCG

                         610       620       630       640       650       660       670       680
                ....|....|....|....|....|....|....|....|....|....|....|....|....|....|....|....|....
JQ292912        CATGCCATTGAGGGGGTCCTAAAACAGCCTCGTGATAGATGGATGAGCAATGCTCGCATGACCCACTATCAAAGCTTGCTGCTC
MEMT001         CATGCCATTGAGGGGGTCCTAAAACAGCCTCCTAATAGGTGGATGAGCAATGCTCG----------------------------
```
