# Supplementary material for: A Novel Coronavirus and a Broad Range of Viruses in Kenyan Cave Bats
Source: Viruses. 2022 Dec 17;14(12):2820. doi: 10.3390/v14122820 (PMC9785147; doi:10.3390/v14122820)
Supplement: Supplementary file 1 [file viruses-14-02820-s001.zip › FigureS5.pdf]

Supplementary Figure S5: Alignment of the Rhinolophus ferrumequinum retrovirus isolate RFRV (JQ303225) with RFRV-Kenya-SYMN003 (ON893141) characterized in the study. Retroviral gag-pol-env regions and primer binding site on the viral genome are highlighted.

```
                    10        20        30        40        50        60        70        80        90       100
            ....|....|....|....|....|....|....|....|....|....|....|....|....|....|....|....|....|....|....|....|
JQ303225    CCCTTGGCTTTAAGTGTTTGGGGTCCTTGTTAAAACCCGCTGCGTCGGGCAGAGACTCAGACCCCAGCTGGCTGGAAATAAACCTCGCTGTGTGACTTGC
SYMN003     CCCTTGGCTTTAAGTGTTTGGGGTCCTTGTTAAAACCCGCTGCGTCGGGCAGAGACTCGGACCCCAGCTGGCTGGAAATAAACCTCGCTGTGTGACTTGC

                   110       120       130       140       150       160       170       180       190       200
            ....|....|....|....|....|....|....|....|....|....|....|....|....|....|....|....|....|....|....|....|
JQ303225    ATTATTGTGCGGGTTCTCTGTCTGTCTGTGGGGGACAATTCCGGACCTTAACATTTGGGGGCTCGTCCGGGATGCCCCCCCCTCAGGGGAACAGACAACC
SYMN003     ATTATTGTGTGGGTTCTCTGTCTGTCTGAGGGGGACAATTCCGGACCTTAACAGGTGCGGCAACGTCCAGGATGCCCCCCCCTCAGGGGAACAGACAACT
                                                                 Primer binding site
                   210       220       230       240       250       260       270       280       290       300
            ....|....|....|....|....|....|....|....|....|....|....|....|....|....|....|....|....|....|....|....|
JQ303225    CCCGATACCGAGGTTTACACAGGGAACCGCGGAAGGAGGTTTGGCCACCTCCAATAGGGGAGGACTGAAACAGGTCCTCGACCGGAGAGACTGAAACAGG
SYMN003     CCCGATACCGAGGTTTACATAGGGAACCGCGGAAGGAGGTTTGGCCACCTCCAATAGGGGAGGACTGAAACAGGTCCTCGACCGGAGAGACTGAAACAGG

                   310       320       330       340       350       360       370       380       390       400
            ....|....|....|....|....|....|....|....|....|....|....|....|....|....|....|....|....|....|....|....|
JQ303225    TCTCTGCCCGGTCCCAGTTGAGAATTCTCACGGGAAGGAAAGTGCGGGTTACGACCCTGGAGGCTCCATTACTTGAAGTCGTAGGAGACGTCCCCGACGA
SYMN003     TCTCTGCCCGGTCCCAGTTGAGAATTCTCACGGGAAGGAAAGTGCGGGTTACGACCCTGGAGGCTCCGTTACTTGAAGTCGTGGGAGACGTCCCCGACGA

                   410       420       430       440       450       460       470       480       490       500
            ....|....|....|....|....|....|....|....|....|....|....|....|....|....|....|....|....|....|....|....|
JQ303225    GAAGGTGGCCCAGCGACGTCCGCAGTGGACGCCGTTTGGACAGGCCCTGGGTAAGGATCTTGAGCGTGGTGAGACTGATGTGATTGAAACGGCGCCTGTG
SYMN003     GAAGGTGGCCCAGCGACGTCCGCAGTGGACGCCATTTGGACGGGCCCTGGGTAAGGATCTTGAGCGTGTTGAGACTGGTGTGATTGAAACGGCGCCTGTG

                   510       520       530       540       550       560       570       580       590       600
            ....|....|....|....|....|....|....|....|....|....|....|....|....|....|....|....|....|....|....|....|
JQ303225    GATGTGGTGTGGTTGACCGGCCGGTGGGTGTTAAAGAGTCCCGTGCGAAGTTGTGTATTTGCTGGCACTGTGTCTTTTGTCCTTTCTTTTGCCTTTTCTT
SYMN003     GATGTGGTGTGGTTGACTGGCCGGTGGGTGTTGAAGAGTCCCGTGCGAAGTTGTGTATTTGCTGGCACTGTGTCTTTTGTCCTTTCTTTTGCCTTTTCTT

                   610       620       630       640       650       660       670       680       690       700
            ....|....|....|....|....|....|....|....|....|....|....|....|....|....|....|....|....|....|....|....|
JQ303225    GGTTTCTCTTTGTGACAATTATGGGACAGGTTCAGGTAACACCTAAGAGCCTGCTCCTGAACCACTTTCCTGAAATCCGCGCCAAGGCTCGTAATCATGG   gag
SYMN003     GGTTTCTCTTTGTGACAATTATGGGACAGGTTCAGGTAACACCTAAGACCCTGCTCCTGAACCACTTTCCTGAAATCCGCGCCAAGGCTCGTAATCATGG

                   710       720       730       740       750       760       770       780       790       800
            ....|....|....|....|....|....|....|....|....|....|....|....|....|....|....|....|....|....|....|....|
JQ303225    TGTGGAAGTGAAGAAAGGTAAGTTTGATACATTCTGCTCTGCAGAATGGCCTACTTTTAATGTGGGCTGGCCCCCCCAGGGAACTTTTTCCCTAGACATT   gag
SYMN003     TGTGGAAGTGAAGAAAGGTAAGTTTGATACATTCTGCTCTGCAGAATGGCCTACTTTTAATGTGGGCTGGCCCCCCCAGGGAACTTTTCCCCTAGACATT

                   810       820       830       840       850       860       870       880       890       900
            ....|....|....|....|....|....|....|....|....|....|....|....|....|....|....|....|....|....|....|....|
JQ303225    ATTAAGAAGGTCCGAGATATTATTAATCTGCGCCATCCGGACCAATATCCCTATATTTTGATGTGGCAAGCCTTAGTAGAGAGTCCTCCCTCCTGGCTTA   gag
SYMN003     ATTAAGAAGGTCCGAGATATTATTAATCGGCGCCATCCGGACCAATACCCCTATATTTTGATGTGGCAAGCCTTAGTAGAGAGTCCTCCCTCCTGGCTTA
```

```
                   910       920       930       940       950       960       970       980       990      1000
           ....|....|....|....|....|....|....|....|....|....|....|....|....|....|....|....|....|....|....|....|
JQ303225   AGCCCTTTATCCCCGACAAGCCAGAAGATCCCCCCCTCCCCCTTAAAGTCCTGACCGTTTCGGGACCCTCCCGCCAGCCGGCGGTGCCCACGGCCGGCCC  gag
SYMN003    AGCCCTTTATCCCCGACAAGCCAGAAGGTCCCCCCCTCCCCCTTAAAGTCCTGACCGTTTCGGGACCCTCCCGCCAGCCGGCGGTGCCCACGGCCGGCCC

                  1010      1020      1030      1040      1050      1060      1070      1080      1090      1100
           ....|....|....|....|....|....|....|....|....|....|....|....|....|....|....|....|....|....|....|....|
JQ303225   GAAACCCCCGGAGAAGCCGACCCAGGGACCCATCCTTCAGGAGGGGTCAGACATATACCCCTCCCTGATAGACCTAGATCTGGAAGAGACCCCCCCTCCT  gag
SYMN003    GAAACCCCCGGAGAAGCCGACCCAGGGACCCATCCTTCAGGAGGGGTCAGACAAATACCCCTCCCTGATAGACCTAGATCTGGAAGAGACCCCCCCTCCT

                  1110      1120      1130      1140      1150      1160      1170      1180      1190      1200
           ....|....|....|....|....|....|....|....|....|....|....|....|....|....|....|....|....|....|....|....|
JQ303225   TACGCTCCGGTGGCGCCGCTCCAGCGGTGGCGCGCGCCCAGCCCGGTGACGCCGCTCCCACCTGAGGCTGCCGCTCCTTCCGAGCTGCCTCACTCTACCG  gag
SYMN003    TACGCGCCGGTGGCGCCGCTCCAGCCACGGCGCGCGCCCAGCCCAGTGGCGCCGCTTTTGCCTGAGGCTGCCGCCCCTCCCGAGCTGCCTCACTCTACCG

                  1210      1220      1230      1240      1250      1260      1270      1280      1290      1300
           ....|....|....|....|....|....|....|....|....|....|....|....|....|....|....|....|....|....|....|....|
JQ303225   CCTCCCCAATGGCTCCCCCTCCCCCGGGTTCCCCTGCCCCAGCTCAAGGGCCGGCGAGGGGATTGAGGCCTCGTAGACGCCGGGAAGAGACCCCAGAGGA  gag
SYMN003    CCTCCCCGATGGCTCCCCCTCCCCCGGGTTCCCCTGCCCCAGCTCAAGGGCCGGCGAGGGGATTGAGGCCTCGTAGACGCCGGGAAGAGACCCCAGAGGA

                  1310      1320      1330      1340      1350      1360      1370      1380      1390      1400
           ....|....|....|....|....|....|....|....|....|....|....|....|....|....|....|....|....|....|....|....|
JQ303225   GGAACCGTCCTCCTCCACCTCCGCTGGGGCGCCGATTCTCCCCTTGCGAGCACTAGGAGGAACTGGTCCAGATGGGGAGCGAGCATACCAGTATTGGCCC  gag
SYMN003    GGAACCGTCCTCCTCCACCTCCACTGGGGCACCGATTCTCCCCGTGCGAGCACTAGGAGGGACTAGTCCAGATGGGGAGCGAGCGTACCAGTATTGGCCC

                  1410      1420      1430      1440      1450      1460      1470      1480      1490      1500
           ....|....|....|....|....|....|....|....|....|....|....|....|....|....|....|....|....|....|....|....|
JQ303225   TTTTCTAGCAGTGATCTGTACAACTGGAAGGCTCAAAACCCTCCTTTTTCTGAGGACCCGAAAGGCCTAACTGACCTGTTCGAGTCTGTCATGCACACAC  gag
SYMN003    TTTTCTAGCAGTGATCTGTACAACTGGAAGGCTCAAAACCCTCCTTTTTCTGAGGACCCGAAAGGCCTAACTGACCTGTTCGAGTCTGTCATGCACACAC

                  1510      1520      1530      1540      1550      1560      1570      1580      1590      1600
           ....|....|....|....|....|....|....|....|....|....|....|....|....|....|....|....|....|....|....|....|
JQ303225   ACAGTCCCACTTGGGATGATTGCCAGCAGCTTCTTAAGACCTTATTCACCACCGAGGAGCGCGAGCGAATCCTCACCGAGGCCAGAAAGAATGTCCCCGG  gag
SYMN003    ACAGTCCCACTTGGGATGATTGCCAGCAGCTTCTTAAGACCTTGTTCACCACCGAGGAGCGCGAGCGAATCCTCACCGAGGCCAGAAAGAATGTCCCAGG

                  1610      1620      1630      1640      1650      1660      1670      1680      1690      1700
           ....|....|....|....|....|....|....|....|....|....|....|....|....|....|....|....|....|....|....|....|
JQ303225   CGACAACGGGCGGCCGACGACCTTGCCGAACCTGATCGACGAGCGCTTTCCCCTGAATAGACCGGATTGGGACTTTGGGAACGCAGAAGGTAGGGAGCGT  gag
SYMN003    CGACAACGGGAGACCGACGACTTTGCCGAACCTTATCGATGAGCGTTTTCCCCTGAATAGACCGGATTGGGACTTTGGGAACGCAGAAGGTAGGGAGCGT

                  1710      1720      1730      1740      1750      1760      1770      1780      1790      1800
           ....|....|....|....|....|....|....|....|....|....|....|....|....|....|....|....|....|....|....|....|
JQ303225   CTCCGAGTCTACCGCCAGACTCTTATGGCAGGTCTCCGAGCGGCGGCACGCCGCCCCACCAATTTGGCCAAGGTAAAAGCTATAATGCAAGGGGATAATG  gag
SYMN003    CTCCGAGTCTACCGCCAGACTCTTATGGCAGGTCTCCGAGCGGCAGCACGCCGCCCCACCAATTTAGCCAAGGTAAAAGCTATAATGCAAGGGGAAAATG
```

```
                      1810      1820      1830      1840      1850      1860      1870      1880      1890      1900
                ....|....|....|....|....|....|....|....|....|....|....|....|....|....|....|....|....|....|....|....|
JQ303225        AAAGCCCGGCCGTGTTTTTAGAACGCCTCTATGATGCTTATAGACAGTACACCCCGTTGGACCCCCTGGCAGAGGAAAACCAGTCGGCTGTAATTATGTC  gag
SYMN003         AAAGCCCGGCCGTGTTTTTAGAACGCCTCTATGATGCTTATAGACAGTACACCCCGTTGGACCCCCTCGCAGAGGAAAACCAGTCGGCTGTAATTATGTC

                      1910      1920      1930      1940      1950      1960      1970      1980      1990      2000
                ....|....|....|....|....|....|....|....|....|....|....|....|....|....|....|....|....|....|....|....|
JQ303225        CTTTATAAACCAGGCTGCCCCAGATATTAGGAAGAAATTGTACAAACAGGAGGGACTGGGAGAAATGTCTATTCGGGATTTAATGAAAGTAGCGGAGAGA  gag
SYMN003         CTTTATAAACCAGGCTGCCCCAGATATTAGGAAGAAATTGTACAAACAGGAGGGACTGGGAGAAATGTCCATTCGGGATTTAATGAAAGTAGCGGAGAGA

                      2010      2020      2030      2040      2050      2060      2070      2080      2090      2100
                ....|....|....|....|....|....|....|....|....|....|....|....|....|....|....|....|....|....|....|....|
JQ303225        GTCTTCAACACTCGAGAGACTCCCGAAGAAAGGGAGGATAGAATTAGAAAAGAAAATCAGGAATTACAGGAACGAATCAGGAAGGAAGACAGAGAGCATC  gag
SYMN003         GTCTTCAACACTCGAGAGACTCCCGAAAAAAGGGAGGATAGAATTAGAAAAGAAAATCAGGAATTACAGGAACGAATCAGGAAGGAAGACAGAGAGCATC

                      2110      2120      2130      2140      2150      2160      2170      2180      2190      2200
                ....|....|....|....|....|....|....|....|....|....|....|....|....|....|....|....|....|....|....|....|
JQ303225        AGAGCAGGGAGAACAGGAGGCAGCAGAGGGAGATGGCCAAGATCTTGTTGGCAGGCGTGCAAAGCACAGTCAGGGTGGGACCGAGTCCGGCCGGACCAGC  gag
SYMN003         AGAGCAGGGAAAACAGGAGGCAGCAGAGGGAGATGGCTAAGATCTTGTTGGCAGGTGTGCAAAGCACAGTCAGGGTGGGRCCGAGTCCGGCAGGACCAGC

                      2210      2220      2230      2240      2250      2260      2270      2280      2290      2300
                ....|....|....|....|....|....|....|....|....|....|....|....|....|....|....|....|....|....|....|....|
JQ303225        CCGACCGTGGAGACCGCGGCCCCGACTGGATAGGGGACAGTGTGCAAACTGCAAAGAGTATGGACATTGGAAGAGGGAGTGCCCCAAGCGCCAGGGCCAA  gag
SYMN003         CCGACCGTGGAGACCGCGGCCCCGACTGGATAGGGGACAGTGTGCAAACTGCAAGGAGTATGGACATTGGAAGAGGGAGTGCCCCAAGCGCCAGGGCCAA

                      2310      2320      2330      2340      2350      2360      2370      2380      2390      2400
                ....|....|....|....|....|....|....|....|....|....|....|....|....|....|....|....|....|....|....|....|
JQ303225        ACAGGGCAAGACGCACGGGTCCTGCTGGCGGGGATGGAGAGTGACTAGGGGAGACGGGACTCGGATCCCCTCCCCGAGTCTTGGGTAACTGCGTATGTGG  pol
SYMN003         ACAGGGCAAGACGCACGGGTCCTGCTGGCGGGGATGGAGAGTGACTAGGGGAGACGGGACTCGGATCCCCTCCCCGAGTCTTGGGTAACTGCGTATGTGG

                      2410      2420      2430      2440      2450      2460      2470      2480      2490      2500
                ....|....|....|....|....|....|....|....|....|....|....|....|....|....|....|....|....|....|....|....|
JQ303225        AGGGGAAGCCAGTAGGATTCATGGTAGACACAGGAGCCCAGTACTCAGTTTTGAATAAGCCTACAGAGCCCTTATCTCAGAAAACCAGTTTGGTGCAAGG  pol
SYMN003         AGGGGAAGCCAGTAGGATTCATGGTAGACACAGGAGCCCAGTACTCAGTTTTGAATAAGCCTACAGAGCCCTTATCTCAGAAAACCAGTTTGGTACAAGG

                      2510      2520      2530      2540      2550      2560      2570      2580      2590      2600
                ....|....|....|....|....|....|....|....|....|....|....|....|....|....|....|....|....|....|....|....|
JQ303225        GGCAACTGGGTCCAAGGCTTATCGGTGGACTAGTAGGCGCCAAGTGGACTTAGGCCGCCACCAAGTGACCCACTCCTTCCTAGTTATCCCTGAATGCCCT  pol
SYMN003         GGCAACTGGGTCCAAGGCTTATCGGTGGACTAGTAAGCGCCAGGTGGACTTAGGCCGCCACCAAGTGACCCACTCCTTCCTAGTTATCCCTGAATGCCCT

                      2610      2620      2630      2640      2650      2660      2670      2680      2690      2700
                ....|....|....|....|....|....|....|....|....|....|....|....|....|....|....|....|....|....|....|....|
JQ303225        GCCCCCTTATTGGGGCGCGATCTCCTGACTAAGATCAGGGCTCAGATCCATTTTGAGCCGGATGGCATTAAGCTATTGGATGGCCAAGGACAGCCCCTCC  pol
SYMN003         GCCCCTTTATTGGGGCGCGATCTCCTGACTAAGATCAGGGCTCAGATCCATTTTGAGCCGGATGGCATTAAGGTATTGGATGGCCAAGGACAGCCCCTCC
```

```
                  2710      2720      2730      2740      2750      2760      2770      2780      2790      2800
              ....|....|....|....|....|....|....|....|....|....|....|....|....|....|....|....|....|....|....|....|
JQ303225      ACATTTTGACCCTGTCTCTTGTGGATGAACATCGCCTGTTCGCCCTGCAGGACAACCCCTACAACCCTCCCTCTACAGAATGGCCCCGTGATATGGATTA
SYMN003       ACATTTTGACCCTGTCTCTTGTGGATGAACATCGCCTGTACGCCCTGCAGGACAAACCCTACAACCCTCCCTCTACAGAATGGCCCTGTGACATGGATTA

                  2810      2820      2830      2840      2850      2860      2870      2880      2890      2900
              ....|....|....|....|....|....|....|....|....|....|....|....|....|....|....|....|....|....|....|....|
JQ303225      TTGGCTTAAAACGTACCCTCAGGCGTGGGCGGAAATAGCGGGTGTGGGCCGGGCGGCCCGCCGAGCACCAGTAGTGGTGGAACTTAAAGCCTCGGCCCAG  pol
SYMN003       TTGGCTTAAAACGTACCCTCAGGCGTGGGCGGAAATAGCGGGTGTGGGCCGGGCGGCCCGCAGAGCACCAGTAGTGGTCGAACTTAAAGCCTCGGCCCAG

                  2910      2920      2930      2940      2950      2960      2970      2980      2990      3000
              ....|....|....|....|....|....|....|....|....|....|....|....|....|....|....|....|....|....|....|....|
JQ303225      CCTATCCGGATCCGCCAGTACCCCATGTCTGCAGAGGCGCGGAAAGGGATCGCCCCGCACATTAACCGTTTACTGGAAGCTGGAATACTGAAACCTTGCC  pol
SYMN003       CCTATCCGGGTCCGCCAGTACCCCATGTCTGCAGAAGCGCGGAAAGGGATTGCCCCGCACATTAACCGTTTACTGGAAGCTGGAATACTGAAACCTTGCC

                  3010      3020      3030      3040      3050      3060      3070      3080      3090      3100
              ....|....|....|....|....|....|....|....|....|....|....|....|....|....|....|....|....|....|....|....|
JQ303225      ATTCTGCCTGGAACACCCCACTTCTCCCCGTTAAGAAACCGGGGGGAAAAGATTATAGGCCAGTCCAGGACTTGAGGGAAGTGAATAAGAGGGTTGAAGA  pol
SYMN003       ATTCTGCCTGGAACACCCCACTTCTCCCCGTTAAGAAACCGGGGGGAAAAGATTATAGGCCAGTCCAGGACTTGAGGGAAGTGAATAAGAGGGTGGAAGA

                  3110      3120      3130      3140      3150      3160      3170      3180      3190      3200
              ....|....|....|....|....|....|....|....|....|....|....|....|....|....|....|....|....|....|....|....|
JQ303225      CATCCACCCCACGGTCCCCAACCCTTATACCTTACTAAGTCACTTGCCCCCTTCACATGTCTGGTATACTACCTTAGACCTAAAGGATGCGTTTTTTAGC  pol
SYMN003       CATCCACCCCACGGTCCCCAACCCTTATACCTTACTAAGTCACTTGCCCCCTTCACATGTCTGGTATACTACCTTAGACCTAAAGGATGCTTTTTTTAGC

                  3210      3220      3230      3240      3250      3260      3270      3280      3290      3300
              ....|....|....|....|....|....|....|....|....|....|....|....|....|....|....|....|....|....|....|....|
JQ303225      ATAGCCCTGGCACCCAGCAGCCAACACATTTTTGCCTTCGAATGGAATAATGGCAATACGGGAACCCCCGGGCAGCTGACCTGGACTAGACTACCGCAAG  pol
SYMN003       ATAGCCCTGGCACCCAGCAGCCAACACATATTTGCCTTCGAATGGAATGATGGCAATACAGGAACCCCCGGGCAGCTGACCTGGACTAGACTACCGCAAG

                  3310      3320      3330      3340      3350      3360      3370      3380      3390      3400
              ....|....|....|....|....|....|....|....|....|....|....|....|....|....|....|....|....|....|....|....|
JQ303225      GCTTCAAAAACTCTCCAACTCTGTTTAATGAAGCCCTAAATCAGGATTTGGACTCGTTTCGCCAGAGCCATAATTCAGTTACGCTCCTGCAGTACGTAGA  pol
SYMN003       GCTTCAAAAACTCTCCAACTCTGTTTAATGAAGCCCTAAATCAGGATTTGGACTCGTTTCGCCAGAGCCATAATTCAGTTACGCTCCTGCAATACGTAGA

                  3410      3420      3430      3440      3450      3460      3470      3480      3490      3500
              ....|....|....|....|....|....|....|....|....|....|....|....|....|....|....|....|....|....|....|....|
JQ303225      TGACTTGCTTCTGGCGGCCCCCTCCGAAGCAGAATGCCGACAGGCCACTGGAGACCTCCTCCAGGAGCTGGGGCAGTTGGGCTATCGGGCCAGTGCAAAG  pol
SYMN003       TGACTTGCTTCTGGCGGCCCCCTCCGAAGCCGAATGCCGACAGGCCACTGGAGACCTCCTCCAGGAGCTGGGGCAGTTGGGCTATCGGGCAAGTGCAAAG

                  3510      3520      3530      3540      3550      3560      3570      3580      3590      3600
              ....|....|....|....|....|....|....|....|....|....|....|....|....|....|....|....|....|....|....|....|
JQ303225      AAGGCTCAAATATGCAGGCAAACAGTCACCTACCTGGGGTATAAACTGAAAGAAGGAACCAGATGGCTGACAGAGGCCATGAAAGAGACTATTCTTAGAC  pol
SYMN003       AAGGCTCAAATATGCAGGCAAACATTCACCTACCTGGGGTATAAGCTGAAAGAGGGAACCAGATGGCTGACTGAGGCCATGAAAGAGACTATTCTTAGAC
```

```
                   3610      3620      3630      3640      3650      3660      3670      3680      3690      3700
               ....|....|....|....|....|....|....|....|....|....|....|....|....|....|....|....|....|....|....|....|
JQ303225       TTCCAGTCCCGACCTCAGCACGAGAGGTCCGTGAGTTTTTAGGGACGACAGGCTACTGCCGGCTGTGGATTTTGGGGTATGCTGAAATAGCAAAACCTCT  pol
SYMN003        TTCCTGTCCCGACCTCAGCACGAGAGGTCCGTGAGTTTTTAGGGACGACAGGCTACTGCCGGCTGTGGATTTTGGGGTATGCTGAAATAGCAAAACCTCT

                   3710      3720      3730      3740      3750      3760      3770      3780      3790      3800
               ....|....|....|....|....|....|....|....|....|....|....|....|....|....|....|....|....|....|....|....|
JQ303225       GTATGAGGCAACCAAGGATAAGGTCCCTTGGGCCTGGGGGTCAGACCAACAGAAGGCCTACGATGAACTCAAGGTCGCTCTCCTAAGAGCCCCGGCTCTG  pol
SYMN003        GTATGAGGCAACCAAGGATAAGGTCCCTTGGGCCTGGGGGTCAGACCAACAGAAGGCCTACGATGAACTCAAGGTCGCTCTCCTAAGAGCCCCGGCTCTG

                   3810      3820      3830      3840      3850      3860      3870      3880      3890      3900
               ....|....|....|....|....|....|....|....|....|....|....|....|....|....|....|....|....|....|....|....|
JQ303225       GCATTGCCAGACCCCCTGAAGCCCTTCACTCTCTTTGTTGATGAGAGGAGGGGAATAGCGAAAGGAGTGCTAATGCAGCGTCTGGGGCCCTGGAAACGTC  pol
SYMN003        GCATTGCCAGACCCCATGAAGCCCTTCACTCTCTTTGTTGATGAGAGGAGGGGAATAGCGAAAGGGGTGCTAATGCAGCGTCTGGGGCCCTGGAAACGTC

                   3910      3920      3930      3940      3950      3960      3970      3980      3990      4000
               ....|....|....|....|....|....|....|....|....|....|....|....|....|....|....|....|....|....|....|....|
JQ303225       CGGTTGCCTATTTATCCAAGAAGCTAGATCCAGTTGCAGCAGGATGGCCCCCGTGCTTAAGGATCATTGCGGCAGTAGCCCTAATGGTGAAGGATGCTGA  pol
SYMN003        CGGTTGCCTATTTATCCAAGAAGCTAGATCCAGTTGCAGCAGGATGGCCCCCGTGCTTAAGGATCATAGCGGCAGTAGCCCTAATGGTGAAGGATGCTGA

                   4010      4020      4030      4040      4050      4060      4070      4080      4090      4100
               ....|....|....|....|....|....|....|....|....|....|....|....|....|....|....|....|....|....|....|....|
JQ303225       TAAACTCACTTTTGGGCAACATCTGAAGGTAGTGACCCCCCATGCGATCGAGGGGGTCCTGAAATATCCCCCTGGTAGGTGGATGACTAATGCCCGACTA  pol
SYMN003        TAAACTCACTTTTGGGCAACATCTGAAGGTAGTGACCCCCCATGCGATTGAGGGGGTCCTGAAATATCCCCCTGGTAGGTGGATGACTAATGCCCGACTA

                   4110      4120      4130      4140      4150      4160      4170      4180      4190      4200
               ....|....|....|....|....|....|....|....|....|....|....|....|....|....|....|....|....|....|....|....|
JQ303225       ACGCATTACCAAGGACTCTTGCTAGATGCACCCCGGATCATCTTCGCTGAACCCACCGCTCTGAATCCAGCCACCCTGCTGCCGACCCCGGATCTGAGAG  pol
SYMN003        ACCCATTACCAAGGACTCTTGCTAGATGCACCCCGGATCATCTTCGCTGAACCCACCGCTCTAAATCCAGCCACCCTGCTGCCGACCCCGGATCTGAGAG

                   4210      4220      4230      4240      4250      4260      4270      4280      4290      4300
               ....|....|....|....|....|....|....|....|....|....|....|....|....|....|....|....|....|....|....|....|
JQ303225       CTCCCCTGCATGATTGCCAAGAGATCATGGCAGAAGTCACCCAGGTGCGCCCCGACCTCCAGGACACCGCACTACCCAACAGTGAGTTGGTATGGTACAC  pol
SYMN003        CTCCCCTGCATGATTGCCAAGAGATCATGGCAGAAGTCACCCAGGTGCGCCCCGACCTCCAGGACACCGCGCTACCCAACAGTGAGTTGGTATGGTACAC

                   4310      4320      4330      4340      4350      4360      4370      4380      4390      4400
               ....|....|....|....|....|....|....|....|....|....|....|....|....|....|....|....|....|....|....|....|
JQ303225       TGATGGAAGCAGCTTCGTTATAGATGGTGTGCGGAGGGCAGGCGCAGCGGTGGTAGACCAAGGGGGAAACATCATTTGGAGTGCCTCGCTTTCCCCGGGG  pol
SYMN003        TGATGGAAGCAGCTTCGTTATAGATGGTGTGCGGAGGGCAGGCGCAGCGGTGGTAGACCAAGGGGGAAACATCATTTGGAGTGCCTCGCTTTCCCCGGGG

                   4410      4420      4430      4440      4450      4460      4470      4480      4490      4500
               ....|....|....|....|....|....|....|....|....|....|....|....|....|....|....|....|....|....|....|....|
JQ303225       ACATCAGCACAGAAGGCCGAACTGATCGCGCTGGCGGAGGCGCTGGAACGGGCCGAAGGGAGACGAGTGACTGTCTACACCGATAGCCGCTACGCCTTTG  pol
SYMN003        ACATCAGCACAGAAGGCCGAACTGATCGCGCTGGCGGAGGCGCTGGAACAGGCCAAAGGGAGACGAGTAACTGTCTACACCGATAGCCGCTATGCCTTTG
```

```
                 4510      4520      4530      4540      4550      4560      4570      4580      4590      4600
             ....|....|....|....|....|....|....|....|....|....|....|....|....|....|....|....|....|....|....|....|
JQ303225     GCACTGTCCATGTGCATGGCGCCATCTACCGGGAAAGAGGCTTTGTTACAGCGGAAGGAAAGGCTCTGCGCAATCTTCCTGAGGTACGAAGACTACTGAT   pol
SYMN003      GCACTGTCCATGTGCATGGCGCTATCTACCGGGAAAGAGTCTTTGTTACAGCGGAAGGAAAGACTCTGCGCAATCTTCCTGAGGTACGAAGACTGCTAAT

                 4610      4620      4630      4640      4650      4660      4670      4680      4690      4700
             ....|....|....|....|....|....|....|....|....|....|....|....|....|....|....|....|....|....|....|....|
JQ303225     GGCCGTGCAAATGCCCCGGGCAGTCGCAGTTGTCCACATCCCTGGGCACCAGTCTGCCCAGACCCCGGAAGCTGAAGGAAACCGGCGAGCGGATGAAGCC   pol
SYMN003      GGCTGTGCAAATGCCCCGGGCGGTCGCAGTTGTCCACATCCCTGGGCACCAGTCTGCACAGAGCCCAGAAGCTGAAGGAAACCGGCGAGCGGATGAAGCC

                 4710      4720      4730      4740      4750      4760      4770      4780      4790      4800
             ....|....|....|....|....|....|....|....|....|....|....|....|....|....|....|....|....|....|....|....|
JQ303225     GCCAAGACAGCGGCAGTAGCTTCATCAGCTTTAGCACTCACCCTGCCCACACCCGAGCTCCCTCGCCTGCCCCCGCGACCTGACTACACTCCAGAAGACC   pol
SYMN003      GCCAAGGCAGTGGCAGTAGCTTCATCAGCTTTAGCACTCACCCTGCCCACACCCGAGCTCCCTCGCCTGCCCCCGCGACCTGACTACACTCCGGAAGACC

                 4810      4820      4830      4840      4850      4860      4870      4880      4890      4900
             ....|....|....|....|....|....|....|....|....|....|....|....|....|....|....|....|....|....|....|....|
JQ303225     TGCGATGGATCCAGAACCACCACTGCCCGGAATCTGATCAGCAGGGGTGGCATCGGGATACAGAAGGAAGATTGATACTGCCGGCACAGCTAGACTGTTT   pol
SYMN003      TGCGATGGATCCAGAACCACCACTGCCCGGAATCTGATCAGCAGGGGTGGCGTCGGGATACAGAAGGAAGATTGATACTGCCGGCACAGCTAGACTGTTT

                 4910      4920      4930      4940      4950      4960      4970      4980      4990      5000
             ....|....|....|....|....|....|....|....|....|....|....|....|....|....|....|....|....|....|....|....|
JQ303225     CTTCTCTCCAACCTGCATCAAGCCACCCACTTAGGAAAAAAGAAGTTGCTGACAATTCTCGAGTCCGCCCGCCTCCGGTTTCCCCGACAAGCGGCTCAGA
SYMN003      CTTCTCTCCAACCTGCATCAAGCCACCCACTTAGGAAAAAAGAAGTTGCTGACAATTCTCGAGTCCGCCCGCCTCCGGTTTCCCCGACAAGCAGCTCAGA

                 5010      5020      5030      5040      5050      5060      5070      5080      5090      5100
             ....|....|....|....|....|....|....|....|....|....|....|....|....|....|....|....|....|....|....|....|
JQ303225     TTCAAGAGATTGTAGATCAGTGCATTGGGTGCCAGGCTATGAGACCCAGTAGGAAAGGACCCCAACATACAGGTACGAGGGTACGGGGAAGAGCGCCGGG
SYMN003      TTCAAGAGATTGTAGATCAGTGCATTGGGTGCCAGGCTATGAGACCCAGTAGGAAAGGACCCCAACATACAGGTACGAGGGTACGGGGAAGAGCGCCGGG

                 5110      5120      5130      5140      5150      5160      5170      5180      5190      5200
             ....|....|....|....|....|....|....|....|....|....|....|....|....|....|....|....|....|....|....|....|
JQ303225     ACGGAATTGGGAAGTGGATTTTACTGAGGTAAAGCCTGGGAGGTATGGGTATAAGTACTTGCTAGTAATGGTTGACACATTTTCGGGCTGGGTGGAAGCC
SYMN003      ACGGAGTTGGGAAGTGGATTTTACGGAGGTAAAGCCTGGGAGGTATGGGTGTAAGTACTTGCTAGTAATGGTTGACACATTTTCTGGCTGGGTGGAAGCC

                 5210      5220      5230      5240      5250      5260      5270      5280      5290      5300
             ....|....|....|....|....|....|....|....|....|....|....|....|....|....|....|....|....|....|....|....|
JQ303225     TTCCCCACGAAACGGGAGACTGCCCAAGTGGTTGCTAAGGCATTACTAGAAGAAATTATTCCCAGATATGGGGTTCCTGAGGTTTTAGGCTCCGATAACG
SYMN003      TTCCCCACGAAACGGGAGACTGCCCAAGTGGTTGCTAAGGCATTACTAGAAGAAATTATTCCCAGATATGGGGTTCCTGAGGTTTTAGGTTCCGATAACG

                 5310      5320      5330      5340      5350      5360      5370      5380      5390      5400
             ....|....|....|....|....|....|....|....|....|....|....|....|....|....|....|....|....|....|....|....|
JQ303225     GCCCAGCTTTCATCAGTAACGTCCTACAGGGACTAGCCCGGGCGATAGGGATCAATTGGAAGTTACATTGTGAATATAATCCCCAGAGCTCAGGGCAGGT
SYMN003      GCCCAGCTTTCATCAGTAACGTCCTACAGGGACTAGCCCGAGCGATAGGGATCAATTGGAAGTTACATTGTGAATATAATCCCCAGAGCTCAGGGCAGGT
```

```
                   5410      5420      5430      5440      5450      5460      5470      5480      5490      5500
           ....|....|....|....|....|....|....|....|....|....|....|....|....|....|....|....|....|....|....|....|
JQ303225   AGAGAGAATGAATCGGACTCTAAGAGGAGACCTTTGTCCAAACTAGCCATCGAGACTGGCGGGGACTGGGTGACCCTCTTACCCTATGCCATCTTCCGGG
SYMN003    AGAGAGAATGAATCGGACTCTAAGAGGAGACCTTTGTCCAAACTAGCCATCGAGACTGGCGGGGACTGGGTGACCCTCTTACCCTATGCCATCTTCCGGG

                   5510      5520      5530      5540      5550      5560      5570      5580      5590      5600
           ....|....|....|....|....|....|....|....|....|....|....|....|....|....|....|....|....|....|....|....|
JQ303225   TCCGGAACTCACCATATGTACATGGTTTGACACCTTTCGAAATTCTGTATGGGGCACCACCCCCCATTATTGTTCGTACTCTACCAGATCATGACCCCAA
SYMN003    TCCGGAACTCACCATATGTACATGGTATGACACCTTTCGAAATTCTGTATGGGGCACCACCCCCCATTATTGTTCGTACGCTACCAGATCATGACCCCAA

                   5610      5620      5630      5640      5650      5660      5670      5680      5690      5700
           ....|....|....|....|....|....|....|....|....|....|....|....|....|....|....|....|....|....|....|....|
JQ303225   TGTGGCCCCAAGTTATCTGGCCAGTTTAAAGGCCCTACAAGGGGTCCAACATGAGATATGGCCCCTAGTGAGTTCCCTGTATGAAATTAAGGACGCCCCG
SYMN003    TGTGGCCCCAAGTTATCTGGCCAGTTTAAAAGCCCTGCAAGGGGTCCAACATGAGATATGGCCCCTAGTGAGTTCCCTGTATGAAATTAAGGACGCCCCG

                   5710      5720      5730      5740      5750      5760      5770      5780      5790      5800
           ....|....|....|....|....|....|....|....|....|....|....|....|....|....|....|....|....|....|....|....|
JQ303225   AACCCGGAACATGGCATCGTTCCAGGGGATTGGGTATGGGTAAGGAGACACAGGTCCCGGACACTGGAGGAAAGATGGAAAGGTCCTTATGTGGTTATTC
SYMN003    AACCCGGAACATGGCATCGTTCCAGGGGATTGGGTATGGGTAAGGAGACACAGGTCCCGGACACTGGAGGAAAGATGGAAAGGTCCTTATGTGGTTATTC

                   5810      5820      5830      5840      5850      5860      5870      5880      5890      5900
           ....|....|....|....|....|....|....|....|....|....|....|....|....|....|....|....|....|....|....|....|
JQ303225   TGGTTACCCCCACTGCCTTAAAGGTTGACGGCATTGGGCCTTGGGTCCATCACTCTCACGTGCGCCGAGCCAGCCAGCTGGAGAAGACGCAAGCTAAGGA
SYMN003    TGGTTACCCCCACTGCCTTAAAGGTTGACGGCATTGGGCCTTGGGTCCATCACTCTCACGTGCGCCGAGCCAGCCAGCTGGAGAAGACGCAAGCTGAGGA

                   5910      5920      5930      5940      5950      5960      5970      5980      5990      6000
           ....|....|....|....|....|....|....|....|....|....|....|....|....|....|....|....|....|....|....|....|
JQ303225   GTGGATCGTACGGCGACACCCTGATAACCCTCTGAAGCTGCAGCTCTCCCGACCTCGGGGAAGCGTCAAGCCCCATGCCTCAGCTAACGATGGAATGGTT
SYMN003    GTGGATCGTACGGCGACACCCTGATAACCCTCTGAAACTGCAGCTCTCCCGACCTCGGGGAAGCGTCAAGCCCCCTGCCTCAGCTAACGATGGAATGGCT   env

                   6010      6020      6030      6040      6050      6060      6070      6080      6090      6100
           ....|....|....|....|....|....|....|....|....|....|....|....|....|....|....|....|....|....|....|....|
JQ303225   GCTAGCCCTAACTCTGCTCAACATCTGGGAGAAGAGCCACGCGGGGATCAACCCACACCAACCCCATAAGCTAACATGGACCCTAACAGATGGACAGACC
SYMN003    GCTAGCCCTAACTCTGCTCAACATCTGGGAGAAGAGCCACGCGGGGATCAACCCACACCAACCCCATAAGCTAACATGGACCCTAACAGATGGACAGACC   env

                   6110      6120      6130      6140      6150      6160      6170      6180      6190      6200
           ....|....|....|....|....|....|....|....|....|....|....|....|....|....|....|....|....|....|....|....|
JQ303225   CAAACAACCCTTAATAGCACCACACATACTGCCCCCATCAATACGTGGTGGCCAGATTTGTTTTTCGACCTGCGTGACATTTTCGGCACTAAACGTGGAC
SYMN003    CAAACAACCCTTAATAGCACCACACATACTGCCCCCGTCAATACGTGGTGGCCAGACTTGTTTTTCGACCTGCGTGACATTTTCGGCACTAAACGTGGAC   env

                   6210      6220      6230      6240      6250      6260      6270      6280      6290      6300
           ....|....|....|....|....|....|....|....|....|....|....|....|....|....|....|....|....|....|....|....|
JQ303225   GGCAGTATGACTACTCAGTCAGGTCCAAGCGGGCTGTAATTGACACCTCTCAAGGACATAGTGCACAAGGGTTTTGGGCCTGCCCAGGGAACCTAAGAAA
SYMN003    GGCAGTATGACTACTCAGTCAGGTCCAAGCGGGCTGTAATTGACACCTCTCAAGGACATGGTGCACAAGGGTTTTGGGCCTGCCCAGGGAACCTAAGAAA   env
```

```
                6310      6320      6330      6340      6350      6360      6370      6380      6390      6400
            ....|....|....|....|....|....|....|....|....|....|....|....|....|....|....|....|....|....|....|....|
JQ303225    CAATTGGAAAACCTGTGGCGGCCCAGACCGCTACTATTGTGGTAGTTGGAGTTGTGTCACCTCCAATGACGGGCCCCGACAGTGGGACGTTGGGAACAGG
SYMN003     CAATTGGAAAACCTGTGGCGGCCCAGACCGCTACTATTGCGGTAGTTGGAGTTGTGTCACCTCCTATGACGGGCCCCGACAGTGGGACGTTGGGAACAGG   env

                6410      6420      6430      6440      6450      6460      6470      6480      6490      6500
            ....|....|....|....|....|....|....|....|....|....|....|....|....|....|....|....|....|....|....|....|
JQ303225    GATCTAGTCAAATTCTCCTTTAGGGACCCCCTCAACCAGGTACCCCAGGTACGTGTCCAATTTAACCAAGACGCGGCACGAAAAGAGCGTGGTTGGTTAT
SYMN003     GATCTAGTTAAATTCTCCTTTAGGGACCCCCACAACCGGGTGCCCCAGGTACGTGTCCAGTTTAACCAAGACGTGGCACGAAGAGAGCGTGGTTGGTTAT   env

                6510      6520      6530      6540      6550      6560      6570      6580      6590      6600
            ....|....|....|....|....|....|....|....|....|....|....|....|....|....|....|....|....|....|....|....|
JQ303225    CAGGATTAACTTGGGGGTTCCAATTAGATATAGGCCGTTGGGCATGGATAGGCCCCCACCCCGGCGGTCTCCTAACTATTCGACTATCGGTCGAAACGAT
SYMN003     CAGGATTAACTTGGGGGTTCCAATTAGACATAGGCYGTTGGGCATGGATAGGCCCCCACCCCGGCGGTCTCCTAACTATTCGACTATCGGTCGAAACGAT   env

                6610      6620      6630      6640      6650      6660      6670      6680      6690      6700
            ....|....|....|....|....|....|....|....|....|....|....|....|....|....|....|....|....|....|....|....|
JQ303225    CAGCACTCAGGTGGGTCCAAATAAGGTACTGGCTCCTCTCGTCCCTACCAAGAACCCAGGTATATCAAGGGATAAGGACACCACAGGAAGGATCGCGGGA
SYMN003     CAGCACTCAGGTGGGTCCAAATAAGGTGCTRGCCCCTCTCGTCCCTACCAAGAACCCAGGTATATCAAGGGATAAGGACACCACAGGAGGGACTGTGGGA   env

                6710      6720      6730      6740      6750      6760      6770      6780      6790      6800
            ....|....|....|....|....|....|....|....|....|....|....|....|....|....|....|....|....|....|....|....|
JQ303225    ACCCAGCCAAAGACCTCTGTAACTCCTTCGACACCTGCTACGCAGACAACCGAAGACTCATTGCGGAAACTGGTGCGCACTGTATACGAGACCCTTAATG
SYMN003     ACCCAGCCAAAGACCTCTGTAACTCCTTCGACACCTGCTACGCAGACAACCGAAGACTCATTGCGGAAACTGGTGCGCACTRTATACGAGACCCTTAATG   env

                6810      6820      6830      6840      6850      6860      6870      6880      6890      6900
            ....|....|....|....|....|....|....|....|....|....|....|....|....|....|....|....|....|....|....|....|
JQ303225    CCACCAGTCCTCACCTCACAACCTCCTGTTGGCTGTGCTATGATGTAAAGCCCCCGTTCTATGAGGCAATAGGACTTAATGCCACTTACAACGCCTCTAA
SYMN003     CCACCAGTCCTAACCTCACAACCTCCTGTTGGCTGTGCTATGATGTAAAGCCCCCGTTCTATGAGGCAATAGGACTTAATGCCACTTACAACGCCTCTAA   env

                6910      6920      6930      6940      6950      6960      6970      6980      6990      7000
            ....|....|....|....|....|....|....|....|....|....|....|....|....|....|....|....|....|....|....|....|
JQ303225    CGGTAAGAACCCTTCTCAGTGTTCATGGGGGGATCGTAAAATTGGCTTAACCCTGCAACTAGTGAGCGGCAATGGGACCTGTCTAGGGAAGGTGCCCCAG
SYMN003     CGGTAAGAACCCTTCTCAGTGTTCATGGGGAAATCGTAAAATTGGCTTAACCATGCAACTAGTGAGCGGCAATGGGACCTGTCTAGGGAAGGTGCCCCAG   env

                7010      7020      7030      7040      7050      7060      7070      7080      7090      7100
            ....|....|....|....|....|....|....|....|....|....|....|....|....|....|....|....|....|....|....|....|
JQ303225    GCTAAACAAAGTTTATGTGCCTCCATAGACAGCTCCCCTAGTTGGAAAAGTGACACTAAGTGGTTAATCCCCAGAACTGATGGATGGTGGATATGTTCAA
SYMN003     GCTAAACAAAGTTTATGTGCCTCCATAGACAGCTCCCCTAGTTGGAAAAGTGACACTAAGTGGTTAATCCCCAGAACTGATGGATGGTGGATATGTTCAA   env

                7110      7120      7130      7140      7150      7160      7170      7180      7190      7200
            ....|....|....|....|....|....|....|....|....|....|....|....|....|....|....|....|....|....|....|....|
JQ303225    AGACTGGCCTCACCCCGTGCCTATCTACCTCGGTCTTTAATGCCGCCAATGAATTCTGTGTCCTAGTAACTGTGCTGCCCCGCATCCTCTATCACCCTGA
SYMN003     AGACTGGCCTCACCCCGTGCCTATCTACCTCGGTCTTTAATGCCGCCAATGAATTCTGTGTCCTAGTAACTGTGCTGCCCCGCATCCTCTATCACCCTGA   env
```

```
                   7210      7220      7230      7240      7250      7260      7270      7280      7290      7300
          ....|....|....|....|....|....|....|....|....|....|....|....|....|....|....|....|....|....|....|....|
JQ303225  GGAGAGTATGTATTCGCATTGGGATAGTGACACAAGTACGAGAAGTAAAAGAGAGCCCATCACCGCACTAACCATTGCCACCCTGTTCAGTTTGAGAATA
SYMN003   GGAGAGTATGTATTCGCATTGGGATAGTGACACAAGTACGAGAAGTAAAGGAGAGCCCATCACTGCACTAACCATTGCCACCCTGTTCAGTTTGGGAATA  env

                   7310      7320      7330      7340      7350      7360      7370      7380      7390      7400
          ....|....|....|....|....|....|....|....|....|....|....|....|....|....|....|....|....|....|....|....|
JQ303225  GCCGGAGCTGGGACCGGCATAGCGTCCCTGGCTACTCAACAATCAGGCATAACCTCCCTAAGGGCGGCCATAGATGAGGACATAGAAAGGTTGGAAACCT
SYMN003   GCCGGAGCCGGGACCGGCATAGCGTCCCTGGCTACTCAACAATCAGGAATGACCTCCCTAAGGGCGGCCATAGATGAGGACATAGAAAGGTTGGAAACCT  env

                   7410      7420      7430      7440      7450      7460      7470      7480      7490      7500
          ....|....|....|....|....|....|....|....|....|....|....|....|....|....|....|....|....|....|....|....|
JQ303225  CGATTAGTCATTTAGAGAAGTCGCTCACCTCTCTATCCGAGGTAGTACTCCAGAATAGAAGAGGACTTGATTTGTTGTTCCTCCAACAAGGGGGACTGTG
SYMN003   CGATTAGTCATTTAGAGAAGTCGCTCACCTCTCTATCCGAGGTAGTACTCCAGAATAGAAGAGGACTTGATTTGTTGTTCCTCCAACAAGGGGGACTATG  env

                   7510      7520      7530      7540      7550      7560      7570      7580      7590      7600
          ....|....|....|....|....|....|....|....|....|....|....|....|....|....|....|....|....|....|....|....|
JQ303225  TGTCGCGCTAGGGGAGGAATGTTGTTTTTATGCTGACCATACTGGTGTAGTAAAAGAATCCATGGCAAAGGTGAGAGAAGGGTTAGCTAAGAGAAAACGG
SYMN003   TGCCGCGCTGGGGGAGGAATGTTGTTTTTATGCTGACCATACTGGTSTGGTAAGAGAACGCATAGGMAAGATGMCCGCTGCCTCAGCTAAGAGAAAACAG  env

                   7610      7620      7630      7640      7650      7660      7670      7680      7690      7700
          ....|....|....|....|....|....|....|....|....|....|....|....|....|....|....|....|....|....|....|....|
JQ303225  GAAAGGGAAGCTCAGGAGAACTGGTTTGAGGCTTGGTTTAATAGATCCCCCTGGCTCACCACCTTAGTATCTACCTTAGTGGGCCCGGTTATCTTGCTGG
SYMN003   GAAAGGGAAGCTCAGGAGAACTGGTTTGAGGCTTGGTTTAACAGATCCCCCTGGCTCACCACCTTAGTATCTACCTTAGTGGGCCCAATTATCTTGCTTG  env

                   7710      7720      7730      7740      7750      7760      7770      7780      7790      7800
          ....|....|....|....|....|....|....|....|....|....|....|....|....|....|....|....|....|....|....|....|
JQ303225  TGCTTATTTTAACCTTTGGCCCTTGCATATTGAACAAGCTTATTAATTTTGTGAAAGATCGTGTTAATACTGTCCAGCTCATGGTCCTAAGACAACAGTA
SYMN003   TGCTTATTCTAACCTTAGGCCCTTGCATATTAAACAAGCTTATTAATTTTGTAAAAGATCGTGTTAATACCGTCCAGCTCATGGTCCTAAGACAACAGTA  env

                   7810      7820      7830      7840      7850      7860      7870      7880      7890      7900
          ....|....|....|....|....|....|....|....|....|....|....|....|....|....|....|....|....|....|....|....|
JQ303225  TGAGACAGTGCCCACCCGCGAGGACCTCTACGGCTGGCCCGTACATGAGCAAGATTCCTCATTATGAACAACAAACGGCGGGGAAATGTAAGAGACCTTT
SYMN003   TGAGACAGTGCCCACCCGTGAGGACTTCTACAGCTGGCCCGTACATGAGCAAGATTCCTCATTATGAACAACACACAGGGGGGAAATGTAAGAGACTGTT

                   7910      7920      7930      7940      7950      7960      7970      7980      7990      8000
          ....|....|....|....|....|....|....|....|....|....|....|....|....|....|....|....|....|....|....|....|
JQ303225  AGCATGAGAACTGCTATCTTAAGGTCAGGGTTAACAGCCCCTAGCCTGAAACAACCTAATTGTAAATTGTAAGAGACCGTTAGCATGAGAACTGCTATCT
SYMN003   AGCATGAGAACTAC-ATCTTTAGGTCAGGGTTAACAGCCCCTAGCCTGAAACAGCATAGTGGGAAA-TGTAAGAGACTGTTAGCATGAGAACTAC-ATCT

                   8010      8020      8030      8040      8050      8060      8070      8080      8090      8100
          ....|....|....|....|....|....|....|....|....|....|....|....|....|....|....|....|....|....|....|....|
JQ303225  TAAGGTCAGGGTTAACAGCCCTTAGCTTGAAACAACCTAATTGTAAA-TTCCAGGATGTGGGCAGTTGCAGCACGGAGACCAGAAGTCCTGTAGCTTATC
SYMN003   TTAGGTCAGGGTTAACAGCCCCTAGCCTGAAACAACATAATTATAAAATTCCAGGATGTGGGCAGTTACAGCATGGAGACGAGAAGTCCTGCAGCTTATC
```

```
                 8110      8120      8130      8140      8150      8160      8170      8180      8190      8200
          ....|....|....|....|....|....|....|....|....|....|....|....|....|....|....|....|....|....|....|....|
JQ303225  TTATA--------CTCCTTGTCCTGTAGTTAATCTTATACTCTTTGAAACTATGTAAGTCCTGTAGTTTATCTTATACTCCTTGAAGCTATGTAAGTCCT
SYMN003   TTATACTCCTGGTCTCCTTGTCCTGTAGTAAATCTTATACTCTTTGAAGCTATGCAAGTCCTGTAGTTTATCTTATACTCTTTGAAGCTATGCAAGTCCT

                 8210      8220      8230      8240      8250      8260      8270      8280      8290      8300
          ....|....|....|....|....|....|....|....|....|....|....|....|....|....|....|....|....|....|....|....|
JQ303225  GTAGTTTATCTTGTACTCTCTGAAGCTATGGAAAATTACTGAGTGCCTTGAAAATGCTATATAAACCCTTGGCTTTAAGTGTTTGGGGTCCTTGTTAAAA
SYMN003   GTAGTTTATCTAGTACTCTCTGAAGCTATGGAAAATTACTGAGTGCCTTGAAAATGCTATATAAACCCTTGGCTTTAAGTGTTTGGGGTCCTTGTTAAAA

                 8310      8320      8330      8340      8350      8360      8370      8380      8390
          ....|....|....|....|....|....|....|....|....|....|....|....|....|....|....|....|....|....|....|...
JQ303225  CCCGCTGCGTCGGGCAGAGACTCGGACCCCAGCTGGCTGGAAATAAACCTCGTTGTGTGACTTGCAAAAAATAAAAAAAAAAAAAAAAAAAAAAAAAA
SYMN003   CCCGCTGCGTCGGGCAGAGACTCGGACCCCAGCTGGCTGGAAATAAACCTCGCTGTGTGACTTGCA--------------------------------
```
