# Supplementary material for: A Novel Coronavirus and a Broad Range of Viruses in Kenyan Cave Bats
Source: Viruses. 2022 Dec 17;14(12):2820. doi: 10.3390/v14122820 (PMC9785147; doi:10.3390/v14122820)
Supplement: Supplementary file 1 [file viruses-14-02820-s001.zip › FigureS6.pdf]

Supplementary Figure S6: Alignment of the Galidia endogenous retrovirus (GERV) sequences detected in bats. Positions are indicated according to the GERV strain AMNH-110064 (GenBank accession: KF313135).

```
                   410       420       430       440       450       460       470       480       490       500
            ....|....|....|....|....|....|....|....|....|....|....|....|....|....|....|....|....|....|....|....|
KF313135    CCCCTTAGAAGGGAATAAAAGAGGAGGATGGAGAAGGCTAAGTGCTGCTCGCACCCGCTACCCGCTTTTGCTTAGCAGGGCGTCAGCCCAGATTCGAATC
SUOHC1004   -------------------------------------------------------------------------------------GCCCGGGTTCGAACC
SUOHC1011   -------------------------------------------------------------------------------------GCCCGGGTTCGAACC
SUOHC2004   -------------------------------------------------------------------------------------GCCCGGGTTCGAACC

                   510       520       530       540       550       560       570       580       590       600
            ....|....|....|....|....|....|....|....|....|....|....|....|....|....|....|....|....|....|....|....|
KF313135    TGTAATAAATTTTCAAAAGGAA-ACCCAAATTCTCGGGGGTCGTCTGTTCTTGCGGGGTCTTCTCTTCATTACATTTGGAGGCCCCAGCGAGATC-CCCG
SUOHC1004   TGTAATAAATCAAATTCTGCTC-AACGAAATTCTCTGGAGTTGTCTGT-CTT-CGCGGGAGACTCCTTATTACATTTGGAGGCCCCAGCGAGATCTCCCG
SUOHC1011   TGTAATAAAACCAAATTCTGCTCAACGAAATTCTCTGGAGTTGTCTGT-CTT-CGCGGGAGACTCCTTATTACATTTGGAGGCCCCAGCGAGATCTCCCG
SUOHC2004   TGTAATAAAACCAAATTCTGCTCAACGAAATTCTCTGGAGTTGTCTGT-CTT-CGCGGGAGACTCCTTATTACATTTGGAGGCCCCAGCGAGATCTCCCG
SUOHC3005   ---------------------------AAATTCTCTGGAGTTGTCTGT-CTT-CGCGGGAGTCTCCTTATTACATTTGGAGGCCCCAGCGAGATCTCCCG
SUOHC2002   ---------------------------AAATTCTCTGGAGTTGTCTGT-CTT-CGCGGGAGTCTCCTTATTACATTTGGAGGCCCCAGCGAGATCTCCCG
SUOHC2001   ---------------------------AAATTCTCTGGAGTTGTCTGT-CTT-CGCGGGAGACTCCTTATTACATTTGGAGGCCCCAGCGAGATCTCCCG
SUOHC3004   ---------------------------AAATTCTCTGGAGTTGTCTGT-CTT-CGCGGGAGTCTCCTTATTACATTTGGAGGCCCCAGCGAGATCTCCCG

                   610       620       630       640       650       660       670       680       690       700
            ....|....|....|....|....|....|....|....|....|....|....|....|....|....|....|....|....|....|....|....|
KF313135    CCAGGGGGG--------TTCGAGGCCCCCCGGGACAGACACCCATCCGCGGATCCTGGGAGCTCCCGTTCGGTATCCTGCACGGCGCCGGTAAGTCAGGC
SUOHC1004   CAAGGGGAAGGCTTCTTTTCGAGCCCCCCA---ACAGACAAC-AGCCG--GATCCGGGACGGTTTGGT-CTGTTTCCTGCGCGGCGCCGGTAAGTCAGG-
SUOHC1011   CAAGGGGAAGGCTTCTTTTCGAGCCCCCCC---ACAGACAAC-AGCCG--GATCCGGGACGGTTTGGT-CTGTTTGCTGCGCGGCGCCGGTAAGTCAGGA
SUOHC2004   CAAGGGGAAGGCTTCTTTTCGAGCCCCCCA---ACAGACAAC-AGCCG--GATCCGGGACGGTTTGGT-CTGTTTCCTGCGCGGCGCCGGTAAGTCAGGA
SUOHC3005   CAAGGGGAAGGCTTCTTTTCGAGCCCCCCCA--ACAGACAAC-AGCCG-GGATCCGGGACGGTTTGGT-CTGTTTCCTGCGCGGCGCCGGTAAGTCAGGA
SUOHC2002   CAAGGGGAAGGCTTCTTTTCGAGCCCCCCA---ACAGACAACAA-CC-CGGATCCGGGACGGTTTGGT-CTGTTTCCTGCGCGGCGCCGGTAAGTCAGGA
SUOHC2001   CAAGGGGAAGGCTTCTTTTCGAGCCCCCCA---ACAGACAAC-AGCCG--GATCCGGGACGGTTTGGT-CTGTTTCCTGCGCGGCGCCGGTAAGTCAGGA
SUOHC3004   CAAGGGGAAGGCTTCTTTTCGAGCCCCCCA---ACAGACAAC-AGCCG--GATCCGGGACGGTTTGGT-CTGTTTCCTGCGCGGCGCCGGTAAGTCAGGA

                   710       720       730       740       750       760       770       780       790       800
            ....|....|....|....|....|....|....|....|....|....|....|....|....|....|....|....|....|....|....|....|
KF313135    CTTTTGTTTCTGGTTG---CTCGCGAGCTCTGAAGGA---GTCTGTGGACGGCGGGACGCCGCCATCGGGT-CCCGGACTCGGCCCAGAGGGGTGACGAC
SUOHC1011   TTTCTGTT-CTGTCTGATACTCGCGAGTTTTCTCTGGTTGGTCGAGGAACGGCGGGACGCCGCCTTCGGGT-TCCCGA----------------------
SUOHC2004   TTTCTGGT-CTGTCTGATACTCGCGAGTTTTCTCTGG---GTCGAGGAACGGCGGGACGCCGCCTTCGGGTTCCCGGAC---------------------
SUOHC3005   TTTCTGGT-CTGTCTGATACTCGCGAG-------------------------------------------------------------------------
SUOHC2002   TTTCTGGT-CTGTTTGATACTCGCGAG-------------------------------------------------------------------------
SUOHC2001   TTTCTGTT-CTGTCTGATACTCGCGAG-------------------------------------------------------------------------
SUOHC3004   TTTCTGTT-CTGTCTGATACTCGCGAGTTTTCTCTGGTTGGTCGAGGAACAGCGGGACGCCGCCTTCGGGT-TCCCGACC--------------------
```

```
                  1010      1020      1030      1040      1050      1060      1070      1080      1090      1100
            ....|....|....|....|....|....|....|....|....|....|....|....|....|....|....|....|....|....|....|....|
KF313135    GATTTCTGCGCCTGCCGCGCCGCGCTGCGATACTTAGAACCAAAAACAGGCCTTCTAGCACTAACTAGTAAGGCCGGCTGCCCCCTCCCTCTTGGCCAAG
SUOHC3003   -------------------------------------------------------------------------------GTCCTCTCCCTCTCGGCCAAG
SUOHC1003   ---------------------------------------------------------------------------------CCTCTCCCTCTCGGCCAAG
SUOHC1006   ---------------------------------------------------------------------------------CCTCTCCCTCTCGGCCAAG
SUOHC1014   ---------------------------------------------------------------------------------CCTCTCCCTCTCGGCCAAG
SUOHC1001   ---------------------------------------------------------------------------------CCTCTCCCTCTCGGCCAAG
SUOHC1002   ---------------------------------------------------------------------------------CCTCTCCCTCTCGGCCAAG
SUOHC3007   ------------------------------------------------------------------------------------CTCCCTCTCGGCCAGG
SUOHC3002   ------------------------------------------------------------------------------------------------CAAG

                  1110      1120      1130      1140      1150      1160      1170      1180      1190      1200
            ....|....|....|....|....|....|....|....|....|....|....|....|....|....|....|....|....|....|....|....|
KF313135    ATGGGACAAGGCAGCTCTAAGGGCCCATTGAGCCCATTGGAATGTCTGATTAAAAACTTCTCCGATTTTCGGAATCGAGCCGCGGGATACGGGGCAACAG
SUOHC3003   ATGGGACAGGGCGACTCAAAGGGCCCCTTGAGCCCCTTAGAATGCCTAATTAAGAATTTTGATGACTTCCGCCGCCGCGCGGACGGGTACGGGGCGCCCG
SUOHC1003   ATGGGACAGGGCGACTCAAAGGGCCCCTTGAGCCCCTTAGAATGCCTAATTAAGAATTTTGATGACTTCCGCCGCCGTGCGGACGGGTACGGGGCCCCCG
SUOHC1006   ATGGGACAGGGCGACTCAAAGGGCCCCTTGAGCCCCTTAGAATGCCTAATTAAGAATTTTGATGACTTCCGCCGCCGCGCGGACGGGTACGGGGCGCCCG
SUOHC1014   ATGGGACAGGGCGACTCAAAGGGCCCCTTGAGCCCCTTAGAATGCCTAATTAAGAATTTTGATGACTTCCGCCGCCGCGCGGACGGGTACGGGGCGCCCG
SUOHC1001   ATGGGACAGGGCGACTCAAAGGGCCCCTTGAGCCCCTTAGAATGCCTAATTAAGAATTTTGATGACTTCCGCCGCCGCGCGGATGGGTACGGGGCGCCCG
SUOHC1002   ATGGGACAGGGCGACTCAAAGGGCCCCTTGAGCCCCTTAGAATGCCTAATTAAGAATTTTGATGACTTCCGCCGCCGCGCGGACGGGTACGGGGCGCCCG
SUOHC3007   ATGGGATAGGGCGACTCAAAGGGCCCCTTGAGCCCCTTAGAATGCCTAATTAAGAATTTTGATGACTTCCGCCGCCGCGCGGATGGGTACGGGGCGCCCG
SUOHC3002   ATGGGACAGGGCGACTCAAAGGGCCCCTTGAGCCCCTTAGAATGCCTAATTAAGAATTTTGATGACTTCCGCTGCCGCGCGGACGGGTACGGGGCGCCCG

                  1210      1220      1230      1240      1250      1260      1270      1280      1290      1300
            ....|....|....|....|....|....|....|....|....|....|....|....|....|....|....|....|....|....|....|....|
KF313135    TGAATGCNTTCGACCTGCGAAAATTCTGTGAGTTGGAATGGCCCACCTTTGGGGTCGGATGGCCAGGTGTGGGGACCCTGGATGTAGGCACGG-CCAGTG
SUOHC3003   TCAATGCTTTTGATTTGAGAAAATTCTGTGAGAGGGAGTGGCCCACGTTCGGGGTCGGCTGGCCAGACATAGGGACCTTTGATCTGGCGACGGTCCAG-G
SUOHC1003   TCAATGCTTTCGATTTGAGAAAATTCTGTGAG--------------------------------------------------------------------
SUOHC1006   TCAATGCTTTCGATTTGAGAAAATTCTGTGAGAGGGAGTGGCCCACGTTCGGGGTCGGCTGGCCAGACATAGGGACCTTTGATCTGGCGACGG-TCCAGG
SUOHC1014   TCAATGCTTTTGATTTGAGAAAATTCTGTGAGAGGGAGTGGCCCACGTTCGGGGTCGGCTGGCCAGACATAGGGACCTTTGATCTGGCGACGG-TCCAGG
SUOHC1001   TCAATGCTTTTGATTTGAGAAAATTCTGTGAGAGGGAGTGGCCCACGTTCGGGGTCGGTTGGCCAGACATAGGGACCTTTGATCTGGCGACGG-TCCAGG
SUOHC1002   TCAATGCTTTTGATTTGAGAAAATTCTGTGAGAGGGAGTGGCCCACGTTCGGGGTCGGCTGGCCAGACATAGGGACCTTTGATCTGGTGACGG-TCCAGG
SUOHC3007   TCAATGCTTTTGATTTGAGAAAATTCTGTGAGAGGGAGTGGCCCACGTTCGGGGTCGGTTGGCCAGACATAGGGACCTTTGATCTGGCGACGGTCCAG-G
SUOHC3002   TCAATGCTTTTGATTTGAGAAAATTCTGTGAGAGGGAGTGGCCCACGTTCGGGGTCGGYTGGCCAGACATAGGGACCTTTGATCTGGCGACGGTCCAG-G

                  1310      1320      1330      1340      1350      1360      1370      1380      1390      1400
            ....|....|....|....|....|....|....|....|....|....|....|....|....|....|....|....|....|....|....|....|
KF313135    CGGTCCGTGCTGTT-TGCTACAGCACCCCTGGCCATCCTGACCAGGTCCCTTACATAGACATCTGGATTGACATCATCTGGGACAGGCCCGGTTATCTGA
SUOHC3003   CGGTCAG-GCGGACATGTTATGGAAATCCTGGGCACCCAGACCAAATCCCGTA-----------------------------------------------
SUOHC1006   CGGTC-----------------------------------------------------------------------------------------------
SUOHC1014   CGGTC-----------------------------------------------------------------------------------------------
SUOHC1001   CGGTC-----------------------------------------------------------------------------------------------
SUOHC1002   CGGTC-----------------------------------------------------------------------------------------------
SUOHC3007   CGGTCAG-GCGGACATGTTATGGAAATCCTGGGCACCCAGACC---------------------------------------------------------
SUOHC3002   CGGTCAG-GCGGACATGTTATGGAAATCCTGGGCACCCAGACCAAAT-----------------------------------------------------
```

```
                   2510      2520      2530      2540      2550      2560      2570      2580      2590      2600
               ....|....|....|....|....|....|....|....|....|....|....|....|....|....|....|....|....|....|....|....|
KF313135       ACCGCTCTAGGGAAGAACCAATGTGCCTACTGCAAGGAGGAAGGACATTGGAAGAGAGAATATTCCAAACGCCCTCGGGAGGGAGAGGGCCACCCCACCC
SUOHC1005      ------------------------GCTTATTGCAAGGAAGAAGGACACTGGAAGAGAGAGTGCCCAAAAAGACCCA---AGGCTGAGGCTCCGCCAGCCC
SUOHC1007      -------------------------------GCAAGGAAGAAGGACACTGGAAGAGAGAGTGCCCAAAAAGACCCA---AGGCTGAGGCTCCGCCAGCCC

                   2610      2620      2630      2640      2650      2660      2670      2680      2690      2700
               ....|....|....|....|....|....|....|....|....|....|....|....|....|....|....|....|....|....|....|....|
KF313135       CGGTCCTGGTCAAAGAGACGGACTAGAGAACTCGGGGCTCCCTC-GCCCTCCAGGAACCCAGGCTAAAACTCTCGGTAGGAGGGCAACTGATTGATTTCT
SUOHC1005      CAGTATTGGTAGAAGAGGAAGACTAGGGCCGCCGTGGGGCC-TCAGTCCTCCGGGAACCCAGGCTAAAACTCTCGGTAGGGGGACAAATGATTGACTTTT
SUOHC1007      CAGTATTGGTAGAAGAGGAAGACTAGGGCCGCCGTGGGGCC-TCAGTCCTCCGGGAACCCAGGCTAAAACTCTCGGTAGGGGGACAAATGATTGACTTTT

                   2710      2720      2730      2740      2750      2760      2770      2780      2790      2800
               ....|....|....|....|....|....|....|....|....|....|....|....|....|....|....|....|....|....|....|....|
KF313135       TGGTGGATACCGGGGCCACCCATTCTGTGGTACAAGCGCCAGTTGGGCCC--CTCACCCCTGAGCGGGTCAGAATTATAGGGGCCACTGGGATATCCAAA
SUOHC1005      TAGTGGACACCGGGGCCACTCATTCGGTAGTTCAAGCCCCTGTTGGGCCCTACTCACGCCAAAA--GGTGAGAATCGTAGGAGCCACTGGAACTCCTCAG
SUOHC1007      TAGTGGACACCGGGGCCACTCATTCGGTAGTTCAAGCCCCTGTTGGGCCCTACTCACGCCAAAA--GGTGAGAATCGTAGGAGCCACTGGAACTCCTCGG

                   2810      2820      2830      2840      2850      2860      2870      2880      2890      2900
               ....|....|....|....|....|....|....|....|....|....|....|....|....|....|....|....|....|....|....|....|
KF313135       AGCTACCCCAAATCGGAGGGTCGAATCACGGACCTGGGTAAAGGACTCGTAACCCACTCCTTCCTTGTGATACCCGAATGTCCTGACCCACTGCTGGGAA
SUOHC1005      ACCTACCCCACCTCTGAGGGCCGGATCACCAATCTGAGCCAGGGTACTGTCACCCACTCCTTCCTCGTTATACCGGAATGCCCCGACCCTCTCCTGGGTA
SUOHC1007      ACCTACCCCACCTCTGAGGGCCGGATCACCAATCTGAGCCAGGGTACTGTCACCCACTCCTTCCTTGTTATACCGGAATGCCCCGACCCTCTCCTGGGTA

                   2910      2920      2930      2940      2950      2960      2970      2980      2990      3000
               ....|....|....|....|....|....|....|....|....|....|....|....|....|....|....|....|....|....|....|....|
KF313135       GGGACCTCCTCCACAAGCTCAGGGCCACCATCACCTTCGATGGGGTAGGGACTCCAGAGGTAAAAACTGAAGGGAAAGTCCTCGTGACCTTGCCCCTGGC
SUOHC1005      GAGATTTGCTACACAAGTTACAGGCCACCAT---------------------------------------------------------------------
SUOHC1007      GAGATTTGCTACACAAGTTGCAGGCCACCAT---------------------------------------------------------------------


                   3210      3220      3230      3240      3250      3260      3270      3280      3290      3300
               ....|....|....|....|....|....|....|....|....|....|....|....|....|....|....|....|....|....|....|....|
KF313135       GGGGAGTACGCGAGAATATCCGAAAACTAAGGGCGGCCGGCATCCTAGTTCCGTGCCACTCGCCCTGGAACACCCCCCTACTCCCAGTTCGAAAGGCCGA
SUOHC3006      -----------------------------------GGGGGTATCCTAGTGCCTTGTCAGTCGGCATGGAACACCCCACTCCTACCGGTAAGGAAGGCGGA

                   3310      3320      3330      3340      3350      3360      3370      3380      3390      3400
               ....|....|....|....|....|....|....|....|....|....|....|....|....|....|....|....|....|....|....|....|
KF313135       GACTGGACAATACCGGATGGTCCAAGACTTAAGAGAGGTGAATAAAAGGGTGGAAACCATTCACCCCACGGTTCCTAACCCTTACACCCTTCTTAGCCTC
SUOHC3006      CTCTAAAGACTTTAGATTGGTGCAGGACTTAAGAGAGGTAAACAAGAGGGTGGAGACCATACACCCCATGGTCCCAAATCCCTACACCCTCCTAAGCCTG

                   3410      3420      3430      3440      3450      3460      3470      3480      3490      3500
               ....|....|....|....|....|....|....|....|....|....|....|....|....|....|....|....|....|....|....|....|
KF313135       CTGCCCCCCGATCACATCTGGTATAGCGTACTCGACCTTAAGGACGCCTTCTTCTGCCTCCCTCTGGCCCCTGGGAGTCAACCATTGTTTGCCTTTGAAT
SUOHC3006      CTGCCTCCTGATCACGCATGGTACAGTGTCCTAGACTTGAAGGATGCTTTCTTCTGCCTCCCCCTGGCGCCAGCGAGCCAGCCCATATTTGCTTTCGAGT

                   3510      3520      3530      3540      3550      3560      3570      3580      3590      3600
               ....|....|....|....|....|....|....|....|....|....|....|....|....|....|....|....|....|....|....|....|
KF313135       GGAGTGACCCAGAAGAAGGGGAGTCAGGACAATTAACATGGACCCGGCTGCCCCAGGGGTTCAAAAACTCACCCACCCTATTTGACGAGGCCCTCAGCCA
SUOHC3006      GGATCGACCCCAAAGAAGGGGAGTCGGGCCAACTGACATGGACTAGACTGCCTCAGGGTTTCAAAAACTCTCC---------------------------

                   3810      3820      3830      3840      3850      3860      3870      3880      3890      3900
               ....|....|....|....|....|....|....|....|....|....|....|....|....|....|....|....|....|....|....|....|
KF313135       GGCTAAAAGGAGGGACTAGGACCCTAACAGAAAGCCGAATAAAAGCGATCGTCCAGATTCCCTCTCCCAAAACCAAGAGACAGGTACGCGAGTTCTTGGG
SUOHC3001      -----------GGGAAGAGGACACTGGCTAACAGCCGGATCCAAGCCATTCTACAGATCCCCGCCCCCACCACTAAGAGGCAAGTCAGGGAATTCTTAGG
```

```
                   3910      3920      3930      3940      3950      3960      3970      3980      3990      4000
           ....|....|....|....|....|....|....|....|....|....|....|....|....|....|....|....|....|....|....|....|
KF313135   CACGGTCGGGTACTGCAGGCTGTGGATCCCGGGGTTCGCAGAACTAGCGAAGCCCCTGCATGCAGTAGCCGGAGGGGGTGCCAGACCCCTGACATGGACT
SUOHC3001  GGCGGTGGGGTACTGCAGGCTCTGGATCCCGGGCTTCGCAGAACTAGCCAGGCCCCTTCACTCCGCAACGGCTGGAGGAAATAAGCCCCTCCTATGGACA

                   4010      4020      4030      4040      4050      4060      4070      4080      4090      4100
           ....|....|....|....|....|....|....|....|....|....|....|....|....|....|....|....|....|....|....|
KF313135   AAGACCGAAGAAGAGGCATTCCAGGCCCTAAAGAGCGCTCTCCTGCAAGCCCCAGCTCTCAGCCTCCCCGACCTTGAAAAGCCCTTCCAGTTATTTGTGG
SUOHC3001  GAGACAGAGGAACAGGCATTCCAAGCCCTTAAGGTCGCCCTCCATCAAGCCCCGGCCCTAGCCCTACCGGACTTAGACAAGCCATTCCAACTCTTCATAG

                   4110      4120      4130      4140      4150      4160      4170      4180      4190      4200
           ....|....|....|....|....|....|....|....|....|....|....|....|....|....|....|....|....|....|....|
KF313135   CAGAGAACAAGGGGGTAGCAAAAGGGGTGCTCACCCAAAGGATAGGGCCCTGGAAAAGACCAGTAGCCTACCTCTCAAGAAAACTAGACCCAGTAGCTGC
SUOHC3001  CTGAAGGGCAAGGGATAGCTAAGGGGGTACTCACTCAGAGATTGGGACCCTGGAAAAGACCAGTAGCCTACCTAAGAGG----CTGGACCCGGTGGCTGC

                   4210      4220      4230      4240      4250      4260      4270      4280      4290      4300
           ....|....|....|....|....|....|....|....|....|....|....|....|....|....|....|....|....|....|....|
KF313135   TGGATGGCCTGGATGCCTACGGGCCATCGCAGCCGCTGCCCTCCTGGTCAAGGAAGCATCAAAACTCACCTTTGGACAAAACTTAGAGGTCACCTCGGCC
SUOHC3001  CGGATGGCCAGGTTGTCTCCGAGCCATCGCGGCGGCAGCCATTCTTGTGAAGGAAGCCACTAAGCTGACCTTTGG-------------------------
```
