# Supplementary material for: A Novel Coronavirus and a Broad Range of Viruses in Kenyan Cave Bats
Source: Viruses. 2022 Dec 17;14(12):2820. doi: 10.3390/v14122820 (PMC9785147; doi:10.3390/v14122820)
Supplement: Supplementary file 1 [file viruses-14-02820-s001.zip › FigureS7.pdf]

Supplementary Figure S7: Alignment of the murine leukemia virus-related virus (MLRV) sequences detected in bats. Positions are indicated according to the MLRV strain TaEV (GenBank accession: X99934).

```
                  10        20        30        40        50        60        70        80        90       100
          ....|....|....|....|....|....|....|....|....|....|....|....|....|....|....|....|....|....|....|....|
X99934    ACGGGGGCGGAACACTCTGTGGTGACACAAAAGGTGGCACCCCTATCAGGGTAGGAAGTTACTATCTTGCGGGCCACCAGAACCAAAACCCATAGACCAT
3008      --------------------------------GGTGGCACCCCTATCAGGGCGTGAAGTTACCATCGTGGGGTCTACCAGAATCAAAGCCCATAGACCAT
3007      ACAGGGGCCGAACACTCTGTGGTGACACAAAAGGTGGCATGTCTATCAGGGTGGGAAGTTACTATCTTGGGGACTACTGGAACCAAAACCCATAGACCAT
3006      ----------------------------------------------------------------------------------------------------
3005      ------------------------------AAGGTGGCACCCCTATCAGGGTGGGAAGTTACCATCATGGGGGCTACCATAACCAAAACCCACAGACCAT
3004      ----------------------TGACACAATAGGTGGCACCCCTATCAGGGCGGGAAGTTACCATTGTGGGGGCCACCGGAACCAAAACCCATAGACCAT
3003      --------------------------ACAAAAGGTGGCACCCCTATCAGGGCAGGAAGTTACCATCTTGCGGGCCTACCGGAATCAAACTCATAGACCAT
3002      ----------------------------------------------------------------------------------------------------
3001      ---------------------------------------------------------------------------ACCAGAACCAAAACCCATAGACCAT
2004      --------------------------ACAAAAGGTGGCACCCCTATCAGGGCAGGAAGCTACCATCGTGGGGGCTACTGGAACCAAAACCCGTAGACCAT
2002      ----------------------------------------------------------------------------------------------------
2001      ---GGGGCGGAACACTCTGTGGTGACACAAAAGGTGGCACCCCTATCAGGGCAGGAAGCTACTATCATGGGGGCTACCAGAACCAAAACCCATAGACCAT
1011      -------------------------------------CACCCCTATCAGGGCGGGAAGTTACCAATGTGGGGGCTACCAGCAACAAAGCCCATAGACCAT
1007      ---GGGGCTGAACACTCTGTGGTGACACAAAAGGTGGCACCCCTATCAGGGCGGGAAGTTACCATCGTGGGGGCTACCAGAACCAAAGCCCATAGACCAT
1006      ----------------------------------------------------------------------------------------------------
1005      ---------------------------------------------------------------------------ACCAGAACCAAAGCCCATACACCAC
1004      ----------------------------------------------------------------------------------------------------
1001      ----------------------------------------------------------------------------------------------------

                 110       120       130       140       150       160       170       180       190       200
          ....|....|....|....|....|....|....|....|....|....|....|....|....|....|....|....|....|....|....|....|
X99934    TTTGTGACCTCTGGAAATGCTGGTTGGGAGGCCACCAGGTGGTTCATGAGTTCCTGTATCTACCAGACTGCCCAGTACCGCTGTTGGACAGGGACCTCCT
3008      TTTGCAGCCCCCGGAAATGCTGGTTGGGAGGCCACCAGGTGGTCCATGAGTTCCTGTATCTACCAGACTGCCCAGTACCGCTGTTGGGCAGGGACCTCCT
3007      TTTGTGGTCCTTGGCAATGCCACTTAGGAGACCACCAGGTGCTTCATGAGTTCCTGTAGCCACGAGACCGCCAAGTACCACTGTTGGGCAGGGACCTCCT
3006      ----------------------------------------------------------------------------------------------------
3005      TTTGTGGTCCCTGGAAATACCAGCTGGGAGGCCACCGGGCGGTTCATGAGTTCCTGTATCTACCAGCGTGCCCAGTACTGCTGTTGTGCAGGGACCTCCT
3004      TTTGCGGCCCCCGGAAATGCCGGTTGGGAGGCCACCAGGTGGTTCATGAGTTCCTGTATCTACCAGACTGCCCAGTACCGCTGTTGGGCAGGAACCTCCT
3003      TTTGTGGCCCCTGGAAATGCAGGTTGGGAGGCCACTAGGTGGTTCATGAGTTCCTGTATCTACCAGACTGCCCAGTACTGCTGTTGGGCAGGAACCTCCT
3002      --------------------------GGAGGCTACCAGGTGGTTCATGAGTTYCTSTATCTACCAGACTGCCCAGTACCGCTGTTGGGCAGGGACCTCCT
3001      TTTGTGAGCTCCAGAATTGCTGGTTGGGAGGCCACCAGGTGGTTCATGAGTTCCTGTATCTACCAGACTGCCCAGTACTGCTGTTGGGCAGGGACCTCCC
2004      TTTGTGACCCCCGGAAATGCTAGTTGGGAGGCCACCAGGTGGTTCATGAGTTCCTGTATCTACCAGACTGCCCAGTACAGCTGTTGGGCAGGGACCTCCT
2002      ----------------------------------------------------------------------------------------------------
2001      TTTGCGGCCCCCAGAATTGCTGGTTGGGAGGCCACCAGGTAGTTCATGAGTTCCTGTATCTACCAGACTGCCCAGTACCACTGTTGGGCAGGGACCTCCT
1011      TTTGTGGTTCCCAGCAATGCCGCTTAGGAGGCCACCAGGTGGTCCATGGGTTCCTGTATCTACCAGACTGCCCAGTACCGCTGTTGGGCAGGGACCTCCT
1007      TTTGTGGCCCCCGGAAATGCCAGTTGGGAGGCCACCAGGTGGTTCATGAGTTCCTGTATCTACCAGACTGCCCAGTACCGCTGTTGGGCAGGGACCTCCT
1006      ----------------------------------------------------------------------------------------------------
1005      TTTGTGGCCCCCGGAAAGGCCAGTTGGGAGGCCACCAGGTGGTCCATGAGTTCCTGTATCTACCAGACTGCCCAGTACCGCTGCTGGACAGGGACCTCCT
1004      ----------------------------------------------------------------------------------------------------
1001      ----------------------------------------------------------------------------------------------------
```

```
                210       220       230       240       250       260       270       280       290       300
       ....|....|....|....|....|....|....|....|....|....|....|....|....|....|....|....|....|....|....|....|
X99934 GGCAAAGATGGGTGCAGAAATAGCTTTTGCTCTGGATGGGTCAGCACAACTGCACCTAGGTGAGGAGGCCTCTCCCATCATCCTGTCCTTGGCGGTACCA
3008   GGCAAAGATGGGTGCAGAAATAGCTTTTGCTCTGGATGGATCAGCACAACTGCGCCTAGGTGAGGAGGCCCCTCCCATGATCCTGTCCTTGGCGGTACCA
3007   GGCAAAGAGGGGTGCAGAAATAGCCTTTGCTCTGGATGGATCAGCACAACTGCACCTAGGTGAGGAGGCCCCTCCCATGATCCTATCCTTGGTGGTACCA
3006   ----------------------------------------------------------------------------------------------------
3005   GGCAAAGATGGGTTCAGAAATAGTTCTTGCTCCGGATGGATCAGCACAACTGCACCTAGGTGAGGAGGCCTCTCCCATGATCCTGTCCTTGGCGGTACCA
3004   GGCAAAGATGGGTGCAGAAATAGCCTTTGCTCCGGATGGGTCAGCACAACTGCACCTAGGTGAGGAGGCCCCTCCCATGATCC-----------------
3003   GGCAAAGATGGGTGCAGAAATAGCCTTTGCTCTGGATGGATCAGCACAACTGCACCTAGGCGAGGAGGCCCCTCCCATGATCCTGTCCTTGGCGGTACCA
3002   GGCAAAGATGGGTGTAGAAGCAGTCTTTGCTCTGGATGGTTCAGCACAACTGCGCCTAGGTGAGGAGGCCCCTCCCATGATCCTGTCCTTGGCGGTACCA
3001   AGCAAAGATGGGTGCAGAAATAGCCTTTGCTCTGGATGGGTCGGCATAACTGCTCCTAGGCAAGGAGACCCCTCCCATGATCCTGCCAAGGACAGGACCA
2004   GGCAAAGATGGGTGCAGAAATAGCCTTTGCTCTGGATGGATCGGCACAACTGTGCCTAGGTGAGGAGGCCCCTCCCATGATCCTGTCCTTGGCGGTACCA
2002   ----------------------------------------------------------------------------------------------------
2001   GGCAAAGATGGGTGCAGAAATAGCCTTTGCTCTGGATGGATCAGCACAACTGCACCTAGGCGAGGAGGCCCCTCCCATGATCCTGTCCTTGGCGGTACCA
1011   GGCAAAGATGGGTGCAGAAATAGCCTTTACTCCGGATGGATCAGCACAACTGCGCCTAGGCGAGGAGGCCCCTCCCATGATCCTGTCCTTGGCGGTACCA
1007   GGCAAAGATGGGTGCAGCACTGGCCDTTGATCTGGATCGATCGGCACAACTGCACCTAGGTGAGGAGGCCCCTCCCATGATCCTGTCCTTGGCGGTACCA
1006   ----------------------------------------------------------------------------------------------------
1005   GGCAAAGATGGGTGCAGAAATAGCCTTTGCTCTGGATGGATCGGCACAACTGCGCCTAGGTGAGGAGGCCCCTCCCATGATCCTGTCCTTGGCAGTACCA
1004   ----------------------------------------------------------------------------------------------------
1001   ----------------------------------------------------------------------------------------------------

                310       320       330       340       350       360       370       380       390       400
       ....|....|....|....|....|....|....|....|....|....|....|....|....|....|....|....|....|....|....|....|
X99934 CAGGAAGAAGAATGGAGGTTGTATACCTCCCCGCCTGATCAGCCAACTGTGGTCCTGGAACTGGAAGCTGAATTCCCATCAGTCTGGGCAGAAGAAAACA
3008   CGGGAAGAGGAATGGAGGTTGTATACCTCCCCGCCTGATGAGCCAATGGTGGCCCCGGAACTGGAGGCTGAATTCCCATCAGTCTGGGCAGAAGAAAACC
3007   CAGGAAGAAGAATGGAGGTTGTATACCTCCCCGCCTGATGAGCCAGTGGTGGCCCCAGAACTGGAGGCTGAATTCCCATCAGTCTGGGCAGAAGAAAACC
3006   ----------------------------------------------------------------------------------------------------
3005   TGGGAAGAGGAATGGAGGTTGTATACCTCCCTGTCTGATGAGCCAACAGTGGCCCCAGAACTGGAGGCTGAATTCCCATCAGTCTGGGCAGAAGAAAACA
3004   ----------------------------------------------------------------------------------------------------
3003   TGGGAAGAGGAATGGAGGTTGTATACCTCCCCGCCTGATGAGCCAACGGTGGCCCCGGAACTGGAGGCTGAATTCCCATCAGTCTGGGCAGAAGAAAACC
3002   TGGGAAGAGGAATGGAGGTTGTATACCTCCCTGCTTGATGAGTCAACAGTGGCCCCGAAAACGG-GGCTGAATTCCCATCAGTCTGGGCAGAAGAAATCC
3001   CAGGAAGAAGAATGGAGGTTGTATACCTCCCCGCCTGATGAGCCAACGGTGGCCCTGGAACTGGAGGCTGAATTCCCATCAGTCTGGGCAGAAGAAAAC-
2004   CAGGAAGAAGAATGGAGGTTGTATACCTCCCCGCCTGATGAGCCAATGGTGGCCCCGGAACTGGAGGCTGAATTCCCATCAGTCTGGGCAGAAGAAAACC
2002   ----------------------------------------------------------------------------------------------------
2001   CGGGAAGAGGAATGGAGGTTGTATACCTCCCCGCCTGATGAGCCAACGGTGGCCCCGGAACTGGAGGCTGAATTCCCATCAGTCTGGGCAGAAGAAAACC
1011   TGGGAAGAAGAATGGAGGTTGTATACCTCCCCACCTGATGAGCCAACAGTGGACCCAGAACTGGAGGCTGAATTCCTATCAGTCTGGGCAGAAGAAAACC
1007   CGGGAAGAAGAATGGAGGTTGTATACCTCCCCGCCTGATGAGCCAACGGTGGCCCTGGAACTGGAGGCTGAATTCCCATCAGTCTGGGCAGAAGAAAACC
1006   ----------------------------------------------------------------------------------------------------
1005   CGGGAAGAGGAATGGAGGTTGTATACCTCCCCGCCTGATGAGCCGACGGTGGCTCTGGAACTGGAGGCAGAATGCCCATCAGTCTGGGCAGAAGAAAGCC
1004   ----------------------------------------------------------------------------------------------------
1001   ----------------------------------------------------------------------------------------------------

                410       420       430       440       450       460       470       480       490       500
       ....|....|....|....|....|....|....|....|....|....|....|....|....|....|....|....|....|....|....|....|
X99934 CCACTGGC-----CAAAAACCATGCCCCAATCCTCATTGACCTGAAGCCTGGAGCCCAACCTGTCAAAATATGCCAGTATATGGTTCTGCTGGAAGCACG
3008   CCCCTGGTCTGGCCAAAAACCATGCCCCAATCCTGACTGACCTGAAGCCTGGAGCCCAGCCTGTCAAAATACGCCAGTACATGGTTCTACGGGAAGCGCG
3007   CTCCAGGTCTGGCCAAAAACCATGCCCCAATCCTGATTGACCTGAAGCCTGGAGCCYAACCTGTCAAAATAAGCCAGTACATTGTTCCACAGGAAGCACG
3006   ------------------------------------------------------------------------GCCAGGATGTGGTCCCGCAGGAAGCGCG
```

```
3005    CCCCTGGTCTGGGCAAAAACCATGCCCCGATACTGATTGACCTGAAGCCTTGAGCCCAGCCTGTCAAAATACGCCAGTACATGGTTCCGCGGGAAGCACA
3004    ----------------------------------------------------------------------------------------------------
3003    CCCCTGGC-----CAAAAACCATGCCCCAATCCTGATTGACCTGAAGCCTGGAGCCCAACCTGTCAAAATATGCCAGTACATGGTTCCGCGGGAAGCACG
3002    CCCCTGGTCTGGCCAAAAACCACACCCCGATCCTGATTGACCTGAAGCCTGGAGCCCAGCCTGTGAAAATACACCAGTACATGGTTCTATGGGAAGCGTG
3001    ----------------------------------------------------------------------------------------------------
2004    CCCCTGGC---GCCAAAAACCATGCCCCAATCCTGATTGACCTGAAGCCTGGAGCCCAACCTGTCAAAATACGCCAGTACATGGTTCCACGGGAAGCACG
2002    ----------------------------------------------------------------------------------------------------
2001    CCCCTGGTATGGCCAAAAACCATACCCCAATCCTGATTGACCTGAAGCCTGGAGCCCAGCCTGTCAAAATATGCCAGTACATGGTTCTGTGGGAAGCACA
1011    CCCTTGGTCTGGCCAAAAACCATGCCCCGATACTGAATGACCTGAAGCCTGGAGCCTGGCCTGTCAAAATACGCCAGTACATGGTTCTGCGGGAAGTGCG
1007    CCCCTGGC-----CAAAAACCACACCCCAATACTGATTGACCTGAAGCCTGGAGCCCAACCTGTCAAAATACGCCAGTACATGGTTCCGCGGGAAGTGCG
1006    ----------------------------------------------------------------------------------------------------
1005    CCCCAGGTC-AATCAGGAGTGGGGC----ATGGTTTTTGGCCAGAAGCCTGGGGGGCAACCTGTCCAAATACACCAGTACATGGTTCCATGGGAAGCACG
1004    ----------------------------------------------------------------------------------------------------
1001    -------------CAATAACCATGTCCCAATCCTGTTTGACCTGAAGCCTGGAGCCTAACCTGTTAAAATGCGCCAGTACATGGTTCCGCAGGAAGCACA

               510       520       530       540       550       560       570       580       590       600
        ....|....|....|....|....|....|....|....|....|....|....|....|....|....|....|....|....|....|....|....|
X99934  TCTAGGGATCCAGATTCACCTAGACCGATTACTCAAGTGCGGACTTTTAAAGCAATGCCAGTCCCCATGGAACACCCCTCTGCTGCCCATAAAGAAACCT
3008    TCTAGGGATCCAGATTCACCTAGACCGACTACTCAAGTGGGGACTTTTAAAGTAATGCCAGTCCCCGTGGAACACCCCTCTGCTGCCCATAAAGAAACCT
3007    TCTAGGGATCCAGATTCACCTAGACCGATTACTCAAGTGGGGACTTTTAAAGCAATGCCAGTCCCCATGGAACACCTCCTTGCTGCCTGTAAAGAAACCT
3006    TCTAGGGATCCAGADTCACCTAGACCAAGTACTCAAGTGGGGACTTTTAAAGCGATGCCAGTCCCCATGGAACACCCCCCTGCTGCCCATAAAGAAACCT
3005    TCTAGTGATCCAGATTCACCTAGACCAACTACTCACGTGGGGACTTTTAAGTCAATGTCAGTCCCCATGGAACACCCCCTGCTGCCTGTAAAGAAACCTG
3004    ----------------------------------------------------------------------------------------------------
3003    TCTAGGGATCCAGATTCACCTAGACCGACTACTCAAGTGGGGACTTTTAAAGCGATGCCAGTCCCCATGGAACACCCCTCTGCTGCCCATAAAGAAACCT
3002    TCTAGGGATCCAGATTCACCTAAACTGACTACTCAAGTGGGGACTTTCAAAGC-----------------------------------------------
3001    ----------------------------------------------------------------------------------------------------
2004    TCTAGGGATCCAGATTCACCTAGACCGACTACTCAAGTGGGGACTTTTAAAGCAATGCCAGTCCCCATGGAACACCCCCCTGCTGCCCGTAAAGAAACCT
2002    -------------------------------------------------------TGCCAGTCCCCATGGAACACCCCCCTGCTGCCTGAAAAGAAACCT
2001    TCTAGGGATCCAGATTCACCTAGACCGACTACTCAAGTGGGGACTTTTAAAGCGATGCCAGTCCCCATGGAACACCCCCCTGCTGCCCATAAAGAAACCT
1011    TCTAGGGATCCAGATTCACCTATACCAAGAACTCAAGTAGGAACTTTCAAAGCAATGCCAGTCTTCGTGGAACATCCACCTGCTGCCCGTAAAGAAACCT
1007    TCTAGGGATCCAGATTCACCTAGACCAACTACTCAAGTGGGGACTTTTAAAGCAATGCCAGTCCCTGTGGAACACCCCCCTGCTGCCCATAAAGAAACCT
1006    ----------------CACCTAGACCAACTACTCACGTGGGGACTTTTAAGTCAATGTCAGTCCCCATGGAACACCCCCCTGCTGCCCATAAAGGACCCT
1005    TCTAGGGATCCAGATTCACCTAGACTGACTACTCAAGTGGGGACTTTTAAAGCGATGCCAGTCCCTGTGGAACACCCCCCTGCTGCCCATAAAGAAACCG
1004    -----------------------------------------------------------AGTCCCCGAGGGACACACCCCTGCTGCCTGTAAAGAAACCT
1001    TCTAGGGATCCAGATTCACCTAGACTGACTACTCATATGGGGACTGGTAAAGCAATGCAAGTCCCCATGGAACACCCCCCTGCTGCCCATAAAGAAACCT

               610       620       630       640       650       660       670       680       690       700
        ....|....|....|....|....|....|....|....|....|....|....|....|....|....|....|....|....|....|....|....|
X99934  GGGACAAACAACTACCGGCCGGTACAAGACTTAAGGGCCATTAATGAAGTGGTCATCATGTTGCATTTGACACTGCCTAACCCATACACTCTGTTAGGAC
3008    GGGACAAACGACTACCACCCAGTACAAGACCTAAGGACCGCTTCATTAATGGTCATCATGTTGCATTCGGCACTGCCTAACCCATACACTCTATTAGGAC
3007    GGGACAAATGACTACCGCCCGGTATAAGACTTAAGGGCCATTAATGAAGTGGTCATCATGTTGCATTCGGCACTGCCTAACCCATACACTCTATTAGGAC
3006    GGGACAAACGACTACCGCCCGGTACAAGACTTAAGGGCCATTAATGAAGCGGTCATCACGTTGCATTCGGCACTGCCTAACCCATACACTCTGTATGGAC
3005    GGACAAA---------------------------------------------------------------------------------------------
3004    ----------------------------------------------------------------------------------------------------
3003    GGGACAAACGACTACCGCCTGGTACAAGACTTAAGGGCCATTAATGAAGTGGTCATCACGTTGCATTTGGCACTGCCTAACCCATACACTCTATTAGGAC
3002    ----------------------------------------------------------------------------------------------------
3001    ----------------------------------------------------------------------------------------------------
2004    GGGACAAACGACTACCGCCCGGTACAAGACTTAAGGGCCATTAATGAAGTGGTCATCACGTTGCATTCGGCACTGCCTAACCCATACACTCTATTAGGAC
```

```
2002      GACATAAATGACTACCTCTCAGTACAACACTTAACAGCCATTAACGAAGCGGTCATCACGTTGCATTCAGCACTGCCTAACCCATACACTCTATTAGGAC
2001      GGGACAAATGACTACCGCCTGGTACAAGACTTAAGGGCCATTAATGAAGCGATCATCATGTTGCATTCGGCACTGCCTAACCCATACACTCTGTTAGGAC
1011      GGGACAAACGACTACCGTCAGGTAAAAGACTTAAGGGCCATTAATGAAGCGGTCAATACGTTGCATTCGGCACTGCCTAACCCATACACTCTATTAGGAC
1007      GGGACAAATGACTACCACCCGGTACAAGACTTAAGGGCCATTAATGAAGTGGTCATCACGTTGCATTCGGCACTGCCTAACCCATACACTCTATTAGGAC
1006      GGGACAAATGACTACTGCCCAGTACATGACTTAAGGGCCATTAATGAAGCGGTCACCACATTGCATTCGGCACTGCCTAACCCAAACACTCTATTAGGAC
1005      GGGACAAACGACTACCGCCCGGTACAAGACTTAAGGGCCACTAATGAGGCGGTCATCATGTTGCATTCGGCACTGCCTAACCCATACACTCTATTAGGAC
1004      GGGACAAACGACTAACGCCCGGTACAAGACTTAAGGGTCTTTAATGAGGAGGTCATCACATTGCATTAAGCACTGCCTAACCCACACACTCTATAAGGAC
1001      GGGACAAATGACTACCGCCCGGTACAAGACTTAAGGGCCATTAATGAAGCGGTCATCACGTTGCATTCGGCACTGCCTAACCCATACACTCTATTAGGAC

                   710       720       730       740       750       760       770       780       790       800
          ....|....|....|....|....|....|....|....|....|....|....|....|....|....|....|....|....|....|....|....|
X99934    TTATACCTTCTGAGGCAGGGTGGTTTACATGCCTGGACTTAAAGGATGCCTTCTTCTGTCTCCAGCTAGCACCCAATAGCCAGCCTCTGTTTGCCTTCAA
3008      TTATACCTTCAGAGGCAGGGTGGTTCACATGACTGGACTTAAAGGATGCCTTTTTCTGCCTCCATCTAGCACCCAATACCCAGCCTCTGTTCACCTTCGA
3007      TTATAGCTTCTGAGGCAGGGTGGTTCACATGCCTGGACTTAAAGGATGCCTTCTTCTGCCTCCAACTAGCACCCAATAGCCAGCCTCTGTTTGCCTTCAA
3006      TTATTGTTCCTGTGGCAGGGTGGTTCACATGCCTGGGCTTAAAGGATGCCTTCTTCTGCCTCCAACTAGCGCCCAATAGCCAGCCTCTGTTCGCCTTTGA
3005      ----------------------------------------------------------------------------------------------------
3004      ----------------------------------------------------------------------------------------------------
3003      TTATACCTTCTGAGGCAGGGTGGTTCACATGCCTGGACTTAAAGGATGCCTTCTTCTGCCTCCAACTAGCGCCCAATAGCCAGCCTCTGTTAGCCTTCGA
3002      ----------------------------------------------------------------------------------------------------
3001      ----------------------------------------------------------------------------------------------------
2004      TTATACCTTCTGAGGCAGGGTGGTTCACATGCCTGGACTTAAAGGATGCCTTCTTCTGCCTCCAACTAGCGCCCAATAGCCAGCCTCTGTTCCCCTTCGA
2002      TTATACCCTCTGAGGCAGGGTGGTTCACATGCCTGGACTTAAAGGATGCCTTCTTCTGCCTCCAACTAGCGCCCAATAGCCAGCCTCTGCTCGCCTTCGA
2001      TTACACCTTCTGAGGCAGGGTGGTTCACATGCCTGGACTTAAAGGATGCCTTCTTCTGCCTCCAACTAGCGCCTAATAGCCATCCTCTGTTCGCCTTCGA
1011      CTATACCTTCTGAGGCAGGGTGGTTCACATGCCTGGACTTAAAGGATGCCTTCTTCTGCCTCCAGCTAGCACCCAATAGCGAGCCTCTGTTCGCCTTCGA
1007      TTATACCTTCTGAGGCAGGGTGGTTCACATGCCTGGACTTAAAGGATGCCTTCTTCTGCCTCCAACTAGCACCCAATAGCCAGCCTCTGTTCACCTTCAA
1006      TCATACCTTGTGAGGCAGAGTGGTTCACATGCCTGGACTTAAAGGATGCCTTCTTCTGCCACCCAATACCAGCCTCTGTCCAGCCTCTGTTGGCCTTCGA
1005      TTATACCTTCTGAGGCAGGGTGGTTCACATGCCTGGACTTAAAGGATGCCTTCTTCTGCCTCCAGCTAGTGCCCAATAGCCAGYCTCTGTTCACCTTCGA
1004      TTACACCTTCTGAGGCAGGGTGCTTCACATGCCTGGACTTAAAGGATGCCTTCTTCTGCCTCCAACTAGCGTCCAATAGCCAGCCTCTGTTTGCCTTCGA
1001      TTATACCCTCTGAGGCAGGGTGGTTCACATGCCTGGACTTAAAGGATGCCTTCTTCTGCCTCCAACTAGTGCCCAATAGCCAGCCTCTGTTCACCTTTGA

                   810       820       830       840       850       860       870       880       890       900
          ....|....|....|....|....|....|....|....|....|....|....|....|....|....|....|....|....|....|....|....|
X99934    ATGGGAAAGCCCCACCACAGGAACAAAGGAACAACTCACTCGGACTAGACTACCTCAAGGATTCAAGAATTCACCAACTTTGTTCAGTGGGGCACTAGCG
3008      ATGGGAAAACCTCACCACAGGAACAAAGGAACAATTCACTTGGAATAGACTACCTCAAGGATTCAAGAATTCATCAACTTGGTTCAGCAGGGCACTAGCG
3007      ATGGGAAAACCCCACCACAGGAACAAAGGGACAACCCACTTGGACTAGAAT---TCAAGGATTCAAGAACTCATCAACTTTGTTCAGCGGGGCACTAGTG
3006      ATGGGAAAACCCCACCACAGGAACAAAGGAACAATTCACTTGGACTAGACTACCTCAAGGATTCAAGAATTCACCAACTTTGTTCAGCGGGGCACTAGCG
3005      ----------------------------------------------------------------------------------------------------
3004      ----------------------------------------------------------------------------------------------------
3003      ATGGGAAAACCCCACCACAGGAACAAAGGAACAACTCACTTGGACTAGACTACCTCAAGGATTCAAGAATTCACCAGCTTTGTTCAGTGGGGCCCTAGCG
3002      ----------------------------------------------------------------------------------------------------
3001      ----------------------------------------------------------------------------------------------------
2004      ATGGGAAAACACCACCAAAGGAACAAAGGAACAATTCACTTGGACTAGACTACCCCAAGGATTCAAGAATTCACCAACTTTGTTCAGCGGGGCACTAGCA
2002      ATGGGAAAACCCCACCACAGGAACAAAGGAACAATTCACTTGGACTAGACTACCCCAAGGATTCAAGAATTCACCAGCTTTGTTCAGCGGGGCACTAGCG
2001      ATGGTAAACCCCCACCACAGGAACAAAGGAACAATTCACTTGGACTAGACTACCTCAAGGATTCAAGAATTCACCAACTTTGTTCAGTGGGGCACTAGCA
1011      ATGGGAAAACCCCACCACAGGAACAAAGGAACAATTCACTTGGACTAGACTACCTCAAGGATTCAAGAATTCACCAACTTTGTTCAGCAGGGCACTAGCA
1007      ATGGGAAAACCCCACCACGGGAACAAAGGAACAATTCACTTGGACTGGACTACCTCAAGGATTCAAAAATTCACCAGCTTTGTTCAGTGGGGTGCTAGCG
1006      ATGGGAAAACTCCACCACACAAACGAAGGAACAATTCACTTGGACTAGACTACCCCAAGGATTCAAGAATTCGCCAACTTTGTTCAGTGGGGCACTAGCG
1005      ATGGGAACACCCCACCACAGGAACAAAGGAACAA------------------------------------------------------------------
```

```
1004     ATGGGAAAACCCCACCACAGGAACAAAGGAACAATTCACTTGGACTAGACTACCCCAAGGATTCAAGAATTCACCAACTTTGTTCAGTGGGGCACTAGCG
1001     ATGGGAAAATCCCACCATAGGAACAAAGGAACAATTCACTTGGACTCAACTACCCCAAGGATTCAAGAATTCACCAGCTTTGTTTAGCAAGACACTGGCG

                 910       920       930       940       950
         ....|....|....|....|....|....|....|....|....|....|....|..
X99934   TCTGACTTAGCCAAGTTCCCGGGACTGAACGTAGGATGTGTCCTCCTCCAGTACGTC
3008     TCTGACTTAGCCAAGTTCCCGGGCTGGAACTTAGGATGTGTCCTC------------
3007     TC-------------------------------------------------------
3006     TCTGACTTAGCCAAGTTCCTGGGACAGAACTTAGGATGTGTCCTCCTCCAATATGT-
3005     ---------------------------------------------------------
3004     ---------------------------------------------------------
3003     TCTGACTTAGCCAAGTTCCCGGGACAGAACTTAGGATGTGTCCTCCTCCAATATGT-
3002     ---------------------------------------------------------
3001     ---------------------------------------------------------
2004     TCTGACTTAGCCAAGTTCCCAGGACGGAACTTAGGATGTGTCCTCCTCCAATACGT-
2002     TCTGACTTAGCCAAGTTCCTGGGACAGAACGTAGGATGTGTCCTCCTCCA-------
2001     TCTGACTTAGCCAAGTTCCCAGGACTGAACGTGGGATGTGTCCTCCTCCA-------
1011     TCTGACTTAGCCAAGTTACCAGGACAGAACTTAGGATGTGTCCTCCTCC--------
1007     TCTGACTTAGCCAAGTTCCCGGGATGGAACTTAGGATG-GTCCTCCTCCA-------
1006     TCTGACTTAGCCAAGTTCCCGGGATGGAACTTAGGATGTGTCCTCCTCCA-------
1005     ---------------------------------------------------------
1004     TCTGCCTTAGCCAAGTTCCCAGGATGGAACTTAGGATGTGTCCTCCTCC--------
1001     TCTGACTTAGCCAAGTTCCCGGGACAGAACTTAGGATGTGTCCTCC-----------
```
