# Supplementary material for: A Novel Coronavirus and a Broad Range of Viruses in Kenyan Cave Bats
Source: Viruses. 2022 Dec 17;14(12):2820. doi: 10.3390/v14122820 (PMC9785147; doi:10.3390/v14122820)
Supplement: Supplementary file 1 [file viruses-14-02820-s001.zip › FigureS8.pdf]

Supplementary Figure S8: Alignment of the Rhinolophus affinis foamy virus 1 sequence detected in a bat. Positions are indicated according to the reference strain (GenBank accession: JQ814855).

```
                  610       620       630       640       650       660       670       680       690       700
          ....|....|....|....|....|....|....|....|....|....|....|....|....|....|....|....|....|....|....|....|
JQ814855  CAACTTACTAATTCAGCGGTGCTTAGATCCGTCCCCCCTTCGAAAATACAACCACCTACCAAACCGTTTGATAAGTTCTATATTGATTATATTGGGCCTC
SUOHC1013 -----------------------------------------------------------TAAACCTTTTGAACTTTTTTACATAGATTATATAGGACCTC

                  710       720       730       740       750       760       770       780       790       800
          ....|....|....|....|....|....|....|....|....|....|....|....|....|....|....|....|....|....|....|....|
JQ814855  TTCCTCCTTCTTTCGGATATACATATGTTCTAGTTATTGTTGATGCTTGTACTGGATTTACTTGGTTATACCCCACTAAAGGTCCTACAGCCACCGCAAC
SUOHC1013 TTCCTCCTTCTCAAGGATATACTTATGTACTGGTTATTGTTGATGATGCTACAGGTTTTACATGGTTATACCCCACAAAGGGACCTACCACTGAGACCAC

                  810       820       830       840       850       860       870       880       890       900
          ....|....|....|....|....|....|....|....|....|....|....|....|....|....|....|....|....|....|....|....|
JQ814855  TGTCAAAGCACTCACTCATCTCTCTGGTACTGCAATTCCTCATGTAATTCATTCTGATCAAGGCCCTGCTTTTATTTCAGCAGTATTTAAAAATTGGGCT
SUOHC1013 TATCAAGGCTCTCACTAATCTCTTTGGTACTGCAATTCCAAGGAAAATACATTCAGATCAAGGACCTGCCTTTACTTCT---------------------
```
