# Supplementary material for: A Novel Coronavirus and a Broad Range of Viruses in Kenyan Cave Bats
Source: Viruses. 2022 Dec 17;14(12):2820. doi: 10.3390/v14122820 (PMC9785147; doi:10.3390/v14122820)
Supplement: Supplementary file 1 [file viruses-14-02820-s001.zip › FigureS9.pdf]

Supplementary Figure S9: Alignment of the astrovirus sequences detected in bats. Positions are indicated according to the bat astrovirus isolate BtAstV/21164-6-A/M.dau/DK/2015 (KF313135). GenBank accessions for sequences obtained in SYMN012 and SYMA008 are ON893139 and ON893140.

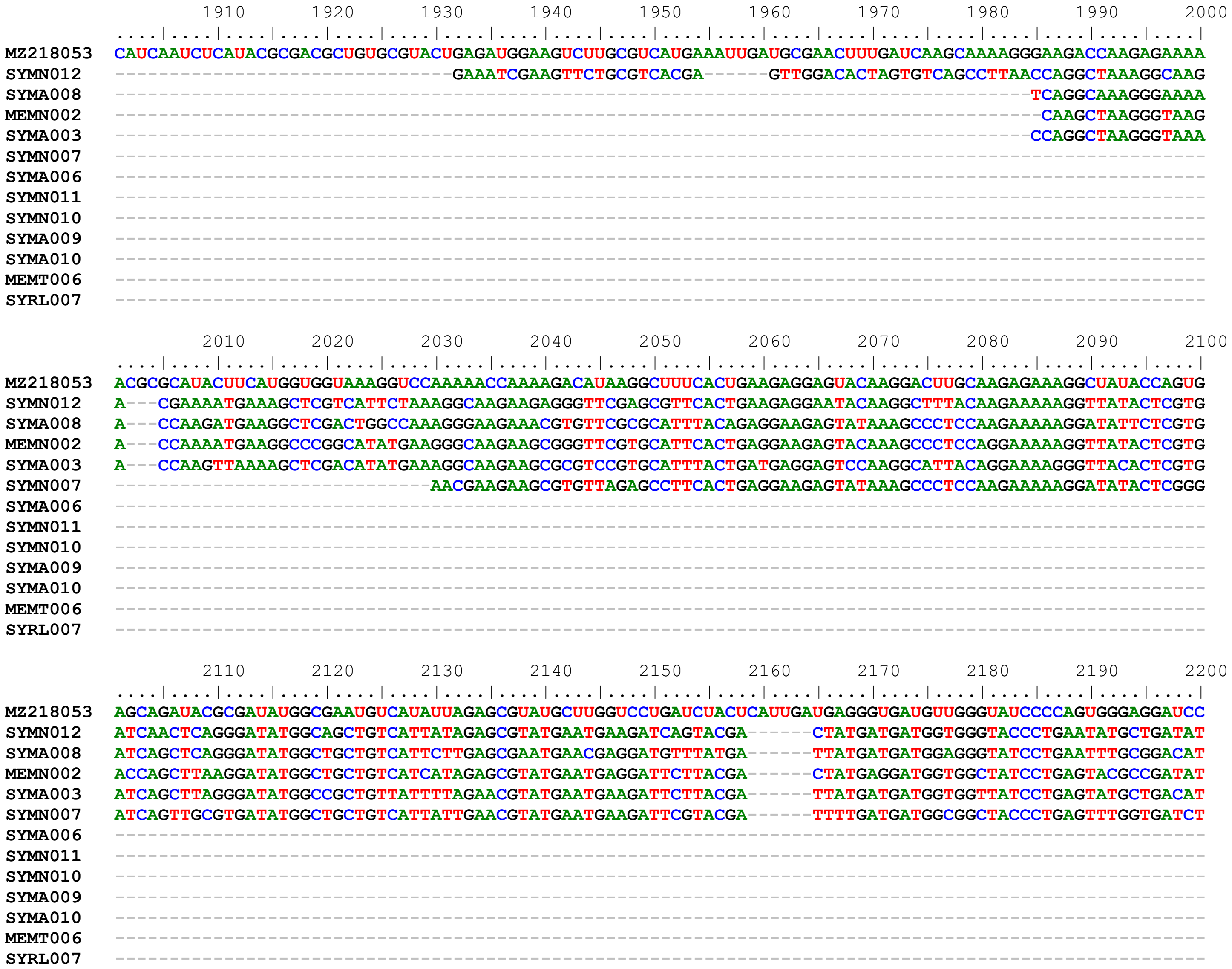

```
                  2210      2220      2230      2240      2250      2260      2270      2280      2290      2300
              ....|....|....|....|....|....|....|....|....|....|....|....|....|....|....|....|....|....|....|....|
MZ218053      CAACGAUGAGGAGCGGCGUGAAAUAGAACGUGAAUGGCUUGGUACCGAUGCUGAGCAUGAGGAUUAUGUUCGCAGUGGGUACCGUCAGUGUGCACCCGUU
SYMN012       AACCGATGAAGAGAGACGTGAAATAGAGAGAGATTGGCTTGGGACAGATGCCGAACATGAAGAGTATGTTCGTGATAGTTATCGACAATCTTATG-----
SYMA008       AACTGAAGAGGAGAAGCGTGAAATAGAGCGTGATTGGCTTGGTACTAATGAAGAACATGAGGATTATGTTCGGGAAAGCTATCGACAATCTTATG-----
MEMN002       TACTGATGAAGAACGACGTGAGATTGAGAGGGATTGGTTGGGTGCCAATGCTGAACATGATGATTATCTACGTGATAGTTATCGTCAGTCTTACG-----
SYMA003       AACTGATGAAGAGCGCCGGGAAATTGAGAGGGACTGGCTTGGCACTGATGCTGAACATGAAGACTACGTCCGGGAGAGTTACAGGCAGTCTTATG-----
SYMN007       TACGGAGGATGAAAAGCGTGAGATTGAACGAGATTGGCTTGGTACTGACACAGAGCATGATGATTATGTCAGGGACAGCTATCGACAATCATATG-----
SYMA006       ----------------------------------------------------------------------------------------------------
SYMN011       ----------------------------------------------------------------------------------------------------
SYMN010       ----------------------------------------------------------------------------------------------------
SYMA009       ----------------------------------------------------------------------------------------------------
SYMA010       ----------------------------------------------------------------------------------------------------
MEMT006       ----------------------------------------------------------------------------------------------------
SYRL007       ----------------------------------------------------------------------------------------------------

                  2310      2320      2330      2340      2350      2360      2370      2380      2390      2400
              ....|....|....|....|....|....|....|....|....|....|....|....|....|....|....|....|....|....|....|....|
MZ218053      CCUGAAACUCCCCCACCGCAACCAAAACGUGAAUACAAAGCAGACAAGAUCACAGUUGAGGCUGAUUUUAAUCCCAAAACACAUCCCAAUGAUGUGUAUG
SYMN012       -------------------------------------TTGTTGAGGCTGTCCCGGTTGAGAGTGATTACAATCCTAAAACACATCCATGGGATGTTTATG
SYMA008       -------------------------------------TTGTTGAGGCTGTTCCCGTTGAAASTGATTATAATCCCAAGACTCATCCTTGGGATGTTTATG
MEMN002       -------------------------------------TTGTTGAAGCTGTTCCTGTTGAGAGTGATTATAACCCTAAGACTCATCCTTGGGACGTGTATG
SYMA003       -------------------------------------TGGTTGAGGCTGTTCCTGTTGAGAGTGATTATAATCCCAAAACTCATCCTTGGGATGTTTATG
SYMN007       -------------------------------------TTGTTGAGGCTGTTCCTGTTGAGTCTGATTATAATCCGAAGACTCATCCTTGGGATGTTTATG
SYMA006       ----------------------------------------------------------------------------------------------------
SYMN011       ----------------------------------------------------------------------------------------------------
SYMN010       ----------------------------------------------------------------------------------------------------
SYMA009       ----------------------------------------------------------------------------------------------------
SYMA010       ----------------------------------------------------------------------------------------------------
MEMT006       ----------------------------------------------------------------------------------------------------
SYRL007       ----------------------------------------------------------------------------------------------------

                  2410      2420      2430      2440      2450      2460      2470      2480      2490      2500
              ....|....|....|....|....|....|....|....|....|....|....|....|....|....|....|....|....|....|....|....|
MZ218053      ACAAGUAUUCACUCAGCAUGUAUUACGUUACUGAUGCUGACGUUAAAUAUGUUGGUGAUGCCUUAACUGAUUAUGAUGAGUAUGUCACGGUUUGGAUGAU
SYMN012       ATAAATATTCTCTCTCTATGTATCACGTAACTGATGCCGATGTTAAGATCGTTGGTCAAGCTTTGTTGGATTACGATCGGTTCCTCACTAAGTGGATGGC
SYMA008       ATAAATATTCTTTGTGCATGTATCATGTCACTGARGCTGACGTTAAGATCGTTGGCCAAGCTCTTTTGGACTATGATCGCTTCTTAACAAAGTGGATGGC
MEMN002       ATAAATACTCTTTGTGTATGTATCATGTTACAGAAGCCGATGTTAAGATCGTTGGTCAGGCGTTGCTTGAYTATGATCGGTTTCTCACTAAGTGGATGGC
SYMA003       ATAAATATTCCCTGTGTATGTATCATGTCACTGAAGCTGATGTTAAGATTGTTGGTCAAGCTTTGCTTGATTACGATCGTTTCCTCACTAAGTGGATGGC
SYMN007       ATAAATATTCCTTGTGTATGTATCATGTTACTGAGGCTGATGTTAAAATTGTCGGTCAAGCCCTTCTTGATTATGATCGCTTTTTATGTAAGTGGATGGC
SYMA006       ----------------------------------------------------------------------------------------------------
SYMN011       ----------------------------------------------------------------------------------------------------
SYMN010       ----------------------------------------------------------------------------------------------------
SYMA009       ----------------------------------------------------------------------------------------------------
SYMA010       ----------------------------------------------------------------------------------------------------
MEMT006       ----------------------------------------------------------------------------------------------------
SYRL007       ----------------------------------------------------------------------------------------------------
```

```
                    2510      2520      2530      2540      2550      2560      2570      2580      2590      2600
           ....|....|....|....|....|....|....|....|....|....|....|....|....|....|....|....|....|....|....|....|
MZ218053   GCGGAACCUAACAUCUAAUGGUGAGUGGAGAACUGGUAUUGACCCUGUUACUGCCCUUAAGGAGCUGUCUGGUAAGAAAUAUGAAUUGGAAUGUAAGAUG
SYMN012    CCGTAATCTTCGTGGTAA---TGAATGGCAGTCTGGCATAGATATTGCTAAAGAATTGAAAGAACTAGCGGATGCCAAATTGCGTCTAGAAATCTTGATG
SYMA008    TCGGAACCTTCGTGGAAA---CGAGTGGCAGTCTGGCATTGATATAGCTAAGGAATTGAAAGAATTGGCGGATGCTAAATTGAGACTTGAAATCTTGATG
MEMN002    TCGCAATTTGCGTGGTAA---TGAGTGGCAATCTGGCATTGATATTGCTAAAGAGCTGCGTGAGCTTGCGGACGCTAAATTACGTCTAGAAATCTTAATG
SYMA003    TCGGAATTTGAGAGGCAA---TGAGTGGCAATCTGGTATTGACATCGCAAAAGAGTTGAAAGAGCTCGCGGATGCCAAATTGCGTCTAGAAATCTTGATG
SYMN007    TCGTAACTTGCGTGGCAA---TGAGTGGCAATCTGGCATCGATATTGCTAAGGAGTTAAAAGAATTGGCGGACGCTAAATTACGTCTTGAGATTTTGATG
SYMA006    ----------------------------------------------------------------------------------------------------
SYMN011    ----------------------------------------------------------------------------------------------------
SYMN010    ----------------------------------------------------------------------------------------------------
SYMA009    ----------------------------------------------------------------------------------------------------
SYMA010    ----------------------------------------------------------------------------------------------------
MEMT006    ----------------------------------------------------------------------------------------------------
SYRL007    ----------------------------------------------------------------------------------------------------

                    2610      2620      2630      2640      2650      2660      2670      2680      2690      2700
           ....|....|....|....|....|....|....|....|....|....|....|....|....|....|....|....|....|....|....|....|
MZ218053   CUUGAUCAUGGGCUUGUUCCUUUUUGUCAACGGAAGAAGAAGGAAAAGCGGCCCCGUAACAUCAAAAAAGCAUCAAAAAACGUGACCAGCCCCCCGAGUC
SYMN012    TCAACTAATGGCTTAACACCTTTTTGCCAGAGGAAAAAGAAAGAACGCAAACCACGTACGATTAAGAAAGCATCAAAAAACTTGAAAACCCCCCCGGAGG
SYMA008    ACTCAAAATGGGTTAACACCTTTTTGTCAACGTAAAAAGAAGGAACGGAAGCCTAGGACTATTAAGAAACTACCAAAAAACCTGAAGACCCCCCCTGTGG
MEMN002    TCACAGAATGGATTGACACCTTTTTGCCAGAGGAAGAAGAAGGAGCGTAAACCTCGGGCTTTAAAGAAAGTTCAAAAAAACGTGAAGACCCCCCCGGAGG
SYMA003    ACTCAGAATGGATTAACACCTTTTTGCCAAAGGAAGAAGAAAGAACGTAAACCACGTACGATTAAGAAAAAACCAAAAAACGTGAAAACCCCCCCGGAGG
SYMN007    ACGCAGAATGGGTTGACTCCTTTTTGCCAACGGAAAAAAAAAGAAAGGAAACCCCGAGCTTTGAAAAAAGTTCCAAAAAACTTGAAGCTCCCCCCTGTGG
SYMA006    ----------------------------------------------------------------------------------------------------
SYMN011    ----------------------------------------------------------------------------------------------------
SYMN010    ----------------------------------------------------------------------------------------------------
SYMA009    ----------------------------------------------------------------------------------------------------
SYMA010    ----------------------------------------------------------------------------------------------------
MEMT006    ----------------------------------------------------------------------------------------------------
SYRL007    ----------------------------------------------------------------------------------------------------

                    2710      2720      2730      2740      2750      2760      2770      2780      2790      2800
           ....|....|....|....|....|....|....|....|....|....|....|....|....|....|....|....|....|....|....|....|
MZ218053   CGGGGGG---------CACAGAGUAACUUACACAGACAUGCACCUUGAAGCUUGGAAAGCCGAUUUGGAUUUGCCUAAACGAAUUGUUGUACCCAAGCCC
SYMN012    GG---------------GGGGCATCGAATACAGCATGATGAAGTTAGATGCTTGGCGCTCGTATCTAGAAGATCCAAAGCGCATTGTCGTACCTGAAAAT
SYMA008    AGGGGGG------GACAGGCACATCACCTACCCAATGATGAAGTTGGGTGCGTGGCGCTCATATGTTAATGATCCTGAGCGCATTGTAGTCCCTGAAGGC
MEMN002    GG---------------GGGGAATCACCTACTCAATGATGAAGTTAGGTTACTGGCGCTCTTATGTTGAAGATCCTGAGCGCATAGTTGTTCCTGATAAT
SYMA003    GG---------------GGGGCATCACCTACTCAATGATGAAGTTAGATGCTTGGCGTTCATATGTTAATAAGCCTGAACGCATAGTTGTTCCTGATAAC
SYMN007    AGGGGGGGGAGAAGGGAACAACATCTCCTACTCAATGATGAAGCTCGGTGCTTGGCGCGACTATTTGTGTGATCCATGTCGCGTTGTAGTTCCTGATAAT
SYMA006    ----------------------------------------------------------------------------------------------------
SYMN011    ----------------------------------------------------------------------------------------------------
SYMN010    ----------------------------------------------------------------------------------------------------
SYMA009    ----------------------------------------------------------------------------------------------------
SYMA010    ----------------------------------------------------------------------------------------------------
MEMT006    ----------------------------------------------------------------------------------------------------
SYRL007    ----------------------------------------------------------------------------------------------------
```

```
                   2810      2820      2830      2840      2850      2860      2870      2880      2890      2900
           ....|....|....|....|....|....|....|....|....|....|....|....|....|....|....|....|....|....|....|....|
MZ218053   UUUCCUGUAAUUGGUAACAUAGACAUUGAUAGGCCCAUUUCUGACUGGAGUGUUGCUAAAGACCCACUGAUUAAUCUCCUUCCUCGUCCACCAUCUUAUA
SYMN012    TTTCCTGTTATTGGTTGTGTTCAAATTGATAGGCCTATTAGTGATTGGTCTTCTCCTAAGGATCCTCTATTAGGTTTGCTACCTAGGCCTCCAAAGTATA
SYMA008    TTTCCTGTTATTGGTAATATTSAAATTGATAGACCTATTAGTGATTGGACAGTCCCACGGGATCCACTTCTCGGATTGTTACCAAAACCACCAAATTATA
MEMN002    TTTCCAGTTATTGGCAATGTCCAAATTGATAGGCCTATCAGTGACTGGTCTGCTCCGGTTGATCCATTAATTGGTTTATTGCCTAAACCTCCGCCTTATC
SYMA003    TTTCCTGTTATTGGAAATATCCAGATTGATAGGCCTATTAGTGACTGGGCTGAACCTAGAGATCCTCTGATTGGTCTTTTGCCTAAACCCCCTAATTATA
SYMN007    TTCCCTGTTATTGGCAATGTTCAAATTGATAGACCCATTAGTGACTGGACTATGCCATCTGACCCTTTGTTGGGTTTGTTACCTAAACCCCCACGTTATG
SYMA006    ----------------------------------------------------------------------------------------------------
SYMN011    ----------------------------------------------------------------------------------------------------
SYMN010    ----------------------------------------------------------------------------------------------------
SYMA009    ----------------------------------------------------------------------------------------------------
SYMA010    ----------------------------------------------------------------------------------------------------
MEMT006    ----------------------------------------------------------------------------------------------------
SYRL007    ----------------------------------------------------------------------------------------------------

                   2910      2920      2930      2940      2950      2960      2970      2980      2990      3000
           ....|....|....|....|....|....|....|....|....|....|....|....|....|....|....|....|....|....|....|....|
MZ218053   CUCCGCUUCAUGAGCCCACUGUGUGGGGUUAUGACGCAUAUGCAAAGAGCUUUGAGAAGUUCUUCUAUGCCGAACCAGAUAGCGAUAUCAAAGGUAAUUA
SYMN012    ATGTTGAACATGGTCCATCAATATGGGGTTATGACGCATATGCTAAAAGTTTTGAAAAATTCTTTTATGCTGAGCCTTTAGAATCCATCAAGAATGTGTA
SYMA008    AAGATSATTATGGTCCATCTGTTTGGGGTTATGACGCATATGCTAAAAGTTTTGAAAAATTCTTTTATGCTGAGCCTTTAGAATCCATCAAGAATGTGTA
MEMN002    GTAACGATTATGGTCCTTCTGTCTGGGGTTATGATGCATATGCTAAAAGCTTTGAAAAGTTTTTCTATGCTGAACCTGTTGAATGTATTCAGAAGGCCTA
SYMA003    GTGTTGAGCACGGTCCTTCTGTGCGGGGTTATGACGCTTATGCTAAAAGTTTTGAGAAATTCTTTTATGCTGAGCCGGTTGAATCAATTAAGACTGTTTA
SYMN007    ACAACAAGTATGGACCCTCTGTTTGGGGTTATGATGCATACGCTAAAAGTTTCGAGAAATTTTTCTTTGCCGAACCTGTCGAATCCATACAGACAGTATA
SYMA006    ------AGTATGGTCCGTCCGTATGGGGCTACGATGCTTATGGTAAAAGTTTTGAAAAATTCTTTTATGCGGAGCCTCTACCTAGTATACGGACTGCTCA
SYMN011    -----------------------------------------------------------------TATGCGCAACCAATGGCCTCTATTCAGACAGCATA
SYMN010    ------------------------------------------------------------------------------------------AGAAGGCTCA
SYMA009    ----------------------------------------------------------------------------------------------------
SYMA010    ----------------------------------------------------------------------------------------------------
MEMT006    ----------------------------------------------------------------------------------------------------
SYRL007    ----------------------------------------------------------------------------------------------------

                   3010      3020      3030      3040      3050      3060      3070      3080      3090      3100
           ....|....|....|....|....|....|....|....|....|....|....|....|....|....|....|....|....|....|....|....|
MZ218053   CCCGCGUGAGUGGGUUUUUGCCAACAGGUGUUUGCUUAGGGAGUAUGGCUUCCUAAAAGGCACUCAUGUCAUUGACAUGACUGCCACUGUUAAAAAUGUU
SYMN012    CCCTAAGTATTGGGATTTTGCCAACAAATGTCTTATTAAAGAGTATGATTATTTAAAAAATTCTCATATGGTTGACATTGTGTCAACTGTAAAAAATGTT
SYMA008    CCCTAAGTATTGGGATTTTGCTAACAAGTGTCTGATTCGTGAGTATGATTATCTTAAGAACTCTCACATAATTGATATAACATCTACAGTTAAAAATGTT
MEMN002    CCCCAAGTATTGGGAGTTTGCTAATAGATGTCTTATTCGAGAATATGATTATCTAAAAAACTCCCATGTTATTGACATTACATCAACTGTCAAGAATGTG
SYMA003    TCCAAAATATTGGGAATTTGCAAACCGGTGGCTTATACGTGAGTATGACTATTTGAAAAACTCTCATGTTATTGATATCACATCTACTGTTAAGAATGTT
SYMN007    TCCTAAGTATTGGGAATTTGCCAATAAGTGTCTCATTAAGGAATATGGATATCTGAAGAATACGCACGTGGTTGATATTACATCAACTGTAAAAAATGTT
SYMA006    CCCACGTTGTTGGGAGTTTGCGAATAGGTGGCTTATTCGTGAGTTTGATTATCTAAAAAATTCCCATATCATTGATATAACTTCAACTGTTAAGAATGTT
SYMN011    TCCACGATGTTGGGAGTTTGCAAATCGTTGGCTCATACGTGAGTTTGACTATTTGAAGAATTCCCATGTTATTGATATTACATCTACTGTTAAAAATGTT
SYMN010    TCCCAAATGTTGGGAATTTGCTAGCCGTTGGCTTATACGGGAGTATGATTATTTAAAGAATACTCATGTTGTGGATATTACTGCAACTGTAAAAAATGTT
SYMA009    ----------------------------------ATTCGTGAGTTTGATTTTCTGAAGAATTCCCATGTCATTGATATTACATCTACTGTTAAAAATGTT
SYMA010    ------------------------------------------------------GAAGAATACCCATGTGATCGATATTACATCTACTGTTAAGAATGTT
MEMT006    ----------------------------------------------------------------------------------------------------
SYRL007    ----------------------------------------------------------------------------------------------------
```

```
                3110      3120      3130      3140      3150      3160      3170      3180      3190      3200
          ....|....|....|....|....|....|....|....|....|....|....|....|....|....|....|....|....|....|....|....|
MZ218053  GAAAGCACACCUGGUUACCCUAAGUUUCGCUACUGGAAGACUGAGGCUGAGUAUUUAGAGGAUAGAGGCUUCUGUGACUACAUCCAACAGCUUGAUGAAU
SYMN012   GATTCTACTCCTGCGTTTCCAAAATTCCTTTTCTGGCGCACAGAGGAGGAATATTTGGAAGATCGTGGGTATGCTGATTACATCAAGCAGTATAATGACA
SYMA008   GATTCTACCCCTGCTTTTCCTAAATTCCTCTATTGGAAAACTGAGGAAGAGTATTTGGAGGAAAGGGGGTATGCTGATTACATTCAACAATACAACGATT
MEMN002   GATTCAACTCCTGCTTTTCCTAAATTTCTCTTCTGGCGTACAGAGGAGGAATATTTGGAGGAGCGTGGGTATSCTGATTATATTAAACAGTATAATGATA
SYMA003   GATTCTACTCCTGCTTTTCCTAAGTTCCTTTTCTGGCGTACTGAAGAGGAGTATTTGGATGAGCGTGGTTATGCTGATTATATCCAGCAGTACAATGATA
SYMN007   CAATCTACACCAGCCTTTCCAAAATTCCTTTTCTGGAAAACGGAAGAGGAGTATTTGGAAGAACGTGGTTATGCTGATTACATACAGCAGTATAATGACA
SYMA006   GATTCTACTCCTGCTTTTCCGAAGTTCTTCTTTTGGAAGACTGAAGAGGAGTATTTGGAAGAGCGTGGGTATGCTGATTATATACAGCAGTATAATGATA
SYMN011   GATTCCACACCTGGCTTTCCAAAATTCCTCTTTTGGAAGACTGAGGAGGAGTATTTGGATGAGCGGGGTTATGCTGATTATATTCAACAGTATAATGACA
SYMN010   GATTCCACTCCCGCATTTCCGAAGTTCCTTTTTTGGCGTACGGAAGAGGAATATTTGGATGATCGTGGTTATGCTGATTATATTAGTCAGTATAATGACA
SYMA009   GATTCCACACCTGGCTTTCCAAAATTCCTCTTTTGGAAGACTGAGGAGGAGTACTTGGATGAGCGGGGTTATGCTGATTATATTCAACAGTATAATGACA
SYMA010   GATTCTACACCTGCTTTTCCTAAGTTCCTCTTCTGGCGTACTGAGGAGGAATATTTGGAGGAGCGTGGGTATGGTGATTATATTCAGCAGTTCAATGATA
MEMT006   ----------------------------------------------------------------------------------------------------
SYRL007   ----------------------------------------------------------------------------------------------------

                3210      3220      3230      3240      3250      3260      3270      3280      3290      3300
          ....|....|....|....|....|....|....|....|....|....|....|....|....|....|....|....|....|....|....|....|
MZ218053  UGAAAAAUGGUUCUAGACCCGACGUGCUGUGGUAUUUAUUUCUUAAGAAAGAAAUUCUUAAAAGUGAUAAGGUCGAAGCUGGUGACAUACGUCAAAUCGU
SYMN012   TTTGTGATGGTGAAAGACCCGATGTATTGTGGTACTTATTTTTAAAGAAAGAGATCTTGAAGATTGATAAAATTAAAGATTCCGATATAAGGCAAATTGT
SYMA008   TGGTTGCTGGTGAACGCCCAGATGTCCTTTGGTATCTCTTCTTGAAGAAGGAGATTTTAAAACTGGATAAAATTAAAGATTCAGATATCCGTCAGATAGT
MEMN002   TACTTGGTGGAGAAAGGCCTGATGTTCTCTGGTATCTCTTTTTGAAAA----------------------------------------------------
SYMA003   TTGGTAATGGTGAACGACCTGATGTTTTGTGGTATCTTTTTCTTAAGAAGGAGATTCTTAGGTTGGATAAGATAAAAGACTCAGATATTAGGCAAATAGT
SYMN007   TTGTGGCTGGTGAGCGTCCAGATGTCCTTTGGTACCTCTTCCTTAAGAAAGAGATACTTAAGTTGGAAAAAATTGATCAGTCTGATATTCGGCAAATTGT
SYMA006   TTGTTGCTGGTGAGCGACCTGATGTTTTTTGGTATCTTTTCCTGAAGAAGGAAATTCTGAAGTTGGATAAAATTAAGGATTCAGATATTCGTCAAATAGT
SYMN011   TAGTTGCGGGTGAGAGACCAGAAGTCCTGTGGTATCTCTTTTTGAAAAAAGAAATTCTTAAATTGGACAAGATTCGGGAATCTGATATCCGTCAAATAGT
SYMN010   TTGTCAATGGTGAGCGTCCCGATGTTTTGTGGTATTTATTTTTAAAGAAGGAGATTCTTAAGTTAGATAAGATCAAGGATTCTGATATAAGGCAGATCGT
SYMA009   TAGTTGCGGGTGAGAGACCAGAAGTCCTGTGGTATCTCTTTTTGAAAAAAGAAATTCTTAAATTGGACAAGATTCGGGAATCTGATATCCGTCAAATAGT
SYMA010   TAGTTGGCGGTGAGAGACCTAATGTTTTGTGGTACCTTTTCCTTAAGAAGGAGATTCTTAAGTTGGACAAGATCAAGGAATCTGATATTAGGCAAATTGT
MEMT006   ---------------------------------------------------------------------------------------------AGATTGT
SYRL007   ----------------------------------------------------------------------------------------------------

                3310      3320      3330      3340      3350      3360      3370      3380      3390      3400
          ....|....|....|....|....|....|....|....|....|....|....|....|....|....|....|....|....|....|....|....|
MZ218053  UUGUCCCGACCCUAUCUAUGCACGUGUCGGAGCGAUGUUCGAGCAAGAUCAGAACAAUCGAAUGAAAACCAUGACAAAGUGGAAACAUGGCCAAUGUGGA
SYMN012   GTGTCCTGACCCTATATTTTCCAGGATTGGTGCCATGTTTGAGCAAGATCAGAATAACCGTATGAAGGATATGACCCGATGGCGGCAATCTCAGTGTGGA
SYMA008   GTGTTCAGATCCAATTTTTGCCCGTATTGGTGCTATGTTTGAGCAAGATCAAAACAACCGTATGAAGGAGATGACCCGCTGGCGACAAGGTCAGTGTGGT
MEMN002   ----------------------------------------------------------------------------------------------------
SYMA003   GTGTCCTGACCCCATATTTTCTAGGATTGGTGCCATGTATGAACAAGATCAAAATAATCGTATGAAGGAGATGACAAGATGGAGGCAGTCCCAGTGTGGT
SYMN007   GTGTGCAGACCCTATTTATAGTCGGATTGGGGCTATGTTTGAACAAGACCAGAACAACCGAATGAAGAGTATGACGCGGTGGAAACAATCACAATGTGGT
SYMA006   TTGTGCAGATCCAATTTATGCTCGTATTGGTGCTATGTACGAGCAAGATCAAAATAACCGTATGAAGGAGATGACCCGTTGGCGTCAAGGTCAATGTGGG
SYMN011   GTGTTCAGACCCAATATATGCTCGTATTGGAGCAATGTATGA----------------------------------------------------------
SYMN010   TTGTCCTGATCCTATTTTTTCTAGAATTGGTGCCATGTTTGAGCAAGATCAAAATAATCGGATGAAAAGTATGACACGGTGGAGGCAATCTCAGTGTGGT
SYMA009   GTGTTCAGACCCAATATATGCTCGTATTGGAGCAATGTATGAGCAAGATCAGAATAATCGGATGAAGGAGATGACCCGTTGGCGTCAAGGCCAATGTGGT
SYMA010   GTGTCCTGATCCTATTTTCTCTCGTATTGGTGCCATGTATGAACAAGATCAGAATAACCGGATGAAGGACATGACAAGGTGGCGTCAGTCACAGTGTGGA
MEMT006   GTGTCCTGATCCCATCTATGCTAGGATAGGTGCCATGTTTGAGCAAGATCAGAATAACAGGATGAAGAACATGACAAGGTGGAAACATGGACAGTGTGGT
SYRL007   ----------------------------------------------------------------------------------------------------
```

```
                   3410      3420      3430      3440      3450      3460      3470      3480      3490      3500
           ....|....|....|....|....|....|....|....|....|....|....|....|....|....|....|....|....|....|....|....|
MZ218053   UGGUCACCCUUUGAGGGUGGCUUUGAUGAGGUUCUUCGCCGCCUCGAGCGUGAUGACAAUCAGUACAUUGAGUUGGAUUGGACUCGGUAUGAUGGUACUA
SYMN012    TGGTCGCCTTTTGAGGGTGGCTTCCATGAGGTTATTTCTAGGTTAGACCGTAGTGGTCATACCTATATCGAGTTCGATTGGACTCGTTATGATGGCACCA
SYMA008    TGGTCCCCTTTTGAAGGTGGCTTTCATCATGCCATTTCACGGTTGGAACGTAAGGGCAATGTTTACATAGAGTTTGATTGGACTCGCTATGATGGAACTA
MEMN002    ----------------------------------------------------------------------------------------------------
SYMA003    TGGTCTCCGTTTGAGGGTGGTTTCCATGATGTCATTTCTAGGTTAGAGCGTGATGGTAATACATACATAGAGTTTGATTGGACGAGATATGATGGCACAA
SYMN007    TGGTCCCCCTTTGAGGGGGGTTTCCATGAGGTTATCTCGCGTCTTGAGAAGCCTGGGAACCATTATATTGAGTTTGATTGGACCCGATATGATGGCACTA
SYMA006    TGGTCTCCCTTTGAGGGTGGCTTTCATGAAGTTATATCTAGATTAGATCGTCCGGGTAATAAGTATATTGAGTTTGATTGGACTCGATATGATGGTACCA
SYMN011    ----------------------------------------------------------------------------------------------------
SYMN010    TGGTCTCCCTTTGAGGGTGGCTTCCATGAGGTTGTTTCCCGTCTTGATCATTCAGGTCATACTTATATTGAGTTCGATTGGACTCGATATGATGGCACGA
SYMA009    TGGTCTCCTTTTGAAGGTGGCTTTCATCAAGCCATCTCCCGATTAGAACGTAAAGGTAATATCTATATAGAGTTTGATTGGACACGCTATGACGGAACTA
SYMA010    TGGTCCCCTTTTGAAGGGGGCTTCCATGAGGTCATCTCTAGATTAGATCGTTCCGGTAACAAATTTATTGAGTTTGATTGGACTCGATATGATGGCACCA
MEMT006    TGGTCACCCTTTGAGGGTGGCTTTGATGAGGTTCTTCGCCGTATTGAAAAGCCTGAAAATCAATATATTGAACTGGATTGGACTCGGTATGATGGCACTA
SYRL007    ----------------------------------------------------------------------------------------------------

                   3510      3520      3530      3540      3550      3560      3570      3580      3590      3600
           ....|....|....|....|....|....|....|....|....|....|....|....|....|....|....|....|....|....|....|....|
MZ218053   UUCCGCUUGAAGUGUUUCGACACAUCAAGUGGUUUAGGUAUCACAUGCUGGAUCCUGCCUACCACACAAGUGUUAAUAAGGGGAUAUACUCGUGGUACGU
SYMN012    TTCCTGTGGAGGTTTTTAGGCACATTAAGAATTTTAGGTTTAGCATGTTGGATCCAGTTTATAAGACCAAATTGAACAGAGACATTTATGATTGGTACAT
SYMA008    TACCTGTTGAGGTATTTAGGCATATTAAAACCTTTCGGTTTAGTATGTTAGATCCAGTTTATAAGACCACAGTTAATTCTTCGATCTACCAGTGGTACAT
MEMN002    ----------------------------------------------------------------------------------------------------
SYMA003    TCCCAACTGAGGTTTTTATACATGTTAAATCATTTAGGTTCAGTATGCTTGACCCAGTATATAAGACACCTCTTAATAGGGGCATTTATGATTGGTACGT
SYMN007    TCCCTGTTGAGGTTTTTCTACATATTAAAAGGTTTCGGTTTTCCATGCTCGATCCGGCTTATAAGAATGATACCCTTAGGTCCATTTATGATTGGTACGT
SYMA006    TTCCTGTTGAGGTTTTTAGGCATATAAAGAGTTTTAGATTTTCTATGCTGGATCCTGTTTATAAGACCACTTGTAATAAATCCATATATGACTGGTACGT
SYMN011    ----------------------------------------------------------------------------------------------------
SYMN010    TTCCAGTTGAGGTTTTCAGGCATATTAAGAACTTTAGATTTGCCATGCTTGACCCAGTCTATAAAACCACAGTCAATAAGGATGTTTATGATTGGTACGT
SYMA009    TTCCTGAGGAGGTTTTTAGGCATATTAAGTCTTTTAGATTTAGTATGCTTGACCCCGTTTATAAGAC---------------------------------
SYMA010    TACCTGTTGAGGTTTTCAGGCATATTAAGAATTTTAGGTTTAGTATGYTAGATCCAGTTTATAAAACAGATATGAACAGATCTATTTATGATTGGTACGT
MEMT006    TACCGCTTGAGGTGTTTCGACACATCAAGTGGTTTAGGTATCAGATGCTTGATTCTTCCTACCATACACCTGAAAATCGTGGTGTTTATGAATGGTATGT
SYRL007    -----------------------------------------------------------------------------------------------TATGT

                   3610      3620      3630      3640      3650      3660      3670      3680      3690      3700
           ....|....|....|....|....|....|....|....|....|....|....|....|....|....|....|....|....|....|....|....|
MZ218053   UGAUCAGAUGUGCCGCAGGCAUGUCCUCUUGCCUAGUGGUGAGGUCACUAUCCAGGAGCGUGGAAACCCUUCUGGUCAGAUCUCUACCACUAUGGAUAAC
SYMN012    TGGTCAACTTATGTACCGTTATGTTTTGTTACCATCTGGAGAAATCACAATTCAGGATAGAGGCAATCCTTCAGGTCAAATATCCACTACTATGGATAAT
SYMA008    TGGTCAACTTATGTACCGACATGTTCTCCTTCCTTCTGGTGAAGTTACCATACAAGATCGTGGCAATCCAAGCGGACAGATCTCCACAACTATGGACAAT
MEMN002    ----------------------------------------------------------------------------------------------------
SYMA003    TGGACAACTTATGTACCGTTATGTTCTTTTACCATCTGGAGAGATTACCATTCAAGATCGTGGTAATCCTTCTGGCCAAATTTCAACCACAATGGACAAT
SYMN007    TGGTCAGCTTATGTACCGTTATGTGTTGTTGCCTTCTGGAGAAATAACCATTCAAGATAGGGGTAACCCTTCAGGGCAAATTTCTACTACCACTGATAAT
SYMA006    TGATCAACTTATGTACCGTCATGTCCTTTTGCCTTCTGGAGAGATTACTATACAGAGTAGGGGTAATCCTAGTGGCCAAATTTCTACAACCATGGATAAT
SYMN011    ----------------------------------------------------------------------------------------------------
SYMN010    TGGTCAGCTATTGTACCGATATGTCCTTTTACCCTCTGGTGAGATTACCATTCAAGATCGTGGTAATCCATCCGGGCAGATTTCCACTACCATGGATAAT
SYMA009    ----------------------------------------------------------------------------------------------------
SYMA010    TGGTCAGCTTGTGTACCGTTATGTTCTTTTACCATCTGGTGAGGTCACTGTTCAGGATAAAGGTAATCCCTCTGGCCAAATTTCTACCACTATGGATAAT
MEMT006    AGATCAGATGTGTAGGCGACATGTTTTGCTGCCTAGTGGTGAAGTTACCGTGCAGGAGCGTGGTAACCCATCTGGTCAGATTTCTACAACTATGGATAAT
SYRL007    TTCTCAATTGTGTCATAGGTATGTTCTTTTGCCTTCAGGTGAAGTTACCATACAAACTAAGGGAAATCCCTCTGGACAGATTTCAACAACTATGGATAAC
```

```
                   3710      3720      3730      3740      3750      3760      3770      3780      3790      3800
           ....|....|....|....|....|....|....|....|....|....|....|....|....|....|....|....|....|....|....|....|
MZ218053   AAUAUGAUUAACACUUUUUUGCAAGCUUUUGAGUUUGCUUAUGUACAUCCAGAUUAUACUGAUUCCAUGCUUGAGGCUGAAUGGAAGAAAUGUGACACCA
SYMN012    AATTTGGTTAATACATTTTTGCAGGCTTTTGAGTTTGCGTATTTGAACCCCGAGTTATCTTTGGATGAACTCGATTGTTTATATGCGCAGTGTGACTCAT
SYMA008    AATATGGTTAATTGTTTTCTCCAGGCTTTTGAGTTTGCGTATTTGAATCCTGGTCTATCTATGGCTGAGTTAGACCTTTTATATGCGCAGTGTGATTCTT
MEMN002    ----------------------------------------------------------------------------------------------------
SYMA003    AATATGGTAAATACTTTCCTCCAGGCTTTTGAGTTTGCGTATTTGAATCCTGGGCTCTCTTTTGGAGAGCTCGATGATTTATATGCGCGTTCTGACTCTT
SYMN007    AATATGGTCAACACCTTTTTGCAAGCTTTCGAGTTTGCTTATCTTAATCCAGAGTTGTCTCTGGATGAATTAGAGGTGTTGTATAGTCAGGGTGATTCTC
SYMA006    AACATGGTTAATTGTTTCCTCCAGGCTTTTGAGTTTGCGTATTTGAATCCTGGCTTGTCCCAACACGAGCTTGATGGTTTATATGCGCAGGCAGACTCTT
SYMN011    ----------------------------------------------------------------------------------------------------
SYMN010    AATTTAGTCAACACTTTTTTGCAGGCTTTTGAGTTTGCGTATGTGAATCCTGAGCTCTCTATGGATGAGCTTGATATTTTGTATGCGCAATGTGACTCAC
SYMA009    ----------------------------------------------------------------------------------------------------
SYMA010    AATCTGGTTAACACTTTTTTGCAGGCTTTTGAGTTTGCGTATGTGAACCCTGAGCTCTCTTTGGATGAGCTTGATTGTTTGTATGCGCAGTGTGACTCAT
MEMT006    AACATGGTTAACACCTTTTTACAAGCATTTGAATTTGCCTATGTTCATCCAGACTATGATGATTCCAGGCTTGAGAGTGAGTGGAAGAAATGTGACACTA
SYRL007    AATATGGTCAATTATTTTTTGCAAGCTTTTGAGTTTGCTTATCTTCATCCTAGTTTTGGCGAAGATGCCCTTGCTTCTGCTTGGTCTAATACTGATACTT

                   3810      3820      3830      3840      3850      3860      3870      3880      3890      3900
           ....|....|....|....|....|....|....|....|....|....|....|....|....|....|....|....|....|....|....|....|
MZ218053   UCGUGUAUGGUGAUGAUAGGAUAUCAUCUUGGCCAUCUGUUCCUCCGAAUUAUGUUGAUACUGUUGUUGAGAUGUAUAAGGUUGUUUUUGGUAUGUGGGU
SYMN012    TAATTTATGGTGATGATAGATTATCATCGTGGCCCTGCATACCTGATAACTATCCTGAAAGTGTTTCTGCCATGTATGAACATGTTTTTGGAATGTGGGT
SYMA008    TAGTCTATGGAGATGATCGGTTGACATCATGGCCGAGTGTTCCCGATGATTATCCCAATGTGGTTTCCCGCATGTATGAACATGTCTTTGGCATGTGGGT
MEMN002    ----------------------------------------------------------------------------------------------------
SYMA003    TAATCTATGGAGATGATAGATTATCATCATGGCCTGTCGTCCCCGATAATTATCAAGAAGGTGTTTCTGCCATGTATGAGCATGTTTTTGGTATGTGGGT
SYMN007    TTATTTATGGAGATGATAGACTCACATCTTGGCCCTGTGTCCCTGAGGATTATCCTAACCTTGTCTCCAGAATGTATGAACATGTATTTGGCATGTGGGT
SYMA006    TAGTTTATGGAGATGATAGATTAACATCTTGGCCGTTTGTCCCCGATGATTATCCTAATTTGGTATCTCGCATGTATGAACATGTCTTTGGCATGTGGGT
SYMN011    ----------------------------------------------------------------------------------------------------
SYMN010    TTATATATGGAGATGATCGTTTGTCATCTTGGCCTGTTGTTCCTGATAATTATCAGGAGCTTGTATCATCTATGTATAAACATATCTTTGGTATGTGGGT
SYMA009    ----------------------------------------------------------------------------------------------------
SYMA010    TAATTTATGGTGATGATAGATTATCATCATGGCCTATTGTTCCTGATGATTATCCTGATCGTGTTTCCCGCATGTACGAACATGTCTTTGGCATGTGGGT
MEMT006    TTGTTTATGGCGATGATAGGATTTCATCTTGGCCYAGTGTTCCACCTAACTATGTTAATGAAGTTGTTGAGATGTATAAAATTGTTTTTGGTATGTGGGT
SYRL007    TGATCTACGGTGATGACCGTATCTCATCTT----------------------------------------------------------------------

                   3910      3920      3930      3940      3950      3960      3970      3980      3990      4000
           ....|....|....|....|....|....|....|....|....|....|....|....|....|....|....|....|....|....|....|....|
MZ218053   UAAGCCUGAAAAGAUAGUAGUUAGAAAUGAGUUAAGUGGUGUUUCUUUUUGUGGUUUUACAGCUAUCCUUUCUGAUGGUAUGUAUCUGCCUGUUCCUAAU
SYMN012    AAAACCAGATAAAGTTAAAGTCTCTGATACACTTGAAGGCCTCACTTTTTGTGGATTTACAGTTATTAGAAGTGGGGGTTACTATTTGCCTATACCTGCA
SYMA008    TAAGGAGAACAAAGTTAAAGTTTCTGATACATTAGAAGGGCTGACCTTTTGTGGATTTACTGTTATTCGATCTGGTGGGATGTATTTACCTGTTCCATCT
MEMN002    ----------------------------------------------------------------------------------------------------
SYMA003    CAAACCAGAAAAAGTTAAAGTCTCTGATACACTTGAAGGCCTGACTTTTTGTGGTTTTACTGTTATTAGTAGTGGCGGCTTTTATTTTCCTGTACCAGCT
SYMN007    TAAACCTGACAAAGTTAAGGTCTCAGATACGCTAGAAGGACTTACTTTCTGTGGTTTTACTGCTATTAAGAGTGATGGGCTTTATCTTCCTGTGCCCGTT
SYMA006    TAAGCCAGAAAAAAATTAAGGTATCAGACACCTTGGAAGGCTTGACCTTTTGTGGTTTTACTGTTATTCGATCTGGAGGTATGTATCTCCCTATTCCATCT
SYMN011    ----------------------------------------------------------------------------------------------------
SYMN010    TAAGCCCGACAAAGTTAAAATATCTGATACTCTTGAGGGTTTGACTTTTTGTGGTTTCACCATCATTCGTAGCGGGGGTTATTATCTTCCTGTTCCATCT
SYMA009    ----------------------------------------------------------------------------------------------------
SYMA010    TAAGGAGAACAAAGTTAAAGTTTCTGATACATTAGAAGGGCTTACTTTTTGTGGATTTACTGTTATTCGGAGTGGCGGATTTTATCTCCCTATTCCATCA
MEMT006    AAAACCTGAAAAGATTGTAGTCAGAAATGAGCTTAGTGGTGTTTCTTTTTGTGGGTTTACAGCTATCCTTTCTGATGGAATGTATTTGCCAGTGCCTAAT
SYRL007    ----------------------------------------------------------------------------------------------------
```

```
                4010      4020      4030      4040      4050      4060      4070      4080      4090      4100
          ....|....|....|....|....|....|....|....|....|....|....|....|....|....|....|....|....|....|....|....|
MZ218053  GAUGCUUAUAAAUUUAUAACAUCUACUCUGACCCCUGUUAAAAACCUACCUGAUUUUGAUGCCCUCGUGGGAAAGAUACUCAGCUUCCAGAUUCUUGUGC
SYMN012   GATGCTTGGAAATTTATTTCATCTACTATGTGTCCTATTAAAGCTTTGCCAGATTTTGATGCGCTCGTGGGAAAGATCCTCAGTTACCAAATTTTGACAC
SYMA008   GATGCCTGGAAATTTATTTCTTCCACTTTAAAACCAACCAAGAGTTTGCCTGATTTTGATGCGCTTGTGGGAAAGATCCTCAGTTACCAAATTTTAACAC
MEMN002   ----------------------------------------------------------------------------------------------------
SYMA003   GATGCTTGGAAGTTTATATCATCTACTATTAATCCAACTAAGCATTTTCCTGATTTTGATGCGCTCGTGGGAAAGATCCTCAGTTACCAAATTTTAACAC
SYMN007   GATGCGTGGAAATTTATTTCTTCCACGCTTAGTCCAACTAAGCAGTTGCCTGATTTTGATGCGCTTGTGGGAAAGATCCTCAGTTACCAAATTTTAACAC
SYMA006   GATGCTTGGAAGTTTATATCTGCTACTTTAAAGCCTACAAAAAGTTTACCTGATTTTGATGCGCTTGTGGGAAAGATCCTCAGTTACCAAATTTTAACAC
SYMN011   ----------------------------------------------------------------------------------------------------
SYMN010   GATGCTTGGAAATTTATTTCTTCAACTTTACATCCAACTAAGCAATTGCCTGACTTCGATGCGCTCGTGGGAAAAATCCTCAGTTACCAAATTTTAACTC
SYMA009   ----------------------------------------------------------------------------------------------------
SYMA010   GATGCTTGGAAGTTTATTTCATCTACTGTTAATCCAACCAAGCAATTGCCTGATTTTGATGCGCTTGTGGGAAAAATCCTCAGTTACCAAATTTTAACAC
MEMT006   GATGCATATAAGTTTATAACATCTACCTTGACACCTGTGAAAAATTTACCTGATTTTGATGCCCTTGTGGGAAAGATACTCAGCTTCCAGATTCTTGTAC
SYRL007   ----------------------------------------------------------------------------------------------------

                4110      4120      4130      4140      4150      4160      4170      4180      4190      4200
          ....|....|....|....|....|....|....|....|....|....|....|....|....|....|....|....|....|....|....|....|
MZ218053  ACAAUCUCCCGGAUGACGAUCCGGUCAAGAUCUGGUUCGAAGAGGCACAUGCUGCCCUUUCACUGCAUGCUUCCGCUAGUGGUGAGGAGCCAAUCCCCAC
SYMN012   ACAATCTACCGGATGACGATCCGGTTAAGAGATGGTTTGAGGATGCGCACTCCGCCCTTGTCATGCATAATCGTGTTAGTGGAGGGGATCCTCTCCCCGT
SYMA008   ATAATTTGCCGGATGACGATCCGGTTAAGAATTGGTTTGAGGCTGCGCATGCCGCCCTTGTCTTGCATAGTCGCGTTGATGGTGGAGATTCCCTCCCCAC
MEMN002   ----------------------------------------------------------------------------------------------------
SYMA003   ACAATTTGCCGGATGACGATCCGGTGAAGAATTGGTTTGAGGATGCGCACTCCGCCCTTGTCATGCATAATCGTGTTAGTGGAGGGGATCCTCTCCCTAT
SYMN007   ACAACCTTCCGGATGACGATCCGGTTAAGGATTGGTTTGAGGCTGCGCATGCCGCTCTAGTCATGCACAATCGCGTTGATGGTGGGGACCCTCTCCCTGT
SYMA006   ATAATTTGCCGGATGACGATCCGGTTAAGAATTGGTTTGAGGCTGCGCACTCCGCCCTTGTCTTGCATAATCGCGTTGATGGTGGGGATGTTCTCCCCAC
SYMN011   ----------------------------------------------------------------------------------------------------
SYMN010   ATAATCTTCCGGATGACGATCCGGTTAAGAATTGGTTTGAGGAAGCGCATGCCGCCCTTGTC--------------------------------------
SYMA009   ----------------------------------------------------------------------------------------------------
SYMA010   ACAATCTTCCGGATGACGATCCGGTTAAGAATTGGTTTGAGGAAGCGCATGCCGCTCTTGTCCTGCATAATCGCGTTGATGGTGGGGATCCTCTCCCCTC
MEMT006   ATAATTTGCCGGATGACGATCCGGTCAAGAACTGGTTTGAGGAGGCACATGCTGCCCTCTCATTGCATGCTGCCGCAGATGGTGGGGAGCCTATCCCCAT
SYRL007   ----------------------------------------------------------------------------------------------------

                4210      4220      4230      4240      4250      4260      4270      4280      4290      4300
          ....|....|....|....|....|....|....|....|....|....|....|....|....|....|....|....|....|....|....|....|
MZ218053  UAUUACGCGGGACAUGCGUGAGUUUCUUUGGAGGGGAGGACCAAAGAUAAAUGGCUGAUUCUGCUCCCAAACCAAAAAGACAGAGACGCGUUCGUCGCCA
SYMN012   CATTACTCGCGATATGCGTGATTTTCTTTGGAG-------------------------------------------------------------------
SYMA008   CATTACCCGTGACATGCGAGATTTTCTTTGGAG-------------------------------------------------------------------
MEMN002   ----------------------------------------------------------------------------------------------------
SYMA003   CATTACTCGCGATATGCGGGATTTTCTTTGGAGG------------------------------------------------------------------
SYMN007   CATTACCCGCGATATGCGTGATTTTCTTTGGAG-------------------------------------------------------------------
SYMA006   CATTACCCGCGATATGCGTGAYTTTCTTTGGAG-------------------------------------------------------------------
SYMN011   ----------------------------------------------------------------------------------------------------
SYMN010   ----------------------------------------------------------------------------------------------------
SYMA009   ----------------------------------------------------------------------------------------------------
SYMA010   CATCACTCGCGACATGAGGGATTTTCTTTGGA--------------------------------------------------------------------
MEMT006   TATCACGCGGGAGATGCGTGAGTTTCTTTGGAG-------------------------------------------------------------------
SYRL007   ----------------------------------------------------------------------------------------------------
```
