# Supplementary material for: A Novel Coronavirus and a Broad Range of Viruses in Kenyan Cave Bats
Source: Viruses. 2022 Dec 17;14(12):2820. doi: 10.3390/v14122820 (PMC9785147; doi:10.3390/v14122820)
Supplement: Supplementary file 1 [file viruses-14-02820-s001.zip › Supplementary Data S1.pdf]

Supplementary Data S1: Coronavirus sequences ontained in the study. The sequences are provided as FASTA files.

>SUOHC3001-ON893136
GGGCACTATACAGTGTTCGATGATGACACTGGACGTGTCCATGATGGCAGTGATTTTGCAC
CCGGAGATCTTTCCACCATGGCCGTTACTTCGCTCGTTGTGCGTAATCGTGCATTTTTGGACC
CGGTTGCTCGCGTCGAGCGTAAAGCTACTGAATTTGTGCAGACATTAGATGTAGCTTCTGAT
CAATTCTTTGCCTTTGGTGATGTTGTGTCACGGAATGTTATTACAGTGCTTTTGTGGCTTTTTA
GTATGCTTAGTATAGTTTTTAAGGCCTTTAAGAGTAAAGATTTAAAAGTTTTTGCTTTAGCCC
CTGAGAGAACTGGTGTTATACTAAGTCGTAGTTTACGCTATAATTTTAAAGCTTGTAAGCAC
GTTTTTAAGCTTAAGATGCGTTGGGTCACATTGTTTTTCAAATTTTTAGTTCTGCTGTACACAT
TGTATGCAGTAGGTTTCTTGTTTGTACGGTTTGGTCCTGTTAACGGTTATGTCTGTGGTGACT
ACGTTTTGGGTTATGCAGAATCAAACTTCGATAAGAATTCATATTGCAATTCTCCGCTGTGT
AAAGTGTGCTTGTATGGCTACCAGGAATTGAGTGACTTTCCTCATACTAACACTGTTTGGGA
GCATATTAAAGACCCACTTATTGGGAATGTTATGCCCTTGTTTTACTTAGTCTTTCTAGCAAT
TTTTGGTGGTCTTCCGATGAGAATTATTGTGTGCTATTTTGTTGCACAGTATGTAAACTTTTTT
GGTAGCAAATTTGGTGTTCAAGACAATGTTTGGCTTCTACAGGTCATTCCATTCTCAGTGTTT
GGTGATGAAATTGTGGTTTGTTTCATTGTTTTTAAGGCACTTTGCTTTTTGCGACATGTCCTCT
TAGGCTGTGATAAAGCTTCTTGTGTTGCTTGTTGTAAAAGTGCGCGTCTTCGTCGTGTACCTA
TGCAGACTATTGTGAATGGAGCTTCAAAATCATTTTATGTTATGGCTAATGGCGGTAAGAGT
TTTTGTAAGAAGCATAACTTCTTCTGTCTTAGTTGTGACTCTTATGGTCCTGGTAATACTTTTA
TTAATGAGGTAATTGCCAAGGAAGTGTCCAATGTGACTAAAACTAATGTTCAACCTACCGG
TACAGCTTGTGTAGACATTGATAAGGTTGAGTTTTCTAATGGGTTTTACTATTTGTATAGTGG
TGAGACATTTTGGAAATACAATTTTGACATTACTGAAGCTAAATATAGCTGTAAAGATGTCC
TTAAGAATTGTAACATTGTGTCTGATTTTATTGTTTACAATAATGTTGGTAGTAATGTGACAC
AGGTTCGTAATGCTTGTGTTTACTTATCTCAACTTTTGTGTAAACCTATTAAACTTGTAGACT
CAGCTCTCCTTGCCACGTTGAACGTAGACTTTAATGGTGCTCTGCATGCAGCTTTTGTGGAA
GTGCTTAATGACAGTTTTTCTAAGGACTTGTCGGGTTGCACTACTATGGCTGAGTGTAAGCA
GGCACTAGGTATTGATGTTAGTGATGATGATTTTGTTAATGCTGTTAGTAACGCACACCGTT
TTAACGTGTTGCTTACAGACGTTTCATTTAACAATTTTGTCACATCCTATGCCAAACCAGAG
GAGAAGTTGTCTACTCATGACCTTGCGACTTGTATGCGTGCTGGTGCTAAGGTTGTCAATCA
TAATGTTCTCGTAAAAGAGAATATGCCTGTTGTGTGGGCTGCTAGAGAATTTAACTTTTTAT
CTGAAGAATGTCGTAAGTATTTGATTAAGACGTCAAAAGCTAAGGGCGTCAATTTCATGCT
TACATTTAACGACAATAAGATGCAAACACACGTCCCCGTTGTTAGCATTATAGCTAAGCAA
GGGGCTGGTGTTTTGCCCACTTGTTACAAATTTTTGTGGTGGTTGTGTTTCTTTGTTGTTGTTG
TGTTCTTTAGTACTGGTTTATTGTCCTTTGAGCAACAGGTTACTAGTGAGACTGATTTTGACT
TTAAGTACATTGAGAATGGTGTGCTTAAAGTGTTCACAGAATCCTTGGATTGTGTGCATAAT
GTCTTTGACAATTTTGTTACATGGCATGACGCAAAATTCGGTTCGGTGCCAAGGGTTGATGC
CAGGTGTCCTATTGTCGTAGGTGTATCTGATGATGTGCGTATGATTCCTGGTATTGCCGCTGG
TGTTAAGCTTGTTGGTAAAACATTGGTTTTTGCACTTCAATCTGTGTTTGGTGATTCTGGTTTA
TGTTATGACCTTAATGGTTTGACAACCACTGCTAGTTGTGTTTTTAATTCGGCTTGTACTATT
CTTAGTGGCGTTGGAGGTAAACACACCTATTGTTACAAGGATGGTCTTGTGTCTGATTCAAA
GTTGTATTCTGACATCTTGCCTAATAGTCATTATAAACTTGAAGATGGCAATTATATTAAAC
TGCCAGAAGTTTTGACTAGGGGTTTTGGCTTCAGAACAATTCGCACCATTGAAACCACATAT
TGCCGTATGGGTCAGTGTATTGATTCTAGAGCTGGTGTGTGTTTTGGTGTTGACCGTTTCTTT
GTATACAGTGCTGAAACTGGTCCAGATTTTATCTGTGGTACAGGCCTTATTTCACTGTTGTAT
AATATCTTTAACATTTTTTCAGGTTCTTTCAGTGTGATGGCTTTGTCTGGTCAAATCATTTTTA
ATTTAGTTGTAGCAACTTTAGTGGTGTTTAGCTGTTTTATGGTTACCAAATTCAAGCGTGTCT
TTGGTGACCTTTCTTTTGCAGTTTTCACAGTTTGTACAACTTCTTGTGTGAATAGCATTTCTTA
CATTATTACACAGAATTATTGTGCTATGTATGTGTATGCTTTCTTGTATTTTATGGCCACTCG
CAATATGCGTTATGCTTGGATTTGGCATAGTGGCTTTGTGCTTGCCTATTTCACACTGGCACC

ATGGTGGCTTATTGTTTGGTATGTCTGTGCTACATGTGTTGGGTTGATTCCTAGTCTTATGAA
ACTTAAGGTTTCTACGCAGCTTTTTGAGGGAGACAAGTTTGTCGGCACTTTTGAGAGTGCTG
CTGCAGGTACTTTTGTGATTGACATGCATTCTTATCAGCGTCTGGTCAATAGTATTGCACCG
GAGAAGCTGAAACAGTATGCTGCCAGTTACAATCGTTATAAGTACTATAGTGGTAGCGCTA
GTGAGGCTGACTACCGTCTTGCTTGTTTCGCTCACCTTGCAAAGGCTATGTTGGACTATGGT
ACCAACCATCAAGACATGCTCTACACTCCTCCCACAGTTTCGTACAATTCTACATTGCAGTC
TGGTCTGCGAAAGATGGCTCAACCATCTGGTGTTGTTGAAAAATGTGTGGTTCGTGTGAGTT
ATGGTAACATGACATTGAATGGTGTTTGGCTTGGTGACACAGTTATTTGTCCTAGACATGTT
ATTGCGTCATCAACCACTTCTACCATTGATTATGAACATGAGGCTTCTATTATGCGTCTTCAT
AATTTTTCTATTGCTGTTGGTAATGTCTTTTTGGGTGTTGTAGGTGTTTCTGTTAGAGGTTGTA
ATCTTCATATTAAGGTTAACCAATCTAATGTTAACACACCCAATTATAGCTTTAAAACTTTG
AAGGCTGGTGATTCTTTCAACATTTTGGCTTGTTATGATGGTGTACCTGCTGGTGTCTATGGT
GTGACACTGCGCACAAATAACACTGTGCGTGGTTCATTTATCAATGGAGCTTGTGGTTCACC
GGGTTACAATATTGTTGGAGGTACAGTTGAGTTTTGTTACCTTCATCAGCTTGAACTTGGCA
GTGGTTGTCATGTTGGTTCTAATCTTGAAGGTGTTATGTATGGTGGTTTTGAGGATCAACCTA
CATTGCAAATTGAAGGCGCTAGTAATTTAGTTACTGTAAATGTTATTTCGTTTCTTTACGGTG
CACTTTTGAATGGTGTTACATGGTGGCTTACAAGTGAACGTGTCACCATAGACGCTTATAAT
GAGTGGGCTCAACAAAATGGTTTTACACAGGTTAGTTCTGTGGATTGTTTTACCATTCTTGC
AGCCAAGACTGGTGTAGACGTGCAACGTATTTTAGCGGCTGTACAGCGTTTGCACAAGGGT
TTTGGTGGTAAGGCTATCCTTGGATTCACTTCTCTTACAGATGAGTTCACTACTGCTGAGGTC
ATACAGCAAATGTATGGTGTCAGTCTACAGAGTGGCAAGGTTTCCAGTACTTTCAAGAATTT
GTTTCTTGTTGGTACTTTCTTTGTTATGCTGTGGTCTGAGTTGATGATGTTCACACCATTTTTCT
GGGTTAGTCCCGGATTTGTCACTCCAATTTTTGCTATGCTCGTTTTGCTGTCAATTATGTTTAC
AGGTTTTATTAAACATAAAGTTTTGTACCTTTATACATTTTTGATTCCAGCAGTTATTGTGAC
GTCTTGTTGCAATTTTCTTGTTGAGCTTGCAATTAATTCTTGGCTTGCATCTGTTCTTGATTAT
CACGCCTCTATTATGAGTGTTGATATTCAGGGTGTGATTAATGTAGTTATTTGTGCTATTGTG
ACTATATTGCACACGTACAGGTTTGCCAAGGCTGGTTTTGTGACTTATGTTACATATTTTCTG
TCGTTGTTTGTGGTATTTTATAATTGGTGGTTTCATGGTGATCTGCTGAGTCTGTTTATGATGT
TGCTTCTCAATGTGTCAGATAGCTGGTATGTTGGTGCTATTGCATACAGAATATCATTGCTTG
CAGCCACACAATTCCCTATGTTGGAACTTGTTGTTGGTAAGGCCAAGGTTGTGATTTTTATTT
ATGTTGCAATTGGTTTTTTGTGCTGTATGTATTATGGTGTTCTGTATTGGATTAACAGATTCTG
TAAGTTGACACTTGGTGTATATGACTTTAAAGTCAGTGCTAATGAGTTTAAGTATATGGTTG
CTAACGGGTTACGTGCTCCTAGAGGTGTGTTTGACTCAATGCTTCTGTCTATGAAACTTGTTG
GTATTGGTGGTGATAAAGTTATAAAAGTTTCCACGGTACAATCCAAACTGACAGATATCAA
GTGCACTAATGTTGTTTTGCTTGGCTGTTTGTCTAGTATGAACATTGCAGCTAATACTAAAGA
ATGGGCTCATTGTGTCGAATTGCACAATAAGATTAATCTTTGTGATGATCCTGAAAAGGCAC
AAGAAATGCTTCTTGCATTGCTAGCTTTCTTTATTAGTAAGCAAAAAGATTTTGGCATTGAT
GATTTGATTGATTCATACTTTGACAGTTCTAGTGTACTGCAGAGTGTTGCGTCTACATTCGTC
AATATGCCTTCTTACATTGCTTATGAAAGTGCGCGGCAAAGTTATGAGGATGCTGTTAATAA
TGGGTCCTCTCCACAGCTCATTAAGCAGCTTAAGCGTGCTATGAATATTGCTAAAGCTGAGT
TTGACCATGAGTCTTCGGTTCAAAAGAAGATTAATCGCATGGCCGAACAAGCTGCTGCTCA
GATGTTCAAGGAAGCACGTGCTGTTAATAGAAAGTCCAAGATTATTAGTGCTATGCACTCTT
TGCTTTTTAGCATGCTTCGTCGCTTGGACATGTCTAGTGTAGACACTATTCTTAGTCTTGCAA
AAGATGGTATAGTTCCTCTGTCGGTGATCCCCGCCACTTGTGCTACTAAACTTTCTATTGTTA
GCAGTGATTTTACGTCATTTGCTAAGATTGTTAAAGATGGTTGTGTGCACTATGCTGGTGTG
GTGTGGTCGATTGCTGAAATCACTGATGCTGATGGTAAGATCGTACATCTTAAGGAGGTTGT
CGAAGAGAACACAGATTCACTTACATGGCCACTCTTTGTTAGTTGCGTGCGTGTTGTGAAGC
TTCAGAATAATGAGATTATTCCTGGTAAGCTTAAACAGCGTGTTGTAAAGGCAGAGGGTGA
TGGTTTCAGTGTTGAAGGTAAAGCACTCTTTAATAATGAGGGTGGTAAAACTTTTATGTATG
CTTTTGTCGCTGACAAACCAGATTTGAAATATGTTAAGTGGGAATTTGATGGTGGTTGTAAC
ACTATTGAACTTGAGCCACCTTGTAGGTTTGCTGTTGAAACTGCAAATGGCACTGTTATAAA
GTACTTGTATTTTGTGCGTAATCTAAACACATTGCGCAGGGGTGCAGTTTTGGGCTTTATTGG

TGCTACAGTGCGTTTGCAAGCCGGTAAACAAACAGAACTTGCTTCTAATTCGGCATTGCTTA
CTATGTGTGCTTTTGCTGTTGATCCTGCAAAGACGTATGTTGACGCTGTTAAGAAGGGTGTT
AAACCTGTCGGGAATTGCGTCAAGATGTTGTCAAATGGTTCAGGTACTGGCCAAGCTATTA
CTGTTGGTGTTGAAGCTAATTCTAATCAAGATTCTTATGGTGGCGCTTCTGTCTGTTTGTATT
GCCGTGCACATGTTGAACATCCTACGATGGATGGTGCGTGTCGTTACAAAGGCAAGTATGT
CCAAGTCCCACTTGGTAATGCGGATCCTATACGGTTCTGTTTAGAAAATGAGCCATGTAAA
GTTTGTATGTGCTGGTTGAATAATGGTTGCACTTGTGATAGAACTTCTATTCAGAGTGTTGAT
ACGAGTTATTTAAACGAGCACGGGGCTCTAGTGCAGCTCGACTAGAGCCCTGTAATGGAAC
TGACACGGATCGTGTAGTCCGTGCTTTTGACATTTACAATAAGGATGTTGCGTGTATTGGCA
AGTTTCTTAAGGTCAATTGTGTCCGTTTTAAGAATCTTGACAAGCATGATGCCTTCTTTGTTG
TAAAACGTTGTACGAAGTCTGTCATGGAACACGAGCAGTCCATATATGACAAGCTTCACAG
TTGTGGTGCAGTTGCAGTTCATGATTTTTTCTTGTGGAAAGATGGCCGTTCAGTTTTTGGTAA
TATTAGCAGGCAAAACCTGACTAAGTATACAATGATGGACTTGTGTTATGCTTTGAGAAATT
TTGATGAGCGTAATTGTGAAACACTTAAAGAAATTCTTGTCCTTACTGGTTGTTGTGACTATT
CATATTTTGAAAATAAGGATTGGTACGACCCAGTTGAAAATGAGGACATTCATAAAGTTTA
TGCACACCTTGGAACTGTTGTCTCTAATGCATTGCTTAAGTGCGTTAAGCTTTGCGATGCGA
TGGTAGAGGCAGGTGTAGTGGGTGTGTTAACACTTGATAACCAAGACCTTAATGGTGATTTT
TATGATTTTGGTGACTTTGCTACGACAATACCTAAAATGGGCGTACCATTATGTACCTCTTA
TTACTCTTATATGATGCCTGTAATGGGTATGACCAATTGTCTTGCATCTGAGTGTTTTATTAA
GAGTGACATTTTTGGTAGTGATTTTAAGACCTATGATTTGCTTGAGTACGACTTTACAGAGC
ACAAAGAGGCTTTGTTTCGTAAGTACTTCAAGTATTGGGGTCAGGATTACCACCCTAATTGT
GCTGATTGTCACGACGAGCTTTGTATTGTTCATTGTGCGAATTTTAATACGTTGTTCGCCACG
ACTATTCCTAATACAGCTTTTGGTCCTCTGTGTCGCAAAGTGTTTGTTGATGGTGTGCCACTT
GTTACTACAGCTGGCTACCATTTCAAGCAGTTGGGCCTTGTTTGGAATAAGGATATAAATAC
TCACTCTACTCGTTTGAGTGTAAATGAGTTGTTACAGTTTGTCACAGACCCTGCACTTTTGAT
AGCATCATCACCAGCCCTTGTGGATCAGCGTACTGTTTGTTTTTCAGTCGCTGCCTTGAGTAC
TGGTATGACAAATCAGACAGTTAAGCCAGGCCACTTTAATAAAGAGTTCTACGACTTTTTG
AGATCGCAAGGATTTTTTGAAGAAGGCTCTGAATTAACTTTAAAGCATTTCTTCTTTGCACA
GAAGGGTGATGCTGCTGTCAAAGATTTTGACTACTATCGTTATAATAGACCAACTATGCTAG
ACATTTGTCAGGCTAGAGTCACTTATAAAATAGTGCAGCGTTATTTTGAGTGCTATGAGGGT
GGCTGTATTGCCGCTAAGGATGTTGTTGTTACTAACCTCAATAAAAGTGCAGGTTACCCACT
TAATAAATTTGGTAAAGCTGGTTTGTATTATGAGTCTCTTAGTTATGAGGAACAAGATGCAC
TATACGCTCTTACAAAGCGTAATGTGTTACCTACTATGACACAGCTTAACCTTAAATATGCA
ATCAGTGGTAAAGAACGTGCTAGGACTGTTGGTGGTGTTTCATTGTTGTCTACTATGACTAC
TAGACAGTTTCATCAGAAACACCTTAAGTCCATTGTTAATACACGTAATGCATCTGTTGTCA
TTGGTACCACAAAATTTTATGGTGGTTGGGACAACATGCTTAAGAACCTTATTGATGGTGTT
GACAATGCGTGCCTTATGGGTTGGGATTACCCTAAGTGTGATCGTGCACTCCCTAATATGAT
TCGCATGATTTCTGCCATGATTTTGGGTTCGAAGCATGTTACTTGCTGTGATTCTTCTGATAA
ATATTACCGTCTCTGTAATGAGCTCGCTCAAGTGTTGACAGAAGTTGTATATTCTAATGGTG
GGTTTTATCTCAAGCCTGGTGGTACCACTTCTGGCGATGCTACAACGGCTTATGCGAACTCT
GTGTTTAATATTTTTCAAGCTGTTAGCGCCAATATTAACAGGTTGCTTAGCATTGATAGCAA
TGTGTGTAATAATATTGGTGTTAAGAGTTTGCAGCGTAAGCTATATGATTGTTGTTACAGAT
CTTCTTCAGTAGATGATTCTTTTGTAGACAGTTATTATTGCTACTTGCGGAAACATTTTTCTAT
GATGATTTTGTCAGATGACGGTGTTGTGTGTTATAATAAAGATTATGCTGATTTGGGTTATGT
GGCAGATATTAATGCCTTTAAAGCTACCCTATACTATCAGAATAATGTCTTTATGAGTACTG
CTAAATGCTGGGTGGAGCCAGACATTAGCAAAGGTCCTCATGAGTTCTGTTCACAGCATAC
TATGCAGATTGTAGATTCTGACGGCAAGTATTATTTGCCATACCCAGACCCGTCTAGAATAT
TGTCTGCTGGTGTATTTGTCGATGATGTTGTTAAGACTGACGCTGTGATATTGTTGGAGCGCT
ACGTCTCATTGGCAATCGACGCATATCCTTTGTCTAAACATCATAATCCTGAATATAGAAAA
GTGTTTTATACACTTCTAGATTGGGTTAAGCATCTACACAACACACTTAATCAGGGCGTGCT
TGAGTCTTTCTCCGTTACATTGCTTGAGGATGTATCCTCTAAGTTTTGGGATGAGAGTTTTTA
TGCTAGTTTGTATGAAAAGTCTACAGCACTTCAAGCCGCAGGCCTATGTGTTGTTTGTGGTT

CTCAAACTGTACTGCGTTGTGGTGATTGTCTTAGACGACCTATGTTGTGCACTAAGTGTGCTT
ACGACCATGTTGTCACCACGAATCATAAATTCATTCTGGCTATAACACCTTACGTTTGTAAT
GCTTCTGGTTGCACCGTTAATGATGTGACTAAGTTGTATTTAGGAGGTCTGAGTTACTGGTGT
ATAGACCATAAACCTACACTGTCACTACCACTTTGTAGTGCAGGTAATGTTTTTGGTTTGTA
TAAAAACTCTGCTACAGGTTCACCGGATGTAGAGGTTTTTAATACACTTGCCACGTCAGATT
GGACTGACGTTAAAGATTACAAGCTGGCTAATGAATGCAAGGATTCTCTTAGGCTTTTTGCG
GCTGAGACTGTTAAAGCCAAAGAAGAGAGTGTTAAATCCTCTTATGCTTGTGCTACACTTA
AAGAAGTTGTTGGGCCACGTGAGTTGATTCTTAGTTGGGAGTGCGGAAAAATTAAACCGCC
ACTTAACCGTAATTCAGTGTTTACGTGTTTCCAGATTTCTAAAGACTCTAAGTTCCAGGTTGG
AGAGTTTACGTTTGAAAAATTGGATTACGGTTCTGATGCTGTGTGTTACAAGTCTACCACCA
CAGTTAAATTAGTGCCTGGTATGATTTTTGTTCTCACATCACACAATGTACAGCCGCTTAGG
GCTCCCACTATTGCTAATCAAGAACGCTATGCTTCAATTTATAAACTGCACCCTACTTTTAA
TATTAGTGATGCTTATGCCAATTTGGTGCCGTATTACCAAATGATTGGCAGACAACGAATCA
CAACTATTCAAGGCCCACCGGGAAGTGGCAAGTCCCATTGTGTTATAGGTCTGGGTCTTTAT
TACCCAGGTGCTAGGATGGTCTTTGCGGCTTGCTCTCATGCAGCCGTAGATTCACTTTGTGTC
AAGGCTGCCACTGCTTATAGTGTAGACAAATGCACTCGTATTATCCCTGCCCGTGCTAGGGT
TGAGTGTTATAGTGGGTTTAAGGTAAATAACACTCAGGCACAGTACATCTTTTCAACAGTCA
ACGCACTCCCTGAGTGTAATGTTGACATAGTTGTTATAGATGAAGTGTCTATGTGTACTAAT
TATGACCTGTCCGTTATTAACCAGCGGTTGTCTTATAAACATATTGTTTATGTGGGTGACCCA
CAGCAATTACCAGCGCCACGTACCATGATCACTAAAGGCGTTCTCGAACCTAAGGACTATA
ATGTTGTAACTCAGAGGATGTGTGCTGTTGGGCCAGATGTCTTCTTGCATAAATGTTACAGA
TGTCCTGCTGAGATTGTTAAAACAGTCTCAGAGTTGGTTTATGAGAATAAGTTTGTACCTGT
TCATGCTGATAGCAAACAGTGTTTTAAACTGTTTTGTAAGGGTAATGTTCAGGTTGACAATG
GTTCTAGTATTAACAGAAAGCAATTGGATATCGTCAAGATGTTCCTTGCTAAGAATCCACGT
TGGTCTAAAGCTGTGTTTATCTCACCTTATAATAGTCAGAATTATGTGGCTAGTAGGGTTTTG
GGTTTGCAGATTCAGACTGTTGATTCCTCTCAAGGTAGTGAATATGACTATGTCATATACAC
ACAGACGTCAGACACAGCACATGCTTGCAATGTTAATAGATTTAATGTTGCAATTACAAGA
GCCAAGAAGGGTATTCTTTGTGTTATGTGTGATCGGGAGTTGTTTGATGCTCTGAAATTTTTC
GAGATCAAGCTTTCTGACTTGCAGGCTAGTGGTGATGGCTGTGGTCTTTTTAAAGATTGTTA
CAGGAAAGATGATGAATTACCTCCATCTCATGCAGCTACCTATATGGCCTTGTCTGACAACT
TTAAGGTCAATGGTGATCTTGCTGTGCAGATTGGCTTTAAAGGTGTGTGTAAGTATGAGCAT
GTTATTTCTTTTATGGGCTTCAGGTTTGATATTAATGTGCCTGAACAGCATAGTCTTTTCTGTA
CCAGGAATTTTGCAATGCGCCACGTGCGTGGTTGGCTTGGTATGGATGTAGAAGGTGCCCA
CGTTACAGGTGAAAATGTAGGTACTAATGTACCATTACAAGTAGGCTTCTCTAATGGTGTTG
ATTTTGTTGTCCAACCGGAAGGTTGTGTTGTCACTGAGACAGGCAGCAGGATCGTACCTGTT
AGAGCTCGTGCTCCGCCAGGTGAACAGTTCACACATTTGGTCCCCCTGCTTCGTAAGGGAC
AACCATGGGCTGTGGTTCGTAAACGCATTGTTCAGATGTGTTGCGATTACTTGGTGAACCTA
TCAGACATATTAATCTTTGTTTTGTGGGCTGGTGGTTTGGAGTTGACTACCATGAGATATTTT
GTTAAAATTGGACCAAGCAAAGTTTGTGAGTGCGGCAAGGTGGCCACTTGTTACAATAGTG
TGACTCATGCATACTATTGTTTCAAGCATGCCTTAGGGTGTGATTATTTGTACAATCCCTATG
TTATTGACATTCAGCAGTGGGATTATACGGGTTCACTTAGCCATAATCATCATGAGCATTGT
AATGTACATCGCAATGAGCATGTTGCATCTGGTGATGCTATTATGACTCGTTGTCTTGCTGTG
TATGATTGCTTTGTCAAGAACGTTGATTGGTCCATTACTTACCCATTCATTGCTAATGAACAT
GCCATTAATAAGAGTGGTAGAGTTGTGCAATCTATGGTTGTCAAAACAGCTATAAAGTTAT
ATAATCCTAAAGTAATTCATGATATTGGTAATCCTAAAGGTATTAGGTGTGCAGTTACTGAT
GCACACTGGTTTTGCTATGATAAGCAGCCCCTCAATTCCAATGTCAAGTTGCTTGAGTATGA
TTATATAACTCATGGTCAAATGGATGGGTTATGCCTATTTTGGAATTGCAATGTTGACATGT
ATCCTGAATTTTCTATTGTGTGCCGTTTTGACACTCGTTGTAGGTCAAATCTCAACTTAGAAG
GTGTTAACGGAGGTTCACTTTATGTCAACAATCATGCATTTTTTACACCTGCATTTGATAAG
CGTGCAATGGCTAAACTTAAGCCTATGCCCTTCTTTTTCTATGATGACACGCCTTGTGATCGT
GTTCATGATCAGATAAATTATGTACCACTGCGTGCTAGTACTTGTATTACAAAGTGTAATAT
TGGTGGTGCTGTTTGCAGTAAGCATGCCAATATGTTTCATGCCTATGTTAACGCATATAATA

CTTTCACGCAGGCTGGTTTTACTATTTGGGTTCCTGTAACTTTTGATTTGTTTAACCTTTGGCA
AACATTTGTCGAAACTAACTTGCAAGGCTTGGAGAACATAGCGTTTAATGTCCTCAAGAAA
GGGTCTTTTGTTGGTGTAGAGGGTGAACTACCGGTTGCTATCGTTAATGACCGTGTGCTGGT
ACGTGATGGCACCGTGGATAATTTGATTTTTACTAATAAAACCTCATTACCTACTAATGTTG
CTTTCGAGCTTTACGCCAAACGTAAAATGGGTTTGACACCACCTCTTGCTGTTCTCAGGAAT
CTTGGTGTTGTTAGTACCTATAAGTTTGTGTTATGGGATTATGAGGCAGAAAGACCATTTAC
TACTTTTACTAAAAATGTTTGCAGTTACACTGATTTTGATGAGGATGTGTGCACTTGCTTTGA
TAATAGCATTCAGGGTTCGTTTGAACGCTTTTCAATGGCTAACAATGCAGTGCTTATTTCCA
CATCTGCTGTTAAGAAGCTTACTTGTATTAAGCTTAACTATGGTTTGCTTAATGGTGTGGCTG
TTTCTAAGACTGGTGACAAGCCTGTTACATGGTATATCTATGTCCGTAAAAATGGTCAATTT
GTTGACCAATTTGACGGCTATTTTACTCAGGGGCGTACTACTATTGATTTCTTACCTCGTAGT
GTTATGGAGGAAGACTTTTTAAACCTTGATATGGGTTTGTTTATTAGTAAGTACGGTCTTGA
GGACTATGCTTTTGAGCATGTCGTGTATGGTGACGTGTCTGGTACTACACTTGGTGGATTAC
ATTTATTAATTTCTCAAGTACGCCTTGGAAAAATGGGTGTCTTGAAAGTGGATGAGTTCACA
CATAGTGAGGATAGTACATTGAAGTCCTGTACAGTCACCTATTCTAATGATCCTAGTAGTAA
AATGGTTTGTACCTATATGGATTTGTTGCTAGATGATTTTGTTACCATTTTAAAATCTTTGGA
TTTGGGTGTAATTTCTAGAGTTCATGAAGTTGTAGTCGATTGTAAGGTTTGGCGTTGGATGTT
GTGGTGTAAGGACCACAAAGTTCAAACGTTCTACCCACAGTTGCAGTCGTCAGAATGGAAA
TGTGGTTATTCCATGCCCTCACTGTATAAAATACAGCGTATGTGTCTTGAGCCCTGTAATTTG
TACAATTATGGTGCTAGTCTTAAGCTACCTGAAGGTATCATGTTTAACGTGGTCAAATACAC
TCAGTTATGTCAATATTTGAATAGTACTACTATGTGTGTACCGCATAATATGCGTGTACTAC
ATCTTGGTGCTGGCTCAGATAAAGGAGTCGCACCTGGTACTGCTGTATTGCGTCGTTGGTTA
CCCAGTGATGCTATTATTGTTGACAATGATGTTAATGACTATGTTAGTGATGCAGATTTCAG
TGTAACAGGGGATTGCGCCACTTTGTACTTACAGGATAAGTTCGACTTAGTGGTTTCAGACA
TGTATGATGGCCGTATTAAACAGTGTGATGGTGTTAACGTTTCAAAGGATGGCTTCTTTCCTT
ACATCAATGGTGTGATATCAGAGAAGCTTTCCCTAGGTGGTACAGTTGCCATTAAGATTACT
GAGTATAGCTGGAATAAGCGGCTGTATGAGTTGATACAAAAGTTTGAATTTTGGACGTTGTT
TTGTACTAGTGTGAATACATCATCGTCTGAAGCTTTTCTAATTGGTGTTAACTATCTTGGCGA
CTACGCTACTCAACCCATTATGGATGGTAATACTATGCACGCTAATTATGTGTTTTGGCGTA
ATTCTACAATTATGTCCATGTCATATAATAGTGTGCTTGATCTAGCAAAGTTTAATTGTAGA
CATAAAGCTACGGTTGTTATTTCACTTAAAGAAAGTGATTTGAATGATGTTGTGGTAGGCCT
TATAAAGAATGGAAAGTTGTTGGTTAGAAAGAGTGGTGTCATTTGTGACTATGGTAACCAT
TTAGTTTCAACTAAATAA
>SUOHC3002
AGTGTTGATACGAGTTATTTAAACGAGCACGGGGCTCTAGTGCAGCTCGACTAGAGCCCTG
TAATGGAACTGACACGGATCGTGTAGTCCGTGCTTTTGACATTTACAATAAGGATGTTGCGT
GTATTGGCAAGTTTCTTAAGGTCAATTGTGTCCGTTTTAAGAATCTTGACAAGCATGATGCC
TTCTTTGTTGTAAAACGTTGTACGAAGTCTGTCATGGAACACGAGCAGTCCATATATGACAA
GCTTCACAGTTGTGGTGCAGTTGCAGTTCATGATTTTTTCTTGTGGAAAGATGGCCGTTCAGT
TTTTGGTAATATTAGCAGGCAAAACCTGACTAAGTATACAATGATGGACTTGTGTTATGCTT
TGAGAAATTTTGATGAGCGTAATTGTGAAACACTTAAAGAAATTCTTGTCCTTACTGGTTGC
TGTGACTATTCATATTTTGAAAATAAGGATTGGTACGACCCAGTTGAAAATGAGGACATTC
ATAAAGTTTATGCACACCTTGGAACTGTTGTCTCTAATGCATTGCTTAAGTGCGTTAAGCTTT
GCGATGCGATGGTAGAGGCAGGTGTAGTGGGTGTGTTAACACTTGATAACCAAGACCTTAA
TGGTGATTTTTATGATTTTGGTGACTTTGCTACAACAATACCTAAAATGGGTGTACCATTATG
TACCTCTTATTACTCTTATATGATGCCTGTAATGGGTATGACCAATTGTCTTGCATCTGAGTG
TTTTATTAAGAGTGACATTTTTGGTAGTGATTTTAAGACCTATGATTTGCTTGAGTACGACTT
TACAGAGCACAAAGAGGCTTTGTTTCGTAAGTACTTCAAGTATTGGGGTCAGGATTACCAC
CCTAATTGTGCTGATTGTCACGACGAGCTTTGTATTGTTCATTGTGCGAATTTTAATACGTTG
TTCGCTACGACTATTCCTAATACGGCTTTTGGTCCTCTGTGTCGCAAAGTGTTTGTTGATGGT
GTGCCACTTGTTACTACAGCTGGCTACCATTTCAAGCAGTTGGGCCTTGTTTGGAATAAGGA
TATAAATACTCACTCTACTCGTTTGAGTGTAAATGAGTTGTTACAGTTTGTCACAGACCCTG

CACTTTTGATAGCATCATCACCAGCCCTTGTGGATCAGCGTACTGTTTGTTTTTCAGTCGCTG
CCTTGAGTACTGGTATGACAAATCAGACAGTTAAGCCAGGCCACTTTAATAAAGAGTTCTA
CGACTTTTTGAGATCGCAAGGATTTTTTGAAGAAGGCTCTGAATTAACTTTAAAGCATTTCT
TCTTTGCACAGAAGGGTGATGCTGCTGTCAAAGATTTTGACTACTATCGTTATAATAGACCA
ACTATGCTAGACATTTGTCAGGCTAGAGTCACTTATAAAATAGTGCAGCGTTATTTTGAGTG
CTATGAGGGTGGCTGTATTGCCGCTAAGGATGTTGTTGTTACTAACCTCAATAAAAGTGCAG
GTTACCCACTTAATAAATTTGGTAAAGCTGGTTTGTATTATGAGTCTCTTAGTTATGAGGAA
CAAGATGCACTATACGCTCTTACAAAGCGTAATGTGTTACCTACTATGACACAGCTTAACCT
TAAATATGCAATCAGTGGTAAAGAACGTGCTAGGACTGTTGGTGGTGTTTCATTGTTGTCTA
CTATGACTACTAGACAGTTTCATCAGAAACACCTTAAGTCCATTGTTAATACACGTAATGCA
TCTGTTGTCATTGGTACCACAAAATTTTATGGTGGTTGGGACAACATGCTTAAGAACCTTAT
TGATGGTGTTGACAATGCGTGCCTTATGGGTTGGGATTACCCTAAGTGTGATCGTGCACTTC
CTAATATGATTCGCATGATTTCTGCCATGATTTTGGGTTCGAAGCATGTTACTTGCTGTGATT
CTTCTGATAAATATTACCGTCTCTGTAATGAGCTCGCTCAAGTGTTGACAGAAGTTGTATAT
TCTAATGGTGGGTTTTATCTCAAGCCTGGTGGTACCACTTCTGGCGATGCTACAACGGCTTA
TGCGAACTCTGTGTTTAATATTTTTCAAGCTGTTAGCGCCAATATTAACAGGTTGCTTAGCAT
TGATAGCAATGTGTGTAATAATATTGGTGTTAAGAGTTTGCAGCGTAAGCTATATGATTGTT
GTTATAGATCTTCTTCAGTAGATGATTCTTTTGTAGACAGTTATTATTGCTACTTGCGGAAAC
ATTTTTCTATGATGATTTTGTCAGATGACGGTGTTGTGTGTTATAATAAAGATTATGCTGATT
TGGGTTATGTGGCAGATATTAATGCCTTTAAAGCTACCCTATATTATCAGAATAATGTCTTT
ATGAGTACTGCTAAATGCTGGGTGGAGCCAGACATTAGCAAAGGTCCTCATGAGTTCTGTT
CACAGCATACTATGCAGATTGTAGATTCTGACGGCAAGTATTATTTGCCATACCCAGACCC
ATCTAGAATATTGTCTGCTGGTGTATTTGTCGATGATGTTGTTAAGACTGACGCTGTGATATT
GTTGGAGCGCTACGTCTCATTGGCAATCGACGCATATCCTTTGTCTAAACATCATAATCCTG
AATATAGAAAAGTGTTTTATACGCTTCTAGATTGGGTTAAGCATCTACACAACACACTTAAT
CAGGGCGTGCTTGAGTCTTTCTCCGTTACATTGCTTGAGGATGTATCCTCTAAGTTTTGGGAT
GAGAGTTTTTATGCTAGTTTGTATGAAAAGTCTACAGCACTTCAAGCCGCAGGCCTATGTGT
TGTTTGTGGTTCTCAAACTGTACTGCGTTGTGGTGATTGTCTTAGACGACCTATGTTGTGCAC
TAAGTGTGCGTATGACCATGTTGTCACCACGAATCATAAATTCATTCTGGCTATAACACCTT
ACGTTTGTAATGCTTCTGGTTGCACCGTTAATGATGTGACTAAGTTGTATTTAGGAGGTCTG
AGTTACTGGTGTATAGACCATAAACCTACGCTGTCACTGCCACTTTGTAGTGCAGGTAATGT
TTTTGGTTTGTATAAAAACTCTGCTACTGGTTCACCGGATGTAGAGGTTTTTAATACACTTGC
CACGTCAGATTGGACTGACGTTAAAGATTACAAGCTGGCTAATGAATGCAAGGATTCTCTT
AGGCTTTTTGCGGCTGAGACTGTTAAAGCCAAAGAAGAGAGTGTTAAATCCTCTTATGCTTG
TGCTACACTTAAAGAAGTTGTTGGGCCACGTGAGTTGATTCTTAGCTGGGAGTGCGGAAAA
ATTAAACCGCCACTTAACCGTAATTCAGTGTTTACGTGTTTCCAGATTTCTAAAGACTCTAA
GTTCCAGGTTGGAGAGTTTACGTTTGAAAAATTGGATTACGGTTCTGATGCTGTGTGCTACA
AGTCTACCACCACAGTCAAATTAGTGCCTGGTATGATTTTTGTTCTCACATCACACAATGTA
CAGCCGCTTAGGGCTCCCACTATTGCTAATCAAGAACGCTATGCTTCAATTTATAAACTGCA
CCCTACTTTTAATATTAGTGATGCTTATGCCAATTTGGTGCCGTACTACCAAATGATTGGCA
GACAACGAATCACTACTATTCAAGGCCCACCGGGAAGTGGCAAGTCTCATTGTGTTATAGG
TCTGGGTCTTTATTACCCAGGTGCTAGGATGGTTTTTGCGGCTTGCTCTCATGCAGCCGTAGA
TTCACTTTGTGTCAAGGCTGCCACTGCTTATAGTGTAGACAAATGCACTCGTATTATCCCTG
CCCGTGCTAGGGTTGAGTGTTATAGTGGGTTTAAGGTAAATAACACTCAGGCACAGTACAT
CTTTTCAACAGTCAACGCACTCCCTGAGTGTAATGTTGACATAGTTGTTATAGATGAAGTGT
CTATGTGTACTAATTATGACCTGTCCGTTATTAATCAGCGGTTGTCTTATAAACATATTGTTT
ATGTGGGTGACCCACAGCAATTACCAGCGCCACGTACCATGATCACTAAAGGCGTTCTCGA
ACCTAAGGACTATAATGTTGTAACTCAGAGGATGTGTGCTGTTGGGCCAGATGTCTTCTTGC
ATAAATGTTACAGATGTCCTGCGGAGATTGTTAAAACAGTCTCAGAGTTGGTTTATGAGAA
TAAGTTTGTACCTGTTCATGCTGATAGCAAACAGTGCTTTAAACTGTTTTGTAAGGGTAATG
TTCAGGTTGACAATGGTTCTAGTATTAACAGAAAGCAATTGGATATCGTTAAGATGTTCCTT
GCTAAGAATCCACGTTGGTCTAAAGCTGTGTTTATCTCACCTTATAATAGTCAAAATTATGT

GGCTAGTAGGGTTTTGGGTTTGCAGATTCAGACTGTTGATTCCTCTCAAGGTAGTGAATATG
ACTATGTCATATACACACAGACGTCAGACACAGCACATGCTTGCAATGTTAATAGATTTAA
TGTTGCAATTACAAGAGCCAAGAAGGGTATTCTTTGTGTTATGTGTGATCGGGATTTGTTTG
ATGCTCTGAAATTTTTCGAGATCAAGCTTTCTGACTTGCAGGCTAGTGGTGATGGCTGTGGT
CTTTTTAAAGATTGTTACAGGAAAGATGATGAATTACCTCCATCTCATGCAGCTACCTATAT
GGCCTTGTCTGACAACTTTAAGGTCAATGGTGATCTTGCTGTGCAGATTGGCTTTAAAGGTG
TGTGTAAGTATGAGCATGTTATTTCTTTTATGGGCTTCAGGTTTGACATTAATGTGCCTGAAC
AGCATAGTCTTTTCTGTACCAGGAATTTTGCAATGCGCCATGTGCGTGGTTGGCTTGGTATG
GATGTAGAAGGTGCCCATGTTACAGGTGAAAATGTAGGTACTAATGTACCATTACAAGTAG
GCTTCTCTAATGGTGTTGATTTTGTTGTCCAACCAGAAGGTTGTGTTGTCACTGAGACAGGC
AGCAGGATCGTACCTGTTAGAGCTCGTGCTCCGCCAGGTGAACAGTTCACACATTTGGTCC
CCCTGCTTCGTAAGGGACAACCATGGGCTGTGGTTCGTAAACGCATTGTTCAGATGTGTTGC
GATTACTTGGTGAACCTATCAGACATATTAATCTTTGTTTTGTGGGCTGGTGGTTTGGAGTTG
ACTACCATGAGATATTTTGTTAAAATTGGACCAATCAAAGTTTGTGAGTGCGGCAAAGTGG
CCACTTGTTACAATAGTGTGACTCATGCATACTATTGTTTCAAGCATGCCTTAGGGTGTGATT
ATTTGTACAATCCTTATGTTATTGACATTCAGCAGTGGGATTATACAGGTTCACTTAGCCAT
AATCATCATGAGCATTGTAATGTACATCGCAATGAGCATGTTGCATCAGGTGATGCTATTAT
GACTCGTTGTCTTGCTGTGTATGATTGCTTCGTCAAGAACGTTGATTGGTCCATTACTTACCC
ATTCATTGCTAATGAACATGCCATTAATAAGAGTGGTAGAGTTGTGCAATCTATGGTTGTCA
AATCAGCTATAAAGTTATATAATCCTAAAGTAATTCATGATATTGGTAATCCTAAAGGTATT
AGGTGTGCAGTTACTGATGCACACTGGTTTTGCTATGATAAGCAGCCCCTCAATTCCAATGT
CAAGTTGCTTGAGTATGATTATATAACTCATGGTCAGATGGATGGGTTATGCCTATTTTGGA
ATTGCAATGTTGACATGTATCCTGAATTTTCTATTGTGTGCCGTTTTGACACTCGTTGTAGGT
CAAATCTCAACTTAGAAGGTGTTAATGGAGGTTCACTTTATGTCAACAACCATGCATTTTTT
ACACCTGCATTTGATAAGCGTGCAATGGCTAAACTTAAGCCTATGCCCTTCTTTTTCTATGAT
GACACGCCTTGCGATCGTGTTCATGATCAAATAAATTATGTACCACTGCGTGCTAGTACTTG
TATTACAAAGTGTAATATTGGTGGTGCTGTTTGCAGTAAGCATGCCAATATGTTTCATGCCT
ATGTTAATGCATATAATACTTTCACGCAGGCTGGTTTTACTATTTGGGTTCCTGTAACTTTTG
ATTTGTTCAACCTTTGGCAAACATTTGTCGAAACTAATTTGCAAGGCTTGGAGAACATAGCG
TTTAATGTCCTCAAGAAAGGGTCTTTTGTTGGTGTAGAGGGTGAACTACCGGTTGCTATCGT
TAATGACCGTGTGCTGGTACGTGATGGCACCGTGGATAATTTGATTTTTACTAATAAAACCT
CATTACCTACTAATGTTGCTTTTGAGCTTTACGCCAAACGTAAAATGGGTTTGACACCACCT
CTTGCTGTTCTCAGGAATCTCGGTGTTGTTAGTACCTATAAGTTTGTGTTATGGGATTATGAG
GCAGAAAGACCATTTACTACTTTTACTAAAAATGTTTGCAGTTACACAGACTTTGATGAGG
ATGTGTGCACTTGCTTTGATAATAGCATTCAGGGTTCGTTTGAACGCTTTTCAATGGCAAAC
AATGCAGTGCTTATTTCCACATCTGCTGTTAAGAAGCTTACTTGTATTAAGCTTAACTATGGT
TTGCTTAATGGTGTGGCTGTTTCTAAGACTGGTGACAAGCCTGTTACATGGTATATCTATGTC
CGTAAAAATGGTCAATTTGTTGACCAATTTGACGGATATTTTACTCAGGGGCGTACTACTAT
TGATTTCTTACCTCGTAGTGTTATGGAGGAAGACTTTTTAAACCTTGATATGGGTTTGTTTAT
TAGTAAGTATGGTCTTGAAGACTATGCTTTTGAGCATGTTGTGTATGGTGACGTGTCTGGTA
CTACACTTGGTGGACTACATTTATTAATTTCTCAAGTACGCCTTGGAAAAATGGGTGTCTTG
AAAGTGGATGAGTTCACACATAGTGAGGATAGTACATTGAAGTCCTGTACAGTCACCTATT
CTAATGATCCTAGTAGTAAAATGGTTTGTACCTATATGGATTTGTTGCTAGATGATTTTGTTA
CCATTTTAAAATCTTTGGATTTGGGTGTAATTTCCAGAGTTCATGAAGTTGTAGTCGATTGTA
AGGTTTGGCGTTGGATGTTGTGGTGTAAGGACCACAAAGTTCAAACGTTCTACCCACAGTT
GCAGTCGTCAGAATGGAAATGTGGCTATTCCATGCCCTCACTGTATAAAATACAGCGTATG
TGTCTTGAGCCCTGTAATTTGTACAATTATGGTGCTAGTCTTAAGCTACCTGAAGGTATCAT
GTTTAACGTGGTCAAATACACTCAGTTATGTCAATATTTGAATAGTACTACTATGTGTGTAC
CGCATAATATGCGTGTACTACATCTTGGTGCTGGCTCAGATAAAGGAGTCGCACCTGGTAC
TGCTGTATTGCGTCGTTGGTTACCCAGTGATGCTATTATTGTTGACAATGATGTTAATGACTA
TGTTAGTGATGCAGATTTCAGTGTAACAGGGGATTGCGCCACTTTGTACTTACAGGATAAGT
TCGACTTAGTGGTTTCAGACATGTATGATGGCCGTATTAAACAGTGTGATGGTGTTAACGTT

TCAAAGGATGGCTTCTTTCCTTACATCAATGGTGTGATATCAGAGAAGCTTTCCCTAGGTGG
TACAGTTGCCATTAAGATTACTGAGTATAGCTGGAATAAGCGGCTGTATGAGTTGATACAA
AAGTTTGAATTTTGGACGTTGTTTTGTACTAGTGTGAATACATCATCGTCTGAAGCTTTTCTA
ATTGGTGTCAACTATCTTGGCGACTACGCCACTCAACTCATTGTGGATGGTAATACTATGCA
CGCTAATTATGTGTTTTGGCGTAATTCTACAATTATGTCCATGTCATATAATAGTGTGCTTGA
TCTAGCAAAGTTTAATTGTAGACATAAAGCTACGGTTGTTATTTCACTTAAAGAAAGTGATT
TGAATGATGTTGTGGTAGGCCTTATAAAGAATGGAAAGTTGTTGGTTAGAAAGAGTGGTGT
CATTTGTGACTATGGTAACCATTTAGTTTCAACTAAATAA
>SUOHC3004
GTGACCTTTCTTTTGCAGTTTTCACAGTTTGTACAACTTCTTGTGTGAATAGCATTTCTTACAT
CATTACACAGAATTATTGTGCTATGTATGTATATGCTTTCTTGTATTTTATGGCCACTCGCAA
TATGCGTTATGCTTGGATTTGGCATAGTGGCTTTGTGCTTGCCTATTTCACACTGGCACCATG
GTGGCTTATTGTTTGGTATGTCTGTGCTACATGTGTTGGGTTGATTCCTAGTCTTATGAAACTT
AAGGTTTCTACGCAGCTTTTTGAGGGAGACAAGTTTGTCGGCACTTTTGAGAGTGCTGCTGC
AGGTACTTTTGTGATTGACATGCATTCTTATCAGCGTCTGGTCAATAGTATTGCACCGGAGA
AGCTGAAACAGTATGCTGCCAGTTACAATCGTTATAAGTACTATAGTGGTAGTGCTAGTGA
GGCTGACTACCGTCTTGCTTGTTTCGCTCACCTTGCAAAGGCTATGTTGGACTATGGTACCA
ACCATCAAGACATGCTCTACACTCCTCCCACAGTTTCGTACAATTCTACATTGCAGTCTGGT
CTGCGAAAGATGGCTCAACCATCTGGTGTTGTTGAAAAATGTGTGGTTCGTGTGAGTTATGG
TAACATGACATTGAATGGTGTTTGGCTTGGTGACACAGTTATTTGTCCTAGACATGTTATTGC
GTCATCAACCACTTCTACCATTGATTATGAACATGAGGCTTCTATTATGCGTCTTCATAATTT
TTCTATTGCTGTTGGTAATGTCTTTTTGGGTGTTGTAGGTGTTTCTGTTAGAGGTTGTAATCTT
CATATTAAGGTTAACCAATCTAATGTTAACACACCCAATTATAGCTTTAAAACTTTGAAGGC
TGGTGATTCTTTCAACATTTTGGCTTGTTATGATGGTGTACCTGCTGGTGTTTATGGTGTGAC
ACTGCGCACAAATAACACTGTGCGTGGTTCATTTATCAATGGAGCTTGTGGTTCACCGGGTT
ACAATATTGTTGGAGGTATAGTTGAGTTTTGTTACCTTCATCAGCTTGAACTTGGCAGTGGTT
GTCATGTTGGTTCTAATCTTGAAGGTGTTATGTATGGTGGTTTTGAGGATCAACCTACATTGC
AAATTGAAGGCGCTAGTAATTTAGTTACTGTAAATGTTATTTCGTTTCTTTACGGTGCACTTT
TGAATGGTGTTACATGGTGGCTTACAAGTGAACGTGTCACCATAGATGCTTATAATGAGTG
GGCTCAACAAAATGGTTTTACACAGGTTAGTTCTGTGGATTGTTTTACCATTCTTGCAGCCA
AGACTGGTGTAGACGTACAACGTATTTTAGCGGCTGTACAGCGTTTGCACAAGGGTTTTGGT
GGTAAGGCTATCCTTGGATTCACTTCTCTTACAGATGAGTTCACCACTGCTGAGGTCATACA
GCAAATGTATGGTGTCAGTCTACAGAGTGGCAAGGTTTCCAGTACTTTCAAGAATTTGTTTC
TTGTTGGTACTTTCTTTGTTATGCTGTGGTCTGAGTTGATGATGTTCACACCATTTTTCTGGGT
TAGTCCCGGATTTGTCACTCCAATTTTTGCTATGCTCGTTTTGCTGTCAATTATGTTTACAGGT
TTTATTAAACATAAAGTTTTGTACCTTTATACATTTTTGATTCCAGCAGTTATTGTGACGTCTT
GTTGCAATTTTCTTGTTGAGCTTGCAATTAATTCTTGGCTTGCATCTGTTCTTGATTATCATGC
CTCTATTATGAGTGTTGATATTCAGGGTGTGATTAATGTAGTTATTTGTGCTATTGTGACTAT
ATTGCACACGTACAGGTTTGCCAAGGCTGGTTTTGTGACTTATGTTACATATTTTCTGTCGTT
GTTTGTGGTATTTTATAATTGGTGGTTTCATGGTGATCTGCTGAGTCTGTTTATGATGTTGCTT
CTCAATGTGTCAGATAGCTGGTATGTTGGTGCTATTGCATACAGAATATCATTACTTGCAGC
CACACAATTCCCTATGTTGGAACTTGTTGTTGGTAAGGCCAAGGTTGTGATTTTTATTTATGT
TGCAATTGGTTTTTTGTGCTGTATGTATTATGGTGTTCTGTATTGGATTAACAGATTCTGTAA
GTTGACACTTGGTGTATATGACTTTAAAGTCAGTGCTAATGAGTTTAAGTATATGGTTGCTA
ACGGGTTACGTGCTCCTAGAGGTGTGTTTGACTCAATGCTTCTGTCTATGAAACTTGTTGGTA
TTGGTGGTGATAAAGTTATAAAAGTTTCCACGGTACAATCCAAACTGACAGATATTAAGTG
CACTAATGTTGTTTTGCTTGGCTGTTTGTCTAGTATGAACATTGCAGCTAATACTAAAGAAT
GGGCTCATTGTGTCGAATTGCACAACAAGATTAATCTTTGTGATGATCCTGAAAAGGCACA
AGAAATGCTTCTTGCATTGCTAGCTTTCTTTATTAGCAAGCAAAAAGATTTTGGCATTGATG
ATTTGATTGATTCATACTTTGACAGTTCTAGTGTACTGCAGAGTGTTGCGTCTACATTCGTCA
ATATGCCTTCTTACATCGCTTATGAAAGTGCGCGGCAAAGTTATGAGGATGCTGTTAATAAT
GGTTCCTCTCCACAGCTCATTAAGCAGCTTAAGCGTGCTATGAATATTGCTAAAGCTGAGTT

TGACCATGAGTCTTCGGTTCAAAAGAAGATTAATCGCATGGCCGAACAAGCTGCTGCTCAG
ATGTTCAAGGAAGCACGTGCTGTTAATAGAAAGTCCAAGATTATTAGTGCTATGCACTCTTT
GCTTTTTAGCATGCTTCGTCGCTTGGACATGTCTAGTGTGGACACTATTCTTAGTCTTGCAAA
AGATGGTATAGTTCCTCTGTCGGTGATTCCCGCCACTTGTGCTACTAAACTTTCTATTGTTAG
CAGTGATTTTACGTCATTTGCTAAGATTGTTAAAGATGGTTGTGTGCACTATGCTGGTGTGGT
GTGGTCGATTGCTGAAATCACTGATGCTGATGGTAAGATCGTACATCTTAAGGAGGTTGTCG
AAGAGAACACAGATTCACTTACATGGCCACTCTTTGTTAGTTGCGTGCGTGTTGTGAAGCTT
CAGAATAATGAGATTATTCCTGGTAAGCTTAAACAGCGTGTTGTAAAGGCAGAGGGTGATG
GTTTCAGTGTTGAAGGTAAAGCACTCTTTAATAATGAGGGTGGTAAAACTTTTATGTACGCT
TTTGTCGCTGACAAACCAGATTTGAAATATGTTAAGTGGGAATTTGATGGTGGTTGTAACAC
TATTGAACTTGAGCCACCTTGTAGGTTTGCTGTTGAAACTGCAAATGGCACTGTTATAAAGT
ACTTGTATTTTGTGCGTAATCTAAACACATTGCGCAGGGGTGCAGTTTTGGGCTTTATTGGTG
CTACAGTGCGTTTGCAAGCCGGTAAACAAACAGAACTTGCTTCTAATTCGGCATTGCTTACT
ATGTGTGCTTTTGCTGTTGATCCTGCCAAGACGTATATTGACGCTGTTAAGAAGGGTGTTAA
ACCTGTCGGGAATTGCGTCAAGATGTTGTCAAATGGTTCAGGTACTGGCCAAGCTATTACTG
TTGGTGTTGAAGCTAATTCTAATCAAGATTCTTATGGTGGCGCTTCTGTCTGTTTGTATTGCC
GTGCACATGTTGAACATCCTACGATGGATGGTGCGTGTCGTTACAAAGGCAAGTATGTTCA
AGTCCCACTTGGTAATGCGGATCCTATACGGTTCTGTTTAGAAAATGAGCCATGTAAAGTTT
GTATGTGCTGGTTGAATAATGGTTGCACTTGTGATAGAACTTCTATTCAGAGTGTTGATACG
AGTTATTTAAACGAGCACGGGGCTCTAGTGCAGCTCGACTAGAGCCCTGTAATGGAACTGA
CACGGATCGTGTAGTCCGTGCTTTTGACATTTACAATAAGGATGTTGCGTGTATTGGCAAGT
TTCTTAAGGTCAATTGTGTCCGTTTTAAGAATCTTGACAAGCATGATGCCTTCTTTGTTGTAA
AACGTTGTACGAAGTCTGTCATGGAACACGAGCAGTCCATATATGACAAGCTTCACAGTTG
TGGTGCAGTTGCAGTTCATGATTTTTTCTTGTGGAAAGATGGCCGTTCAGTTTTTGGTAATAT
TAGCAGGCAAAACCTGACTAAGTATACAATGATGGACTTGTGTTATGCTTTGAGAAATTTTG
ATGAGCGTAATTGTGAAACACTTAAAGAAATTCTTGTCCTTACTGGTTGCTGTGACTATTCA
TATTTTGAAAATAAGGATTGGTACGATCCAGTTGAAAATGAGGACATTCACAAAGTTTATG
CACACCTTGGAACTGTTGTCGCTAATGCATTGCTTAAGTGCGTTAAGCTTTGCGATGCGATG
GTAGAGGCAGGTGTAGTGGGTGTGTTAACACTTGATAACCAAGACCTTAATGGTGATTTTTA
TGATTTTGGTGACTTTGCTACAACAATACCTAAAATGGGTGTACCATTATGTACCTCTTATTA
CTCTTATATGATGCCTGTAATGGGTATGACCAATTGTCTTGCATCTGAGTGTTTTATTAAGAG
TGACATTTTTGGTAGTGATTTTAAGACCTATGATTTGCTTGAGTACGACTTTACAGAGCACA
AAGAGGCTTTGTTTCGTAAGTACTTCAAGTATTGGGGTCAGGATTACCACCCTAATTGTGCT
GATTGTCACGACGAGCTTTGTATTGTTCATTGTGCGAATTTTAATACGTTGTTCGCTACGACT
ATTCCTAATACGGCTTTTGGTCCTCTGTGTCGCAAAGTGTTTGTTGATGGTGTGCCACTTGTT
ACTACAGCTGGCTACCATTTCAAGCAGTTGGGCCTTGTTTGGAATAAGGATATAAATACTC
ACTCTACTCGTTTGAGTGTAAATGAGTTGTTACAGTTTGTCACAGACCCTGCACTTTTGATAG
CTTCATCACCTGCCCTTGTTGATCAGCGTACTGTTTGTTTTTCAGTCGCTGCCTTGAGTACTG
GTATGACAAATCAGACAGTTAAGCCAGGCCACTTTAATAAAGAGTTCTACGACTTTTTGAG
ATCGCAAGGATTTTTTGAAGAAGGCTCTGAATTAACTTTAAAGCATTTCTTCTTTGCACAGA
AGGGTGATGCTGCTGTCAAAGATTTTGACTACTATCGTTATAATAGACCAACTATGCTAGAC
ATTTGTCAGGCTAGAGTCACTTATAAAATAGTGCAGCGTTATTTTGAGTGCTATGAGGGTGG
CTGTATTGCCGCTAAGGATGTTGTTGTTACTAACCTCAATAAAAGTGCAGGTTATCCACTTA
ATAAATTTGGTAAAGCTGGTTTGTATTATGAGTCTCTTAGTTATGAGGAACAAGATGCACTA
TACGCTCTTACAAAGCGTAATGTGTTACCTACTATGACACAGCTTAACCTTAAATATGCAAT
CAGTGGTAAAGAACGTGCTAGGACTGTTGGTGGTGTTTCATTGTTGTCTACTATGACTACTA
GACAGTTTCATCAGAAACACCTTAAGTCCATTGTTAATACACGTAATGCATCTGTTGTCATT
GGTACCACAAAATTTTATGGTGGTTGGGACAACATGCTTAAGAACCTTATTGATGGTGTCG
ACAATGCGTGCCTTATGGGTTGGGATTATCCTAAGTGCGATCGTGCACTTCCTAATATGATT
CGCATGATTTCTGCCATGATTTTGGGTTCGAAGCATGTTACTTGCTGTGATTCTTCTGATAAA
TATTACCGTCTCTGTAATGAGCTCGCTCAAGTGTTGACAGAAGTTGTATATTCTAATGGCGG
GTTTTATCTCAAGCCTGGTGGTACCACTTCAGGCGATGCTACAACGGCTTATGCGAACTCTG

TGTTTAATATTTTTCAAGCTGTTAGCGCCAATATTAACAGGTTGCTCAGCATTGATAGCAAT
GTGTGTAATAATATTGGTGTTAAGAGTCTGCAGCGTAAGCTATATGATTGTTGTTATAGATC
TTCTTCAGTAGATGATTCTTTTGTAGACAGTTATTATTGCTACTTGCGGAAACATTTTTCTATG
ATGATTTTGTCAGATGACGGTGTTGTGTGTTATAATAAAGATTATGCTGATTTGGGTTATGTG
GCAGATATTAATGCCTTTAAAGCTACCCTATATTATCAGAATAATGTCTTTATGAGTACTGC
TAAATGCTGGGTGGAGCCAGACATTAGCAAAGGTCCTCATGAGTTCTGTTCACAGCATACA
ATGCAGATTGTAGATTCTGACGGCAAGTATTATTTGCCATACCCAGACCCATCTAGAATATT
GTCTGCTGGTGTATTTGTCGATGATGTTGTTAAGACTGACGCTGTGATATTGTTGGAGCGCTA
CGTCTCATTGGCAATCGACGCATATCCTTTGTCTAAACATCATAATCCTGAGTATAGAAAAG
TGTTTTATACGCTTCTAGATTGGGTTAAGCACCTACACAACACACTTAATCAGGGCGTGCTT
GAGTCTTTCTCCGTTACATTGCTTGAGGATGTATCCTCTAAGTTTTGGGATGAGAGTTTTTAT
GCTAGTTTGTATGAAAAGTCTACAGCACTTCAAGCCGCAGGCCTATGTGTTGTTTGTGGTTC
TCAAACTGTACTGCGTTGTGGTGATTGTCTTAGACGACCCATGTTGTGCACTAAGTGTGCGT
ATGACCATGTTGTCACCACGAATCATAAATTCATTCTGGCTATAACACCTTATGTTTGTAAT
GCTTCTGGTTGCACCGTTAATGATGTGACTAAGTTGTATTTAGGAGGTCTGAGTTACTGGTGT
ATAGACCATAAACCTACGCTGTCACTGCCACTTTGTAGTGCAGGTAATGTTTTTGGTTTGTA
CAAGAACTCTGCTACTGGTTCACCGGATGTAGAGGTTTTTAATACACTTGCCACGTCAGATT
GGACTGACGTTAAAGACTACAAGCTGGCTAATGAATGCAAGGATTCTCTTAGGCTTTTTGC
GGCTGAGACTGTTAAAGCCAAAGAAGAGAGTGTTAAATCCTCTTATGCTTGTGCTACACTT
AAAGAAGTTGTTGGGCCACGTGAGTTGATTCTTAGCTGGGAGTGCGGAAAAATTAAACCGC
CACTTAACCGTAATTCAGTGTTTACGTGTTTCCAGATTTCTAAAGACTCTAAGTTCCAGGTTG
GAGAGTTTACGTTCGAAAAATTGGATTACGGTTCTGATGCTGTGTGCTACAAGTCTACCACC
ACAGTCAAATTAGTGCCTGGTATGATTTTTGTTCTCACATCACACAATGTACAGCCGCTTAG
GGCTCCCACTATTGCTAATCAAGAACGCTATGCTTCAATTTACAAACTGCACCCCACTTTTA
ATATTAGTGATGCTTATGCCAATTTGGTGCCGTACTACCAAATGATTGGCAGACAACGAAT
CACTACTATTCAAGGCCCACCGGGAAGTGGTAAGTCGCATTGTGTTATAGGTCTGGGTCTTT
ATTACCCAGGTGCTAGGATGGTTTTTGCGGCTTGCTCTCATGCAGCCGTAGATTCACTTTGTG
TCAAGGCTGCCACTGCTTATAGTGTAGACAAATGCACTCGTATTATCCCTGCCCGTGCTAGG
GTTGAGTGTTATAGTGGATTTAAGGTAAATAACACTCAGGCACAGTACATCTTTTCAACAGT
CAATGCACTCCCTGAGTGTAATGTTGACATAGTTGTTATAGATGAAGTGTCTATGTGTACTA
ATTATGACCTGTCCGTTATTAATCAGCGGTTGTCTTACAAACATATTGTTTATGTGGGTGACC
CACAGCAATTACCCGCGCCACGTACCATGATCACTAAAGGCGTTCTCGAACCTAAGGACTA
TAATGTTGTAACTCAGAGGATGTGTGCTGTTGGGCCAGATGTCTTCTTGCATAAATGTTACA
GATGTCCTGCGGAGATTGTTAAAACAGTCTCAGAGTTGGTTTACGAGAATAAGTTTGTACCT
GTTCATGCTGATAGCAAACAGTGCTTTAAACTGTTTTGTAAGGGTAATGTTCAGGTTGACAA
TGGTTCTAGTATTAACAGAAAGCAATTGGATATCGTTAAGATGTTCCTTGCTAAGAATCCAC
GTTGGTCTAAAGCTGTGTTTATCTCACCTTATAATAGTCAAAATTATGTGGCTAGTAGGGTTC
TGGGTTTGCAGATTCAGACCGTTGATTCCTCTCAAGGTAGTGAATATGACTATGTCATATAT
ACACAGACGTCAGACACAGCACATGCTTGCAATGTTAATAGATTTAATGTTGCAATCACAA
GAGCCAAGAAGGGTATTCTTTGTGTTATGTGTGATCGGGATCTGTTTGATGCTCTGAAATTTT
TCGAGATCAAGCTTTCTGACTTGCAGGCTAGTGGCGACGGCTGTGGTCTTTTTAAAGATTGT
TACAGGAAAGATGATGAATTACCTCCATCTCATGCAGCTACCTATATGGCCTTGTCTGACA
ATTTTAAGGTCAATGGTGATCTTGCTGTGCAGATTGGCTTTAAAGGTGTGTGTAAGTATGAG
CATGTTATTTCTTTTATGGGCTTCAGGTTTGACATTAATGTGCCTGAACAGCATAGTCTTTTC
TGTACCAGGAATTTTGCAATGCGCCATGTGCGTGGTTGGCTTGGTATGGATGTAGAAGGTGC
CCATGTTACAGGTGAAAATGTAGGTACGAACGTACCATTACAAGTAGGCTTCTCTAATGGT
GTTGATTTTGTTGTCCAACCAGAAGGTTGTGTTGTCACTGAGACAGGCAGCAGGATCGTACC
TGTTAGAGCTCGTGCTCCGCCAGGTGAACAGTTCACACATTTGGTCCCCCTGCTTCGTAAGG
GACAACCATGGGCTGTGGTTCGTAAACGCATTGTGCAGATGTGTTGCGATTACTTGGTGAAC
CTATCAGACATATTAATCTTTGTTTTGTGGGCTGGTGGTTTGGAGTTGACTACCATGAGATAT
TTTGTTAAAATTGGACCAATCAAAGTTTGTGAGTGCGGCAAAGTGGCCACTTGTTACAATA
GTGTGACTCATGCATACTATTGCTTCAAGCATGCCTTAGGGTGTGATTATTTGTACAATCCTT

ATGTTATTGACATTCAGCAGTGGGATTATACAGGTTCACTTAGCCATAATCATCATGAGCAT
TGTAATGTACATCGCAATGAGCATGTTGCATCAGGTGATGCTATTATGACTCGTTGTCTTGC
TGTGTATGATTGCTTCGTCAAGAACGTTGACTGGTCCATTACTTACCCATTCATTGCTAATGA
ACATGCCATTAATAAGAGTGGTAGAGTTGTGCAATCTATGGTTGTCAAATCAGCTATAAAG
TTATATAATCCTAAAGTAATTCATGATATTGGTAATCCTAAAGGTATTAGGTGTGCAGTTAC
TGATGCACACTGGTTTTGCTATGATAAGCAGCCCCTCAATTCCAATGTCAAGTTGCTTGAGT
ATGATTATATAACTCATGGTCAGATGGATGGGTTATGCCTATTTTGGAATTGCAATGTTGAC
ATGTATCCTGAATTTTCTATTGTGTGCCGTTTTGACACTCGTTGTAGGTCAAATCTCAACTTA
GAAGGTGTTAATGGAGGTTCACTTTATGTCAACAACCATGCATTTTTTACACCTGCCTTTGAT
AAGCGCGCAATGGCTAAACTTAAGCCTATGCCCTTCTTTTTCTATGATGACACGCCTTGCGA
TCGTGTTCAGGATCAAATAAATTATGTACCACTGCGTGCTAGTACTTGTATTACAAAGTGTA
ATATTGGTGGTGCTGTTTGCAGTAAGCATGCCAACATGTTTCATGCCTATGTTAATGCATAT
AATACTTTCACGCAGGCGGGTTTTACTATTTGGGTTCCTGTAACTTTTGATTTATTCAACCTTT
GGCAGACATTTGTTGAAACTAATTTGCAAGGCTTGGAGAACATAGCGTTTAATGTCCTCAA
GAAAGGGTCCTTTGTTGGTGTAGAGGGTGAACTACCGGTTGCTATCGTTAATGACCGTGTGC
TGGTACGTGATGGCACCGTGGATAATTTGATTTTTACTAATAAAACCTCATTACCCACTAAT
GTTGCTTTTGAGCTTTACGCCAAGCGTAAAATGGGTTTGACACCACCTCTTGCTGTTCTCAG
GAATCTCGGTGTTGTTAGTACCTATAAGTTTGTGCTATGGGATTATGAGGCAGAAAGACCAT
TTACTACTTTTACTAAAAATGTTTGCAGTTACACCGACTTTGATGAGGATGTGTGCACTTGCT
TTGATAATAGCATTCAGGGTTCGTTTGAACGCTTTTCAATGGCAAACAATGCAGTGCTTATT
TCCACGTCTGCTGTTAAGAAGCTTACTTGTATTAAGCTTAACTATGGTTTGCTTAATGGTGTG
GCTGTTTCTAAGACTGGTGACAAGCCTGTTACATGGTATATCTATGTCCGGAAAAATGGTCA
ATTTGTTGACCAATTTGACGGATACTTTACTCAGGGGCGTACTACTATTGATTTCTTACCTCG
TAGTGTTATGGAGGAAGACTTTTTAAACCTTGATATGGGTTTGTTCATTAGCAAGTATGGTC
TTGAAGACTATGCTTTTGAACATGTTGTGTATGGTGACGTGTCTGGTACTACACTTGGTGGA
CTCCATTTATTAATTTCTCAAGTACGCCTTGGAAAAATGGGTGTCTTGAAAGTTGATGAGTT
CACACATAGTGAGGATAGTACATTGAAGTCCTGTACAGTCACCTATTCTAATGATCCTAGTA
GTAAAATGGTTTGTACCTATATGGATTTGTTGCTAGATGATTTTGTTACCATTTTAAAATCTT
TGGATTTGGGTGTAATTTCCAGAGTTCATGAAGTTGTAGTTGATTGTAAGGTTTGGCGTTGG
ATGTTGTGGTGTAAGGACCACAAAGTTCAAACGTTCTACCCACAGTTGCAGTCGTCAGAAT
GGAAATGTGGCTATTCCATGCCCTCACTGTATAAAATACAGCGTATGTGTCTTGAGCCCTGT
AATTTGTACAATTATGGTGCTAGTCTTAAGCTACCTGAAGGTATCATGTTTAACGTGGTTAA
ATACACTCAGTTGTGTCAATACTTGAATAGTACTACTATGTGTGTACCGCATAATATGCGTG
TACTACATCTTGGTGCTGGCTCAGATAAAGGAGTCGCACCGGGTACTGCTGTATTGCGTCGT
TGGTTACCCAGTGATGCTATTATTGTTGACAATGATGTTAATGACTATGTTAGTGATGCAGA
TTTCAGTGTAACAGGGGATTGCGCCACTTTGTACTTACAGGATAAGTTCGACTTAGTGGTTT
CAGACATGTATGATGGCCGTATTAAACAGTGTGATGGTGTTAACGTTTCAAAGGATGGCTT
CTTTCCTTACATCAATGGTGTGATATCAGAGAAGCTTTCCCTAGGTGGTACAGTTGCCATTA
AGATTACTGAGTATAGCTGGAATAAGCGGCTGTATGAGTTGATACAAAAGTTTGAATTTTG
GACGTTGTTTTGTACTAGTGTGAATACATCATCGTCTGAAGCTTTTCTAATTGGTGTCAACTA
TCTTGGCGACTACGCCACTCAACCCATTGTGGATGGTAATACTATGCACGCTAATTATGTGT
TTTGGCGTAATTCTACCATTATGTCAATGTCATATAATAGTGTGCTTGACCTAGCAAAATTTA
ATTGTAGACACAAAGCCACTGTTGTTATTTCACTTAAAGAAAGTGATTTGAATGATGTTGTG
GTAGGCCTCATAAAGAATGGAAAGTTGTTGGTTAGAAAGAGTGGTGTCATTTGTGACTATG
GTAACCATTTAGTTTCAACTAAATAA
>SUOHC3005
AGTGTTGATACGAGTTATTTAAACGAGCACGGGGCTCTAGTGCAGCTCGACTAGAGCCCTG
TAATGGAACTGACACGGATCGTGTAGTCCGTGCTTTTGACATTTACAATAAGGATGTTGCGT
GTATTGGCAAGTTTCTTAAGGTCAATTGTGTCCGTTTTAAGAATCTTGACAAGCATGATGCC
TTCTTTGTTGTAAAACGTTGTACGAAGTCTGTCATGGAACACGAGCAGTCCATATATGACAA
GCTTCACAGTTGTGGTGCAGTTGCAGTTCATGATTTTTTCTTGTGGAAAGATGGCCGTTCAGT

TTTTGGTAATATTAGCAGGCAAAACCTGACTAAGTATACAATGATGGACTTGTGTTATGATT
TGAGAAATTTTGATGAGAGTAATTGTGAAACACTTAAAGAA
>SUOHC3007
ACTATTCAAGGCCCACCGGGAAGTGGCAAGTCTCATTGTGTTGTAGGTCTGGGTCTTTATTA
CCCAGGTGCTAGGATGGTCTTTGCGGCTTGCTCTCATGCAGCCGTAGATTCACTTTGTGTTA
AGGCTGCCACTGCTTATAGTGTAGACAAATGCACTCGTATTATCCCTGCCCGTGCTAGGGTT
GAGTGTTATAGTGGGTTTAAGGTAAATAACACTCAGGCACAGTATATCTTTTCAACAGTCA
ACGCACTCCCTGAGTGTAATGTTGACATAGTTGTTATAGATGAAGTGTCTATGTGTACTAAT
TATGACCTGTCCGTTATTAATCAGCGGTTGTCTTATAAACATATTGTTTATGTGGGTGATCCA
CAGCAATTACCAGCGCCACGTACCATGATCACTAAAGGCGTCCTCGAACCTAAGGACTATA
ATGTTGTAACTCAGAGGATGTGTGCTGTTGGGCCAGATGTCTTCTTGCATAAATGTTACAGA
TGTCCTGCTGAGATTGTTAAAACAGTCTCAGAGTTGGTTTATGAGAATAAGTTTGTACCTGT
TCATGCTGATAGCAAACAGTGCTTTAAACTGTTTTGTAAGGGTAATGTTCAGGTTGACAATG
GTTCTAGTATTAACAGAAAGCAATTGGATATCGTTAAGATGTTCCTTGCTAAGAATCCACGT
TGGTCTAAAGCTGTGTTTATCTCACCTTATAATAGTCAGAATTATGTGGCTAGTAGGGTTTTG
GGTTTGCAGATTCAGACTGTTGATTCCTCTCAAGGTAGTGAATATGACTATGTCATATACAC
ACAGACATCAGACACAGCACATGCTTGCAATGTTAATAGATTTAATGTTGCAATTACAAGA
GCCAAGAAGGGTATTCTTTGCGTTATGTGTGATCGGGAGTTGTTTGATGCTCTGAAATTTTTC
GAAATCAAGCTTTCTGACTTGCAGGCTAGTGGTGATGGCTGTGGTCTTTTTAAAGATTGTTA
CAGGAAAGATGATGAATTACCTCCATCTCATGCAGCTACCTATATGGCCTTGTCTGACAACT
TTAAGGTCAATGGTGATCTTGCTGTGCAGATTGGCTTTAAAGGTGTGTGTAAGTATGAGCAT
GTTATTTCTTTTATGGG
>SUOHC2001-ON893137
AGTGTCGACACGAGTTATTTAAACGAGCACGGGGCTCTAGTGCAGCTCGACTAGAGCCCTG
TAATGGAACTGACACGGATCGTGTAGTCCGTGCTTTTGACATTTACAATAAGGATGTTGCGT
GTATTGGCAAGTTTCTTAAGGTCAATTGTGTCCGTTTTAAGAATCTTGACAAGCATGATGCTT
TCTTTGTTGTAAAACGTTGTACGAAGTCTGTCATGGAACACGAGCAGTCCATATATGACAA
GCTTCACAGTTGTGGTGCAGTTGCAGTTCATGATTTTTTCTTGTGGAAAGATGGCCGTTCAGT
TTTTGGTAATATTAGCAGGCAAAACCTGACTAAGTATACAATGATGGACTTGTGTTATGCTT
TGAGAAATTTTGATGAGCGTAATTGTGAAACACTTAAAGAAATTCTTGTCCTTACTGGTTGC
TGTGACTATTCATATTTTGAAAATAAGGATTGGTACGACCCAGTTGAAAATGAGGACATTC
ATAAAGTTTATGCACACCTTGGAACTGTTGTCTCTAATGCATTGCTTAAGTGTGTTAAGCTTT
GCGATGCGATGGTAGAGGCAGGTGTAGTGGGTGTGTTAACACTTGATAACCAAGACCTTAA
TGGTGATTTTTATGATTTTGGTGACTTTGCTACAACAATACCTAAAATGGGTGTACCATTATG
TACCTCTTATTACTCTTATATGATGCCTGTAATGGGTATGACCAATTGTCTTGCATCTGAGTG
TTTTATTAAGAGTGACATTTTTGGTAGTGATTTTAAGACCTATGATTTGCTTGAGTACGACTT
TACAGAGCACAAAGAGGCTTTGTTTCGTAAGTACTTCAAGTATTGGGGTCAGGATTACCAC
CCTAATTGTGCTGATTGTCACGACGAGCTTTGTATTGTTCATTGTGCGAATTTTAATACGTTG
TTCGCCACGACTATTCCTAATACAGCTTTTGGTCCTCTGTGTCGCAAAGTGTTTGTTGATGGT
GTGCCACTTGTTACTACAGCTGGCTACCATTTCAAGCAGTTGGGCCTTGTTTGGAATAAGGA
TATAAATACTCACTCTACTCGTTTGAGTGTAAATGAGTTGTTACAGTTTGTCACAGACCCTG
CACTTTTGATAGCATCATCACCAGCCCTTGTGGATCAGCGTACTGTTTGTTTTTCAGTCGCTG
CCTTGAGTACTGGTATGACAAATCAGACAGTTAAGCCAGGCCACTTTAATAAAGAGTTCTA
CGACTTTTTGAGATCGCAAGGATTTTTTGAAGAAGGCTCTGAATTAACTTTAAAGCATTTCT
TCTTTGCACAGAAGGGTGATGCTGCTGTCAAAGATTTTGACTACTATCGTTATAATAGACCA
ACTATGCTAGACATTTGTCAGGCTAGAGTCACTTATAAAATAGTGCAGCGTTATTTTGAGTG
CTATGAGGGTGGCTGTATTGCCGCTAAGGATGTTGTTGTTACTAACCTCAATAAAAGTGCAG
GTTACCCACTTAATAAATTTGGTAAAGCTGGTTTGTATTATGAGTCTCTTAGTTATGAGGAA
CAAGATGCACTATACGCTCTTACAAAGCGTAATGTGTTACCTACTATGACACAGCTTAACCT
TAAATATGCAATCAGTGGTAAAGAACGTGCTAGGACTGTTGGTGGTGTTTCATTGTTGTCTA
CTATGACTACTAGACAGTTTCATCAGAAACACCTTAAGTCCATTGTTAATACACGTAATGCA
TCTGTTGTCATTGGTACCACAAAATTTTATGGTGGTTGGGACAACATGCTTAAGAACCTTAT

TGATGGTGTTGACAATGCGTGCCTTATGGGTTGGGATTACCCTAAGTGTGATCGTGCACTCC
CTAATATGATTCGCATGATTTCTGCCATGATTTTGGGTTCGAAGCATGTTACTTGCTGTGATT
CTTCTGATAAATATTACCGTCTCTGTAATGAGCTCGCTCAAGTGTTGACAGAAGTTGTATAT
TCTAATGGTGGGTTTTATCTCAAGCCTGGTGGTACCACTTCTGGCGATGCTACAACGGCTTA
TGCGAACTCTGTGTTTAATATTTTTCAAGCTGTTAGCGCCAATATTAACAGGTTGCTTAGCAT
TGATAGCAATGTGTGTAATAATATTGGTGTTAAGAGTTTGCAGCGTAAGCTATATGATTGTT
GTTACAGATCTTCTTCAGTAGATGATTCTTTTGTAGACAGTTATTATTGCTACTTGCGGAAAC
ATTTTTCTATGATGATTTTGTCAGATGACGGTGTTGTGTGTTATAATAAAGATTATGCTGATT
TGGGTTATGTGGCAGATATCAATGCCTTTAAAGCTACCCTATATTATCAGAATAATGTCTTT
ATGAGTACTGCTAAATGCTGGGTGGAGCCAGACATTAGCAAAGGTCCTCATGAGTTCTGTT
CACAGCATACTATGCAGATTGTAGATTCTGACGGCAAGTATTATTTGCCATACCCAGACCC
ATCTAGAATATTGTCTGCTGGTGTATTTGTCGATGATGTTGTTAAGACTGACGCTGTGATATT
GTTGGAGCGCTACGTCTCATTGGCAATCGACGCATATCCTTTGTCTAAACATCATAATCCTG
AATATAGAAAAGTGTTTTATACACTTCTAGATTGGGTTAAGCATCTACACAACACACTTAAT
CAGGGCGTGCTTGAGTCTTTCTCCGTTACATTGCTTGAGGATGTATCCTCTAAGTTTTGGGAT
GAGAGTTTTTATGCTAGTTTGTATGAAAAGTCTACAGTACTTCAAGCCGCAGGCCTATGTGT
TGTTTGTGGTTCTCAAACTGTACTGCGTTGTGGTGATTGTCTTAGACGACCTATGTTGTGCAC
TAAGTGTGCTTACGACCATGTTGTCACCACGAATCATAAATTCATTCTGGCTATAACACCTT
ACGTTTGTAATGCTTCTGGTTGCACCGTTAATGATGTGACTAAGTTGTATTTAGGAGGTCTG
AGTTACTGGTGTATAGACCATAAACCTACACTGTCACTACCACTTTGTAGTGCAGGTAATGT
TTTTGGTTTGTATAAAAACTCTGCTACAGGTTCACCGGATGTAGAGGTTTTTAATACACTTGC
CACGTCAGATTGGACTGACGTTAAAGATTACAAGCTGGCTAATGAATGCAAGGATTCTCTT
AGGCTTTTTGCGGCTGAGACTGTTAAAGCCAAAGAAGAGAGTGTTAAATCCTCTTATGCTTG
TGCTACACTTAAAGAAGTTGTTGGGCCACGTGAGTTGATTCTTAGTTGGGAGTGCGGAAAA
ATTAAACCGCCACTTAACCGTAATTCAGTGTTTACGTGTTTCCAGATTTCTAAAGACTCTAA
GTTCCAGGTTGGAGAGTTTACGTTTGAAAAATTGGATTACGGTTCTGATGCTGTGTGTTACA
AGTCTACCACCACAGTTAAATTAGTGCCTGGTATGATTTTTGTTCTCACATCACACAATGTA
CAGCCGCTTAGGGCTCCCACTATTGCTAATCAAGAACGCTATGCTTCAATTTATAAACTGCA
CCCTACTTTTAATATTAGTGATGCTTATGCCAATTTGGTGCCGTACTACCAAATGATTGGCA
GACAACGAATCACTACTATTCAAGGCCCACCGGGAAGTGGCAAGTCCCATTGTGTTATAGG
TCTGGGTCTTTATTACCCAGGTGCTAGGATGGTCTTTGCGGCTTGCTCTCATGCAGCCGTAG
ATTCACTTTGTGTCAAGGCTGCCACTGCTTATAGTGTAGACAAATGCACTCGTATTATCCCT
GCCCGTGCTAGGGTTGAGTGTTATAGTGGGTTTAAGGTAAATAACACTCAGGCACAGTACA
TCTTTTCAACAGTCAACGCACTCCCTGAGTGTAATGTTGACATAGTTGTTATAGATGAAGTG
TCTATGTGTACTAATTATGACCTGTCCGTTATTAACCAGCGGTTGTCTTATAAACATATTGTT
TATGTGGGTGACCCACAGCAATTACCAGCGCCACGTACCATGATCACTAAAGGCGTTCTCG
AACCTAAGGACTATAATGTTGTAACTCAGAGGATGTGTGCTGTTGGGCCAGATGTCTTCTTG
CATAAATGTTACAGATGTCCTGCTGAGATTGTTAAAACAGTCTCAGAGTTGGTTTATGAGAA
TAAGTTTGTACCTGTTCATGCTGATAGCAAACAGTGTTTTAAACTGTTTTGTAAGGGCAATG
TTCAGGTTGACAATGGTTCTAGTATTAACAGAAAGCAATTGGATATCGTCAAGATGTTCCTT
GCTAAGAATCCACGTTGGTCTAAAGCTGTGTTTATCTCACCTTATAATAGTCAGAATTATGT
GGCTAGTAGGGTTTTGGGTTTGCAGATTCAGACTGTTGACTCCTCTCAAGGTAGTGAATATG
ACTATGTCATATACACACAGACGTCAGACACAGCACATGCTTGCAATGTTAATAGATTTAA
TGTTGCAATTACAAGAGCCAAGAAGGGTATTCTTTGTGTTATGTGTGATCGGGAGTTGTTTG
ATGCTCTGAAATTTTTCGAGATCAAGCTTTCTGACTTGCAGGCTAGTGGTGATGGCTGTGGT
CTTTTTAAAGATTGTTACAGGAAAGATGATGAATTACCTCCATCTCATGCGGCTACCTATAT
GGCCTTGTCTGACAACTTTAAGGTCAATGGTGATCTTGCTGTTCAGATTGGCTTTAAAGGTG
TGTGTAAGTATGAGCATGTTATTTCTTTTATGGGCTTCAGGTTTGATATTAATGTGCCTGAAC
AGCATAGTCTTTTCTGTACCAGGAATTTTGCAATGCGCCACGTGCGTGGTTGGCTTGGTATG
GATGTAGAAGGTGCCCACGTTACAGGTGAAAATGTAGGTACTAATGTACCATTACAAGTAG
GCTTCTCTAATGGTGTTGATTTTGTTGTCCAACCGGAAGGTTGTGTTGTCACTGAGACAGGC
AGCAGGATCGTACCTGTTAGAGCTCGTGCTCCGCCAGGTGAACAGTTCACACATTTGGTCC

CCCTGCTTCGTAAGGGACAACCGTGGGCTGTGGTTCGTAAACGCATTGTTCAGATGTGTTGC
GATTACTTGGTGAACCTATCAGACATATTAATCTTTGTTTTGTGGGCTGGTGGTTTGGAGTTG
ACTACCATGAGATATTTTGTTAAAATTGGACCAAGCAAAGTTTGTGAGTGCGGCAAGGTGG
CCACTTGTTACAATAGTGTGACTCATGCATACTACTGTTTCAAGCATGCCTTAGGGTGTGAT
TATTTGTACAATCCTTATGTTATTGACATTCAGCAGTGGGATTATACAGGTTCACTTAGCCAT
AATCATCATGAGCATTGTAATGTACATCGCAATGAGCATGTTGCATCTGGTGATGCTATTAT
GACTCGTTGTCTTGCTGTGTATGATTGCTTCGTCAAGAACGTTGATTGGTCCATTACTTACCC
ATTCATTGCTAATGAGCATGCCATTAATAAGAGTGGTAGAGTTGTGCAATCTATGGTTGTCA
AAACAGACCATGAGTTATATAATCCTAAAGTAATTCATGATATTGGTAATCCTAAAGGTAT
TAGGTGTGCAGTTACTGATGCACACTGGTTTTGCTATGATAAGCAGCCCCTCAATTCCAATG
TCAAGTTGCTTGAGTATGATTATATAACTCATGGTCAAATGGATGGGTTATGCCTATTTTGG
AATTGCAATGTTGACATGTATCCTGAATTTTCTATTGTGTGCCGTTTTGACACTCGTTGTAGG
TCAAATCTCAACTTAGAAGGTGTTAACGGAGGTTCACTTTATGTCAACAATCATGCATTTTT
TACACCTGCATTTGATAAGCGTGCAATGGCTAAACTTAAGCCTATGCCCTTCTTTTTCTATGA
TGACACGCCTTGCGATCGTGTTCATGACCAGATAAATTATGTACCACTGCGTGCTAGTACTT
GTATTACAAAGTGTAATATTGGTGGTGCTGTTTGCAGTAAGCATGCCAATATGTTTCATGCC
TATGTTAATGCATATAATACTTTCACGCAGGCTGGTTTTACTATTTGGGTTCCTGTAACTTTT
GATTTGTTTAACCTTTGGCAAACATTTGTCGAAACTAACTTGCAAGGCTTGGAGAACATAGC
GTTTAATGTCCTCAAGAAAGGGTCTTTTGTTGGTGTAGAGGGTGAACTACCGGTTGCTATCG
TTAATGACCGTGTGCTGGTACGTGATGGCACCGTGGATAATTTGATTTTTACTAATAAAACC
TCATTACCTACTAATGTTGCTTTCGAGCTTTACGCCAAACGTAAAATGGGTTTGACACCACC
TCTTGCTGTTCTCAGGAATCTCGGTGTTGTTAGTACCTATAAGTTTGTGTTATGGGATTATGA
GGCAGAAAGACCATTTACTACTTTTACTAAAAATGTTTGCAGTTACACAGACTTTGACGAG
GATGTGTGCACTTGCTTTGATAATAGCATTCAGGGTTCGTTTGAACGCTTTTCAATGGCTAA
CAATGCAGTGCTTATTTCCACATCTGCTGTTAAGAAGCTTACTTGTATTAAGCTTAACTATG
GTTTGCTTAATGGTGTGGCTGTTTCTAAGACTGGTGACAAGCCTGTTACATGGTATATCTATG
TCCGTAAAAATGGTCAATTTGTTGACCAATTTGACGGATATTTTACTCAGGGGCGTACTACT
ATTGATTTCTTACCTCGTAGTGTTATGGAGGAAGACTTTTTAAACCTTGATATGGGTTTGTTT
ATTAGTAAGTACGGTCTTGAGGACTATGCTTTTGAGCATGTCGTGTATGGTGACGTGTCTGG
TACTACACTTGGTGGACTACATTTATTAATTTCTCAAGTACGCCTTGGAAAAATGGGTGTCT
TGAAAGTGGATGAGTTCACACATAGTGAGGATAGTACATTGAAGTCCTGTACAGTCACCTA
TTCTAATGATCCTAGTAGTAAAATGGTTTGTACCTATATGGATTTGTTGCTAGATGATTTTGT
TACCATTTTAAAATCTTTGGATTTGGGTGTAATTTCTAGAGTTCATGAAGTTGTAGTCGATTG
TAAGGTTTGGCGTTGGATGTTGTGGTGTAAGGACCACAAAGTTCAAACGTTCTACCCACAG
TTGCAGTCGTCAGAATGGAAATGTGGTTATTCCATGCCCTCACTGTATAAAATACAGCGTAT
GTGTCYTGAGCCCTGTAATTTGTACAATTATGGTGCTAGTCTTAAGCTACCTGAAGGTATCA
TGTTTAACGTGGTCAAATACACTCAGTTATGTCAATATTTGAATAGTACTACTATGTGTGTA
CCGCATAATATGCGTGTACTACATCTTGGTGCTGGCTCAGATAAAGGAGTCGCACCTGGTA
CTGCTGTATTGCGTCGTTGGTTACCCAGTGATGCTATTATTGTTGACAATGATGTTAATGACT
ATGTTAGTGATGCAGATTTCAGTGTAACAGGGGATTGCGCCACTTTGTACTTACAGGATAA
GTTCGACTTAGTGGTTTCAGACATGTATGATGGCCGTATTAAACAGTATGATGGTGTTAATG
TTTCAAAGGATGGCTTCTTTCCTTACATCAATGGTGTGATATCAGAGAAGCTTTCCCTAGGT
GGTACAGTTGCCATTAAGATTACTGAGTATAGCTGGAATAAGCGGCTGTATGAGTTGATAC
AAAAGTTTGAATTTTGGACGTTGTTTTGTACTAGTGTGAATACATCATCGTCTGAAGCTTTTC
TAATTGGTGTCAACTATCTTGGCGACTACGCTACTCAGCCCATTATGGATGGCAATACTATG
CACGCTAATTATGTGTTTTGGCGTAATTCTACAATTATGTCCATGTCATATAATAGTGTGCTT
GATCTAGCAAAGTTTAATTGTAGACATAAAGCTACGGTTGTTATTTCACTTAAAGAAAGTG
ATTTGAATGATGTTGTGGTAGGCCTTATAAAGAATGGAAAGTTGTTGGTTAGAAAGAGTGG
TGTCATTTGTGACTATGGTAACCATTTAGTTTCAACTAAATAA
>SUOHC2004
GTGTTGTTTGTGGTTCTCAAACTGTACTGCGTTGTGGTGATTGTCTTAGACGACCTATGTTGT
GCACTAAGTGTGCGTATGACCATGTTGTCACCACGAATCATAAATTCATTCTGGCTATAACA

CCTTACGTTTGTAATGCTTCTGGTTGCACCGTTAATGATGTGACTAAGTTGTATTTAGGAGGT
CTGAGTTACTGGTGTATAGACCATAAACCTACGCTGTCACTGCCACTTTGTAGTGCAGGTAA
TGTTTTTGGTTTGTATAAAAACTCTGCTACTGGTTCACCGGATGTAGAGGTTTTTAATACACT
TGCCACGTCAGATTGGACTGACGTTAAAGATTACAAGCTGGCTAATGAATGCAAGGATTCT
CTTAGGCTTTTTGCGGCTGAGACTGTTAAAGCCAAAGAAGAGAGTGTTAAATCCTCTTATGC
TTGTGCTACACTTAAAGAAGTTGTTGGGCCACGTGAGTTGATTCTTAGCTGGGAGTGTGGAA
AAATTAAACCGCCACTTAACCGTAATTCAGTGTTTACGTGTTTCCAGATTTCTAAAGACTCT
AAGTTCCAGGTTGGAGAGTTTACGTTTGAAAAATTGGATTACGGTTCTGATGCTGTGTGCTA
CAAGTCTACCACCACAGTCAAATTAGTGCCTGGTATGATTTTTGTTCTCACATCACACAATG
TACAGCCGCTTAGGGCTCCCACTATTGCTAATCAAGAACGCTATGCTTCAATTTATAAGCTG
CACCCTACTTTTAATATTAGTGATGCTTATGCCAATTTGGTGCCGTACTACCAAATGATTGG
CAGACAACGAATCACTACTATTCAAGGCCCACCGGGAAGTGGCAAGTCTCATTGTGT
>SUOHC1001
AGTGTTGATACGAGTTATTTAAACGAGCACGGGGCTCTAGTGCAGCTCGACTAGAGCCCTG
TAATGGAACTGACACGGATCGTGTAGTCCGTGCTTTTGACATTTACAATAAGGATGTTGCGT
GTATTGGCAAGTTTCTTAAGGTCAATTGTGTCCGTTTTAAGAATCTTGACAAGCATGATGCC
TTCTTTGTTGTAAAACGTTGTACGAAGTCTGTCATGGAACACGAGCAGTCCATATATGACAA
GCTTCACAGTTGTGGTGCAGTTGCAGTTCATGATTTTTTCTTGTGGAAAGATGGCCGTTCAGT
TTTTGGTAATATTAGCAGGCAAAACCTGACTAAGTATACAATGATGGACTTGTGTTATGCTT
TGAGAAATTTTGATGAGCGTAATTGTGAAACACTTAAAGAAATTCTTGTCCTTACTGGTTGC
TGTGACTATTCATATTTTGAAAATAAGGATTGGTACGACCCAGTTGAAAATGAGGACATTC
ATAAAGTTTATGCACACCTTGGAACTGTTGTCTCTAATGCATTGCTTAAGTGCGTTAAGCTTT
GCGATGCGATGGTAGAGGCAGGTGTAGTGGGTGTGTTAACACTTGATAACCAAGACCTTAA
TGGTGATTTTTATGATTTTGGTGACTTTGCTACAACAATACCTAAAATGGGTGTACCATTATG
TACCTCTTATTACTCTTATATGATGCCTGTAATGGGTATGACCAATTGTCTTGCATCTGAGTG
TTTTATTAAGAGTGACATTTTTGGTAGTGATTTTAAGACCTATGATTTGCTTGAGTACGACTT
TACAGAGCACAAAGAGGCTTTGTTTCGTAAGTACTTCAAGTATTGGGGTCAGGATTACCAC
CCTAATTGTGCTGATTGTCACGACGAGCTTTGTATTGTTCATTGTGCGAATTTTAATACGTTG
TTCGCTACGACTATTCCTAATACGGCTTTTGGTCCTCTGTGTCGCAAAGTGTTTGTTGATGGT
GTGCCACTTGTTACTACAGCTGGCTACCATTTCAAGCAGTTGGGCCTTGTTTGGAATAAGGA
TATAAATACTCACTCTACTCGTTTGAGTGTAAATGAGTTGTTACAGTTTGTCACAGACCCTG
CACTTTTGATAGCATCATCACCAGCCCTTGTGGATCAGCGTACTGTTTGTTTTTCAGTCGCTG
CCTTGAGTACTGGTATGACAAATCAGACAGTTAAGCCAGGCCACTTTAATAAAGAGTTCTA
CGACTTTTTGAGATCGCAAGGATTTTTTGAAGAAGGCTCTGAATTAACTTTAAAGCATTTCT
TCTTTGCACAGAAGGGTGATGCTGCTGTCAAAGATTTTGACTACTATCGTTATAATAGACCA
ACTATGCTAGACATTTGTCAGGCTAGAGTCACTTATAAAATAGTGCAGCGTTATTTTGAGTG
CTATGAGGGTGGCTGTATTGCCGCTAAGGATGTTGTTGTTACTAACCTCAATAAAAGTGCAG
GTTACCCACTTAATAAATTTGGTAAAGCTGGTTTGTATTATGAGTCTCTTAGTTATGAGGAA
CAAGATGCACTATACGCTCTTACAAAGCGTAATGTGTTACCTACTATGACACAGCTTAACCT
TAAATATGCAATCAGTGGTAAAGAACGTGCTAGGACTGTTGGTGGTGTTTCATTGTTGTCTA
CTATGACTACTAGACAGTTTCATCAGAAACACCTTAAGTCCATTGTTAATACACGTAATGCA
TCTGTTGTCATTGGTACCACAAAATTTTATGGTGGTTGGGACAACATGCTTAAGAACCTTAT
TGATGGTGTTGACAATGCGTGCCTTATGGGTTGGGATTACCCTAAGTGTGATCGTGCACTTC
CTAATATGATTCGCATGATTTCTGCCATGATTTTGGGTTCGAAGCATGTTACTTGCTGTGATT
CTTCTGATAAATATTACCGTCTCTGTAATGAGCTCGCTCAAGTGTTGACAGAAGTTGTATAT
TCTAATGGTGGGTTTTATCTCAAGCCTGGTGGTACCACTTCTGGCGATGCTACAACGGCTTA
TGCGAACTCTGTGTTTAATATTTTTCAAGCTGTTAGCGCCAATATTAACAGGTTGCTTAGCAT
TGATAGCAATGTGTGTAATAATATTGGTGTTAAGAGTTTGCAGCGTAAGCTATATGATTGTT
GTTATAGATCTTCTTCAGTAGATGATTCTTTTGTAGACAGTTATTATTGCTACTTGCGGAAAC
ATTTTTCTATGATGATTTTGTCAGATGACGGTGTTGTGTGTTATAATAAAGATTATGCTGATT
TGGGTTATGTGGCAGATATTAATGCCTTTAAAGCTACCCTATATTATCAGAATAATGTCTTT
ATGAGTACTGCTAAATGCTGGGTGGAGCCAGACATTAGCAAAGGTCCTCATGAGTTCTGTT

CACAGCATACTATGCAGATTGTAGATTCTGACGGCAAGTATTATTTGCCATACCCAGACCC
ATCTAGAATATTGTCTGCTGGTGTATTTGTCGATGATGTTGTTAAGACTGACGCTGTGATATT
GTTGGAGCGCTACGTCTCATTGGCAATCGACGCATATCCTTTGTCTAAACATCATAATCCTG
AATATAGAAAAGTGTTTTATACGCTTCTAGATTGGGTTAAGCATCTACACAACACACTTAAT
CAGGGCGTGCTTGAGTCTTTCTCCGTTACATTGCTTGAGGATGTATCCTCTAAGTTTTGGGAT
GAGAGTTTTTATGCTAGTTTGTATGAAAAGTCTACAGCACTTCAAGCCGCAGGCCTATGTGT
TGTTTGTGGTTCTCAAACTGTACTGCGTTGTGGTGATTGTCTTAGACGACCTATGTTGTGCAC
TAAGTGTGCGTATGACCATGTTGTCACCACGAATCATAAATTCATTCTGGCTATAACACCTT
ACGTTTGTAATGCTTCTGGTTGCACCGTTAATGATGTGACTAAGTTGTATTTAGGAGGTCTG
AGTTACTGGTGTATAGACCATAAACCTACGCTGTCACTGCCACTTTGTAGTGCAGGTAATGT
TTTTGGTTTGTATAAAAACTCTGCTACTGGTTCACCGGATGTAGAGGTTTTTAATACACTTGC
CACGTCAGATTGGACTGACGTTAAAGATTACAAGCTGGCTAATGAATGCAAGGATTCTCTT
AGGCTTTTTGCGGCTGAGACTGTTAAAGCCAAAGAAGAGAGTGTTAAATCCTCTTATGCTTG
TGCTACACTTAAAGAAGTTGTTGGGCCACGTGAGTTGATTCTTAGCTGGGAGTGCGGAAAA
ATTAAACCGCCACTTAACCGTAATTCAGTGTTTACGTGTTTCCAGATTTCTAAAGACTCTAA
GTTCCAGGTTGGAGAGTTTACGTTTGAAAAATTGGATTACGGTTCTGATGCTGTGTGCTACA
AGTCTACCACCACAGTCAAATTAGTGCCTGGTATGATTTTTGTTCTCACATCACACAATGTA
CAGCCGCTTAGGGCTCCCACTATTGCTAATCAAGAACGCTATGCTTCAATTTATAAACTGCA
CCCTACTTTTAATATTAGTGATGCTTATGCCAATTTGGTGCCGTACTACCAAATGATTGGCA
GACAACGAATCACTACTATTCAAGGCCCACCGGGAAGTGGCAAGTCTCATTGTGTTATAGG
TCTGGGTCTTTATTACCCAGGTGCTAGGATGGTTTTTGCGGCTTGCTCTCATGCAGCCGTAGA
TTCACTTTGTGTCAAGGCTGCCACTGCTTATAGTGTAGACAAATGCACTCGTATTATCCCTG
CCCGTGCTAGGGTTGAGTGTTATAGTGGGTTTAAGGTAAATAACACTCAGGCACAGTACAT
CTTTTCAACAGTCAACGCACTCCCTGAGTGTAATGTTGACATAGTTGTTATAGATGAAGTGT
CTATGTGTACTAATTATGACCTGTCCGTTATTAATCAGCGGTTGTCTTATAAACATATTGTTT
ATGTGGGTGACCCACAGCAATTACCAGCGCCACGTACCATGATCACTAAAGGCGTTCTCGA
ACCTAAGGACTATAATGTTGTAACTCAGAGGATGTGTGCTGTTGGGCCAGATGTCTTCTTGC
ATAAATGTTACAGATGTCCTGCGGAGATTGTTAAAACAGTCTCAGAGTTGGTTTATGAGAA
TAAGTTTGTACCTGTTCATGCTGATAGCAAACAGTGCTTTAAACTGTTTTGTAAGGGTAATG
TTCAGGTTGACAATGGTTCTAGTATTAACAGAAAGCAATTGGATATCGTTAAGATGTTCCTT
GCTAAGAATCCACGTTGGTCTAAAGCTGTGTTTATCTCACCTTATAATAGTCAAAATTATGT
GGCTAGTAGGGTTTTGGGTTTGCAGATTCAGACTGTTGATTCCTCTCAAGGTAGTGAATATG
ACTATGTCATATACACACAGACGTCAGACACAGCACATGCTTGCAATGTTAATAGATTTAA
TGTTGCAATTACAAGAGCCAAGAAGGGTATTCTTTGTGTTATGTGTGATCGGGATTTGTTTG
ATGCTCTGAAATTTTTCGAGATCAAGCTTTCTGACTTGCAGGCTAGTGGTGATGGCTGTGGT
CTTTTTAAAGATTGTTACAGGAAAGATGATGAATTACCTCCATCTCATGCAGCTACCTATAT
GGCCTTGTCTGACAACTTTAAGGTCAATGGTGATCTTGCTGTGCAGATTGGCTTTAAAGGTG
TGTGTAAGTATGAGCATGTTATTTCTTTTATGGGCTTCAGGTTTGACATTAATGTGCCTGAAC
AGCATAGTCTTTTCTGTACCAGGAATTTTGCAATGCGCCATGTGCGTGGTTGGCTTGGTATG
GATGTAGAAGGTGCCCATGTTACAGGTGAAAATGTAGGTACTAATGTACCATTACAAGTAG
GCTTCTCTAATGGTGTTGATTTTGTTGTCCAACCAGAAGGTTGTGTTGTCACTGAGACAGGC
AGCAGGATCGTACCTGTTAGAGCTCGTGCTCCGCCAGGTGAACAGTTCACACATTTGGTCC
CCCTGCTTCGTAAGGGACAACCATGGGCTGTGGTTCGTAAACGCATTGTTCAGATGTGTTGC
GATTACTTGGTGAACCTATCAGACATATTAATCTTTGTTTTGTGGGCTGGTGGTTTGGAGTTG
ACTACCATGAGATATTTTGTTAAAATTGGACCAATCAAAGTTTGTGAGTGCGGCAAAGTGG
CCACTTGTTACAATAGTGTGACTCTTGCATACTATTGTTTCAAGCATGCCTGAGGGTGTGATT
ATTTGTACAATCCTTATGTTATTGACATTCAGCAGTGGGATTATACAGGTTCACT
>SUOHC1004
CAGTTTTCACAGTTTGTACAACTTCTTGTGTGAATAGCATTTCTTACATCATTACACAGAATT
ATTGTGCTATGTATGTATATGCTTTCTTGTATTTTATGGCCACTCGCAATATGCGTTATGCTTG
GATTTGGCATAGTGGCTTTGTGCTTGCCTATTTCACACTGGCACCATGGTGGCTTATTGTTTG
GTATGTCTGTGCTACATGTGTTGGGTTGATTCCTAGTCTTATGAAACTTAAGGTTTCTACGCA

GCTTTTTGAGGGAGACAAGTTTGTCGGCACTTTTGAGAGTGCTGCTGCAGGTACTTTTGTGA
TTGACATGCATTCTTATCAGCGTCTGGTCAATAGTATTGCACCGGAGAAGCTGAAACAGTA
TGCTGCCAGTTACAATCGTTATAAGTATTATAGTGGTAGTGCTAGTGAGGCTGACTACCGTC
TTGCTTGTTTCGCTCACCTTGCAAAGGCTATGTTGGACTATGGTACCAACCATCAAGACATG
CTCTACACTCCTCCCACAGTTTCGTACAATTCTACATTGCAGTCTGGTCTGCGAAAGATGGC
TCAACCATCTGGTGTTGTTGAAAAATGTGTGGTTCGTGTGAGTTATGGTAACATGACATTGA
ATGGTGTTTGGCTTGGTGACACAGTTATTTGTCCTAGACATGTTATTGCGTCATCAACCACTT
CTACCATTGATTATGAACATGAGGCTTCTATTATGCGTCTTCATAATTTTTCTATTGCTGTTG
GTAATGTCTTTTTGGGTGTTGTAGGTGTTTCTGTTAGAGGTTGTAATCTTCATATTAAGGTTA
ACCAATCTAATGTTAACACACCCAATTATAGCTTTAAAACTTTGAAGGCTGGTGATTCTTTC
AACATTTTGGCTTGTTATGATGGTGTACCTGCTGGTGTTTATGGTGTGACACTGCGCACAAA
TAACACTGTGCGTGGTTCATTTATCAATGGAGCTTGTGGTTCACCGGGTTACAATATTGTTG
GAGGTATAGTTGAGTTTTGTTACCTTCATCAGCTTGAACTTGGCAGTGGTTGTCATGTTGGTT
CTAATCTTGAAGGTGTTATGTATGGTGGTTTTGAGGATCAACCTACATTGCAAATTGAAGGC
GCTAGTAATTTAGTTACTGTAAATGTTATTTCGTTTCTTTACGGTGCACTTTTGAATGGTGTTA
CATGGTGGCTTACAAGTGAACGTGTCACCATAGATGCTTATAATGAGTGGGCTCAACAAAA
TGGTTTTACACAGGTTAGTTCTGTGGATTGTTTTACCATTCTTGCAGCCAAGACTGGTGTAGA
CGTACAACGTATTTTAGCGGCTGTACAGCGTTTGCACAAGGGTTTTGGTGGTAAGGCTATCC
TTGGATTCACTTCTCTTACAGATGAGTTCACCACTGCTGAGGTCATACAGCAAATGTATGGT
GTCAGTCTACAGAGTGGCAAGGTTTCCAGTACTTTCAAGAATTTGTTTCTTGTTGGTACTTTC
TTTGTTATGCTGTGGTCTGAGTTGATGATGTTCACACCATTTTTCTGGGTTAGTCCCGGATTT
GTCACTCCAATTTTTGCTATGCTCGTTTTGCTGTCAATTATGTTTACAGGTTTTATTAAACATA
AAGTTTTGTACCTTTATACATTTTTGATTCCAGCAGTTATTGTGACGTCTTGTTGCAATTTTCT
TGTTGAGCTTGCAATTAATTCTTGGCTTGCATCTGTTCTTGATTATCATGCCTCTATTATGAGT
GTTGATATTCAGGGTGTGATTAATGTAGTTATTTGTGCTATTGTGACTATATTGCACACGTAC
AGGTTTGCCAAGGCTGGTTTTGTGACTTATGTTACATATTTTCTGTCGTTGTTTGTGGTATTTT
ATAATTGGTGGTTTCATGGTGATCTGCTGAGTCTGTTTATGATGTTGCTTCTCAATGTGTCAG
ATAGCTGGTATGTTGGTGCTATTGCATACAGAATATCATTACTTGCAGCCACACAATTCCCT
ATGTTGGAACTTGTTGTTGGTAAGGCCAAGGTTGTGATTTTTATTTATGTTGCAATTGGTTTTT
TGTGCTGTATGTATTATGGTGTTCTGTATTGGATTAACAGATTCTGTAAGTTGACACTTGGTG
TATATGACTTTAAAGTCAGTGCTAATGAGTTTAAGTATATGGTTGCTAACGGGTTACGTGCT
CCTAGAGGTGTGTTTGACTCAATGCTTCTGTCTATGAAACTTGTTGGTATTGGTGGTGATAA
AGTTATAAAAGTTTCCACGGTACAATCCAAACTGACAGATATTAAGTGCACTAATGTTGTTT
TGCTTGGCTGTTTGTCTAGTATGAACATTGCAGCTAATACTAAAGAATGGGCTCATTGTGTC
GAATTGCACAACAAGATTAATCTTTGTGATGATCCTGAAAAGGCACAAGAAATGCTTCTTG
CATTGCTAGCTTTCTTTATTAGCAAGCAAAAAGATTTTGGCATTGATGATTTGATTGATTCAT
ACTTTGACAGTTCTAGTGTACTGCAGAGTGTTGCGTCTACATTCGTCAATATGCCTTCTTACA
TCGCTTATGAAAGTGCGCGGCAAAGTTATGAGGATGCTGTTAATAATGGTTCCTCTCCACAG
CTCATTAAGCAGCTTAAGCGTGCTATGAATATTGCTAAAGCTGAGTTTGACCATGAGTCTTC
GGTTCAAAAGAAGATTAATCGCATGGCCGAACAAGCTGCTGCTCAGATGTTCAAGGAAGC
ACGTGCTGTTAATAGAAAGTCCAAGATTGTTAGTGCTATGCACTCTTTGCTTTTTAGCATGCT
TCGTCGCTTGGACATGTCTAGTGTGGACACTATTCTTAGTCTTGCAAAAGATGGTATAGTTC
CTCTGTCGGTGATTCCCGCCACTTGTGCTACTAAACTTTCTATTGTTAGCAGTGATTTTACGT
CATTTGCTAAGATTGTTAAAGATGGTTGTGTGCACTATGCTGGTGTGGTGTGGTCGATTGCT
GAAATCACTGATGCTGATGGTAAGATCGTACATCTTAAGGAGGTTGTCGAAGAGAACACA
GATTCACTTACATGGCCACTCTTTGTTAGTTGCGTGCGTGTTGTGAAGCTTCAGAATAATGA
GATTATTCCTGGTAAGCTTAAACAGCGTGTTGTAAAGGCAGAGGGTGATGGTTTCAGTGTTG
AAGGTAAAGCACTCTTTAATAATGAGGGTGGTAAAACTTTTATGTACGCTTTTGTCGCTGAC
AAACCAGATTTGAAATATGTTAAGTGGGAATTTGATGGTGGTTGTAACACTATTGAACTTGA
GCCACCTTGTAGGTTTGCTGTTGAAACTGCAAATGGCACTGTTATAAAGTACTTGTATTTTGT
GCGTAATCTAAACACATTGCGCAGGGGTGCAGTTTTGGGCTTTATTGGTGCTACAGTGCGTT
TGCAAGCCGGTAAACAAACAGAACTTGCTTCTAATTCGGCATTGCTTACTATGTGTGCTTTT

GCTGTTGATCCTGCCAAGACGTATATTGACGCTGTTAAGAAGGGTGTTAAACCTGTCGGGA
ATTGCGTCAAGATGTTGTCAAATGGTTCAGGTACTGGCCAAGCTATTACTGTTGGTGTTGAA
GCTAATTCTAATCAAGATTCTTATGGTGGCGCTTCTGTCTGTTTGTATTGCCGTGCACATGTT
GAACATCCTACGATGGATGGTGCGTGTCGTTACAAAGGCAAGTATGTTCAAGTCCCACTTG
GTAATGCGGATCCTATACGGTTCTGTTTAGAAAATGAGCCATGTAAAGTTTGTATGTGCTGG
TTGAATAATGGTTGCACTTGTGATAGAACTTCTATTCAGAGTGTTGATACGAGTTATTTAAA
CGAGCACGGGGCTCTAGTGCAGCTCGACTAGAGCCCTGTAATGGAACTGACACGGATCGT
GTAGTCCGTGCTTTTGACATTTACAATAAGGATGTTGCGTGTATTGGCAAGTTTCTTAAGGTC
AATTGTGTCCGTTTTAAGAATCTTGACAAGCATGATGCCTTCTTTGTTGTAAAACGTTGTACG
AAGTCTGTCATGGAACACGAGCAGTCCATATATGACAAGCTTCACAGTTGTGGTGCAGTTG
CAGTTCATGATTTTTTCTTGTGGAAAGATGGCCGTTCAGTTTTTGGTAATATTAGCAGGCAA
AACCTGACTAAGTATACAATGATGGACTTGTGTTATGCTTTGAGAAATTTTGATGAGCGTAA
TTGTGAAACACTTAAAGAAATTCTTGTCCTTACTGGTTGCTGTGACTATTCATATTTTGAAAA
TAAGGATTGGTACGACCCAGTTGAAAATGAGGACATTCATAAAGTTTATGCACACCTTGGA
ACTGTTGTCTCTAATGCATTGCTTAAGTGCGTTAAGCTTTGCGATGCGATGGTAGAGGCAGG
TGTAGTGGGTGTGTTAACACTTGATAACCAAGACCTTAATGGTGATTTTTATGATTTTGGTG
ACTTTGCTACAACAATACCTAAAATGGGTGTACCATTATGTACCTCTTATTACTCTTATATGA
TGCCTGTAATGGGTATGACCAATTGTCTTGCATCTGAGTGTTTTATTAAGAGTGACATTTTTG
GTAGTGATTTTAAGACCTATGATTTGCTTGAGTACGACTTTACAGAGCACAAAGAGGCTTTG
TTTCGTAAGTACTTCAAGTATTGGGGTCAGGATTACCACCCTAATTGTGCTGATTGTCACGA
CGAGCTTTGTATTGTTCATTGTGCGAATTTTAATACGTTGTTCGCTACGACTATTCCTAATAC
GGCTTTTGGTCCTCTGTGTCGCAAAGTGTTTGTTGATGGTGTGCCACTTGTTACTACAGCTGG
CTACCATTTCAAGCAGTTGGGCCTTGTTTGGAATAAGGATATAAATACTCACTCTACTCGTT
TGAGTGTAAATGAGTTGTTACAGTTTGTCACAGACCCTGCACTTTTGATAGCATCATCACCA
GCCCTTGTGGATCAGCGTACTGTTTGTTTTTCAGTCGCTGCCTTGAGTACTGGTATGACAAAT
CAGACAGTTAAGCCAGGCCACTTTAATAAAGAGTTCTACGACTTTTTGAGATCGCAAGGAT
TTTTTGAAGAAGGCTCTGAATTAACTTTAAAGCATTTCTTCTTTGCACAGAAGGGTGATGCT
GCTGTCAAAGATTTTGACTACTATCGTTATAATAGACCAACTATGCTAGACATTTGTCAGGC
TAGAGTCACTTATAAAATAGTGCAGCGTTATTTTGAGTGCTATGAGGGTGGCTGTATTGCCG
CTAAGGATGTTGTTGTTACTAACCTCAATAAAAGTGCAGGTTACCCACTTAATAAATTTGGT
AAAGCTGGTTTGTATTATGAGTCTCTTAGTTATGAGGAACAAGATGCACTATACGCTCTTAC
AAAGCGTAATGTGTTACCTACTATGACACAGCTTAACCTTAAATATGCAATCAGTGGTAAA
GAACGTGCTAGGACTGTTGGTGGTGTTTCATTGTTGTCTACTATGACTACTAGACAGTTTCAT
CAGAAACACCTTAAGTCCATTGTTAATACACGTAATGCATCTGTTGTCATTGGTACCACAAA
ATTTTATGGTGGTTGGGACAACATGCTTAAGAACCTTATTGATGGTGTTGACAATGCGTGCC
TTATGGGTTGGGATTACCCTAAGTGTGATCGTGCACTTCCTAATATGATTCGCATGATTTCTG
CCATGATTTTGGGTTCGAAGCATGTTACTTGCTGTGATTCTTCTGATAAATATTACCGTCTCT
GTAATGAGCTCGCTCAAGTGTTGACAGAAGTTGTATATTCTAATGGTGGGTTTTATCTCAAG
CCTGGTGGTACCACTTCTGGCGATGCTACAACGGCTTATGCGAACTCTGTGTTTAATATTTTT
CAAGCTGTTAGCGCCAATATTAACAGGTTGCTTAGCATTGATAGCAATGTGTGTAATAATAT
TGGTGTTAAGAGTTTGCAGCGTAAGCTATATGATTGTTGTTATAGATCTTCTTCAGTAGATG
ATTCTTTTGTAGACAGTTATTATTGCTACTTGCGGAAACATTTTTCTATGATGATTTTGTCAG
ATGACGGTGTTGTGTGTTATAATAAAGATTATGCTGATTTGGGTTATGTGGCAGATATTAAT
GCCTTTAAAGCTACCCTATATTATCAGAATAATGTCTTTATGAGTACTGCTAAATGCTGGGT
GGAGCCAGACATTAGCAAAGGTCCTCATGAGTTCTGTTCACAGCATACTATGCAGATTGTA
GATTCTGACGGCAAGTATTATTTGCCATACCCAGACCCATCTAGAATATTGTCTGCTGGTGT
ATTTGTCGATGATGTTGTTAAGACTGACGCTGTGATATTGTTGGAGCGCTACGTCTCATTGG
CAATCGACGCATATCCTTTGTCTAAACATCATAATCCTGAATATAGAAAAGTGTTTTATACG
CTTCTAGATTGGGTTAAGCATCTACACAACACACTTAATCAGGGCGTGCTTGAGTCTTTCTC
CGTTACATTGCTTGAGGATGTATCCTCTAAGTTTTGGGATGAGAGTTTTTATGCTAGTTTGTA
TGAAAAGTCTACAGCACTTCAAGCCGCAGGCCTATGTGTTGTTTGTGGTTCTCAAACTGTAC
TGCGTTGTGGTGATTGTCTTAGACGACCTATGTTGTGCACTAAGTGTGCGTATGACCATGTT

GTCACCACGAATCATAAATTCATTCTGGCTATAACACCTTACGTTTGTAATGCTTCTGGTTGC
ACCGTTAATGATGTGACTAAGTTGTATTTAGGAGGTCTGAGTTACTGGTGTATAGACCATAA
ACCTACGCTGTCACTGCCACTTTGTAGTGCAGGTAATGTTTTTGGTTTGTATAAAAACTCTGC
TACTGGTTCACCGGATGTAGAGGTTTTTAATACACTTGCCACGTCAGATTGGACTGACGTTA
AAGATTACAAGCTGGCTAATGAATGCAAGGATTCTCTTAGGCTTTTTGCGGCTGAGACTGTT
AAAGCCAAAGAAGAGAGTGTTAAATCCTCTTATGCTTGTGCTACACTTAAAGAAGTTGTTG
GGCCACGTGAGTTGATTCTTAGCTGGGAGTGCGGAAAAATTAAACCGCCACTTAACCGTAA
TTCAGTGTTTACGTGTTTCCAGATTTCTAAAGACTCTAAGTTCCAGGTTGGAGAGTTTACGTT
TGAAAAATTGGATTACGGTTCTGATGCTGTGTGCTACAAGTCTACCACCACAGTCAAATTA
GTGCCTGGTATGATTTTTGTTCTCACATCACACAATGTACAGCCGCTTAGGGCTCCCACTATT
GCTAATCAAGAACGCTATGCTTCAATTTATAAACTGCACCCTACTTTTAATATTAGTGATGC
TTATGCCAATTTGGTGCCGTACTACCAAATGATTGGCAGACAACGAATCACTACTATTCAA
GGCCCACCGGGAAGTGGCAAGTCTCATTGTGTTATAGGTCTGGGTCTTTATTACCCAGGTGC
TAGGATGGTTTTTGCGGCTTGCTCTCATGCAGCCGTAGATTCACTTTGTGTCAAGGCTGCCA
CTGCTTATAGTGTAGACAAATGCACTCGTATTATCCCTGCCCGTGCTAGGGTTGAGTGTTAT
AGTGGGTTTAAGGTAAATAACACTCAGGCACAGTACATCTTTTCAACAGTCAACGCACTCC
CTGAGTGTAATGTTGACATAGTTGTTATAGATGAAGTGTCTATGTGTACTAATTATGACCTG
TCCGTTATTAATCAGCGGTTGTCTTATAAACATATTGTTTATGTGGGTGACCCACAGCAATT
ACCAGCGCCACGTACCATGATCACTAAAGGCGTTCTCGAACCTAAGGACTATAATGTTGTA
ACTCAGAGGATGTGTGCTGTTGGGCCAGATGTCTTCTTGCATAAATGTTACAGATGTCCTGC
GGAGATTGTTAAAACAGTCTCAGAGTTGGTTTATGAGAATAAGTTTGTACCTGTTCATGCTG
ATAGCAAACAGTGCTTTAAACTGTTTTGTAAGGGTAATGTTCAGGTTGACAATGGTTCTAGT
ATTAACAGAAAGCAATTGGATATCGTTAAGATGTTCCTTGCTAAGAATCCACGTTGGTCTA
AAGCTGTGTTTATCTCACCTTATAATAGTCAAAATTATGTGGCTAGTAGGGTTTTGGGTTTGC
AGATTCAGACTGTTGATTCCTCTCAAGGTAGTGAATATGACTATGTCATATACACACAGAC
GTCAGACACAGCACATGCTTGCAATGTTAATAGATTTAATGTTGCAATTACAAGAGCCAAG
AAGGGTATTCTTTGTGTTATGTGTGATCGGGATTTGTTTGATGCTCTGAAATTTTTCGAGATC
AAGCTTTCTGACTTGCAGGCTAGTGGTGATGGCTGTGGTCTTTTTAAAGATTGTTACAGGAA
AGATGATGAATTACCTCCATCTCATGCAGCTACCTATATGGCCTTGTCTGACAACTTTAAGG
TCAATGGTGATCTTGCTGTGCAGATTGGCTTTAAAGGTGTGTGTAAGTATGAGCATGTTATTT
CTTTTATGGGCTTCAGGTTTGACATTAATGTGCCTGAACAGCATAGTCTTTTCTGTACCAGGA
ATTTTGCAATGCGCCATGTGCGTGGTTGGCTTGGTATGGATGTAGAAGGTGCCCATGTTACA
GGTGAAAATGTAGGTACTAATGTACCATTACAAGTAGGCTTCTCTAATGGTGTTGATTTTGT
TGTCCAACCAGAAGGTTGTGTTGTCACTGAGACAGGCAGCAGGATCGTACCTGTTAGAGCT
CGTGCTCCGCCAGGTGAACAGTTCACACATTTGGTCCCCCTGCTTCGTAAGGGACAACCAT
GGGCTGTGGTTCGTAAACGCATTGTTCAGATGTGTTGCGATTACTTGGTGAACCTATCAGAC
ATATTAATCTTTGTTTTGTGGGCTGGTGGTTTGGAGTTGACTACCATGAGATATTTTGTTAAA
ATTGGACCAATCAAAGTTTGTGAGTGCGGCAAAGTGGCCACTTGTTACAATAGTGTGACTC
ATGCATACTATTGTTTCAAGCATGCCTTAGGGTGTGATTATTTGTACAATCCTTATGTTATTG
ACATTCAGCAGTGGGATTATACAGGTTCACTTAGCCATAATCATCATGAGCATTGTAATGTA
CATCGCAATGAGCATGTTGCATCAGGTGATGCTATTATGACTCGTTGTCTTGCTGTGTATGAT
TGCTTCGTCAAGAACGTTGATTGGTCCATTACTTACCCATTCATTGCTAATGAACATGCCATT
AATAAGAGTGGTAGAGTTGTGCAATCTATGGTTGTCAAATCAGCTATAAAGTTATATAATC
CTAAAGTAATTCATGATATTGGTAATCCTAAAGGTATTAGGTGTGCAGTTACTGATGCACAC
TGGTTTTGCTATGATAAGCAGCCCCTCAATTCCAATGTCAAGTTGCTTGAGTATGATTATAT
AACTCATGGTCAGATGGATGGGTTATGCCTATTTTGGAATTGCAATGTTGATATGTATCCTG
AATTTTCTATTGTGTGCCGTTTTGACACTCGTTGTAGGTCAAATCTCAACTTAGAAGGTGTTA
ATGGAGGTTCACTTTATGTCAACAACCATGCATTTTTTACACCTGCATTTGATAAGCGTGCA
ATGGCTAAACTTAAGCCTATGCCCTTCTTTTTCTATGATGACACGCCTTGCGATCGTGTTCAT
GATCAAATAAATTATGTACCACTGCGTGCTAGTACTTGTATTACAAAGTGTAATATTGGTGG
TGCTGTTTGCAGTAAGCATGCCAATATGTTTCATGCCTATGTTAATGCATATAATACTTTCAC
GCAGGCTGGTTTTACTATTTGGGTCCCTGTAACTTTTGATTTGTTCAACCTTTGGCAAACATT

TGTCGAAACTAATTTGCAAGGCTTGGAGAACATAGCGTTTAATGTCCTCAAGAAAGGGTCT
TTTGTTGGTGTAGAGGGTGAACTACCGGTTGCTATCGTTAATGACCGTGTGCTGGTACGTGA
TGGCACCGTGGATAATTTGATTTTTACTAATAAAACCTCATTACCTACTAATGTTGCTTTTGA
GCTTTACGCCAAACGTAAAATGGGTTTGACACCACCTCTTGCTGTTCTCAGGAATCTCGGTG
TTGTTAGTACCTATAAGTTTGTGTTATGGGATTATGAGGCAGAAAGACCATTTACTACTTTTA
CTAAAAATGTTTGCAGTTACACAGACTTTGATGAGGATGTGTGCACTTGCTTTGATAATAGC
ATTCAGGGTTCGTTTGAACGCTTTTCAATGGCAAACAATGCAGTGCTTATTTCCACATCTGC
TGTTAAGAAGCTTACTTGTATTAAGCTTAACTATGGTTTGCTTAATGGTGTGGCTGTTTCTAA
GACTGGTGACAAGCCTGTTACATGGTATATCTATGTCCGTAAAAATGGTCAATTTGTTGACC
AATTTGACGGATATTTTACTCAGGGGCGTACTACTATTGATTTCTTACCTCGTAGTGTTATGG
AGGAAGACTTTTTAAACCTTGATATGGGTTTGTTTATTAGTAAGTATGGTCTTGAAGACTAT
GCTTTTGAGCATGTTGTGTATGGTGACGTGTCTGGTACTACACTTGGTGGACTACATTTATTA
ATTTCTCAAGTACGCCTTGGAAAAATGGGTGTCTTGAAAGTGGATGAGTTCACACATAGTG
AGGATAGTACATTGAAGTCCTGTACAGTCACCTATTCTAATGATCCTAGTAGTAAAATGGTT
TGTACCTATATGGATTTGTTGCTAGATGATTTTGTTACCATTTTAAAATCTTTGGATTTGGGTG
TAATTTCCAGAGTTCATGAAGTTGTAGTCGATTGTAAGGTTTGGCGTTGGATGTTGTGGTGT
AAGGACCACAAAGTTCAAACGTTCTACCCACAGTTGCAGTCGTCAGAATGGAAATGTGGCT
ATTCCATGCCCTCACTGTATAAAATACAGCGTATGTGTCTTGAGCCCTGTAATTTGTACAAT
TATGGTGCTAGTCTTAAGCTACCTGAAGGTATCATGTTTAACGTGGTCAAATACACTCAGTT
ATGTCAATATTTGAATAGTACTACTATGTGTGTACCGCATAATATGCGTGTACTACATCTTG
GTGCTGGCTCAGATAAAGGAGTCGCACCTGGTACTGCTGTATTGCGTCGTTGGTTACCCAGT
GATGCTATTATTGTTGACAATGATGTTAATGACTATGTTAGTGATGCAGATTTCAGTGTAAC
AGGGGATTGCGCCACTTTGTACTTACAGGATAAGTTCGACTTAGTGGTTTCAGACATGTATG
ATGGCCGTATTAAACAGTGTGATGGTGTTAACGTTTCAAAGGATGGCTTCTTTCCTTACATC
AATGGTGTGATATCAGAGAAGCTTTCCCTAGGTGGTACAGTTGCCATTAAGATTACTGAGT
ATAGCTGGAATAAGCGGCTGTATGAGTTGATACAAAAGTTTGAATTTTGGACGTTGTTTTGT
ACTAGTGTGAATACATCATCGTCTGAAGCTTTTCTAATTGGTGTCAACTATCTTGGCGACTA
CGCCACTCAACTCATTGTGGATGGTAATACTATGCACGCTAATTATGTGTTTTGGCGTAATT
CTACAATTATGTCCATGTCATATAATAGTGTGCTTGATCTAGCAAAGTTTAATTGTAGACAT
AAAGCTACGGTTGTTATTTCACTTAAAGAAAGTGATTTGAATGATGTTGTGGTAGGCCTTAT
AAAGAATGGAAAGTTGTTGGTTAGAAAGAGTGGTGTCATTTGTGACTATGGTAACCATTTA
GTTTCAACTAAATAA
>SUOHC1005-ON893138
ATGCTGGAACCATAGAGCGTGGGCACTATACAGTGTTCGATGATGACACTGGACGTGTCCA
TGATGGCAGTGATTTTGCACCCGGAGACCTTTCCACCATGGCCGTTACTTCGCTCGTTGTGC
GTAATCGTGCATTTTTGGACCCGGTTGCTCGCGTCGAGCGTAAAGCTACTGAATTTGTGCAG
ACATTAGATGCAGCTTCTGATCAATTCTTTGCCTTTGGTGATGTTGTGTCGCGGAATGTTATT
ACAGTGCTTTTGTGGCTTTTTAGTATGCTTAGTATAGTTTTTAAGGCCTTTAAGAGTAAAGAT
TTAAAAGTTTTTGCTTTAGCCCCTGAGAGAACTGGTGTTATACTAAGTCGTAGTTTACGCTAT
AATTTTAAAGCTTGTAAGCACGTTTTTAAGCTTAAGATGCGTTGGGTCACATTGTTTTTCAAA
TTTTTAGTTCTGCTGTACACATTGTATGCAGTAGGTTTCTTGTTTGTACGGTTTGGTCCTGTTA
ACGGTTATGTCTGTGGTGACTACGTTTTGGGTTATGCAGAATCAAACTTCGATAAGAATTCA
TATTGCAATTCTCCGCTGTGTAAAGTGTGCTTGTATGGCTACCAGGAATTGAGTGACTTTCCT
CATACTAACACTGTTTGGGAGCATATTAAAGACCCACTTATTGGGAATGTTATGCCCTTGCT
TTACTTAGTCTTTCTAGCAATTTTTGGTGGTCTTCCGATGAGAATTATTGTGTGCTATTTTGTT
GCACAGTATGTAAACTTTTTTGGTAGCAAATTTGGTGTTCAAGACAATGTTTGGCTTCTACA
GGTCATTCCATTCTCAGTGTTTGGTGATGAAATTGTGGTTTGTTTCATTGTTTTTAAGGCACTT
TGCTTTTTGCGACATGTCCTCTTAGGCTGTGATAAAGCTTCTTGTGTTGCTTGTTGTAAAAGT
GCGCGTCTTCGTCGTGTACCTATGCAGACTATTGTGAATGGAGCTTCAAAATCATTTTATGTT
ATGGCTAATGGCGGTAAGAGTTTTTGTAAGAAGCATAACTTCTTCTGTCTTAGTTGTGACTCT
TATGGTCCTGGTAATACTTTTATTAATGAGGTAATTGCCAAGGAAGTGTCCAATGTGACTAA
AACTAATGTTCAACCTACCGGTACAGCTTGTGTAGACATTGATAAGGTTGAGTTTTCTAATG

GGTTTTACTATTTGTATAGTGGTGAGACATTTTGGAAATACAATTTTGACATTACTGAAGCT
AAATATAGCTGTAAAGATGTCCTTAAGAATTGTAACATTGTGTCTGATTTTATTGTTTACAAT
AATGTTGGTAGTAATGTGACACAGGTTCGTAATGCTTGTGTTTACTTATCTCAACTTTTGTGT
AAACCTATTAAACTTGTAGACTCAGCTCTCCTTGCCACGTTGAACGTAGACTTTAATGGTGC
TCTGCATGCAGCTTTTGTGGAAGTGCTTAATGACAGTTTTTCTAAGGACTTGTCGGGTTGCAC
TACTATGGCTGAGTGTAAGCAGGCACTAGGTATTGATGTTAGTGATGATGATTTTGTTAATG
CTGTTAGTAACGCACACCGTTTTAACGTGTTGCTTACAGATGTTTCATTTAACAATTTTGTCA
CATCCTATGCCAAACCAGAGGAGAAGTTGTCTACTCATGATCTTGCGACTTGTATGCGTGCT
GGTGCTAAGGTTGTCAATCATAATGTTCTCGTAAAAGAGAATATGCCTGTTGTGTGGGCTGC
TAGAGAATTTAACTTTTTATCTGAAGAATGTCGTAAGTATTTGATTAAGACGTCAAAAGCTA
AGGGCGTCAATTTCATGCTTACATTTAACGACAATAAGATGCAAACACACGTCCCCGTTGT
TAGCATTATAGCTAAGCAAGGGGCTGGTGTTTTGCCCACTTGTTACAAATTTTTGTGGTGGTT
GTGTTTCTTTGTTGTTGTTGTGTTCTTTAGTACTGGTTTATTGTCCTTTGAGCAACAGGTTACT
AGTGAGACTGATTTTGACTTTAAGTACATTGAGAATGGTGTGCTTAAAGTGTTCACAGAATC
CTTGGATTGTGTGCATAATGTCTTCGACAATTTTGTTACATGGCATGACGCAAAATTCGGTT
CGGTGCCAAGGGTTGATGCCAGGTGTCCTATTGTCGTAGGTGTATCTGATGATGTGCGTATG
ATTCCTGGTATTGCCGCTGGTGTTAAGCTCGTTGGTAAGACATTGGTTTTTGCACTTCAATCT
GTGTTTGGTGATTCTGGTTTATGTTATGACCTTAATGGTTTGACAACCACTGCTAGTTGTGTTT
TTAATTCGGCTTGTACTATTCTTAGTGGCGTTGGAGGTAAACACACCTATTGTTACAAGGAT
GGTCTTGTGTCTGATTCAAAGTTGTATTCTGATATCTTGCCTAATAGTCATTATAAACTTGAA
GATGGCAATTATATTAAATTGCCAGAAGTCTTGACTAGGGGTTTTGGCTTCAGAACAATTCG
CACCATTGAAACCACATATTGCCGTATGGGTCAGTGTATTGATTCTAGAGCTGGTGTGTGTT
TTGGTGTAGACCGTTTCTTTGTATACAGTGCTGAAACTGGTCCAGATTTTATCTGTGGTACAG
GCCTCATTTCACTGTTGTATAATATCTTTAACATTTTTTCAGGTTCTTTCAGTGTGATGGCTTT
GTCTGGTCAAATCATTTTTAATTTAGTTGTAGCAACTTTAGTGGTGTTTAGCTGTTTTATGGTT
ACCAAATTCAAACGTGTCTTTGGTGACCTTTCTTTTGCAGTTTTCACAGTTTGTACAACTTCT
TGTGTGAATAGCATTTCTTACATCATTACACAGAATTATTGTGCTATGTATGTATATGCTTTC
TTGTATTTTATGGCCACTCGCAATATGCGTTATGCTTGGATTTGGCATAGTGGCTTTGTGCTT
GCCTATTTCACACTGGCACCATGGTGGCTTATTGTTTGGTATGTCTGTGCTACATGTGTTGGG
TTGATTCCTAGTCTTATGAAACTTAAGGTTTCTACGCAGCTTTTTGAGGGAGACAAGTTTGTC
GGCACTTTTGAGAGTGCTGCTGCAGGTACTTTTGTGATTGACATGCATTCTTATCAGCGTCTG
GTCAATAGTATTGCACCGGAGAAGCTGAAACAGTATGCTGCCAGTTACAATCGTTATAAGT
ACTATAGTGGTAGTGCTAGTGAGGCTGACTACCGTCTTGCTTGTTTCGCTCACCTTGCAAAG
GCTATGTTGGACTATGGTACCAACCATCAAGACATGCTCTACACTCCTCCCACAGTTTCGTA
CAATTCTACATTGCAGTCTGGTCTGCGAAAGATGGCTCAACCATCTGGTGTTGTTGAAAAAT
GTGTGGTTCGTGTGAGTTATGGTAACATGACATTGAATGGTGTTTGGCTTGGTGACACAGTT
ATTTGTCCTAGACATGTTATTGCGTCATCAACCACTTCTACCATTGATTATGAACATGAGGC
TTCTATTATGCGTCTTCATAATTTTTCTATTGCTGTTGGTAATGTCTTTTTGGGTGTTGTAGGT
GTTTCTGTTAGAGGTTGTAATCTTCATATTAAGGTTAACCAATCTAATGTTAACACACCCAA
TTATAGCTTTAAAACTTTGAAGGCTGGTGATTCTTTCAACATTTTGGCTTGTTATGATGGTGT
ACCTGCTGGTGTTTATGGTGTGACACTGCGCACAAATAACACTGTGCGTGGTTCATTTATCA
ATGGAGCTTGTGGTTCACCGGGTTACAATATTGTTGGAGGTATAGTTGAGTTTTGTTACCTTC
ATCAGCTTGAACTTGGCAGTGGTTGTCATGTTGGTTCTAATCTTGAAGGTGTTATGTATGGTG
GTTTTGAGGATCAACCTACATTGCAAATTGAAGGCGCTAGTAATTTAGTTACTGTAAATGTT
ATTTCGTTTCTTTACGGTGCACTTTTGAATGGTGTTACATGGTGGCTTACAAGTGAACGTGTC
ACCATAGATGCTTATAATGAGTGGGCTCAACAAAATGGTTTTACACAGGTTAGTTCTGTGG
ATTGTTTTACCATTCTTGCAGCCAAGACTGGTGTAGACGTACAACGTATTTTAGCGGCTGTA
CAGCGTTTGCACAAGGGTTTTGGTGGTAAGGCTATCCTTGGATTCACTTCTCTTACAGATGA
GTTCACCACTGCTGAGGTCATACAGCAAATGTATGGTGTCAGTCTACAGAGTGGCAAGGTT
TCCAGTACTTTCAAGAATTTGTTTCTTGTTGGTACTTTCTTTGTTATGCTGTGGTCTGAGTTGA
TGATGTTCACACCATTTTTCTGGGTTAGTCCCGGATTTGTCACTCCAATTTTTGCTATGCTCGT
TTTGCTGTCAATTATGTTTACAGGTTTTATTAAACATAAAGTTTTGTACCTTTATACATTTTTG

ATTCCAGCAGTTATTGTGACGTCTTGTTGCAATTTTCTTGTTGAGCTTGCAATTAATTCTTGGC
TTGCATCTGTTCTTGATTATCATGCCTCTATTATGAGTGTTGATATTCAGGGTGTGATTAATG
TAGTTATTTGTGCTATTGTGACTATATTGCACACGTACAGGTTTGCCAAGGCTGGTTTTGTGA
CTTATGTTACATATTTTCTGTCGTTGTTTGTGGTATTTTATAATTGGTGGTTTCATGGTGATCT
GCTGAGTCTGTTTATGATGTTGCTTCTCAATGTGTCAGATAGCTGGTATGTTGGTGCTATTGC
ATACAGAATATCATTACTTGCAGCCACACAATTCCCTATGTTGGAACTTGTTGTTGGTAAGG
CCAAGGTTGTGATTTTTATTTATGTTGCAATTGGTTTTTTGTGCTGTATGTATTATGGTGTTCT
GTATTGGATTAACAGATTCTGTAAGTTGACACTTGGTGTATATGACTTTAAAGTCAGTGCTA
ATGAGTTTAAGTATATGGTTGCTAACGGGTTACGTGCTCCTAGAGGTGTGTTTGACTCAATG
CTTCTGTCTATGAAACTTGTTGGTATTGGTGGTGATAAAGTTATAAAAGTTTCCACGGTACA
ATCCAAACTGACAGATATTAAGTGCACTAATGTTGTTTTGCTTGGCTGTTTGTCTAGTATGA
ACATTGCAGCTAATACTAAAGAATGGGCTCATTGTGTCGAATTGCACAACAAGATTAATCT
TTGTGATGATCCTGAAAAGGCACAAGAAATGCTTCTTGCATTGCTAGCTTTCTTTATTAGCA
AGCAAAAAGATTTTGGCATTGATGATTTGATTGATTCATACTTTGACAGTTCTAGTGTACTG
CAGAGTGTTGCGTCTACATTCGTCAATATGCCTTCTTACATCGCTTATGAAAGTGCGCGGCA
AAGTTATGAGGATGCTGTTAATAATGGTTCCTCTCCACAGCTCATTAAGCAGCTTAAGCGTG
CTATGAATATTGCTAAAGCTGAGTTTGACCATGAGTCTTCGGTTCAAAAGAAGATTAATCGC
ATGGCCGAACAAGCTGCTGCTCAGATGTTCAAGGAAGCACGTGCTGTTAATAGAAAGTCCA
AGATTATTAGTGCTATGCACTCTTTGCTTTTTAGCATGCTTCGTCGCTTGGACATGTCTAGTG
TGGACACTATTCTTAGTCTTGCAAAAGATGGTATAGTTCCTCTGTCGGTGATTCCCGCCACTT
GTGCTACTAAACTTTCTATTGTTAGCAGTGATTTTACGTCATTTGCTAAGATTGTTAAAGATG
GTTGTGTGCACTATGCTGGTGTGGTGTGGTCGATTGCTGAAATCACTGATGCTGATGGTAAG
ATCGTACATCTTAAGGAGGTTGTCGAAGAGAACACAGATTCACTTACATGGCCACTCTTTGT
TAGTTGCGTGCGTGTTGTGAAGCTTCAGAATAATGAGATTATTCCTGGTAAGCTTAAACAGC
GTGTTGTAAAGGCAGAGGGTGATGGTTTCAGTGTTGAAGGTAAAGCACTCTTTAATAATGA
GGGTGGTAAAACTTTTATGTACGCTTTTGTCGCTGACAAACCAGATTTGAAATATGTTAAGT
GGGAATTTGATGGTGGTTGTAACACTATTGAACTTGAGCCACCTTGTAGGTTTGCTGTTGAA
ACTGCAAATGGCACTGTTATAAAGTACTTGTATTTTGTGCGTAATCTAAACACATTGCGCAG
GGGTGCAGTTTTGGGCTTTATTGGTGCTACAGTGCGTTTGCAAGCCGGTAAACAAACAGAA
CTTGCTTCTAATTCGGCATTGCTTACTATGTGTGCTTTTGCTGTTGATCCTGCCAAGACGTAT
ATTGACGCTGTTAAGAAGGGTGTTAAACCTGTCGGGAATTGCGTCAAGATGTTGTCAAATG
GTTCAGGTACTGGCCAAGCTATTACTGTTGGTGTTGAAGCTAATTCTAATCAAGATTCTTAT
GGTGGCGCTTCTGTCTGTTTGTATTGCCGTGCACATGTTGAACATCCTACGATGGATGGTGC
GTGTCGTTACAAAGGCAAGTATGTTCAAGTCCCACTTGGTAATGCGGATCCTATACGGTTCT
GTTTAGAAAATGAGCCATGTAAAGTTTGTATGTGCTGGTTGAATAATGGTTGCACTTGTGAT
AGAACTTCTATTCAGAGTGTTGATACGAGTTATTTAAACGAGCACGGGGCTCTAGTGCAGC
TCGACTAGAGCCCTGTAATGGAACTGACACGGATCGTGTAGTCCGTGCTTTTGACATTTACA
ATAAGGATGTTGCGTGTATTGGCAAGTTTCTTAAGGTCAATTGTGTCCGTTTTAAGAATCTTG
ACAAGCATGATGCCTTCTTTGTTGTAAAACGTTGTACGAAGTCTGTCATGGAACACGAGCA
GTCCATATATGACAAGCTTCACAGTTGTGGTGCAGTTGCAGTTCATGATTTTTTCTTGTGGAA
AGATGGCCGTTCAGTTTTTGGTAATATTAGCAGGCAAAACCTGACTAAGTATACAATGATG
GACTTGTGTTATGCTTTGAGAAATTTTGATGAGCGTAATTGTGAAACACTTAAAGAAATTCT
TGTCCTTACTGGTTGCTGTGACTATTCATATTTTGAAAATAAGGATTGGTACGACCCAGTTG
AAAATGAGGACATTCATAAAGTTTATGCACACCTTGGAACTGTTGTCTCTAATGCATTGCTT
AAGTGCGTTAAGCTTTGCGATGCGATGGTAGAGGCAGGTGTAGTGGGTGTGTTAACACTTG
ATAACCAAGACCTTAATGGTGATTTTTATGATTTTGGTGACTTTGCTACAACAATACCTAAA
ATGGGTGTACCATTATGTACCTCTTATTACTCTTATATGATGCCTGTAATGGGTATGACCAAT
TGTCTTGCATCTGAGTGTTTTATTAAGAGTGACATTTTTGGTAGTGATTTTAAGACCTATGAT
TTGCTTGAGTACGACTTTACAGAGCACAAAGAGGCTTTGTTTCGTAAGTACTTCAAGTATTG
GGGTCAGGATTACCACCCTAATTGTGCTGATTGTCACGACGAGCTTTGTATTGTTCATTGTG
CGAATTTTAATACGTTGTTCGCTACGACTATTCCTAATACGGCTTTTGGTCCTCTGTGTCGCA
AAGTGTTTGTTGATGGTGTGCCACTTGTTACTACAGCTGGCTACCATTTCAAGCAGTTGGGC

CTTGTTTGGAATAAGGATATAAATACTCACTCTACTCGTTTGAGTGTAAATGAGTTGTTACA
GTTTGTCACAGACCCTGCACTTTTGATAGCATCATCACCAGCCCTTGTGGATCAGCGTACTG
TTTGTTTTTCAGTCGCTGCCTTGAGTACTGGTATGACAAATCAGACAGTTAAGCCAGGCCAC
TTTAATAAAGAGTTCTACGACTTTTTGAGATCGCAAGGATTTTTTGAAGAAGGCTCTGAATT
AACTTTAAAGCATTTCTTCTTTGCACAGAAGGGTGATGCTGCTGTCAAAGATTTTGACTACT
ATCGTTATAATAGACCAACTATGCTAGACATTTGTCAGGCTAGAGTCACTTATAAAATAGT
GCAGCGTTATTTTGAGTGCTATGAGGGTGGCTGTATTGCCGCTAAGGATGTTGTTGTTACTA
ACCTCAATAAAAGTGCAGGTTACCCACTTAATAAATTTGGTAAAGCTGGTTTGTATTATGAG
TCTCTTAGTTATGAGGAACAAGATGCACTATACGCTCTTACAAAGCGTAATGTGTTACCTAC
TATGACACAGCTTAACCTTAAATATGCAATCAGTGGTAAAGAACGTGCTAGGACTGTTGGT
GGTGTTTCATTGTTGTCTACTATGACTACTAGACAGTTTCATCAGAAACACCTTAAGTCCATT
GTTAATACACGTAATGCATCTGTTGTCATTGGTACCACAAAATTTTATGGTGGTTGGGACAA
CATGCTTAAGAACCTTATTGATGGTGTTGACAATGCGTGCCTTATGGGTTGGGATTACCCTA
AGTGTGATCGTGCACTTCCTAATATGATTCGCATGATTTCTGCCATGATTTTGGGTTCGAAGC
ATGTTACTTGCTGTGATTCTTCTGATAAATATTACCGTCTCTGTAATGAGCTCGCTCAAGTGT
TGACAGAAGTTGTATATTCTAATGGTGGGTTTTATCTCAAGCCTGGTGGTACCACTTCTGGC
GATGCTACAACGGCTTATGCGAACTCTGTGTTTAATATTTTTCAAGCTGTTAGCGCCAATATT
AACAGGTTGCTTAGCATTGATAGCAATGTGTGTAATAATATTGGTGTTAAGAGTTTGCAGCG
TAAGCTATATGATTGTTGTTATAGATCTTCTTCAGTAGATGATTCTTTTGTAGACAGTTATTA
TTGCTACTTGCGGAAACATTTTTCTATGATGATTTTGTCAGATGACGGTGTTGTGTGTTATAA
TAAAGATTATGCTGATTTGGGTTATGTGGCAGATATTAATGCCTTTAAAGCTACCCTATATT
ATCAGAATAATGTCTTTATGAGTACTGCTAAATGCTGGGTGGAGCCAGACATTAGCAAAGG
TCCTCATGAGTTCTGTTCACAGCATACTATGCAGATTGTAGATTCTGACGGCAAGTATTATTT
GCCATACCCAGACCCATCTAGAATATTGTCTGCTGGTGTATTTGTCGATGATGTTGTTAAGA
CTGACGCTGTGATATTGTTGGAGCGCTACGTCTCATTGGCAATCGACGCATATCCTTTGTCT
AAACATCATAATCCTGAATATAGAAAAGTGTTTTATACGCTTCTAGATTGGGTTAAGCATCT
ACACAACACACTTAATCAGGGCGTGCTTGAGTCTTTCTCCGTTACATTGCTTGAGGATGTAT
CCTCTAAGTTTTGGGATGAGAGTTTTTATGCTAGTTTGTATGAAAAGTCTACAGCACTTCAA
GCCGCAGGCCTATGTGTTGTTTGTGGTTCTCAAACTGTACTGCGTTGTGGTGATTGTCTTAGA
CGACCTATGTTGTGCACTAAGTGTGCGTATGACCATGTTGTCACCACGAATCATAAATTCAT
TCTGGCTATAACACCTTACGTTTGTAATGCTTCTGGTTGCACCGTTAATGATGTGACTAAGTT
GTATTTAGGAGGTCTGAGTTACTGGTGTATAGACCATAAACCTACGCTGTCACTGCCACTTT
GTAGTGCAGGTAATGTTTTTGGTTTGTATAAAAACTCTGCTACTGGTTCACCGGATGTAGAG
GTTTTTAATACACTTGCCACGTCAGATTGGACTGACGTTAAAGATTACAAGCTGGCTAATGA
ATGCAAGGATTCTCTTAGGCTTTTTGCGGCTGAGACTGTTAAAGCCAAAGAAGAGAGTGTT
AAATCCTCTTATGCTTGTGCTACACTTAAAGAAGTTGTTGGGCCACGTGAGTTGATTCTTAG
CTGGGAGTGCGGAAAAATTAAACCGCCACTTAACCGTAATTCAGTGTTTACGTGTTTCCAG
ATTTCTAAAGACTCTAAGTTCCAGGTTGGAGAGTTTACGTTTGAAAAATTGGATTACGGTTC
TGATGCTGTGTGCTACAAGTCTACCACCACAGTCAAATTAGTGCCTGGTATGATTTTTGTTCT
CACATCACACAATGTACAGCCGCTTAGGGCTCCCACTATTGCTAATCAAGAACGCTATGCT
TCAATTTATAAACTGCACCCTACTTTTAATATTAGTGATGCTTATGCCAATTTGGTGCCGTAC
TACCAAATGATTGGCAGACAACGAATCACTACTATTCAAGGCCCACCGGGAAGTGGCAAG
TCTCATTGTGTTATAGGTCTGGGTCTTTATTACCCAGGTGCTAGGATGGTTTTTGCGGCTTGC
TCTCATGCAGCCGTAGATTCACTTTGTGTCAAGGCTGCCACTGCTTATAGTGTAGACAAATG
CACTCGTATTATCCCTGCCCGTGCTAGGGTTGAGTGTTATAGTGGGTTTAAGGTAAATAACA
CTCAGGCACAGTACATCTTTTCAACAGTCAACGCACTCCCTGAGTGTAATGTTGACATAGTT
GTTATAGATGAAGTGTCTATGTGTACTAATTATGACCTGTCCGTTATTAATCAGCGGTTGTCT
TATAAACATATTGTTTATGTGGGTGACCCACAGCAATTACCAGCGCCACGTACCATGATCA
CTAAAGGCGTTCTCGAACCTAAGGACTATAATGTTGTAACTCAGAGGATGTGTGCTGTTGG
GCCAGATGTCTTCTTGCATAAATGTTACAGATGTCCTGCGGAGATTGTTAAAACAGTCTCAG
AGTTGGTTTATGAGAATAAGTTTGTACCTGTTCATGCTGATAGCAAACAGTGCTTTAAACTG
TTTTGTAAGGGTAATGTTCAGGTTGACAATGGTTCTAGTATTAACAGAAAGCAATTGGATAT

CGTTAAGATGTTCCTTGCTAAGAATCCACGTTGGTCTAAAGCTGTGTTTATCTCACCTTATAA
TAGTCAAAATTATGTGGCTAGTAGGGTTTTGGGTTTGCAGATTCAGACTGTTGATTCCTCTCA
AGGTAGTGAATATGACTATGTCATATACACACAGACGTCAGACACAGCACATGCTTGCAAT
GTTAATAGATTTAATGTTGCAATTACAAGAGCCAAGAAGGGTATTCTTTGTGTTATGTGTGA
TCGGGATTTGTTTGATGCTCTGAAATTTTTCGAGATCAAGCTTTCTGACTTGCAGGCTAGTGG
TGATGGCTGTGGTCTTTTTAAAGATTGTTACAGGAAAGATGATGAATTACCTCCATCTCATG
CAGCTACCTATATGGCCTTGTCTGACAACTTTAAGGTCAATGGTGATCTTGCTGTGCAGATT
GGCTTTAAAGGTGTGTGTAAGTATGAGCATGTTATTTCTTTTATGGGCTTCAGGTTTGACATT
AATGTGCCTGAACAGCATAGTCTTTTCTGTACCAGGAATTTTGCAATGCGCCATGTGCGTGG
TTGGCTTGGTATGGATGTAGAAGGTGCCCATGTTACAGGTGAAAATGTAGGTACTAATGTA
CCATTACAAGTAGGCTTCTCTAATGGTGTTGATTTTGTTGTCCAACCAGAAGGTTGTGTTGTC
ACTGAGACAGGCAGCAGGATCGTACCTGTTAGAGCTCGTGCTCCGCCAGGTGAACAGTTCA
CACATTTGGTCCCCCTGCTTCGTAAGGGACAACCATGGGCTGTGGTTCGTAAACGCATTGTT
CAGATGTGTTGCGATTACTTGGTGAACCTATCAGACATATTAATCTTTGTTTTGTGGGCTGGT
GGTTTGGAGTTGACTACCATGAGATATTTTGTTAAAATTGGACCAATCAAAGTTTGTGAGTG
CGGCAAAGTGGCCACTTGTTACAATAGTGTGACTCATGCATACTATTGTTTCAAGCATGCCT
TAGGGTGTGATTATTTGTACAATCCTTATGTTATTGACATTCAGCAGTGGGATTATACAGGTT
CACTTAGCCATAATCATCATGAGCATTGTAATGTACATCGCAATGAGCATGTTGCATCAGGT
GATGCTATTATGACTCGTTGTCTTGCTGTGTATGATTGCTTCGTCAAGAACGTTGATTGGTCC
ATTACTTACCCATTCATTGCTAATGAACATGCCATTAATAAGAGTGGTAGAGTTGTACAATC
TATGGTTGTCAAATCAGCTATAAAGTTATATAATCCTAAAGTAATTCATGATATTGGTAATC
CTAAAGGTATTAGGTGTGCAGTTACTGATGCACACTGGTTTTGCTATGATAAGCAGCCCCTC
AATTCCAATGTCAAGTTGCTTGAGTATGATTATATAACTCATGGTCAGATGGATGGGTTATG
CCTATTTTGGAATTGCAATGTTGACATGTATCCTGAATTTTCTATTGTGTGCCGTTTTGACAC
TCGTTGTAGGTCAAATCTCAACTTAGAAGGTGTTAATGGAGGTTCACTTTATGTCAACAACC
ATGCATTTTTTACACCTGCATTTGATAAGCGTGCAATGGCTAAACTTAAGCCTATGCCCTTCT
TTTTCTATGATGACACGCCTTGCGATCGTGTTCATGATCAAATAAATTATGTACCACTGCGT
GCTAGTACTTGTATTACAAAGTGTAATATTGGTGGTGCTGTTTGCAGTAAGCATGCCAATAT
GTTTCATGCCTATGTTAATGCATATAATACTTTCACGCAGGCTGGTTTTACTATTTGGGTTCC
TGTAACTTTTGATTTGTTCAACCTTTGGCAAACATTTGTCGAAACTAATTTGCAAGGCTTGGA
GAACATAGCGTTTAATGTCCTCAAGAAAGGGTCTTTTGTTGGTGTAGAGGGTGAACTACCG
GTTGCTATCGTTAATGACCGTGTGCTGGTACGTGATGGCACCGTGGATAATTTGATTTTTACT
AATAAAACCTCATTACCTACTAATGTTGCTTTTGAGCTTTACGCCAAACGTAAAATGGGTTT
GACACCACCTCTTGCTGTTCTCAGGAATCTCGGTGTTGTTAGTACCTATAAGTTTGTGTTATG
GGATTATGAGGCAGAAAGACCATTTACTACTTTTACTAAAAATGTTTGCAGTTACACAGAC
TTTGATGAGGATGTGTGCACTTGCTTTGATAATAGCATTCAGGGTTCGTTTGAACGCTTTTCA
ATGGCAAACAATGCAGTGCTTATTTCCACATCTGCTGTTAAGAAGCTTACTTGTATTAAGCT
TAACTATGGTTTGCTTAATGGTGTGGCTGTTTCTAAGACTGGTGACAAGCCTGTTACATGGT
ATATCTATGTCCGTAAAAATGGTCAATTTGTTGACCAATTTGACGGATATTTTACTCAGGGG
CGTACTACTATTGATTTCTTACCTCGTAGTGTTATGGAGGAAGACTTTTTAAACCTTGATATG
GGTTTGTTTATTAGTAAGTATGGTCTTGAAGACTATGCTTTTGAGCATGTTGTGTATGGTGAC
GTGTCTGGTACTACACTTGGTGGACTACATTTATTAATTTCTCAAGTACGCCTTGGAAAAAT
GGGTGTCTTGAAAGTGGATGAGTTCACACATAGTGAGGATAGTACATTGAAGTCCTGTACA
GTCACCTATTCTAATGATCCTAGTAGTAAAATGGTTTGTACCTATATGGATTTGTTGCTAGAT
GATTTTGTTACCATTTTAAAATCTTTGGATTTGGGTGTAATTTCCAGAGTTCATGAAGTTGTA
GTCGATTGTAAGGTTTGGCGTTGGATGTTGTGGTGTAAGGACCACAAAGTTCAAACGTTCTA
CCCACAGTTGCAGTCGTCAGAATGGAAATGTGGCTATTCCATGCCCTCACTGTATAAAATA
CAGCGTATGTGTCTTGAGCCCTGTAATTTGTACAATTATGGTGCTAGTCTTAAGCTACCTGA
AGGTATCATGTTTAACGTGGTCAAATACACTCAGTTATGTCAATATTTGAATAGTACTACTA
TGTGTGTACCGCATAATATGCGTGTACTACATCTTGGTGCTGGCTCAGATAAAGGAGTCGCA
CCTGGTACTGCTGTATTGCGTCGTTGGTTACCCAGTGATGCTATTATTGTTGACAATGATGTT
AATGACTATGTTAGTGATGCAGATTTCAGTGTAACAGGGGATTGCGCCACTTTGTACTTACA

GGATAAGTTCGACTTAGTGGTTTCAGACATGTATGATGGCCGTATTAAACAGTGTGATGGTG
TTAACGTTTCAAAGGATGGCTTCTTTCCTTACATCAATGGTGTGATATCAGAGAAGCTTTCC
CTAGGTGGTACAGTTGCCATTAAGATTACTGAGTATAGCTGGAATAAGCGGCTGTATGAGT
TGATACAAAAGTTTGAATTTTGGACGTTGTTTTGTACTAGTGTGAATACATCATCGTCTGAA
GCTTTTCTAATTGGTGTCAACTATCTTGGCGACTACGCCACTCAACTCATTGTGGATGGTAAT
ACTATGCACGCTAATTATGTGTTTTGGCGTAATTCTACAATTATGTCCATGTCATATAATAGT
GTGCTTGATCTAGCAAAGTTTAATTGTAGACATAAAGCTACGGTTGTTATTTCACTTAAAGA
AAGTGATTTGAATGATGTTGTGGTAGGCCTTATAAAGAATGGAAAGTTGTTGGTTAGAAAG
AGTGGTGTC
>SUOHC1012
ACAACAGCAACAGAAACAAGGGTAATTCTAAAGATGACCTTGTTGCTGCTGTACGTGAAG
CTCTTATGGGACTCGGTTTTCAATCGGAACGCAGAGGATCTGAAACTTCTGGCAAAAACAC
ACCCAAGCAACAGCGGGCTAAGTCTCCTGCAAGACCTTCACAGTCACCCAAGAGACAAGC
AGACAGGCCTGAATGGAAACGAGTGCCGAATGCTGGCAACTCTGTTACAGTCTGCTTCGGC
CCACGCGATGCGTCCCATAACTTTGGAGACGCACAATTAGTTGCAGAGGGTGTTGACTCAA
AGGGTTACCCACAGATTGCTGAACTAGTGCCAACACCAGCAGCATTGCTTTTTGGCAGTAG
GATTTCAGTTCGTGAGACACCTAATGACTTTGAAATTAAGTTTCATTACAAGATGCATGTCC
CTAAGACAGATAAAGCTCTTGCAGCATTTCTGCCTCAGGTTGATGCCTAT
>SUOHC1013
GGTACAGGCCTTATTTCACTGTTGTATAATATCTTTAACATTTTTTCAGGTTCTTTCAGTGTGA
TGGCTTTGTCTGGTCAAATCATTTTTAATTTAGTTGTAGCAACTTTAGTGGTGTTTAGCTGTTT
TATGGTTACCAAATTCAAGCGTGTCTTTGGTGACCTTTCTTTTGCAGTTTTCACAGTTTGTAC
AACTTCTTGTGTGAATAGCATTTCTTACATCATTACACAGAATTATTGTGCTATGTATGTATA
TGCTTTCTTGTATTTTATGGCCACTCGCAATATGCGTTATGCTTGGATTTGGCATAGTGGCTTT
GTGCTTGCCTATTTCACACTGGCACCATGGTGGCTTATTGTTTGGTATGTCTGTGCTACATGT
GTTGGGTTGATTCCTAGTCTTATGAAACTTAAGGTTTCTACGCAGCTTTTTGAGGGAGACAA
GTTTGTCGGCACTTTTGAGAGTGCTGCTGCAGGTACTTTTGTGATTGACATGCATTCTTATCA
GCGTCTGGTCAATAGTATTGCACCGGAGAAGCTGAAACAGTATGCTGCCAGTTACAATCGT
TATAAGTACTATAGTGGTAGCGCTAGTGAGGCTGACTACCGTCTTGCTTGTTTCGCTCACCTT
GCAAAGGCTATGTTGGACTATGGTACCAACCATCAAGACATGCTCTACACTCCTCCTACAG
TTTCGTACAATTCTACATTGCAGTCTGGTCTGCGAAAGATGGCTCAACCATCTGGTGTTGTT
GAAAAATGTGTGGTTCGTGTGAGTTATGGTAACATGACATTGAATGGTGTTTGGCTTGGTGA
CACAGTTATTTGTCCTAGACATGTTATTGCGTCATCAACCACTTCTACCATTGATTATGAAC
ATGAGGCTTCTATTATGCGTCTTCATAATTTTTCTATTGCTGTTGGTAATGTCTTTTTGGGTGT
TGTAGGTGTTTCTGTTAGAGGTTGTAATCTTCATATTAAGGTTAACCAATCTAATGTTAACAC
ACCCAATTATAGCTTTAAAACTTTGAAGGCTGGTGATTCTTTCAACATTTTAGCTTGTTATGA
TGGTGTACCTGCTGGTGTCTATGGTGTGACACTGCGTACAAATAACACTGTGCGTGGTTCAT
TTATCAATGGAGCTTGTGGTTCACCGGGTTACAATATTGTTGGAGGTACAGTCGAGTTTTGT
TACCTTCATCAGCTTGAACTTGGCAGTGGTTGTCATGTTGGTTCTAACCTTGAAGGTGTTATG
TATGGTGGTTTTGAGGATCAACCTACATTGCAAATTGAAGGCGCTAGTAATTTAGTTACTGT
AAATGTTATTTCGTTTCTTTACGGTGCACTTTTGAATGGTGTTACATGGTGGCTTACAAGTGA
GCGTGTCACCATAGATGCTTATAATGAGTGGGCTCAACAAAATGGTTTTACACAGGTTAGTT
CTGTGGATTGTTTTACCATTCTTGCAGCCAAGACTGGTGTAGACGTACAACGTATTTTAGCG
GCTGTACAGCGTTTGCACAAGGGTTTTGGTGGTAAGGCTATCCTTGGATTTACTTCTCTTACA
GATGAGTTCACTACTGCTGAGGTCATACAGCAAATGTATGGTGTCAGTCTACAGAGTGGCA
AGGTTTCCAGTACTTTCAAGAATTTGTTTCTTGTTGGTACTTTCTTTGTTATGCTGTGGTCTGA
GTTGATGATGTTTACACCATTTTTCTGGGTTAGTCCTGGATTTGTCACTCCAATTTTTGCTATG
CTCGTTTTGCTGTCAATTATGTTTACAGGTTTTATTAAACATAAAGTTTTGTACCTTTATACAT
TTTTGATTCCAGCAGTTATTGTGACGTCTTGTTGCAATTTTCTTGTTGAGCTTGCAATTAATTC
TTGGCTTGCATCTGTTCTTGATTATCACGCCTCTATTATGAGTGTTGATATTCAGGGTGTGAT
TAATGTAGTTATTTGTGCTATTGTGACTATATTGCACACGTACAGGTTTGCCAAGGCTGGTTT
TGTGACTTATGTTACATATTTTCTGTCGTTGTTTGTGGTATTTTATAATTGGTGGTTTCATGGT

GATCTGCTGAGTCTGTTTATGATGTTGCTTCTCAATGTGTCAGATAGCTGGTATGTTGGTGCT
ATTGCATACAGAATATCATTACTTGCAGCCACACAATTCCCTATGTTGGAACTTGTTGTTGG
TAAGGCCAAGGTTGTGATTTTTATTTATGTTGCAATTGGTTTTTTGTGCTGTATGTATTATGGT
GTTCTGTATTGGATTAACAGATTCTGTAAGTTGACACTTGGTGTATATGACTTTAAAGTCAGT
GCTAATGAGTTTAAGTATATGGTTGCTAACGGGTTACGTGCTCCTAGAGGTGTGTTTGACTC
AATGCTTCTGTCTATGAAACTTGTTGGTATTGGTGGTGATAAAGTTATAAAAGTTTCCACGG
TACAATCCAAACTGACAGATATCAAGTGCACTAATGTTGTTTTGCTTGGCTGTTTGTCTAGT
ATGAACATTGCAGCTAATACTAAAGAATGGGCTCATTGTGTCGAATTGCACAACAAGATTA
ATCTTTGTGATGATCCTGAAAAGGCACAAGAAATGCTTCTTGCATTGCTAGCTTTTTTTATTA
GTAAGCAAAAAGATTTTGGCATTGATGATTTGATTGATTCATACTTTGACAGTTCTAGTGTA
CTGCAGAGTGTTGCGTCTACATTCGTCAATATGCCTTCTTACATTGCTTATGAAAGTGCGCG
GCAAAGTTATGAGGATGCTGTTAATAATGGGTCCTCTCCACAGCTCATTAAGCAGCTTAAG
CGTGCTATGAATATTGCTAAAGCTGAGTTTGACCATGAGTCTTCGGTTCAAAAGAAGATTA
ATCGCATGGCCGAACAAGCTGCTGCTCAGATGTTCAAGGAAGCACGTGCTGTTAATAGAAA
GTCCAAGATTATTAGTGCTATGCACTCTTTGCTTTTTAGCATGCTTCGTCGCTTGGACATGTC
TAGTGTAGACACTATTCTTAGTCTTGCAAAAGATGGTATAGTTCCTCTGTCGGTGATCCCCG
CCACTTGTGCTACTAAACTTTCTATTGTTAGCAGTGATTTTACGTCATTTGCTAAGATTGTTA
AAGATGGTTGTGTGCACTATGCTGGTGTGGTGTGGTCGATTGCTGAAATCACTGATGCTGAT
GGTAAGATCGTACATCTTAAGGAGGTTGTCGAAGAGAACACAGATTCACTTACATGGCCAC
TCTTTGTTAGTTGCGTGCGTGTTGTGAAGCTTCAGAATAATGAGATTATTCCTGGTAAGCTTA
AACAGCGTGTTGTAAAGGCAGAGGGTGATGGTTTCAGTGTTGAAGGTAAAGCACTCTTTAA
TAATGAGGGTGGTAAAACTTTTATGTACGCTTTTGTCGCTGACAAACCAGATTTGAAATATG
TTAAGTGGGAATTTGATGGTGGTTGTAACACTATTGAACTTGAGCCACCTTGTAGGTTTGCT
GTTGAAACTGCAAATGGCACTGTTATAAAGTACTTGTATTTTGTGCGTAATCTAAATACATT
GCGCAGGGGTGCAGTTTTGGGCTTTATTGGTGCTACAGTGCGTTTGCAAGCCGGTAAACAA
ACAGAACTTGCTTCTAATTCGGCATTGCTTACTATGTGTGCTTTTGCTGTTGATCCTGCAAAG
ACGTATATTGACGCCGTTAAGAAGGGTGTTAAACCTGTTGGGAATTGCGTCAAGATGTTGTC
AAATGGTTCAGGTACTGGCCAAGCTATTACTGTTGGTGTTGAAGCTAATTCTAATCAAGATT
CTTATGGTGGCGCTTCTGTCTGTTTGTATTGCCGTGCACATGTTGAACATCCTACGATGGATG
GTGCGTGTCGTTACAAAGGCAAGTATGTTCAAGTCCCACTTGGTAATGCGGATCCTATACG
GTTCTGTTTAGAAAATGAGCCATGTAAAGTTTGTATGTGCTGGTTGAATAATGGTTGCACTT
GTGATAGAACTTCTATTCAGAGTGTCGACACGAGTTATTTAAACGAGCACGGGGCTCTAGT
GCAGCTCGACTAGAGCCCTGTAATGGAACTGACACGGATCGTGTAGTCCGTGCTTTTGACA
TTTACAATAAGGATGTTGCGTGTATTGGCAAGTTTCTTAAGGTCAACTGTGTCCGTTTTAAG
AATCTTGACAAGCATGATGCTTTCTTTGTTGTAAAACGTTGTACGAAGTCTGTCATGGAACA
CGAGCAGTCCATATATGACAAGCTTCACAGTTGTGGTGCAGTTGCAGTTCATGATTTTTTCTT
GTGGAAAGATGGCCGTTCAGTTTTTGGTAATATTAGCAGGCAAAACCTGACTAAGTATACA
ATGATGGACTTGTGTTATGCTTTGAGAAATTTTGATGAGCGTAATTGCGAAACACTTAAAGA
AATTCTTGTCCTTACTGGTTGCTGTGACTATTCATATTTTGAAAATAAGGATTGGTACGACCC
AGTTGAAAATGAGGACATTCATAAAGTTTATGCACACCTTGGAACTGTTGTCTCTAATGCAT
TGCTTAAGTGTGTTAAGCTTTGCGATGCGATGGTAGAGGCAGGTGTAGTGGGTGTGTTAACA
CTTGATAACCAAGACCTTAATGGTGATTTTTATGATTTTGGTGACTTTGCTACAACAATACCT
AAAATGGGTGTACCATTATGTACCTCTTATTACTCTTATATGATGCCTGTAATGGGTATGAC
CAATTGTCTTGCATCTGAGTGTTTTATTAAGAGTGACATTTTTGGTAGTGATTTTAAGACCTA
TGATTTGCTTGAGTACGACTTTACAGAGCACAAAGAGGCTTTGTTTCGTAAGTACTTCAAGT
ATTGGGGTCAGGATTACCACCCTAATTGTGCTGATTGTCACGACGAGCTTTGTATTGTTCATT
GTGCGAATTTTAATACGTTGTTCGCCACGACTATTCCTAATACAGCTTTTGGTCCTCTGTGTC
GCAAAGTGTTTGTTGATGGTGTGCCACTTGTTACTACAGCTGGCTACCATTTCAAGCAGTTG
GGCCTTGTTTGGAATAAGGATATAAATACTCACTCTACTCGTTTGAGTGTAAATGAGTTGTT
ACAGTTTGTCACAGACCCTGCACTTTTGATAGCATCATCACCAGCCCTTGTGGATCAGCGTA
CTGTTTGTTTTTCAGTCGCTGCCTTGAGTACTGGTATGACAAATCAGACAGTTAAGCCAGGC
CACTTTAATAAAGAGTTCTACGACTTTTTGAGATCGCAAGGATTTTTTGAAGAAGGCTCTGA

ATTAACTTTAAAGCATTTCTTCTTTGCACAGAAGGGTGATGCTGCTGTCAAAGATTTTGACT
ACTATCGTTATAATAGACCAACTATGCTAGACATTTGTCAGGCTAGAGTCACTTATAAAATA
GTGCAGCGTTATTTTGAGTGCTATGAGGGTGGCTGTATTGCCGCTAAGGATGTTGTTGTTACT
AACCTCAATAAAAGTGCAGGTTACCCACTTAATAAATTTGGTAAAGCTGGTTTGTATTATGA
GTCTCTTAGTTATGAGGAACAAGATGCACTATACGCTCTTACAAAGCGTAATGTGTTACCTA
CTATGACACAGCTTAACCTTAAATATGCAATCAGTGGTAAAGAACGTGCTAGGACTGTTGG
TGGTGTTTCATTGTTGTCTACTATGACTACTAGACAGTTTCATCAGAAACACCTTAAGTCCAT
TGTTAATACACGTAATGCATCTGTTGTCATTGGTACCACAAAATTTTATGGTGGTTGGGACA
ACATGCTTAAGAACCTTATTGATGGTGTTGACAATGCGTGCCTTATGGGTTGGGATTACCCT
AAGTGTGATCGTGCACTCCCTAATATGATTCGCATGATTTCTGCCATGATTTTGGGTTCGAA
GCATGTTACTTGCTGTGATTCTTCTGATAAATATTACCGTCTCTGTAATGAGCTCGCTCAAGT
GTTGACAGAAGTTGTATATTCTAATGGTGGGTTTTATCTCAAGCCTGGTGGTACCACTTCTG
GCGATGCTACAACGGCTTATGCGAACTCTGTGTTTAATATTTTTCAAGCTGTTAGCGCCAAT
ATTAACAGGTTGCTTAGCATTGATAGCAATGTGTGTAATAATATTGGTGTTAAGAGTTTGCA
GCGTAAGCTATATGATTGTTGTTACAGATCTTCTTCAGTAGATGATTCTTTTGTAGACAGTTA
TTATTGCTACTTGCGGAAACATTTTTCTATGATGATTTTGTCAGATGACGGTGTTGTGTGTTA
TAATAAAGATTATGCTGATTTGGGTTATGTGGCAGATATTAATGCCTTTAAAGCTACCCTAT
ATTATCAGAATAATGTCTTTATGAGTACTGCTAAATGCTGGGTGGAGCCAGACATTAGCAA
AGGTCCTCATGAGTTCTGTTCACAGCATACTATGCAGATTGTAGATTCTGACGGCAAGTATT
ATTTGCCATACCCAGACCCATCTAGAATATTGTCTGCTGGTGTATTTGTCGATGATGTTGTTA
AGACTGACGCTGTGATATTGTTGGAGCGCTACGTCTCATTGGCAATCGACGCATATCCTTTG
TCTAAACATCATAATCCTGAATATAGAAAAGTGTTTTATACACTTCTAGATTGGGTTAAGCA
TCTACACAACACACTTAATCAGGGCGTGCTTGAGTCTTTCTCCGTTACATTGCTTGAGGATG
TATCCTCTAAGTTTTGGGATGAGAGTTTTTATGCTAGTTTGTATGAAAAGTCTACAGTACTTC
AAGCCGCAGGCCTATGTGTTGTTTGTGGTTCTCAAACTGTACTGCGTTGTGGTGATTGTCTTA
GACGACCTATGTTGTGCACTAAGTGTGCTTACGACCATGTTGTCACCACGAATCATAAATTC
ATTCTGGCTATAACACCTTACGTTTGTAATGCTTCTGGTTGCACCGTTAATGATGTGACTAAG
TTGTATTTAGGAGGTCTGAGTTACTGGTGTATAGACCATAAACCTACACTGTCACTACCACT
TTGTAGTGCAGGTAATGTTTTTGGTTTGTATAAAAACTCTGCTACAGGTTCACCGGATGTAG
AGGTTTTTAATACACTTGCCACGTCAGATTGGACTGACGTTAAAGATTACAAGCTGGCTAAT
GAATGCAAGGATTCTCTTAGGCTTTTTGCGGCTGAGACTGTTAAAGCCAAAGAAGAGAGTG
TTAAATCCTCTTATGCTTGTGCTACACTTAAAGAAGTTGTTGGGCCACGTGAGTTGATTCTTA
GTTGGGAGTGCGGAAAAATTAAACCGCCACTTAACCGTAATTCAGTGTTTACGTGTTTCCAG
ATTTCTAAAGACTCTAAGTTCCAGGTTGGAGAGTTTACGTTTGAAAAATTGGATTACGGTTC
TGATGCTGTGTGTTACAAGTCTACCACCACAGTTAAATTAGTGCCTGGTATGATTTTTGTTCT
CACATCACACAATGTACAGCCGCTTAGGGCTCCCACTATTGCTAATCAAGAACGCTATGCT
TCAATTTATAAACTGCACCCTACTTTTAATATTAGTGATGCTTATGCCAATTTGGTGCCGTAC
TACCAAATGATTGGCAGACAACGAATCACTACTATTCAAGGCCCACCGGGAAGTGGCAAG
TCCCATTGTGTTATAGGTCTGGGTCTTTATTACCCAGGTGCTAGGATGGTCTTTGCGGCTTGC
TCTCATGCAGCCGTAGATTCACTTTGTGTCAAGGCTGCCACTGCTTATAGTGTAGACAAATG
CACTCGTATTATCCCTGCCCGTGCTAGGGTTGAGTGTTATAGTGGGTTTAAGGTAAATAACA
CTCAGGCACAGTACATCTTTTCAACAGTCAACGCACTCCCTGAGTGTAATGTTGACATAGTT
GTTATAGATGAAGTGTCTATGTGTACTAATTATGACCTGTCCGTTATTAACCAGCGGTTGTCT
TATAAACATATTGTTTATGTGGGTGACCCACAGCAATTACCAGCGCCACGTACCATGATCA
CTAAAGGCGTTCTCGAACCTAAGGACTATAATGTTGTAACTCAGAGGATGTGTGCTGTTGG
GCCAGATGTCTTCTTGCATAAATGTTACAGATGTCCTGCTGAGATTGTTAAAACAGTCTCAG
AGTTGGTTTATGAGAATAAGTTTGTACCTGTTCATGCTGATAGCAAACAGTGTTTTAAACTG
TTTTGTAAGGGCAATGTTCAGGTTGACAATGGTTCTAGTATTAACAGAAAGCAATTGGATAT
CGTCAAGATGTTCCTTGCTAAGAATCCACGTTGGTCTAAAGCTGTGTTTATCTCACCTTATA
ATAGTCAGAATTATGTGGCTAGTAGGGTTTTGGGTTTGCAGATTCAGACTGTTGACTCCTCT
CAAGGTAGTGAATATGACTATGTCATATACACACAGACGTCAGACACAGCACATGCTTGCA
ATGTTAATAGATTTAATGTTGCAATTACAAGAGCCAAGAAGGGTATTCTTTGTGTTATGTGT

GATCGGGAGTTGTTTGATGCTCTGAAATTTTTCGAGATCAAGCTTTCTGACTTGCAGGCTAG
TGGTGATGGCTGTGGTCTTTTTAAAGATTGTTACAGGAAAGATGATGAATTACCTCCATCTC
ATGCGGCTACCTATATGGCCTTGTCTGACAACTTTAAGGTCAATGGTGATCTTGCTGTTCAG
ATTGGCTTTAAAGGTGTGTGTAAGTATGAGCATGTTATTTCTTTTATGGGCTTCAGGTTTGAT
ATTAATGTGCCTGAACAGCATAGTCTTTTCTGTACCAGGAATTTTGCAATGCGCCACGTGCG
TGGTTGGCTTGGTATGGATGTAGAAGGTGCCCACGTTACAGGTGAAAATGTAGGTACTAAT
GTACCATTACAAGTAGGCTTCTCTAATGGTGTTGATTTTGTTGTCCAACCGGAAGGTTGTGTT
GTCACTGAGACAGGCAGCAGGATCGTACCTGTTAGAGCTCGTGCTCCGCCAGGTGAACAGT
TCACACATTTGGTCCCCCTGCTTCGTAAGGGACAACCGTGGGCTGTGGTTCGTAAACGCATT
GTTCAGATGTGTTGCGATTACTTGGTGAACCTATCAGACATATTAATCTTTGTTTTGTGGGCT
GGTGGTTTGGAGTTGACTACCATGAGATATTTTGTTAAAATTGGACCAAGCAAAGTTTGTGA
GTGCGGCAAGGTGGCCACTTGTTACAATAGTGTGACTCATGCATACTACTGTTTCAAGCATG
CCTTAGGGTGTGATTATTTGTACAATCCTTATGTTATTGACATTCAGCAGTGGGATTATACAG
GTTCACTTAGCCATAATCATCATGAGCATTGTAATGTACATCGCAATGAGCATGTTGCATCT
GGTGATGCTATTATGACTCGTTGTCTTGCTGTGTATGATTGCTTCGTCAAGAACGTTGATTGG
TCCATTACTTACCCATTCATTGCTAATGAACATGCCATTAATAAGAGTGGTAGAGTTGTGCA
ATCTATGGTTGTCAAAACAGCTATAAAGTTATATAATCCTAAAGTAATTCATGATATTGGTA
ATCCTAAAGGTATTAGGTGTGCAGTTACTGATGCACACTGGTTTTGCTATGATAAGCAGCCC
CTCAATTCCAATGTCAAGTTGCTTGAGTATGATTATATAACTCATGGTCAAATGGATGGGTT
ATGCCTATTTTGGAATTGCAATGTTGACATGTATCCTGAATTTTCTATTGTGTGCCGTTTTGA
CACTCGTTGTAGGTCAAATCTCAACTTAGAAGGTGTTAACGGAGGTTCACTTTATGTCAACA
ATCATGCATTTTTTACACCTGCATTTGATAAGCGTGCAATGGCTAAACTTAAGCCTATGCCC
TTCTTTTTCTATGATGACACGCCTTGCGATCGTGTTCATGACCAGATAAATTATGTACCACTG
CGTGCTAGTACTTGTATTACAAAGTGTAATATTGGTGGTGCTGTTTGCAGTAAGCATGCCAA
TATGTTTCATGCCTATGTTAATGCATATAATACTTTCACGCAGGCTGGTTTTACTATTTGGGT
TCCTGTAACTTTTGATTTGTTTAACCTTTGGCAAACATTTGTCGAAACTAACTTGCAAGGCTT
GGAGAACATAGCGTTTAATGTCCTCAAGAAAGGGTCTTTTGTTGGTGTAGAGGGTGAACTA
CCGGTTGCTATCGTTAATGACCGTGTGCTGGTACGTGATGGCACCGTGGATAATTTGATTTTT
ACTAATAAAACCTCATTACCTACTAATGTTGCTTTCGAGCTTTACGCCAAACGTAAAATGGG
TTTGACACCACCTCTTGCTGTTCTCAGGAATCTCGGTGTTGTTAGTACCTATAAGTTTGTGTT
ATGGGATTATGAGGCAGAAAGACCATTTACTACTTTTACTAAAAATGTTTGCAGTTACACA
GACTTTGACGAGGATGTGTGCACTTGCTTTGATAATAGCATTCAGGGTTCGTTTGAACGCTT
TTCAATGGCTAACAATGCAGTGCTTATTTCCACATCTGCTGTTAAGAAGCTTACTTGTATTA
AGCTTAACTATGGTTTGCTTAATGGTGTGGCTGTTTCTAAGACTGGTGACAAGCCTGTTACA
TGGTATATCTATGTCCGTAAAAATGGTCAATTTGTTGACCAATTTGACGGATATTTTACTCA
GGGGCGTACTACTATTGATTTCTTACCTCGTAGTGTTATGGAGGAAGACTTTTTAAACCTTG
ATATGGGTTTGTTTATTAGTAAGTACGGTCTTGAGGACTATGCTTTTGAGCATGTCGTGTATG
GTGACGTGTCTGGTACTACACTTGGTGGACTACATTTATTAATTTCTCAAGTACGCCTTGGA
AAAATGGGTGTCTTGAAAGTGGATGAGTTCACACATAGTGAGGATAGTACATTGAAGTCCT
GTACAGTCACCTATTCTAATGATCCTAGTAGTAAAATGGTTTGTACCTATATGGATTTGTTGC
TAGATGATTTTGTTACCATTTTAAAATCTTTGGATTTGGGTGTAATTTCTAGAGTTCATGAAG
TTGTAGTCGATTGTAAGGTTTGGCGTTGGATGTTGTGGTGTAAGGACCACAAAGTTCAAACG
TTCTACCCACAGTTGCAGTCGTCAGAATGGAAATGTGGTTATTCCATGCCCTCACTGTATAA
AATACAGCGTATGTGTCTTGAGCCCTGTAATTTGTACAATTATGGTGCTAGTCTTAAGCTAC
CTGAAGGTATCATGTTTAACGTGGTCAAATACACTCAGTTATGTCAATATTTGAATAGTACT
ACTATGTGTGTACCGCATAATATGCGTGTACTACATCTTGGTGCTGGCTCAGATAAAGGAGT
CGCACCTGGTACTGCTGTATTGCGTCGTTGGTTACCCAGTGATGCTATTATTGTTGACAATG
ATGTTAATGACTATGTTAGTGATGCAGATTTCAGTGTAACAGGGGATTGCGCCACTTTGTAC
TTACAGGATAAGTTCGACTTAGTGGTTTCAGACATGTATGATGGCCGTATTAAACAGTATGA
TGGTGTTAATGTTTCAAAGGATGGCTTCTTTCCTTACATCAATGGTGTGATATCAGAGAAGC
TTTCCCTAGGTGGTACAGTTGCCATTAAGATTACTGAGTATAGCTGGAATAAGCGGCTGTAT
GAGTTGATACAAAAGTTTGAATTTTGGACGTTGTTTTGTACTAGTGTGAATACATCATCGTCT

GAAGCTTTTCTAATTGGTGTCAACTATCTTGGCGACTACGCTACTCAGCCCATTATGGATGG
CAATACTATGCACGCTAATTATGTGTTTTGGCGTAATTCTACAATTATGTCCATGTCATATAA
TAGTGTGCTTGATCTAGCAAAGTTTAATTGTAGACATAAAGCTACGGTTGTTATTTCACTTA
AAGAAAGTGATTTGAATGATGTTGTGGTAGGCCTTATAAAGAATGGAAAGTTGTTGGTTAG
AAAGAGTGGTGTCATTTGTGACTATGGTAACCATTTAGTTTCAACTAAATAA
>SYRL007
GATGGTGTTGTTTGTTATAATAAGGAGTATGCGGCTTTAGGTTATGTTGGTGATATATCAGC
ATTTAAAGCTACTCTTTATTATCAAAACAATGTTTTTATGAGCACATCTAAGTGTTGGGTAG
AAGAAGACCTTAGTGTCGGACCACATGAATTCTGTTCACAACACACAATGCAAATTGTTGA
TGCTGATGGTACATATTATCTCCCATACCCAGATCCTAGTAGAATTATTAGTGCTGGTGTTTT
TGTGGATGATGTTGTGARAACTGATCCTATTATCTTGTTAGAACGTTATGTGTCACTGGCAAT
AGATGCATACCCACTTTCTAAGCATCCCAACAGAGAGTACCGTAAGGTTTTCTATGTTCTGT
TGGATTGGGTTAAGCATCTGCACAACACACTTAATGAAGGCATTCTTGAAAC
