# Supplementary material for: A Novel Coronavirus and a Broad Range of Viruses in Kenyan Cave Bats
Source: Viruses. 2022 Dec 17;14(12):2820. doi: 10.3390/v14122820 (PMC9785147; doi:10.3390/v14122820)
Supplement: Supplementary file 1 [file viruses-14-02820-s001.zip › TableS2.pdf]

Supplementary Table S2: Overview of the coronavirus contigs obtained in positive samples

Location	Sample	Read count		Contigs										
Site 2 (Soysambu Conservancy)	SYRL007	145		Contig 1 (425bp)										
			Region	ORF1ab										
			Range	1-425										
			ID	94.33%										
Site 3 (Mount Suswa)	SUOHC1001	1067		Contig 1 (5329bp)										
			Region	ORF1ab										
			Range	1-5329										
			ID	98.45%										
	SUOHC1004	8673		Contig 1 (11893bp)	Contig 2 (809bp)	Contig 3 (1235bp)			Contig 4 (476bp)	Contig 5 (610bp)				
			Region	ORF1ab	S partial	E partial	M complete	N partial	N partial	N partial	ORFx complete			
			Range	1-11893	1-809	1-39	49-729	740-1235	1-476	1-123	137-367			
			ID	93.06%	65.93%	75.00%	94.25%	83.83%	88.59%	84.85%	66.67%			
	SUOHC1005	52196		Contig 1 (14647bp)	Contig 2 (847bp)	Contig 3 (571bp)		Contig 4 (1235bp)			Contig 5 (476bp)	Contig 6 (612bp)		
			Region	ORF1ab	S partial	NS3B partial	E partial	E partial	M complete	N partial	N partial	N partial	ORFx complete	
			Range	1-14647	1-847	1-543	524-571	1-39	49-729	740-1235	1-476	1-123	137-367	
			ID	91.27%	64.89%	74.43%	75.00%	75.00%	94.25%	83.83%	88.59%	84.85%	66.67%	
	SUOHC1012	45		Contig 1 (476bp)										
			Region	N partial										
			Range	1-476										
			ID	88.59%										
	SUOHC1013	34507		Contig 1 (12065bp)	Contig 2 (525bp)	Contig 3 (1107bp)			Contig 4 (446bp)	Contig 5 (611bp)				
			Region	ORF1ab	NS3B partial	E partial	M complete	N partial	N partial	N partial	ORFx complete			
			Range	1-12065	1-525	1-36	46-726	736-1107	1-446	1-123	137-367			
			ID	93.09%	74.29%	72.73%	93.81%	83.74%	81.76%	81.82%	66.67%			
	SUOHC2001	1037		Contig 1 (8048bp)	Contig 2 (781bp)									
			Region	ORF1ab	S partial									
			Range	1-8048	1-781									
			ID	97.65%	65.42%									
	SUOHC2004	171		Contig 1 (865bp)										
			Region	ORF1ab										
			Range	1-865										
			ID	98.95%										
	SUOHC3001	22943		Contig 1 (14666bp)	Contig 2 (539bp)	Contig 3 (1235bp)			Contig 4 (476bp)	Contig 5 (608bp)				
			Region	ORF1ab	NS3B partial	E partial	M complete	N partial	N partial	N partial	ORFx complete			
			Range	1-14666	1-539	1-39	49-729	740-1235	1-476	1-122	136-366			
			ID	91.41%	73.74%	75.00%	94.25%	83.83%	88.59%	87.10%	66.67%			
	SUOHC3002	11893		Contig 1 (8048bp)	Contig 2 (809bp)	Contig 3 (557bp)		Contig 4 (1831bp)			Contig 5 (612bp)			
			Region	ORF1ab	S partial	NS3B partial	E partial	E partial	M complete	N partial	N partial	ORFx complete		
			Range	1-8048	1-809	1-548	549-557	1-39	49-729	740-1831	1-123	137-372		
			ID	97.76%	65.93%	74.30%	75.00%	75.00%	94.25%	79.40%	84.85%	66.67%		
	SUOHC3004	12100		Contig 1 (11908bp)	Contig 2 (970bp)	Contig 3 (485bp)	Contig 4 (1197bp)	Contig 5 (2274bp)					Contig 6 (443bp)	Contig 7 (413bp)
			Region	ORF1ab	S partial	S partial	S partial	Spartial	NS3 complete	E complete	M complete	N partial	N partial	ORFx partial
			Range	1-11908	1-970	1-485	1-1197	1-353	352-1040	989-1216	1226-1096	1917-2274	1-443	1-164
			ID	94.57%	62.97%	72.05%	79.35%	79.31%	70.37%	73.61%	94.25%	85.71%	82.19%	66.67%
	SUOHC3005	30		Contig 1 (413bp)										
			Region	ORF1ab										
			Range	1-413										
			ID	96.38%										
	SUOHC3007	226		Contig 1 (1133bp)										
			Region	ORF1ab										
			Range	1-1133										
			ID	98.67%										

Identities are provided according to the closely-related virus sequences and based on nucleotide BLAST results. (bp: base pairs).
