# Supplementary material for: A Novel Coronavirus and a Broad Range of Viruses in Kenyan Cave Bats
Source: Viruses. 2022 Dec 17;14(12):2820. doi: 10.3390/v14122820 (PMC9785147; doi:10.3390/v14122820)
Supplement: Supplementary file 1 [file viruses-14-02820-s001.zip › TableS3.pdf]

Supplementary Table S3: Overview of the of the retrovirus contigs obtained in positive samples.

Location	Sample	Virus	Target region	Longest contig	Read count	ID
Menengai Crater	MEMT001	MMER	nsp	663bp	6949	98.94%
		MRER	pol	270bp	4	93.81%
	MEMT002	MMER	nsp	663bp	662	98.79%
	MEMT003	MMER	nsp	663bp	4068	98.94%
	MEMT004	MMER	nsp	663bp	164	99.09%
	MEMT005	MMER	nsp	663bp	5864	98.94%
	MEMT006	MMER	nsp	543bp	15	98.52%
	MEMT007	MMER	nsp	663bp	4232	98.94%
		MDER	nsp	201bp	10	86.56%
	MEMT008	MMER	nsp	663bp	3951	98.94%
	MEMT009	MMER	nsp	563bp	93	98.57%
Soysambu Conservancy	SYMN003	RFRV	gag-pol-env	8363bp	3318	97.07%
	SYRL001	RFRV	env	1619bp	265	95.39%
	SYRL002	RFRV	pol-env	2897bp	396	95.47%
	SYRL004	RFRV	env	906bp	110	95.41%
	SYRL005	RFRV	pol	409bp	315	93.64%
	SYRL006	RFRV	env	1877bp	257	92.31%
	SYRL007	RFRV	pol	609bp	145	94.83%
	SYRL008	RFRV	pol-env	822bp	150	95.14%
	SYRL009	RFRV	pol	1551bp	275	98.19%
	SYMA002	RFRV	pol-env	350bp	62	97.42%
Mount Suswa	SUOHC1001	GERV	gag	223bp	333	74.55%
		MLRV	pol	533bp	32	89.49%
	SUOHC1002	GERV	gag	223bp	182	74.55%
	SUOHC1003	GERV	gag	151bp	6	76.32%
	SUOHC1004	GERV	5' LTR	204bp	167	64.07%
		MLRV	pol	390bp	31	90.00%
	SUOHC1005	GERV	gag-pol	401bp	686	71.78%
		MLRV	pro-pol	754bp	31	87.78%
	SUOHC1006	GERV	gag	223bp	232	75.00%
		MLRV	pol	434bp	16	87.78%
	SUOHC1007	GERV	gag-pol	394bp	996	71.78%
		MLRV	pro-pol	941bp	56	91.22%
	SUOHC1011	GERV	5' LTR	282bp	262	60.49%
		MLRV	pro-pol	912bp	30	88.48%
	SUOHC1013	RAFV1	pol	220bp	933	76.81%
	SUOHC1014	GERV	gag	223bp	55	74.55%
	SUOHC2001	GERV	5' LTR	190bp	271	60.82%
		MLRV	pro-pol	947bp	45	92.71%
	SUOHC2002	GERV	5' LTR	191bp	129	59.27%
		MLRV	pol	395bp	74	90.12%
	SUOHC2004	GERV	5' LTR	281bp	257	58.33%
		MLRV	pro-pol	927bp	63	92.88%
	SUOHC3001	GERV	pol	460bp	667	71.98%
		MLRV	pro-pol	324bp	24	91.35%
	SUOHC3002	GERV	gag	249bp	138	70.38%
		MLRV	pro-pol	426bp	15	86.65%
	SUOHC3003	GERV	gag	272bp	364	71.41%
		MLRV	pro-pol	925bp	45	93.41%
	SUOHC3004	GERV	5' LTR	242bp	1161	60.97%
		MLRV	pro	261bp	19	92.72%
	SUOHC3005	GERV	5' LTR	192bp	103	59.79%
	SUOHC3006	GERV	pol	338bp	590	75.74%
		MLRV	pol	484bp	30	91.39%
	SUOHC3007	GERV	gag	257bp	209	70.91%
		MLRV	pro-pol	899bp	23	89.85%
	SUOHC3008	MLRV	pro-pol	913bp	32	90.25%

MMER: *Myotis myotis* endogenous retrovirus; MRER: *Myotis ricketti* endogenous retrovirus; MDER: *Myotis daubentonii* endogenous retrovirus; RFRV: *Rhinolophus ferrumequinum* retrovirus; GERV: *Galidia* endogenous retrovirus; MLRV: murine leukemia virus-related virus. Identities are provided according to the closely-related virus sequences and based on nucleotide BLAST results. (bp: base pairs)
