# Supplementary material for: A Novel Coronavirus and a Broad Range of Viruses in Kenyan Cave Bats
Source: Viruses. 2022 Dec 17;14(12):2820. doi: 10.3390/v14122820 (PMC9785147; doi:10.3390/v14122820)
Supplement: Supplementary file 1 [file viruses-14-02820-s001.zip › TableS4.pdf]

Supplementary Table S4: Overview of the astrovirus and miscellaneous virus contigs obtained in positive samples

Location	Sample	Virus	Target region	Longest contig	Read count	ID
Menengai Crater	MEMN002	Bat astrovirus	ORF1ab	1194 bp	48	82.15%
	MEMN006	Bat astrovirus	ORF1ab	940 bp	249	84.61%
		Rhopalosiphum padi virus	ORF1	270 bp	4	96.29%
	MEMN009	Actinidia yellowing virus 2	Coat protein	481 bp	83	84.94%
Soysambu Conservancy	SYMA003	Bat astrovirus	ORF1ab	2181bp	168	82.48%
	SYMA004	Bole tick virus 4	Polyprotein	292 bp	14	99.31%
	SYMA006	Bat astrovirus	ORF1ab	1327 bp	203	78.15%
	SYMA008	Bat astrovirus	ORF1ab	2189 bp	185	80.14%
		Bat herpesvirus	Helicase/primase	381 bp	34	81.62%
	SYMA009	Bat astrovirus	ORF1ab	533 bp	13	78.99%
	SYMA010	Bat astrovirus	ORF1ab	1178 bp	1499	82.05%
	SYRL004	Bat astrovirus	Capsid precursor	380 bp	12	77.10%
	SYRL007	Bat astrovirus	ORF1ab	235 bp	8	75.21%
	SYMN007	Bat astrovirus	ORF1ab	2153 bp	1021	78.84%
	SYMN010	Bat astrovirus	ORF1ab	1172 bp	27	80.90%
	SYMN011	Bat astrovirus	ORF1ab	377 bp	14	76.92%
		Cyclovirus	Rep	1052 bp	212	90.02%
	SYMN012	Bat astrovirus	ORF1ab	2227 bp	2239	79.41%

Identities are provided according to the closely-related virus sequences and based on nucleotide BLAST results. (bp: base pairs).
