# Supplementary figures and images for: A Novel Coronavirus and a Broad Range of Viruses in Kenyan Cave Bats
Source: Viruses. 2022 Dec 17;14(12):2820. doi: 10.3390/v14122820 (PMC9785147; doi:10.3390/v14122820)

Tree scale: 0.25

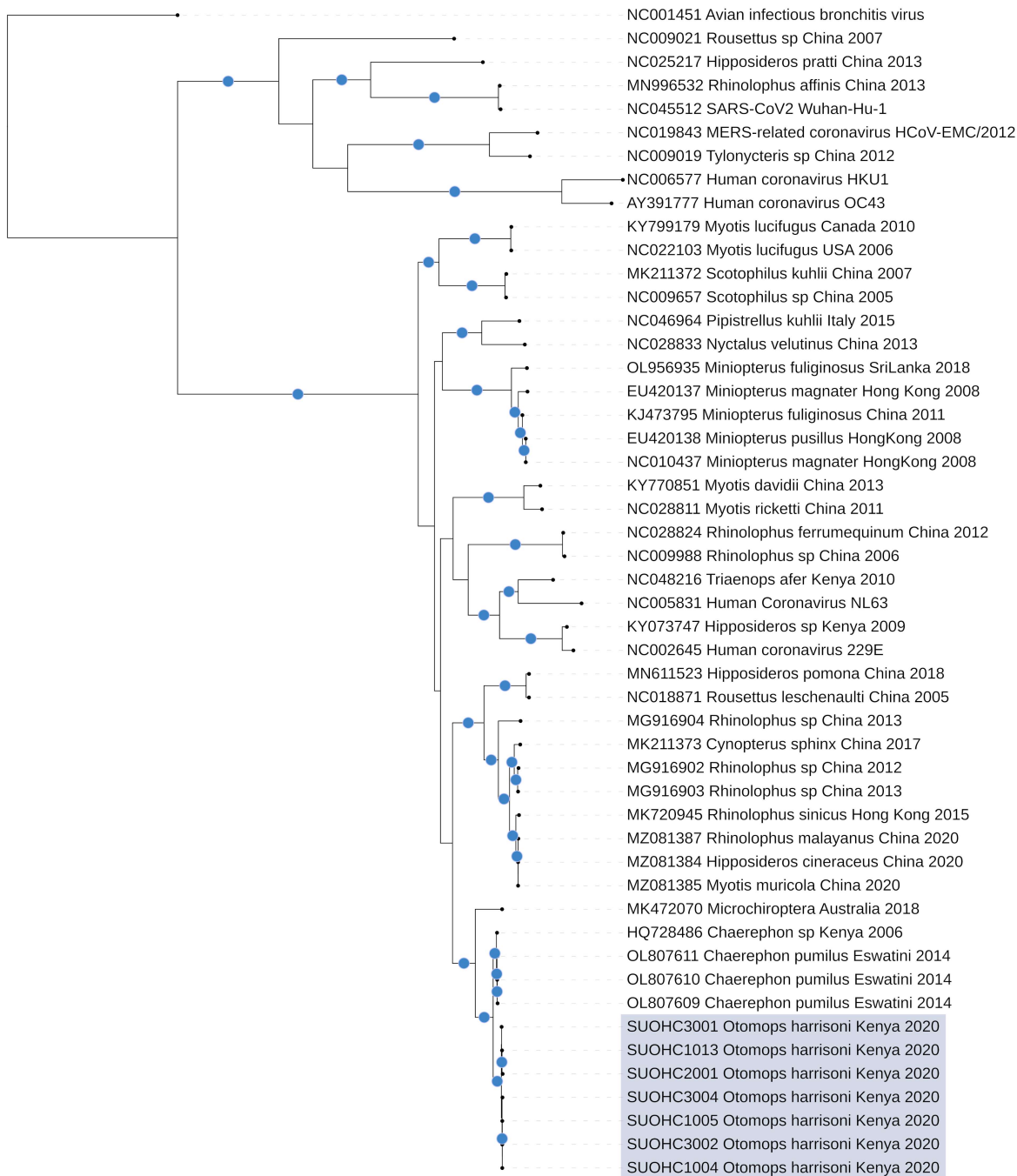

Alpha coronavirus

Supplement: Supplementary file 1 [file viruses-14-02820-s001.zip › FigureS1.pdf]
